# The theoretical investigation of the stability and the racemization of pristine, functionalized, and doped expanded helicenes

**DOI:** 10.55730/1300-0527.3752

**Published:** 2025-07-22

**Authors:** Bedirhan ÖZTÜRK, Berkay SÜTAY

**Affiliations:** Department of Chemistry, Faculty of Science and Letters, İstanbul Technical University, İstanbul, Turkiye

**Keywords:** Helicene, expanded helicene, Möbius molecule, topology, polyaromatic hydrocarbon

## Abstract

The Hückel aromatic and Möbius aromatic compounds, consisting of topologically different arrangements of adjacent benzene rings in the polycyclic aromatic hydrocarbon (PAH) family, have long attracted interest in both synthesis and theoretical chemistry. Among these, [n]-azene and [n]-circulene derivatives are more thoroughly explored due to their simple architectures and more accessible synthesis pathways. However, there are active ongoing studies on the synthesis of both [n]-helicene and expanded [n]-helicene structures, as well as Möbius circulenes, which are Möbius isomers of [n]-circulenes. In this regard, there is limited information available on both the experimental and theoretical characterization of the synthesized molecules. In this study, we employed Density Functional Theory (DFT) to investigate the thermodynamic stabilities, electronic structures, and optical and molecular properties of both synthesized and hypothetical expanded [n]-helicenes and Möbius [n+1]-circulenes. Particular emphasis was placed on the racemization barriers—an essential and tunable parameter in the design of functional chiral materials and their molecular properties. The racemization energies were computed for pristine, functionalized, and heteroatom-doped variants of expanded [n]-helicenes. Given their critical role in determining configurational stability, the racemization barriers are crucial for the material applications of functional chiral molecules and especially relevant to applications involving [n]-helicenes or helicene-like structures.

## 1. Introduction

Polyaromatic hydrocarbons (PAHs) have played an important role in the development of organic electronic devices in recent years due to their exemplary optical, electrical, and magnetic properties. These compounds are synthesized using well-known organic synthetic chemistry tools. Recently, the synthesis, isolation, and characterization of large molecules composed of fused aromatic rings, as exemplified by graphene sheets, nanorods, and fullerenes, has encouraged chemists and physicists to create and study smaller fragmented molecules. These molecules include planar and nonplanar graphenes, buckybowl structures, nanobelt structures, nanographenes, [n]-helicenes, and [n]-circulenes, while beyond these conjugated systems, there are also molecules in which the aromatic rings are bonded but not fused, such as cycloparaphenylenes [1]. All these new generation small polycyclic aromatic molecules can offer interesting optical and electrical properties, especially as semiconductors.

As molecular architectures that have been studied with interest by both chemists and physicists for decades due to their three-dimensional structure, “geodesic arenes” are important not only for their mathematical significance in terms of their topological properties, but also for their physical properties. Although the synthesis of these compounds is difficult, valuable results are obtained for both synthesis and characterization stages, especially when theoretical calculations are supported by pre-experimental studies and a priori predictions. Efforts to explore new frontiers of chemical synthesis have enabled such molecules to transform from what were once proposals or theoretical models into tangible substances. In particular, conjugated molecules exhibiting Möbius topology constitute an intriguing family of molecules due to their surprising structures and unique aromaticity. In order to determine aromaticity in ring-conjugated molecules showing Möbius conformation, the rule of having 4n number of delocalized electrons in π ring systems was proposed, in contrast to the Hückel rule [2,3]. This pioneering prediction was confirmed in 2003 with the obtainment of the first synthetic Möbius aromatic compound [4]. Recently, significant progress has been made in the synthesis of Möbius-type molecules, and a number of extended porphyrin derivatives have been isolated [5]. In particular, a family of molecules consisting of 12 fused phenyl rings has attracted attention since the mid-1950s. The aromaticity of the molecule Kekulene (C_48_H_24_), which was synthesized in 1978 as the first member of this family of compounds, has been frequently discussed, and a recent atomic force microscope study suggested that Kekulene consists of alternating benzene rings and nonaromatic rings [6]. Nanobelt was synthesized in 2017 [7]. It is a cyclophenacene in which the rings are arranged alternately in a cata-synthetic and peri-fusion pattern. Its fully kata-synthetic fusion analog is kata-[12]-circulene. Theoretical calculations show that this molecule has a saddle-like geometry. The 12 rings of cycloazine are joined in a para fashion. This molecule, like kata-[12]-circulene, has not yet been prepared, although this family of molecules was proposed as far back as 1954. These four molecules have very different geometries: planar (kekulene), ribbon (nanobelt and cycloazene), and saddle geometry (kata-[12]-circulene). In 2021, the synthesis of infinitene (C_48_H_24_), in which 12 adjacent phenyl rings form the infinite symbol, was reported [8]. This molecule can be thought of as a strip that has been cut, twisted one end one complete turn, and rejoined. The synthesis of this structure inspired the search for a Möbius form consisting of 12 fused phenyl rings [9]. The main difference between the Möbius topology (half-turn, single-surface strip) and the Hückel topology is that it involves a half-turn: a strip is cut, one end is bent by a half-turn, and then the ends of the strip are rejoined. Molecular graphs of Möbius systems cannot be drawn in a plane without their edges intersecting.

Other molecules from the PAH class, the one-dimensional azenes (metafused benzene rings), two-dimensional phenazenes, and three-dimensional helicenes (ortho-fused benzene rings), are three classes of isomeric benzenoid hydrocarbons that differ in the form of cyclization (annelation). The importance of helicenes and applications across these species has been summarized in a recent review [10,11]. In particular, the skeleton of a helix consists of ortho-fused aromatic rings, and due to steric hindrance between the terminal rings, its backbone twists in opposite directions, forming a helical structure. In this regard, the most important stereodynamic feature of [n]-helicenes is the interconversion of their enantiomers. The Gibbs activation energy value of this process determines the speed of the enantiomer isolation process and affects the configurational stability of the [n]-helicenes. Racemization of helices was first discovered in 1956 when a partial racemization was observed while determining the melting point of the [6]-helicene [12]. Later, Martin and Marchant analyzed the kinetics for the thermal racemization of a series of [n]-helicenes (n = 6–9) in naphthalene and determined the corresponding activation barriers to be 35 kcal/mol for n = 6 and about 41 kcal/mol for the remaining helicenes [13]. For the case of the [5]-helicene, Lindner proposed a mechanism involving a planar transition state (TS) with C_2V_ symmetry [14]. However, for [6]-helicene or longer helicenes, a planar TS is impossible due to the resulting steric hindrances. The author found that the racemization of the [6]-helicene involves a C_s_ symmetry TS that is nonplanar and nonchiral. When the racemization process of small [n]-helicenes (n = 4–8) was investigated theoretically by the Density Functional Theory (DFT) method, the calculated barrier values were consistent with the experimental values, and it was reported that the barriers increased up to n = 6 and formed a plateau in the 40–45 kcal/mol band for n > 6 [15]. This increase up to n = 6 has been attributed to strong intramolecular interactions between the terminal loops in the transition state (TS). The same barriers were recalculated with the DFT method, but with a different functional and including the [9]-helicene as well, and the calculated barriers were significantly improved [16]. The barrier of the [9]-helicene is also on the same plateau (40.8 kcal/mol), indicating that the additional rings add less steric energy in TS than in the helical structure.

In addition, [n]-helicenes exhibit significant differences in terms of their chemical and physical properties because they present chirality as a result of their molecular geometry. While azenes are excellent semiconductors but are difficult to isolate due to their reactivity, especially in the presence of light and oxygen, [n]-helicenes have distinct cryooptical properties and high chemical stability, which can be explained by the Clar sextet number. Another important issue regarding helicenes is their stereodynamic properties, and their predicted configurational stability can be expressed in terms of the Gibbs activation energy of enantiomerization. This barrier value determines whether the compound can be separated into enantiomers under ambient conditions and provides initial information about the configurational stability of the compound under study. Therefore, to access chiral functional materials based on [n]-helicenes, it is necessary to design molecules with high activation free energy. In addition to the nonlinear optical properties of [n]-helicene structures [17], these structures have also found application in organocatalysis [18], chirality detection [19], chemical detector [20], and hetero-atom substitution [21–24].

Although the chemistry and physics of [n]-helicenes are well-developed, their applications still remain limited to the laboratory scale. New functional chiral molecules that are resistant to enantiomerization, with both high charge carrier mobility and fluorescence quantum yield, are needed to use the results of these studies in daily applications. The existence of expanded [n]-helicenes was only reported in 2018 with the synthesis of the expanded [13]-helicene, and therefore there is only limited information on these molecules in the literature. This study aims to contribute to the literature by presenting theoretical results on the racemization mechanism of the expanded helicene and the factors affecting the configurational stability in order to facilitate the designing process of these new expanded [n]-helicene molecules.

## 2. Computational details

In the present work, the geometry optimizations of all studied molecules were performed by using DFT method in Gaussian ’16 suite [25]. Calculations were carried out by using B3LYP functional in a balanced double-zeta 6–31G(d,p) basis. This functional was selected for all calculations, because its mean absolute deviation in the racemization barriers of helicenes was found to be lower compared to the Minnesota functional and almost comparable to its dispersion-corrected counterpart, i.e. B3LYP-D3 functional [26]. The initial structures of expanded [n]-helicenes were directly adapted from their experimentally known and/or proposed 3D molecular structures as a family of PAH compounds. The convergence at a minimum energy structure was confirmed by no imaginary frequency on a frequency analysis. The convergence criteria were used as 10^−8^ Hartrees threshold in energy, 10^−4^ Hartrees/Bohr threshold in root mean square (RMS) force and 10^−4^ Hartrees/Radians threshold in maximum force. The same integration grid (ultrafine) was used in all computations in order for the calculated energies and molecular properties to be comparable. That may also help reducing the numerical instability in DFT calculations.

The transition state structures were initially guessed from a TS structure for n = 13 member in reference 29. The experimental activation barrier data were collected at room temperature, that is why the activation barriers and the related thermochemical data were all calculated at room temperature. Since no expanded [n]-helicenes were synthesized, except for the pristine n = 13 and OMe-functionalized n = 17 members, there is no specific and common solvent employed in the present study. Therefore, considering that we made theoretical predictions for pristine, functionalized, and doped members of the series for n = 11–16, all calculations were performed under solvent-free conditions to ensure the consistency and comparability of the results. The transition state (TS) structures were modeled (see supporting information), and the existence of a single imaginary frequency was confirmed. The Hessian matrix was calculated explicitly once at the beginning of the computation which was followed by the updated scheme of Berny optimization algorithm. Time-dependent DFT method (TD-DFT) was used to compute the circular dichroism (CD) spectra of racemic components at the same level of theory.

## 3. Results and discussion

### 3.1. Expanded helicenes

According to IUPAC, the number of rings (n) in helix structures starts from n = 5 [27]. It is known that helicene molecules with n values ranging from 5–14 have been synthesized under laboratory conditions, and recently, the synthesis of the [16]-helicene has been reported [28]. The existence of expanded [n]-helicenes was reported in 2018, and it is known that only expanded [13]-helicene has been synthesized under laboratory conditions [29]. Expanded helicenes with n = 15 and 17 have been synthesized with -OMe functionalization but could not be obtained in pristine form [30]. [n]-helicenes consist of n number of ortho-fused rings (ooo…o) while in the expanded [n]-helicenes with the same number of rings, the first two rings are ortho-fused, and the remaining rings follow an alternating ortho-meta arrangement (oo-omomo…). Thus, a broader 3D structure becomes possible compared to the helicenes structures.

**Figure 1:**
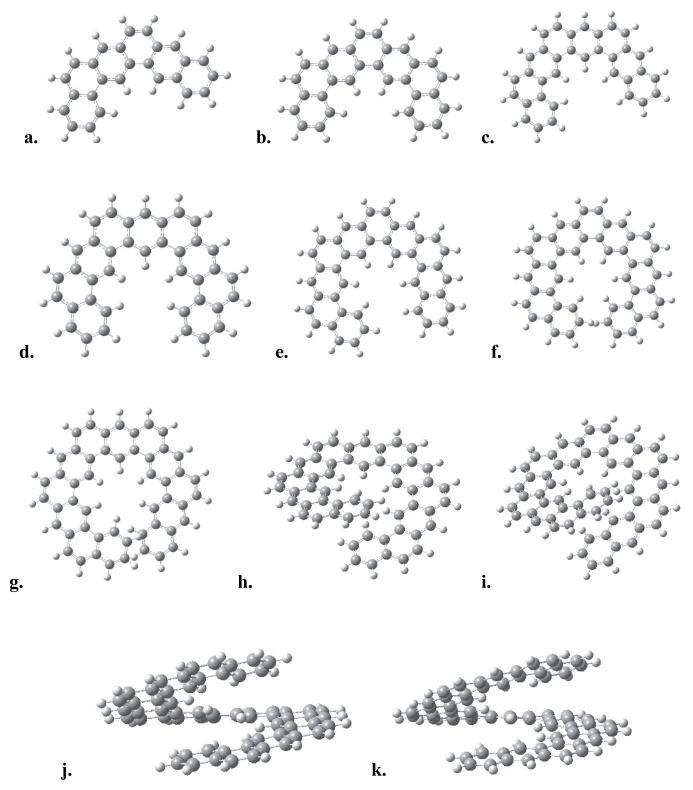
The optimized geometries of expanded [n]-helicenes for n equals a. 6, b. 7, c. 8, d. 9, e. 10, f. 11, g. 12, h. 13, i. 14, j. 15, k. 16.

For a given number of n, the number of rings fused in the ortho- and meta-directions in an expanded [n]-helicene structure—with respect to its odd and even integer values—are shown in Table 1. Such a classification is a direct consequence of the topology of expanded [n]-helicenes. The optimized geometries of expanded helicene structures were shown in Figure 1. For n values between 6 and 10, the three-dimensional geometries are planar, while the geometries starting from n = 11 exhibit a helical structure.

**Figure 2:**
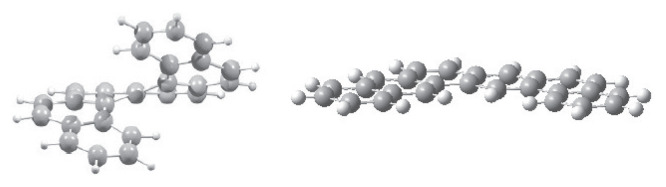
[6]-helicene (left) and expanded [6]-helicene (right).

The [5]- and [6]-helicene molecules are not planar and exhibit a tendency to form helices even in the smallest values of n, whereas the expanded [6]-helicene structure is planar as shown in Figure 2.

The benzene rings at two opposite ends of the molecules begin to stack vertically for the values n > 12, Figure 1h–1k. The calculated HOMO-LUMO energy gaps, molecular surface areas, and the molecular surface area-to-volume ratios (A/V) of the expanded [n]-helicenes, for n: 6–16, are given in Table 2.

**Figure 3:**
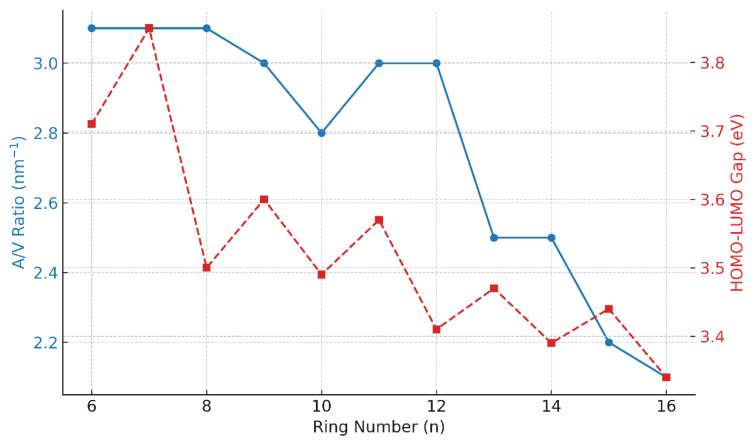
The HOMO-LUMO gap and the surface are-to-volume ratio (A/V) of expanded [n]-helicenes.

The HOMO-LUMO energy gap of expanded [n]-helicenes exhibits an alternating trend, as shown in Table 2. The highest available gap value (3.7 eV) gradually decreases with increasing n, reaching a value of 3.3 eV, as shown in Figure 3. To assess the statistical reliability and reproducibility of the calculated energy gap values across the expanded [n]-helicene series (n = 6–16), several statistical descriptors were computed and analyzed. The mean energy gap value was determined to be 3.52 eV with a standard deviation value of 0.14 eV in 95% confidence level (Figures S1 and S2). The root mean square deviation (RMSD) value was also precited to be 0.143 eV, suggesting an almost symmetrical distribution around the average. A strong linear correlation (−0.86) was observed between the number of rings and the energy gap values, highlighting a systematic trend where the gap decreases with increasing electron delocalization. These findings demonstrate the robustness and internal consistency of the electronic structure data predicted by DFT.

The energy gap remains relatively stable as the number of rings increases. This feature makes it a tunable parameter via chemical functionalization or doping. Studies on tellurophene-based helicene structures have proven that they can be potential candidates for optoelectronic materials according to the HOMO-LUMO energy gap values, and it is anticipated that the expanded [n]-helicenes can be used in the field of optical and electronic materials in the future in terms of their controllable parameters [31].

The surface area-to-volume ratios decrease with increasing number of rings from 3 nm^−1^ to 2 nm^−1^. The decrease in A/V ratio may be considered as an analog of surface tension at the molecular scale, in other words, the molecules prefer a spherical-like structure in 3D, as seen in Figure 1. The HOMO-LUMO gap trend, decreasing moderately from 3.3 to 3.8 eV, is parallel to the increase in conjugation length and delocalization across the helical molecular skeleton. In contrast to classical helicene structures, which exhibit weaker electronic delocalization due to tight ortho-fusion and curvature-induced strain, expanded [n]-helicenes exhibit reduced strain and a more extended π-manifold which led to the distinct electronic profiles. This is supported by the linear anticorrelation between the calculated ΔE_HL_ values and n number. Their relatively narrow ΔE_HL_ range ensures stability while preserving their electronic responsiveness under functionalization and doping processes which may make them suitable candidates for tunable organic semiconductors.

To get more insight on the structural and electronic trends observed in expanded [n]-helicenes, we emphasize that the surface area-to-volume ratio (A/V), HOMO-LUMO gap (ΔE_HL_), and racemization barriers (ΔG_act_) are interrelated descriptors of both electronic delocalization and conformational complexity. The A/V ratio serves as an indicator of three-dimensional molecular structure and the surface compactness. The decreasing trend in A/V ratios with increasing n number indicates the natural progression of these systems toward a more compact, spherical-like conformations, akin to molecular folding or entropic compaction. This transition from planar to helical topologies, particularly pronounced beyond n = 10 (Figure 1e), indicates an increase in dimensionality in their topological structures and enhances weak noncovalent interactions like π–π and CH/π interactions between terminal rings, reminiscent of interfacial stacking in supramolecular assemblies. From a materials science perspective, the surface area-to-volume ratio serves as a potential important parameter for surface accessibility and curvature, which are critical parameters in host–guest chemistry, molecular recognition, and enantioselective catalysis. Expanded [n]-helicenes with higher surface area values and tunable curvature may offer potential candidates for optoelectronic devices, supramolecular sensing, and charge transport materials.

**Figure 4:**
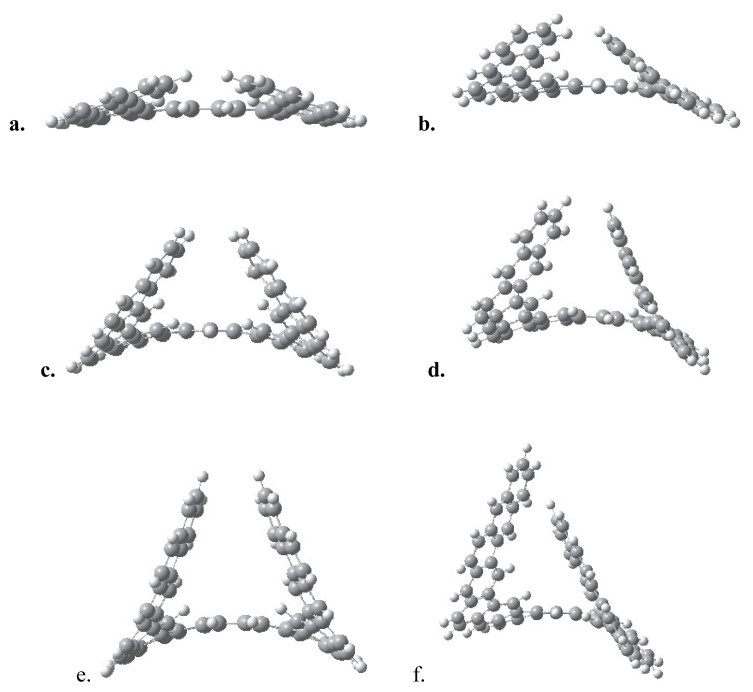
Transition state geometries of expanded [n]-helicenes for n equals a. 11, b. 12, c. 13, d. 14, e. 15, and f. 16.

The vertical positions of the rings at two opposite ends of the molecular framework are important in the racemization of the expanded [n]-helicenes. The transition state geometries of expanded helicenes with n = 11–16 are shown in Figures 4a–4f.

The benzene rings at the opposite ends are positioned with a vertical interval of 2.8 Å for n = 11 (Figure 4a), and topologically, a low barrier value is expected for the interconversion of R and P enantiomers. In fact, the racemization barrier was calculated as 18.6 kJ/mol by DFT method, which is a relatively low barrier value. However, for n > 12, as the rings at the opposite ends begin to stack vertically about a 3.5 Å distance interval, higher barrier values are expected for the conversion of the R enantiomer to the P enantiomer. The barrier value calculated by the DFT method is 33.8 kJ/mol for n = 12 member (Figure 4b). As the number of rings increases, the vertical gap at two opposite ends also gets wider, and more rings begin to stack on top of each other, leading to an increase in the activation barriers as expected. The distance between the closest terminal hydrogens in opposite arms of the molecule (d values in Table 3) also increases by n number.

**Figure 5:**
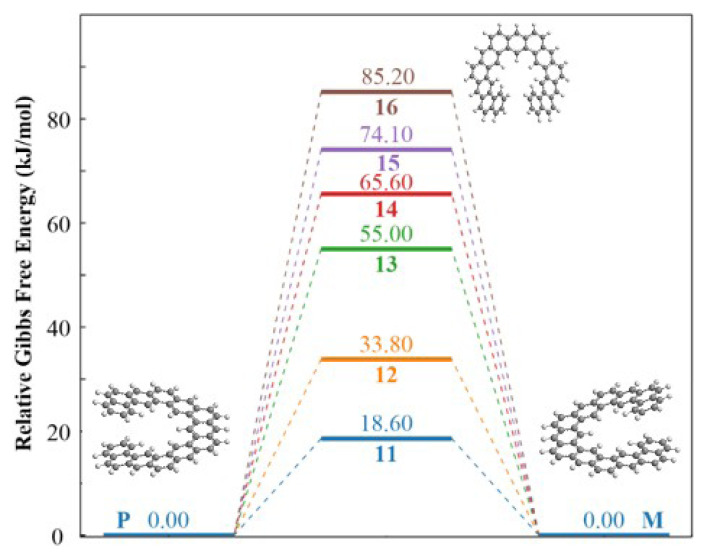
Reaction profiles of expanded [n]-helicenes for n equals 11 (blue), 12 (orange), 13 (green), 14 (red), 15 (violet), and 16 (brown). (The reactant and product species are positioned at the zero level, since the ordinate represents relative energy).

The activation barrier of the expanded [13]-helicene was predicted to be 55 kJ/mol in this study, which is the same with the reported value of 54.4 kJ/mol in the literature [29]. On the other hand, an activation barrier value for the methoxy-modified expanded [17]-helicene has been reported by the group that synthesized the molecule as 95.4 kJ/mol [28]. Although the barrier value is very high for [n]-helicenes with the same number of rings, the barrier values for expanded [n]-helicenes are predicted to be significantly lower, e.g., the energy barrier for [12]-helicene was reported as 272 kJ/mol in literature [32], while the barrier value is predicted to be 34 kJ/mol for the expanded [12]-helicene. Notably, an almost linear correlation (0.987) was found between the number of rings and the activation barrier, confirming the expected trend that the steric and the structural complexity enhances the configurational stability of these molecules (Figures S3 and S4). The reaction profiles of expanded [n]-helicenes with n = 11–16 are shown in Figure 5.

Since racemization barriers can be sensitive to the choice of functional, the racemization barrier values were also calculated by using different functionals to test the reliability of the computational procedure applied. In a previous study, both meta-GGA and hybrid functionals, along with their dispersion corrected counterparts, were shown to predict the racemization barriers of classical helicenes within chemical accuracy [26]. A variety of functionals from different types, i.e. hybrid, meta-GGA, and dispersion-corrected functionals were used for this purpose. The results for expanded [12]- and [13]-helicenes were summarized in Table 4.

All TS structures for expanded [12]-helicene successfully converged in the case of each functional. In contrast, expanded [13]-helicene showed convergence failure with M06-2X and B3LYP-D3. This likely stems from the large conjugated system, which may be more sensitive to self-interaction errors in M06-2X and the unbalanced dispersion correction in B3LYP-D3. A higher HF exchange, as in M06-2X, did not improve convergence and may in fact worsen geometry optimization. As can be inferred from Table 4, PBE and M06 functionals performed well without convergence issues. PBE gave barriers nearly identical to B3LYP, while M06 increased them by about 1 kcal/mol. B3LYP-D3, known to overestimate interaction energies, appears to similarly overestimate reaction barriers here (relative to both B3LYP and Minnesota functionals). Overall, B3LYP gives reliable barrier predictions, and meta-GGA functionals perform well once the convergence is achieved.

**Figure 6:**
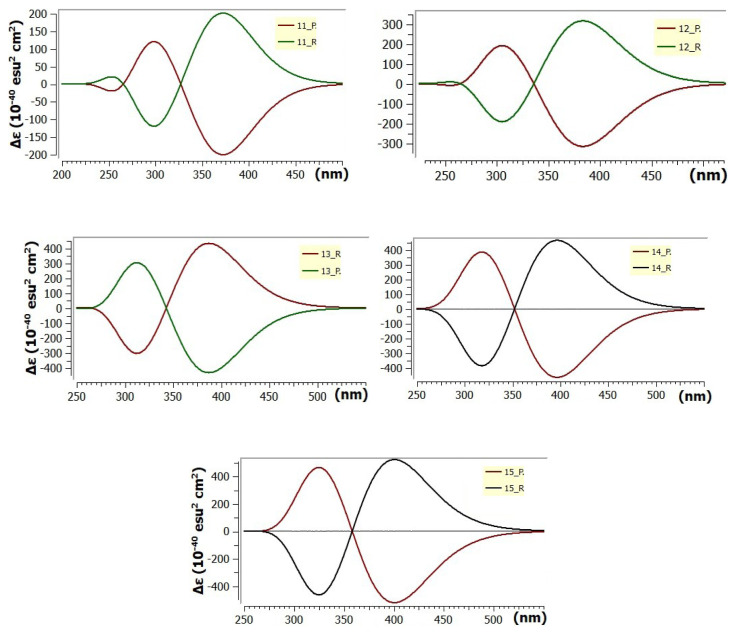
CD spectra of expanded [n]-helicenes (for n = 11–15).

The circular dichroism (CD) spectra of the R and P enantiomers forming a racemic mixture were calculated using the TD-DFT method in the same basis set. The graphs of the molar circular dichroism band intensities as a function of wavelength are shown in Figure 6.

Expanded helicenes are expected to exhibit enhanced chiroptical properties compared to classical helicenes, however, their synthesis remains highly challenging. Due to their low racemization barrier values, isolating their enantiomers via chiral liquid chromatography is particularly difficult. Considering that, the calculated CD spectra provide critical insights into the chiral optical activity of expanded [n]-helicenes with direct implications for experimental enantiomeric separation, Figure 6. Distinct Cotton effects are predicted for the R and P enantiomers across the UV-VIS region, with systematically red-shifted band positions as the n number increases. This red-shift corresponds to an enhanced π-conjugation and the extension of effective chromophoric pathways. For the expanded [11]- and [12]-helicenes, the peaks are calculated at 265 and 330 nm respectively. Further red-shifting is predicted in expanded [13]-, [14]-, and [15]-helicenes with the peak positions at 345, 355, and 360 nm, respectively. The magnitude of the corresponding rotatory strengths correlates directly with both the helical pitch and symmetry of the chiral π-system. Notably, the chirality in expanded [n]-helicene structures becomes observable beginning with the expanded [11]-helicene; however, due to a projective gap between terminal rings, its associated peak value is lower than those of the members with larger n number. Starting from the expanded [12]-helicene structure where the terminal rings coincide projectively, a significant increase in the peak value is observed. The lowest energy transitions, primarily of π–π* type, are optically active and contribute to the diagnostic bands appearing in 330–360 nm region. Importantly, these bands are well separated from the spectral region of many achiral organic chromophores, rendering them particularly advantageous for the experimental CD-based determination of enantiomeric excess.

### 3.2. Functionalized expanded helicenes

**Figure 7:**
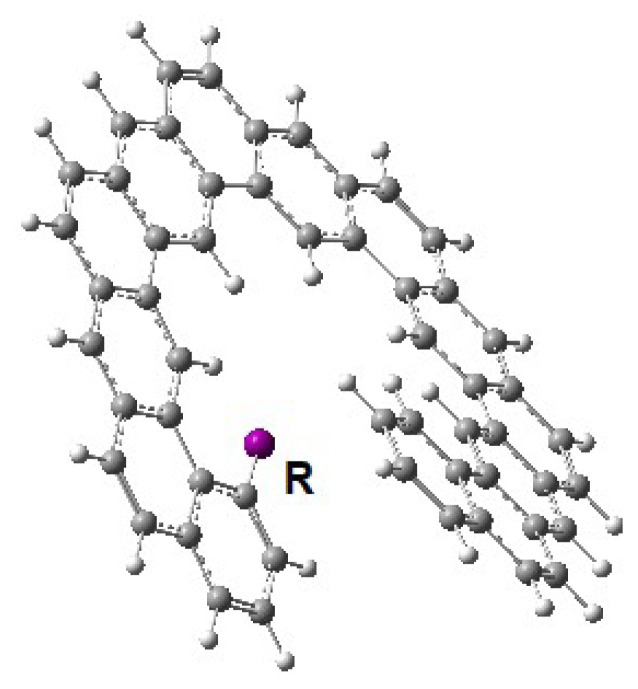
The geometry of functionalized expanded [13]-helicene.

In order to understand the effect of functionalization on the electronic properties and the activation barriers of expanded [n]-helicene structures, the expanded helicene molecules for n: 12–14 were functionalized with common R groups such as -Me, -OH, -OMe, -NH_2_, and -COOH, as shown in Figure 7.

**Figure 8:**
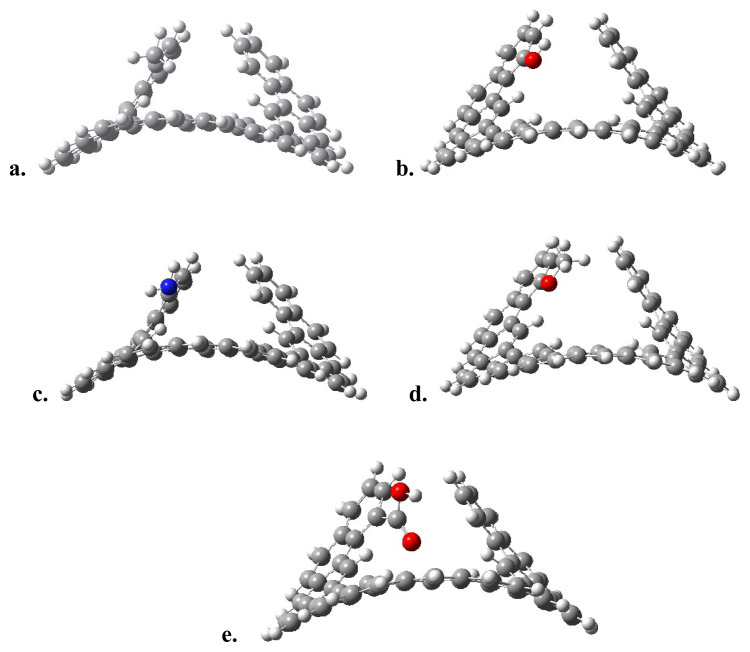
Transition state geometries of functionalized [13]-helicenes (a. Me, b. OH, c. NH_2_, d. OME, e. COOH).

The transition state geometries found for the racemization of these structures are shown in Figure 8 for the expanded [13]-helicene, as an example. The corresponding activation barriers are summarized in Table 5.

It is obvious that, as seen in Figure 8, the transition state geometry is preserved under the functionalization of the structure. This indicates that the racemization process also occurs in the presence of these functional groups. However, it is observed that functionalization does not show a significant effect on the activation barriers for the expanded [13]-helicene, with the change being at most 5 kJ/mol. For a further justification of this theoretical prediction, the effect of functionalization on the activation barriers for expanded [12]- and [14]-helicenes was also calculated. The largest difference is again in the order of 5 kJ/mol.

Based on the average RMSD value of B3LYP functional for reaction barriers, the changes about 5 kJ/mol -or lower values- have an insignificant effect on the activation barrier. Only for the expanded [12]-helicene, a decrease in the barrier of about 10 kJ/mol is predicted in the presence of the -COOH group. However, as the value of n increases, the change caused by the -COOH group becomes negligible. As a result, it is predicted that functionalization with these functional groups has no significant effect on controlling the racemization barrier of expanded [n]-helicenes. Likewise, its effects on the racemization barrier, the functionalization did not change the HOMO-LUMO energy gaps significantly.

### 3.3. Doped expanded helicenes

The effect of doping type modification on the HOMO-LUMO gap and the racemization barrier, boron-, nitrogen-, silicon-, and phosphorus-doped expanded helicene structures were studied.

**Figure 9:**
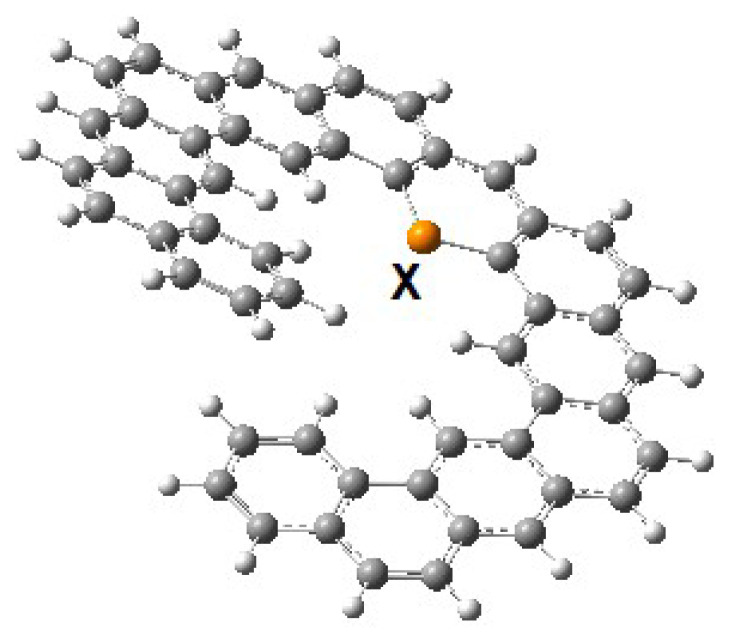
The doped expanded [13]-helicene structure (X: B, N, Si, P).

The doped expanded helicene structure is shown in Figure 9. The corresponding racemization barriers for these molecules were summarized in Table 6.

Compared to functionalization, the doping of the expanded helicene structure significantly changes the racemization barrier. It is obvious that the doping with boron, silicon, and phosphorus significantly lowers the barrier while nitrogen doping increases the barrier. On the other hand, for n > 15, it is observed that the effect of doping on the activation barrier becomes negligible.

The effect of doping process on the racemization barrier values may be attributed to both electronic redistribution and local steric perturbations introduced by dopant atoms. In particular, doping with an electron-deficient element like boron causes an electronic destabilization which facilitates the inversion process by lowering the barrier for breaking planarity at the transition state. The doping with boron atom consistently decreases barrier values across the series, with a reduction of around 10–15 kJ/mol relative to the pristine structures. The dopants like silicon and phosphorus, larger and more electropositive atoms than carbon, may induce the flexibility/relaxation in the backbone. Sterically, this reduces the enthalpic cost of achieving the twisted transition state structure. In contrast, the nitrogen atom, as an electron-rich dopant with a similar covalent radius to carbon, tends to increase the electron density in the π-system, stabilizing the helical structure of the molecule and thus increasing the barrier value. These effects are particularly pronounced for lower n values (n = 11–13) where local bonding environments are more sensitive to substitution. For higher n values (n exceeds 14), the increasing configurational rigidity renders the impact of local doping less significant.

The effect of doping on the HOMO-LUMO gap is also remarkable. All four dopands significantly lower the energy gap. Among them, silicon and phosphorus are the most effective dopands to decrease the gap value, while nitrogen dopand was found less effective in controlling the energy gap.

### 3.4. Möbius [n+1]-circulenes

Given their nonplanar, π-conjugated architectures and a comparable molecular formula, Möbius [n+1]-circulenes present an intriguing parallel to expanded [n]-helicene structures. While differing in their topology in terms of a twist in their structure along with their symmetry properties, both families of compounds offer unique platforms for exploring chiroptical behavior, electronic transitions, and aromaticity within a comparable structural framework (C_4n_H_2n_). Subtle distinctions and/or similarities in energy gaps (ΔE_HL_) and surface area-to-volume ratios (A/V) further enrich the comparative landscape, offering a deeper insight into the structure–property relationships relevant to chiral optoelectronic applications.

**Figure 10:**
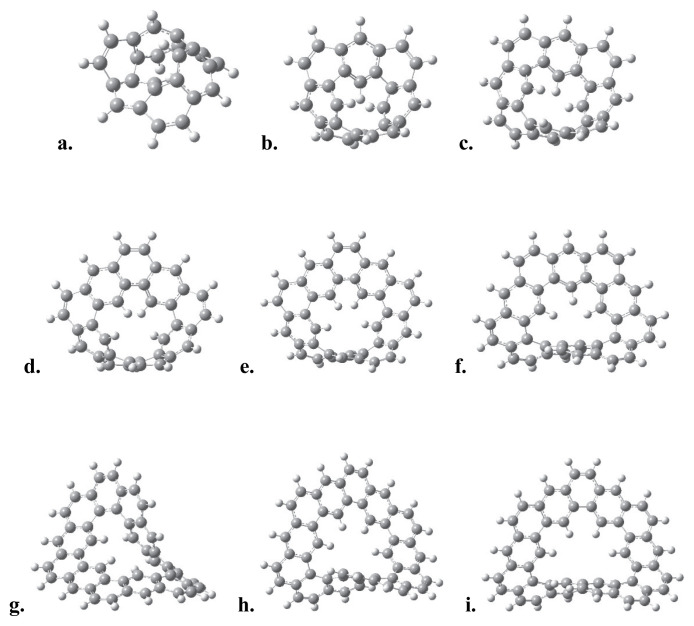
The optimized geometries of Möbius [n + 1]-circulenes for n+1 is a. 7, b. 8, c. 9, d. 10, e. 11, f. 12, g. 13, h. 14, i. 15.

For an [n]-circulene structure, having the same number of rings with the [n]-helicene and the expanded [n]-helicene, a Möbius circulene isomer with one missing ring is possible with the same molecular formula C_4n_H_2n_. Then, for the corresponding Möbius isomer structure, the Möbius [n+1]-circulene notation is valid, e.g., the Möbius isomer of the [12]-circulene, which is the D_6h_-symmetric planar Kekulene structure, with the same molecular formula is the C_2_-symmetric Möbius [13]-circulene (also known as Möbius Kekulene), Figure 10.

The smallest possible stable Möbius circulene structure was predicted to be Möbius 7-circulene; in other words, the lower limit for the number of rings (n) for the thermodynamically stable Möbius circulenes is 6. The calculated HOMO-LUMO gaps, molecular surface areas, and A/V ratios for the Möbius [n+1]-circulenes are given in Table 7. For the smallest member of this molecular family, Möbius [7]-circulene, the HOMO-LUMO gap is found to be 2.33 eV. For all the other molecules, this energy gap ranges from 3.1–3.3 eV, with the difference being optically insignificant.

The isomers of MK13, MK14, and MK15 with the same molecular formulas (C_48_H_24_, C_52_H_26,_ and C_56_H_28_ respectively) were also included in Table 7 to make a comparison: Kekulene and infinitene, [13]-circulene, and septulene. The surface area-to-volume ratios of the Möbius circulenes were found to be approximately 2.3 nm^−1^, and A/V ratios are found to be lower compared to [n]-circulenes that do not show a half or full twist topologically. It is noteworthy that both the HOMO-LUMO gap and A/V ratio of Möbius [13]-circulene and its Möbius isomer, infinitene, are found nearly identical. Their similar, slightly lower, energy gaps and surface area-to-volume ratio characteristics—particularly similar to larger expanded helicenes—may suggest potential integration in devices requiring matched or complementary electronic behavior but distinct chiroptical response.

## 4. Conclusion

Since the first report on their existence in 2018, expanded [n]-helicenes have remained a relatively underexplored class of PAH molecules. The only member synthesized in its pristine form is the expanded [13]-helicene molecule, while the larger homologues with n = 15 and 17 were only synthesized in methoxy functionalized form. In this work, the structural and electronic properties of expanded [n]-helicenes (for n = 6–16) were studied by using DFT method. The key characteristics such as topological configurations, HOMO-LUMO energy gaps, molecular surface area-to-volume ratios, and the racemization barriers were examined. The HOMO-LUMO energy interval of expanded helicenes were predicted to be in 3.3–3.7 eV interval and found slightly decreasing with n number. The molecular surface area to volume ratio of expanded helicenes were predicted to be around 2–3 nm^−1^. The racemization barriers were also predicted and found to be significantly lower compared to the classical helicene analogs with the same n number, suggesting enhanced configurational flexibility. The circular dichroism spectra for racemic pairs were predicted using time-dependent DFT, offering a priori data for the chemistry of expanded [n]-helicene molecules. The effect of functionalization on the racemization barriers was found insignificant, whereas the effect of heteroatom-doping was predicted to be important to control the barrier energies for smaller expanded helicenes (n < 15). For larger expanded helicenes, the effect of dopand atom on the racemization barriers was predicted to be insignificant. The structural and electronic properties of Möbius circulene isomers, which have the same molecular formulae as the [n]-helicene or expanded [n]-helicenes, were also investigated. The smallest stable Möbius circulene was predicted to be Möbius [7]-circulene. The HOMO-LUMO energy gaps of Möbius [n + 1]-circulenes (for n + 1 = 8–15) were all predicted to be around 3.2 eV. The surface area-to-volume (A/V) ratios of Möbius circulenes were found lower compared to [n]-circulene analogs. Notably, the Möbius [13]-circulene and infinitene molecules exhibited nearly identical electronic features. These insights may facilitate the design of novel chiral molecular systems with tunable electronic properties.

## Supplementary Information



## Figures and Tables

**Table 1:** The topological construction of expanded [n]-helicenes (n: the number of rings).

n is even	$\frac{\text{n}}{2}+1$ ortho, $\frac{\text{n}}{2}-1$ meta	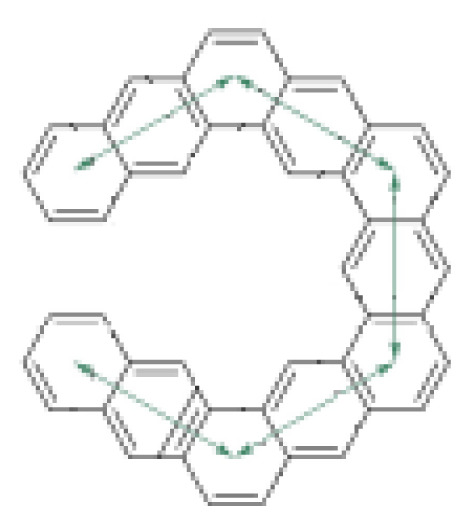
n is odd	$\frac{\text{n}+3}{2}$ ortho, $\frac{\text{n}-3}{2}$ meta	

**Table 2:** HOMO-LUMO gaps (ΔE_HL_ in eV) and surface area-to-volume ratios (A/V in nm^−1^) of expanded helicenes.

n	ΔE_HL_	A [cm^2^]	A/V
6	3.71	1.26 × 10^−14^	3.1
7	3.85	1.43 × 10^−14^	3.1
8	3.50	1.61 × 10^−14^	3.1
9	3.60	1.78 × 10^−14^	3.0
10	3.49	1.95 × 10^−14^	2.8
11	3.57	2.07 × 10^−14^	3.0
12	3.41	2.15 × 10^−14^	3.0
13	3.47	2.16 × 10^−14^	2.5
14	3.39	2.18 × 10^−14^	2.5
15	3.44	2.18 × 10^−14^	2.2
16	3.34	2.17 × 10^−14^	2.1

**Table 3:** The activation barriers (ΔG_act_) of expanded [n]-helicenes (d parameter was defined in the text).

n	ΔG_act_ [kJ/mol]	d [Å]
11	18.6	2.0
12	33.8	2.2
13	55.0	2.3
14	65.6	2.7
15	74.1	2.9
16	85.2	3.6

**Table 4:** Activation barriers of expanded [n]-helicenes calculated using different DFT functionals (N/A: convergence failure).

	ΔG_act_ [kJ/mol]	
Functional	n = 12	n = 13
B3LYP	33.8	55.0
PBE	34.7	55.6
M06	38.5	58.5
M06-2X	38.0	N/A
B3LYP-D3	40.1	N/A

**Table 5:** The racemization barriers (ΔG_act_) and HOMO-LUMO gaps (ΔE_HL_) of functionalized expanded helicenes (n: 12–14).

	ΔG_act_ [kJ/mol]			ΔE_HL_ [eV]		
R	12	13	14	12	13	14
H	33.8	55.0	65.6	3.41	3.47	3.39
OH	38.4	59.8	71.4	3.34	3.40	3.37
Me	31.8	51.5	64.0	3.41	3.47	3.39
OMe	38.1	56.7	66.6	3.39	3.44	3.38
NH_2_	33.3	54.7	66.8	3.40	3.44	3.38
COOH	24.2	51.9	68.6	3.38	3.39	3.32

**Table 6:** The racemization barriers (ΔG_act_) and HOMO-LUMO gaps (ΔE_HL_) of doped expanded helicenes (n: 11–15).

	ΔG_act_ [kJ/mol]					ΔE_HL_ [eV]				
dopant	11	12	13	14	15	11	12	13	14	15
C	18.6	33.8	55.0	65.6	74.1	3.57	3.41	3.47	3.39	3.44
B	8.9	23.5	45.9	57.8	69.1	3.23	3.20	3.19	3.15	3.16
N	31.6	48.1	70.2	79.8	86.5	3.43	3.32	3.37	3.24	3.28
Si	2.8	6.8	29.2	42.6	54.8	3.13	3.04	3.07	3.08	3.07
P	6.2	13.1	38.9	50.8	62.3	3.16	3.06	3.08	3.05	3.06

**Table 7:** HOMO-LUMO gaps (ΔE_HL_ in eV) and surface area-to-volume (A/V) ratios (nm^−1^) of Möbius [n+1]-circulenes.

n+1	ΔE_HL_	A [cm^2^]	A/V
7	2.33	8 × 10^−15^	2.2

8	3.15	9 × 10^−15^	2.4

9	3.06	11 × 10^−15^	2.4

10	3.10	12 × 10^−15^	2.3

11	3.07	14 × 10^−15^	2.5

12	3.24	15 × 10^−15^	2.2

13	3.27	16 × 10^−15^	2.3
Kekulene	3.53	21 × 10^−15^	2.9
Infinitene	3.01	16 × 10^−15^	2.3

14	3.22	18 × 10^−15^	2.3
[13]-circulene	2.91	22 × 10^−15^	3.5

15	3.30	18 × 10^−15^	2.1
Septulene	3.43	23 × 10^−15^	2.9
