# Supplementary material for: The theoretical investigation of the stability and the racemization of pristine, functionalized, and doped expanded helicenes
Source: Turk J Chem. 2025 Jul 22;49(5):549–63. doi: 10.55730/1300-0527.3752 (PMC12604923; doi:10.55730/1300-0527.3752)
Supplement: Supplementary file 1 [file tjc-49-05-549-_supporting_Information.docx]

Supporting Information

The theoretical investigation of the stability and the racemization of pristine, functionalized and doped expanded helicenes

Bedirhan Öztürk, Berkay Sütay *

Istanbul Teknik Üniversitesi, Kimya Bölümü, Sarıyer, Istanbul. Corresponding author: [sutay@itu.edu.tr](mailto:sutay@itu.edu.tr)

Cartesian coordinates for the optimized structures

Expanded [6]-helicene:

X Y Z

**-----------------------------------------------------------------**

| **C** | **5.37959100** | **-2.67019200** | **0.00000000** |
| --- | --- | --- | --- |
| **C** | **4.03924300** | **-3.09424400** | **0.00000000** |
| **C** | **3.01152300** | **-2.16661200** | **0.00000000** |
| **C** | **5.66456500** | **-1.31782100** | **0.00000000** |
| **C** | **4.63220500** | **-0.35342200** | **0.00000000** |
| **C** | **3.26961300** | **-0.77708800** | **0.00000000** |
| **C** | **4.94453500** | **1.05021800** | **0.00000000** |
| **C** | **3.96509900** | **1.99097000** | **0.00000000** |
| **C** | **2.57794600** | **1.61753100** | **0.00000000** |
| **C** | **2.21337500** | **0.23269600** | **0.00000000** |
| **C** | **0.84713000** | **-0.09031400** | **0.00000000** |
| **C** | **-0.16649200** | **0.87182700** | **0.00000000** |
| **C** | **0.21317100** | **2.25391200** | **0.00000000** |
| **C** | **1.56754500** | **2.58783000** | **0.00000000** |
| **C** | **-1.59486700** | **0.52631400** | **0.00000000** |
| **C** | **-2.56803300** | **1.59236200** | **0.00000000** |
| **C** | **-2.12013600** | **2.96419100** | **0.00000000** |
| **C** | **-0.80285700** | **3.27815700** | **0.00000000** |
| **C** | **-2.06262900** | **-0.78418500** | **0.00000000** |
| **C** | **-3.43655200** | **-1.09962300** | **0.00000000** |
| **C** | **-4.39934900** | **-0.03348700** | **0.00000000** |
| **C** | **-3.92699800** | **1.29147400** | **0.00000000** |
| **C** | **-3.90514100** | **-2.44540300** | **0.00000000** |
| **C** | **-5.25054000** | **-2.72292700** | **0.00000000** |
| **C** | **-6.20230200** | **-1.66710000** | **0.00000000** |
| **C** | **-5.78681800** | **-0.35792800** | **0.00000000** |
| **H** | **1.98883000** | **-2.52813800** | **0.00000000** |
| **H** | **6.69649700** | **-0.97439000** | **0.00000000** |
| **H** | **5.99170400** | **1.34308800** | **0.00000000** |
| **H** | **4.21305800** | **3.04960400** | **0.00000000** |
| **H** | **0.56592600** | **-1.13545500** | **0.00000000** |
| **H** | **1.85017300** | **3.63872700** | **0.00000000** |
| **H** | **-2.87519400** | **3.74654400** | **0.00000000** |
| **H** | **-0.48115000** | **4.31675000** | **0.00000000** |
| **H** | **-1.36421600** | **-1.61535100** | **0.00000000** |
| **H** | **-4.64696000** | **2.10756000** | **0.00000000** |
| **H** | **-7.26304800** | **-1.90280400** | **0.00000000** |
| **H** | **-6.51190900** | **0.45258900** | **0.00000000** |
| **H** | **6.18390500** | **-3.40057800** | **0.00000000** |
| **H** | **3.80555800** | **-4.15543800** | **0.00000000** |
| **H** | **-3.17549900** | **-3.25194100** | **0.00000000** |
| **H** | **-5.59463500** | **-3.75357900** | **0.00000000** |

Expanded [7]-helicene:

X Y Z

**-----------------------------------------------------------------**

| **C** | **5.30648800** | **3.39519500** | **0.00026200** |
| --- | --- | --- | --- |
| **C** | **3.90452300** | **3.49648800** | **0.00010700** |
| **C** | **3.12036500** | **2.35531700** | **-0.00003400** |
| **C** | **5.89760600** | **2.14578400** | **0.00022600** |
| **C** | **5.11741800** | **0.96816500** | **0.00008100** |
| **C** | **3.69386500** | **1.06389600** | **0.00000500** |
| **C** | **5.74763900** | **-0.32475600** | **-0.00005400** |

| **C** | **5.01392700** | **-1.46723300** | **-0.00025000** |
| --- | --- | --- | --- |
| **C** | **3.57762900** | **-1.42668900** | **-0.00023300** |
| **C** | **2.90049300** | **-0.16402200** | **-0.00005500** |
| **C** | **1.49766100** | **-0.16695700** | **0.00009900** |
| **C** | **0.73364100** | **-1.33891100** | **-0.00003200** |
| **C** | **1.42546900** | **-2.59533000** | **-0.00018300** |
| **C** | **2.82134300** | **-2.60457300** | **-0.00028400** |
| **C** | **-0.73364100** | **-1.33891100** | **0.00003200** |
| **C** | **-1.42546900** | **-2.59533000** | **0.00018300** |
| **C** | **-0.67765300** | **-3.82703200** | **0.00011600** |
| **C** | **0.67765300** | **-3.82703200** | **-0.00011700** |
| **C** | **-1.49766100** | **-0.16695700** | **-0.00009900** |
| **C** | **-2.90049300** | **-0.16402200** | **0.00005500** |
| **C** | **-3.57762900** | **-1.42668900** | **0.00023300** |
| **C** | **-2.82134300** | **-2.60457300** | **0.00028400** |
| **C** | **-3.69386500** | **1.06389600** | **-0.00000500** |
| **C** | **-5.11741800** | **0.96816500** | **-0.00008100** |
| **C** | **-5.74763900** | **-0.32475600** | **0.00005400** |
| **C** | **-5.01392700** | **-1.46723300** | **0.00025000** |
| **C** | **-3.12036500** | **2.35531700** | **0.00003400** |
| **C** | **-3.90452300** | **3.49648800** | **-0.00010700** |
| **C** | **-5.30648800** | **3.39519500** | **-0.00026200** |
| **C** | **-5.89760600** | **2.14578400** | **-0.00022600** |
| **H** | **2.04167100** | **2.46962000** | **-0.00026100** |
| **H** | **6.98109600** | **2.05139300** | **0.00028400** |
| **H** | **6.83421100** | **-0.36612400** | **-0.00002300** |
| **H** | **5.50123300** | **-2.43921600** | **-0.00039700** |
| **H** | **0.98296100** | **0.78498400** | **0.00048000** |
| **H** | **3.34043100** | **-3.56103500** | **-0.00035900** |
| **H** | **-1.23190900** | **-4.76243100** | **0.00021500** |
| **H** | **1.23190900** | **-4.76243100** | **-0.00021500** |
| **H** | **-0.98296100** | **0.78498400** | **-0.00048000** |
| **H** | **-3.34043100** | **-3.56103500** | **0.00035900** |
| **H** | **-6.83421100** | **-0.36612400** | **0.00002300** |
| **H** | **-5.50123300** | **-2.43921600** | **0.00039700** |
| **H** | **-2.04167100** | **2.46962000** | **0.00026100** |
| **H** | **-6.98109600** | **2.05139300** | **-0.00028400** |
| **H** | **5.91935700** | **4.29232400** | **0.00038600** |
| **H** | **3.43080500** | **4.47443700** | **0.00007100** |
| **H** | **-3.43080500** | **4.47443700** | **-0.00007100** |
| **H** | **-5.91935700** | **4.29232400** | **-0.00038600** |

Expanded [8]-helicene:

X Y Z

**-----------------------------------------------------------------**

| **C** | **-4.88605000** | **4.11324800** | **-0.00010600** |
| --- | --- | --- | --- |
| **C** | **-3.81920000** | **3.24812900** | **-0.00021500** |
| **C** | **-6.21701300** | **3.61348900** | **0.00015700** |
| **C** | **-6.45028400** | **2.26009300** | **0.00029800** |
| **C** | **-5.36741900** | **1.33363900** | **0.00018300** |
| **C** | **-4.02196100** | **1.83746000** | **-0.00007500** |
| **C** | **-2.94729200** | **0.92568300** | **-0.00017200** |
| **C** | **-5.55771700** | **-0.05974000** | **0.00028200** |
| **C** | **-4.48937700** | **-0.95237600** | **0.00018300** |
| **C** | **-3.13475200** | **-0.45349100** | **-0.00000100** |
| **C** | **-4.72279600** | **-2.37593400** | **0.00020400** |
| **C** | **-3.69699700** | **-3.26057600** | **0.00004000** |
| **C** | **-2.32474900** | **-2.81745600** | **-0.00007500** |
| **C** | **-2.02640200** | **-1.41735300** | **-0.00002300** |
| **C** | **-0.68179300** | **-1.02958600** | **0.00002100** |
| **C** | **0.38168700** | **-1.94306700** | **-0.00009400** |
| **C** | **0.06789500** | **-3.33998800** | **-0.00021100** |
| **C** | **-1.27338600** | **-3.73693200** | **-0.00017500** |
| **C** | **1.78901600** | **-1.53328500** | **-0.00007900** |

| **C** | **2.80439000** | **-2.54676500** | **-0.00006700** |
| --- | --- | --- | --- |
| **C** | **2.43006500** | **-3.93729100** | **-0.00023000** |
| **C** | **1.12767800** | **-4.31480800** | **-0.00031800** |
| **C** | **2.19562000** | **-0.19407800** | **-0.00007400** |
| **C** | **3.54138900** | **0.20005000** | **0.00000100** |
| **C** | **4.54452100** | **-0.82365100** | **0.00018600** |
| **C** | **4.14770800** | **-2.16549300** | **0.00010800** |
| **C** | **3.96060100** | **1.60083200** | **-0.00008600** |
| **C** | **5.35420800** | **1.90628600** | **0.00024700** |
| **C** | **6.32047400** | **0.84057400** | **0.00055200** |
| **C** | **5.93512500** | **-0.46120900** | **0.00047800** |
| **C** | **3.04954700** | **2.68076800** | **-0.00051100** |
| **C** | **3.48399600** | **3.99555600** | **-0.00052400** |
| **C** | **4.85839300** | **4.28961200** | **-0.00012400** |
| **C** | **5.77473800** | **3.25470500** | **0.00023600** |
| **H** | **-7.05037000** | **4.31079600** | **0.00024400** |
| **H** | **-7.46688700** | **1.87384400** | **0.00049700** |
| **H** | **-1.94484300** | **1.34245400** | **-0.00044900** |
| **H** | **-6.57238500** | **-0.45313500** | **0.00041400** |
| **H** | **-5.75299400** | **-2.72355600** | **0.00033000** |
| **H** | **-3.88867000** | **-4.33081200** | **0.00001900** |
| **H** | **-0.45266200** | **0.02818600** | **0.00025500** |
| **H** | **-1.50549500** | **-4.80011900** | **-0.00019300** |
| **H** | **3.22287300** | **-4.68130900** | **-0.00030500** |
| **H** | **0.85573800** | **-5.36750600** | **-0.00047400** |
| **H** | **1.43551100** | **0.57621200** | **-0.00008500** |
| **H** | **4.91323200** | **-2.93892400** | **0.00019300** |
| **H** | **7.37531500** | **1.10443800** | **0.00082700** |
| **H** | **6.67440900** | **-1.25847000** | **0.00066500** |
| **H** | **1.98183600** | **2.48936800** | **-0.00091800** |
| **H** | **6.84150200** | **3.46641400** | **0.00050400** |
| **H** | **2.75612900** | **4.80237600** | **-0.00086400** |
| **H** | **5.19656300** | **5.32209400** | **-0.00012200** |
| **H** | **-4.71762000** | **5.18669300** | **-0.00021400** |
| **H** | **-2.80034900** | **3.62869300** | **-0.00041400** |

Expanded [9]-helicene:

X Y Z

**-----------------------------------------------------------------**

| **C** | **-4.28345800** | **-4.75568400** | **0.00129100** |
| --- | --- | --- | --- |
| **C** | **-3.00292700** | **-4.17624700** | **0.00116600** |
| **C** | **-5.39872600** | **-3.93916700** | **0.00092800** |
| **C** | **-5.27435300** | **-2.53209600** | **0.00045700** |
| **C** | **-3.97745000** | **-1.93744100** | **0.00040100** |
| **C** | **-2.85778100** | **-2.79924700** | **0.00071800** |
| **C** | **-6.44498000** | **-1.69620000** | **-0.00003500** |
| **C** | **-6.34494000** | **-0.34221000** | **-0.00054200** |
| **C** | **-5.06312300** | **0.30723300** | **-0.00050900** |
| **C** | **-3.86544600** | **-0.47966800** | **0.00000800** |
| **C** | **-2.63410800** | **0.19187100** | **0.00016500** |
| **C** | **-4.96048500** | **1.70289700** | **-0.00087300** |
| **C** | **-3.72912600** | **2.36094800** | **-0.00075000** |
| **C** | **-2.52140200** | **1.58669800** | **-0.00025700** |
| **C** | **-3.65977200** | **3.79957600** | **-0.00099600** |
| **C** | **-2.46805100** | **4.44595700** | **-0.00070600** |
| **C** | **-1.22454000** | **3.71949300** | **-0.00027500** |
| **C** | **-1.23285500** | **2.28690900** | **-0.00016100** |
| **C** | **0.00000000** | **1.62150800** | **0.00000000** |
| **C** | **1.23285500** | **2.28690900** | **0.00016100** |
| **C** | **1.22454000** | **3.71949300** | **0.00027500** |
| **C** | **0.00000000** | **4.39324000** | **0.00000000** |
| **C** | **2.52140200** | **1.58669800** | **0.00025700** |
| **C** | **3.72912600** | **2.36094800** | **0.00075000** |
| **C** | **3.65977200** | **3.79957600** | **0.00099600** |

| **C** | **2.46805100** | **4.44595700** | **0.00070600** |
| --- | --- | --- | --- |
| **C** | **2.63410800** | **0.19187100** | **-0.00016500** |
| **C** | **3.86544600** | **-0.47966800** | **-0.00000800** |
| **C** | **5.06312300** | **0.30723300** | **0.00050900** |
| **C** | **4.96048500** | **1.70289700** | **0.00087300** |
| **C** | **3.97745000** | **-1.93744100** | **-0.00040100** |
| **C** | **5.27435300** | **-2.53209600** | **-0.00045700** |
| **C** | **6.44498000** | **-1.69620000** | **0.00003500** |
| **C** | **6.34494000** | **-0.34221000** | **0.00054200** |
| **C** | **2.85778100** | **-2.79924700** | **-0.00071800** |
| **C** | **3.00292700** | **-4.17624700** | **-0.00116600** |
| **C** | **4.28345800** | **-4.75568400** | **-0.00129100** |
| **C** | **5.39872600** | **-3.93916700** | **-0.00092800** |
| **H** | **-6.39613200** | **-4.37275900** | **0.00097100** |
| **H** | **-1.85516800** | **-2.38513300** | **0.00053500** |
| **H** | **-7.41971600** | **-2.17807300** | **-0.00001700** |
| **H** | **-7.23676500** | **0.27974900** | **-0.00095500** |
| **H** | **-1.72767700** | **-0.39928200** | **0.00076600** |
| **H** | **-5.87294400** | **2.29589900** | **-0.00121900** |
| **H** | **-4.59298400** | **4.35746500** | **-0.00138000** |
| **H** | **-2.42674800** | **5.53243200** | **-0.00083000** |
| **H** | **0.00000000** | **0.53929700** | **0.00000000** |
| **H** | **0.00000000** | **5.48146900** | **0.00000000** |
| **H** | **4.59298400** | **4.35746500** | **0.00138000** |
| **H** | **2.42674800** | **5.53243200** | **0.00083000** |
| **H** | **1.72767700** | **-0.39928200** | **-0.00076600** |
| **H** | **5.87294400** | **2.29589900** | **0.00121900** |
| **H** | **7.41971600** | **-2.17807300** | **0.00001700** |
| **H** | **7.23676500** | **0.27974900** | **0.00095500** |
| **H** | **1.85516800** | **-2.38513300** | **-0.00053500** |
| **H** | **6.39613200** | **-4.37275900** | **-0.00097100** |
| **H** | **-4.39447500** | **-5.83646800** | **0.00166800** |
| **H** | **-2.12035700** | **-4.81019100** | **0.00139500** |
| **H** | **4.39447500** | **-5.83646800** | **-0.00166800** |
| **H** | **2.12035700** | **-4.81019100** | **-0.00139500** |

Expanded [10]-helicene:

X Y Z

**-----------------------------------------------------------------**

| **H** | **-2.91259000** | **6.38043700** | **-0.22559300** |
| --- | --- | --- | --- |
| **C** | **-3.12486800** | **5.31575200** | **-0.18356300** |
| **H** | **-1.04435600** | **4.72766100** | **-0.26775300** |
| **C** | **-2.07334400** | **4.38336700** | **-0.20549800** |
| **C** | **-4.42987900** | **4.86622500** | **-0.11058900** |
| **C** | **-4.72562100** | **3.48597300** | **-0.05705600** |
| **C** | **-3.66270200** | **2.53448900** | **-0.07480000** |
| **C** | **-2.34017400** | **3.02582300** | **-0.15152700** |
| **C** | **-6.08947800** | **3.03407700** | **0.01138100** |
| **C** | **-6.39294600** | **1.71146500** | **0.05618000** |
| **C** | **-5.36041900** | **0.71224200** | **0.04214900** |
| **C** | **-3.98498100** | **1.10963300** | **-0.01770000** |
| **C** | **-3.00665500** | **0.10456500** | **-0.01961100** |
| **C** | **-5.67366900** | **-0.65114000** | **0.08432600** |
| **C** | **-4.69160500** | **-1.64338100** | **0.07457000** |
| **C** | **-3.30990900** | **-1.26058000** | **0.02760700** |
| **C** | **-5.04949500** | **-3.03829300** | **0.10703300** |
| **C** | **-4.10208100** | **-4.00779900** | **0.09099000** |
| **C** | **-2.69983300** | **-3.68131600** | **0.05072900** |
| **C** | **-2.28508700** | **-2.30978700** | **0.02699800** |
| **C** | **-0.91154200** | **-2.03763400** | **0.00536100** |
| **C** | **0.07043200** | **-3.03759300** | **-0.00241200** |
| **C** | **-0.36034100** | **-4.40424300** | **0.00493300** |
| **C** | **-1.72939500** | **-4.68639300** | **0.03536400** |
| **C** | **1.50676700** | **-2.74959200** | **-0.01989900** |

| **C** | **2.43074600** | **-3.84310000** | **-0.05238300** |
| --- | --- | --- | --- |
| **C** | **1.94229800** | **-5.19692000** | **-0.05284600** |
| **C** | **0.61280200** | **-5.46424700** | **-0.02064800** |
| **C** | **2.02805600** | **-1.44779600** | **-0.00307900** |
| **C** | **3.39925300** | **-1.17014400** | **-0.02259600** |
| **C** | **4.31055600** | **-2.27350100** | **-0.06980900** |
| **C** | **3.80350900** | **-3.57450100** | **-0.08051200** |
| **C** | **3.93757300** | **0.19645200** | **0.00359500** |
| **C** | **5.36888200** | **0.38035600** | **-0.03702800** |
| **C** | **6.23286200** | **-0.77353900** | **-0.09340600** |
| **C** | **5.73180900** | **-2.03188800** | **-0.10532000** |
| **C** | **3.13568700** | **1.33197700** | **0.07005700** |
| **C** | **3.66811100** | **2.63680300** | **0.09485500** |
| **C** | **5.09320300** | **2.81217400** | **0.04740200** |
| **C** | **5.90440900** | **1.66509800** | **-0.01701600** |
| **C** | **2.83861600** | **3.79332500** | **0.16744300** |
| **C** | **3.38567900** | **5.05308900** | **0.18916900** |
| **C** | **4.79589200** | **5.22526600** | **0.13927500** |
| **C** | **5.62634600** | **4.13346300** | **0.07067200** |
| **H** | **-5.25430800** | **5.57536000** | **-0.09502600** |
| **H** | **-1.50547900** | **2.33328700** | **-0.17291600** |
| **H** | **-6.87816200** | **3.78249300** | **0.02358900** |
| **H** | **-7.42776300** | **1.38131100** | **0.10440700** |
| **H** | **-1.96681900** | **0.40123700** | **-0.06245100** |
| **H** | **-6.71988200** | **-0.94791400** | **0.12415700** |
| **H** | **-6.10532300** | **-3.29556300** | **0.14094400** |
| **H** | **-4.38320300** | **-5.05788800** | **0.11120000** |
| **H** | **-0.59218100** | **-1.00369100** | **-0.00637500** |
| **H** | **-2.05045900** | **-5.72610600** | **0.04717900** |
| **H** | **2.66965200** | **-6.00471100** | **-0.07596800** |
| **H** | **0.25370200** | **-6.49047500** | **-0.01681500** |
| **H** | **1.33623100** | **-0.61608200** | **0.02855700** |
| **H** | **4.50048900** | **-4.40969200** | **-0.11057000** |
| **H** | **7.30665800** | **-0.60546500** | **-0.12275800** |
| **H** | **6.39581300** | **-2.89197300** | **-0.14402800** |
| **H** | **2.05480900** | **1.23777900** | **0.10885000** |
| **H** | **6.98550800** | **1.78539800** | **-0.04952600** |
| **H** | **5.21355900** | **6.22822400** | **0.15685600** |
| **H** | **6.70571000** | **4.26097300** | **0.03386400** |
| **H** | **1.75975500** | **3.65940800** | **0.20789800** |
| **H** | **2.74185000** | **5.92661100** | **0.24553100** |

Expanded [11]-helicene:

X Y Z

**-----------------------------------------------------------------**

| **H** | **1.99535200** | **-6.32336300** | **-1.30586100** |
| --- | --- | --- | --- |
| **C** | **2.38455600** | **-5.33529000** | **-1.07624800** |
| **H** | **0.47261700** | **-4.34473900** | **-1.30263200** |
| **C** | **1.52744700** | **-4.22085500** | **-1.07557900** |
| **C** | **3.72514200** | **-5.16242600** | **-0.78779700** |
| **C** | **4.25029500** | **-3.88469700** | **-0.49254000** |
| **C** | **3.38548900** | **-2.74980400** | **-0.48354200** |
| **C** | **2.02072200** | **-2.95992400** | **-0.78304800** |
| **C** | **5.65097300** | **-3.71984600** | **-0.21026900** |
| **C** | **6.17755200** | **-2.49689300** | **0.05468600** |
| **C** | **5.35124200** | **-1.32149000** | **0.07302100** |
| **C** | **3.94520100** | **-1.43371200** | **-0.18002600** |
| **C** | **3.16997300** | **-0.26531800** | **-0.13191900** |
| **C** | **5.89895500** | **-0.05844400** | **0.32383300** |
| **C** | **5.12203400** | **1.10110000** | **0.34445000** |
| **C** | **3.70705600** | **1.00013500** | **0.12990800** |
| **C** | **5.72641000** | **2.39089800** | **0.55798200** |
| **C** | **4.98448600** | **3.52525000** | **0.54216700** |
| **C** | **3.55825500** | **3.48156300** | **0.34699400** |

| **C** | **2.89707100** | **2.22211100** | **0.17335400** |
| --- | --- | --- | --- |
| **C** | **1.50123200** | **2.22257400** | **0.05140200** |
| **C** | **0.73070700** | **3.39349600** | **0.05566200** |
| **C** | **1.41403900** | **4.64867200** | **0.16099600** |
| **C** | **2.80235600** | **4.65631800** | **0.32236300** |
| **C** | **-0.73069700** | **3.39349900** | **-0.05564700** |
| **C** | **-1.41402600** | **4.64867800** | **-0.16096300** |
| **C** | **-0.67230400** | **5.88016800** | **-0.08787300** |
| **C** | **0.67231900** | **5.88016500** | **0.08792500** |
| **C** | **-1.50122500** | **2.22257800** | **-0.05140300** |
| **C** | **-2.89706300** | **2.22212100** | **-0.17335600** |
| **C** | **-3.55824400** | **3.48157600** | **-0.34698800** |
| **C** | **-2.80234300** | **4.65632800** | **-0.32233800** |
| **C** | **-3.70705100** | **1.00014700** | **-0.12992000** |
| **C** | **-5.12202400** | **1.10111300** | **-0.34449400** |
| **C** | **-5.72639600** | **2.39091200** | **-0.55802800** |
| **C** | **-4.98447200** | **3.52526500** | **-0.54218200** |
| **C** | **-3.16997500** | **-0.26530500** | **0.13193000** |
| **C** | **-3.94520500** | **-1.43369900** | **0.18001900** |
| **C** | **-5.35123700** | **-1.32147900** | **-0.07307700** |
| **C** | **-5.89894500** | **-0.05843300** | **-0.32390200** |
| **C** | **-3.38550200** | **-2.74978800** | **0.48356300** |
| **C** | **-4.25030400** | **-3.88468500** | **0.49251900** |
| **C** | **-5.65097100** | **-3.71983800** | **0.21019000** |
| **C** | **-6.17754400** | **-2.49688400** | **-0.05477800** |
| **H** | **4.40116900** | **-6.01440700** | **-0.78849800** |
| **H** | **1.33280700** | **-2.12113000** | **-0.79821700** |
| **H** | **6.28443700** | **-4.60353500** | **-0.22173200** |
| **H** | **7.23951600** | **-2.38230600** | **0.25784300** |
| **H** | **2.10731800** | **-0.34558000** | **-0.32146200** |
| **H** | **6.97002700** | **0.02367900** | **0.49783500** |
| **H** | **6.80125900** | **2.43322400** | **0.71609900** |
| **H** | **5.45240700** | **4.49616900** | **0.68522400** |
| **H** | **0.99306200** | **1.27295300** | **-0.05863300** |
| **H** | **3.31316000** | **5.61145700** | **0.42720300** |
| **H** | **-1.22227800** | **6.81517100** | **-0.16115300** |
| **H** | **1.22229500** | **6.81516600** | **0.16122000** |
| **H** | **-0.99305700** | **1.27295400** | **0.05861300** |
| **H** | **-3.31314300** | **5.61147100** | **-0.42717100** |
| **H** | **-6.80124100** | **2.43324000** | **-0.71616700** |
| **H** | **-5.45239100** | **4.49618500** | **-0.68523500** |
| **H** | **-2.10732600** | **-0.34556400** | **0.32150600** |
| **H** | **-6.97001200** | **0.02369000** | **-0.49793400** |
| **H** | **-6.28443200** | **-4.60352900** | **0.22162100** |
| **H** | **-7.23950100** | **-2.38230100** | **-0.25797400** |
| **C** | **-3.72516000** | **-5.16241200** | **0.78779700** |
| **C** | **-2.38458600** | **-5.33527100** | **1.07631200** |
| **C** | **-1.52748300** | **-4.22083000** | **1.07569200** |
| **C** | **-2.02075000** | **-2.95990100** | **0.78314000** |
| **H** | **-4.40118200** | **-6.01439600** | **0.78846400** |
| **H** | **-1.99538800** | **-6.32334200** | **1.30594000** |
| **H** | **-0.47266500** | **-4.34471000** | **1.30280200** |
| **H** | **-1.33284200** | **-2.12110200** | **0.79835500** |

Bor Doped Expanded [11]-helicene:

X Y Z

**-----------------------------------------------------------------**

| **H** | **2.83719700** | **-6.29876300** | **-0.93840800** |
| --- | --- | --- | --- |
| **C** | **3.12243400** | **-5.26368700** | **-0.77177200** |
| **H** | **1.10436800** | **-4.50072100** | **-0.95975800** |
| **C** | **2.14653300** | **-4.25148300** | **-0.78095400** |
| **C** | **4.44652500** | **-4.93064900** | **-0.55712700** |
| **C** | **4.83814000** | **-3.58962200** | **-0.34644900** |
| **C** | **3.85186300** | **-2.55891000** | **-0.34348700** |

| **C** | **2.50741900** | **-2.93140600** | **-0.56773200** |
| --- | --- | --- | --- |
| **C** | **6.22263200** | **-3.25528000** | **-0.14766000** |
| **C** | **6.61842100** | **-1.96844600** | **0.03034400** |
| **C** | **5.66738200** | **-0.89161500** | **0.04175700** |
| **C** | **4.27077300** | **-1.17568500** | **-0.12712900** |
| **C** | **3.37205000** | **-0.10056700** | **-0.08286200** |
| **C** | **6.08512500** | **0.43559700** | **0.21092700** |
| **C** | **5.18412600** | **1.50080100** | **0.22864300** |
| **C** | **3.78636400** | **1.22410900** | **0.09211100** |
| **C** | **5.61612000** | **2.86834300** | **0.37061500** |
| **C** | **4.73508600** | **3.90157500** | **0.36732100** |
| **C** | **3.31027300** | **3.69703900** | **0.24327000** |
| **C** | **2.84842200** | **2.32112500** | **0.12439800** |
| **C** | **0.33904100** | **3.41402300** | **0.05423200** |
| **C** | **1.02697000** | **4.69457100** | **0.13740400** |
| **C** | **2.42822900** | **4.79433300** | **0.23850800** |
| **C** | **-1.09991300** | **3.35018100** | **-0.02729000** |
| **C** | **-1.84724800** | **4.56776100** | **-0.08580700** |
| **C** | **-1.14542400** | **5.82359900** | **-0.01679000** |
| **C** | **0.20663700** | **5.88238300** | **0.10158500** |
| **C** | **-1.78901900** | **2.13072700** | **-0.04528400** |
| **C** | **-3.18377400** | **2.05088800** | **-0.13334800** |
| **C** | **-3.92083400** | **3.27667600** | **-0.24790200** |
| **C** | **-3.23732200** | **4.49733000** | **-0.20892800** |
| **C** | **-3.91828200** | **0.78286200** | **-0.11475300** |
| **C** | **-5.34045600** | **0.80336800** | **-0.29903100** |
| **C** | **-6.02504300** | **2.06126100** | **-0.45176500** |
| **C** | **-5.35144900** | **3.23754400** | **-0.40888500** |
| **C** | **-3.29741800** | **-0.45326300** | **0.09541500** |
| **C** | **-3.99629600** | **-1.66897600** | **0.11613800** |
| **C** | **-5.41047900** | **-1.63767400** | **-0.11418700** |
| **C** | **-6.04203400** | **-0.40382500** | **-0.30877000** |
| **C** | **-3.34920100** | **-2.95523800** | **0.36880800** |
| **C** | **-4.13764900** | **-4.14395500** | **0.34179800** |
| **C** | **-5.54936700** | **-4.06010000** | **0.07878800** |
| **C** | **-6.15812900** | **-2.86476800** | **-0.13134400** |
| **H** | **5.21286700** | **-5.70236200** | **-0.55357800** |
| **H** | **1.73144900** | **-2.17342000** | **-0.58907100** |
| **H** | **6.95179000** | **-4.06181700** | **-0.15259900** |
| **H** | **7.66942500** | **-1.72676100** | **0.16907300** |
| **H** | **2.30748800** | **-0.28447600** | **-0.19493600** |
| **H** | **7.14786400** | **0.63994600** | **0.32559600** |
| **H** | **6.68176100** | **3.05950800** | **0.47206700** |
| **H** | **5.08934500** | **4.92509000** | **0.46389200** |
| **H** | **2.85934700** | **5.78888900** | **0.31313800** |
| **H** | **-1.73073600** | **6.73926100** | **-0.05404400** |
| **H** | **0.71076000** | **6.84392700** | **0.16233600** |
| **H** | **-1.19952900** | **1.22006600** | **0.01012700** |
| **H** | **-3.80878400** | **5.42092300** | **-0.27768600** |
| **H** | **-7.10378700** | **2.04499000** | **-0.58614000** |
| **H** | **-5.88108800** | **4.18209600** | **-0.50641800** |
| **H** | **-2.22847300** | **-0.47099000** | **0.26440800** |
| **H** | **-7.11914300** | **-0.38448800** | **-0.46264100** |
| **H** | **-6.12403800** | **-4.98301400** | **0.06197100** |
| **H** | **-7.22805700** | **-2.81271900** | **-0.31766700** |
| **C** | **-3.52694800** | **-5.39415400** | **0.58612600** |
| **C** | **-2.17604400** | **-5.48817700** | **0.86306600** |
| **C** | **-1.39495400** | **-4.31979300** | **0.90234400** |
| **C** | **-1.97195700** | **-3.08480600** | **0.65644500** |
| **H** | **-4.14504200** | **-6.28860000** | **0.55863900** |
| **H** | **-1.72129900** | **-6.45598600** | **1.05546200** |
| **H** | **-0.33439600** | **-4.38130500** | **1.12978500** |
| **H** | **-1.34244000** | **-2.20242100** | **0.70205400** |

B 1.41904000 2.46381000 0.06393500

Nitrogene Doped Expanded [11]-helicene:

X Y Z

**-----------------------------------------------------------------**

| **H** | **-0.47856500** | **-6.19323300** | **1.73717500** |
| --- | --- | --- | --- |
| **C** | **-1.06483200** | **-5.32976000** | **1.43523800** |
| **H** | **0.56961800** | **-3.93043100** | **1.66738200** |
| **C** | **-0.47488900** | **-4.05423600** | **1.39816200** |
| **C** | **-2.39523600** | **-5.47521400** | **1.08918400** |
| **C** | **-3.17293700** | **-4.36279700** | **0.69831200** |
| **C** | **-2.57829500** | **-3.06700000** | **0.65063200** |
| **C** | **-1.21801400** | **-2.95060600** | **1.01300000** |
| **C** | **-4.56135500** | **-4.52480400** | **0.35987400** |
| **C** | **-5.33135300** | **-3.46276100** | **0.00983100** |
| **C** | **-4.78335600** | **-2.13538000** | **-0.04886500** |
| **C** | **-3.39563600** | **-1.92376500** | **0.24984900** |
| **C** | **-2.88981700** | **-0.61917500** | **0.15347900** |
| **C** | **-5.59058800** | **-1.04079400** | **-0.38228400** |
| **C** | **-5.08829800** | **0.26056600** | **-0.43719200** |
| **C** | **-3.69519700** | **0.47156000** | **-0.18162000** |
| **C** | **-5.94351800** | **1.38476800** | **-0.72133600** |
| **C** | **-5.46147900** | **2.65317300** | **-0.72118400** |
| **C** | **-4.06834700** | **2.91665700** | **-0.47200300** |
| **C** | **-3.16086900** | **1.83106000** | **-0.24587500** |
| **C** | **-1.34823700** | **3.26994600** | **-0.07137000** |
| **C** | **-2.18695100** | **4.42442300** | **-0.20472800** |
| **C** | **-3.54801200** | **4.21055500** | **-0.42912300** |
| **C** | **0.09254600** | **3.46255300** | **0.08086100** |
| **C** | **0.60101200** | **4.79340600** | **0.20972600** |
| **C** | **-0.29422700** | **5.91766700** | **0.12058600** |
| **C** | **-1.62203200** | **5.74337200** | **-0.10011800** |
| **C** | **0.98722500** | **2.38629600** | **0.08656600** |
| **C** | **2.37005900** | **2.56139900** | **0.22854000** |
| **C** | **2.86330000** | **3.89279800** | **0.43931700** |
| **C** | **1.97266600** | **4.96974400** | **0.41154200** |
| **C** | **3.33267700** | **1.45675400** | **0.16545200** |
| **C** | **4.71762900** | **1.73055500** | **0.42001500** |
| **C** | **5.14598000** | **3.07879200** | **0.68931800** |
| **C** | **4.26718100** | **4.11058700** | **0.67688900** |
| **C** | **2.96984200** | **0.14667600** | **-0.16689000** |
| **C** | **3.89415900** | **-0.90650400** | **-0.24929000** |
| **C** | **5.26736900** | **-0.62541000** | **0.04996700** |
| **C** | **5.63961100** | **0.68393800** | **0.37363700** |
| **C** | **3.52220600** | **-2.26597000** | **-0.63828700** |
| **C** | **4.53101600** | **-3.27443100** | **-0.68363400** |
| **C** | **5.89015000** | **-2.94353900** | **-0.34955300** |
| **C** | **6.24277900** | **-1.67918500** | **-0.00337900** |
| **H** | **-2.86656400** | **-6.45501600** | **1.11817000** |
| **H** | **-0.73766000** | **-1.97818200** | **1.00305500** |
| **H** | **-4.98653100** | **-5.52482100** | **0.40130300** |
| **H** | **-6.38294700** | **-3.59629100** | **-0.23205700** |
| **H** | **-1.84642100** | **-0.41048200** | **0.35393300** |
| **H** | **-6.64532900** | **-1.20999000** | **-0.58961300** |
| **H** | **-6.99575300** | **1.19321200** | **-0.91656100** |
| **H** | **-6.11860100** | **3.49811600** | **-0.91271200** |
| **H** | **-4.21144400** | **5.06276500** | **-0.56540100** |
| **H** | **0.12426400** | **6.91648700** | **0.21645800** |
| **H** | **-2.28867600** | **6.59784600** | **-0.18862000** |
| **H** | **0.56038500** | **1.39748800** | **-0.02868000** |
| **H** | **2.36209100** | **5.97719000** | **0.54362400** |
| **H** | **6.20222700** | **3.25239800** | **0.88018900** |

| **H** | **4.60458900** | **5.12865900** | **0.85514900** |
| --- | --- | --- | --- |
| **H** | **1.93073500** | **-0.05579900** | **-0.39304500** |
| **H** | **6.68701800** | **0.89626700** | **0.57886500** |
| **H** | **6.63571000** | **-3.73411000** | **-0.38896600** |
| **H** | **7.27497800** | **-1.43719600** | **0.23791900** |
| **C** | **4.18806900** | **-4.59060800** | **-1.06454900** |
| **C** | **2.88870900** | **-4.92189400** | **-1.40015600** |
| **C** | **1.89053300** | **-3.93319400** | **-1.36038400** |
| **C** | **2.20434600** | **-2.63672500** | **-0.98700800** |
| **H** | **4.97181800** | **-5.34423200** | **-1.09255700** |
| **H** | **2.63955700** | **-5.93789800** | **-1.69374800** |
| **H** | **0.86560200** | **-4.18329100** | **-1.61724500** |
| **H** | **1.40947300** | **-1.89891700** | **-0.97085200** |
| **N** | **-1.84472800** | **2.02407200** | **-0.07857900** |

Silicone Doped Expanded [11]-helicene:

X Y Z

**-----------------------------------------------------------------**

| **H** | **4.29346800** | **6.37437900** | **0.26017500** |
| --- | --- | --- | --- |
| **C** | **4.40256100** | **5.29441600** | **0.21315200** |
| **H** | **2.27476000** | **4.90869000** | **0.28678000** |
| **C** | **3.26619600** | **4.46743400** | **0.22651100** |
| **C** | **5.65873700** | **4.72205800** | **0.14169100** |
| **C** | **5.82091200** | **3.32009500** | **0.08149100** |
| **C** | **4.67121300** | **2.47552000** | **0.09160500** |
| **C** | **3.40155100** | **3.09070600** | **0.16632300** |
| **C** | **7.13520500** | **2.73947300** | **0.01323700** |
| **C** | **7.30969400** | **1.39383900** | **-0.03928600** |
| **C** | **6.18644700** | **0.49832000** | **-0.03267600** |
| **C** | **4.85434600** | **1.02615700** | **0.02826000** |
| **C** | **3.78527300** | **0.12036900** | **0.02537100** |
| **C** | **6.36714200** | **-0.88878300** | **-0.08332200** |
| **C** | **5.29327700** | **-1.78047000** | **-0.07988800** |
| **C** | **3.95449400** | **-1.26964000** | **-0.02903800** |
| **C** | **5.50063200** | **-3.20322800** | **-0.12312900** |
| **C** | **4.45754000** | **-4.06815900** | **-0.11387700** |
| **C** | **3.08320400** | **-3.61582100** | **-0.06864800** |
| **C** | **2.82982100** | **-2.20059200** | **-0.03260700** |
| **C** | **0.00899600** | **-3.17522400** | **-0.00742900** |
| **C** | **0.68300900** | **-4.44560500** | **-0.02986700** |
| **C** | **2.08113300** | **-4.60318500** | **-0.06222400** |
| **C** | **-1.44935400** | **-3.13732100** | **0.01377200** |
| **C** | **-2.18648600** | **-4.36509700** | **0.03272300** |
| **C** | **-1.47324500** | **-5.61318100** | **0.01613600** |
| **C** | **-0.11856600** | **-5.65004100** | **-0.01796800** |
| **C** | **-2.17573900** | **-1.93658900** | **0.01496400** |
| **C** | **-3.57373200** | **-1.88289600** | **0.03964900** |
| **C** | **-4.29513400** | **-3.12188100** | **0.07309700** |
| **C** | **-3.58314500** | **-4.32452200** | **0.06546600** |
| **C** | **-4.32864200** | **-0.62526300** | **0.03302500** |
| **C** | **-5.76138000** | **-0.67790600** | **0.08033600** |
| **C** | **-6.43225200** | **-1.95180300** | **0.12241300** |
| **C** | **-5.73430800** | **-3.11428700** | **0.11473900** |
| **C** | **-3.71706900** | **0.63195000** | **-0.02170000** |
| **C** | **-4.43601400** | **1.83634100** | **-0.02819300** |
| **C** | **-5.86620300** | **1.76838300** | **0.02955200** |
| **C** | **-6.48680900** | **0.51498500** | **0.08065900** |
| **C** | **-3.79248400** | **3.14741700** | **-0.09266800** |
| **C** | **-4.60651500** | **4.31912000** | **-0.08787000** |
| **C** | **-6.03811000** | **4.19557800** | **-0.02254600** |
| **C** | **-6.63948400** | **2.97952000** | **0.03179700** |
| **H** | **6.54741600** | **5.34892700** | **0.13215600** |

| **H** | **2.50410600** | **2.48146800** | **0.18031500** |
| --- | --- | --- | --- |
| **H** | **7.99233800** | **3.40844100** | **0.00694700** |
| **H** | **8.30810200** | **0.96598100** | **-0.08799800** |
| **H** | **2.77449600** | **0.50914900** | **0.06898100** |
| **H** | **7.37978100** | **-1.28536100** | **-0.12513700** |
| **H** | **6.52273400** | **-3.57272900** | **-0.16042100** |
| **H** | **4.63600500** | **-5.14014600** | **-0.14307000** |
| **H** | **0.61844200** | **-0.36789400** | **0.03044500** |
| **H** | **2.43608500** | **-5.63115000** | **-0.08453900** |
| **H** | **-2.04958800** | **-6.53528500** | **0.02862100** |
| **H** | **0.40209800** | **-6.60423800** | **-0.03397100** |
| **H** | **-1.61887900** | **-1.00692100** | **-0.00409500** |
| **H** | **-4.13529200** | **-5.26217300** | **0.08536500** |
| **H** | **-7.51891000** | **-1.95777000** | **0.15681600** |
| **H** | **-6.25081200** | **-4.07066100** | **0.14242400** |
| **H** | **-2.63668900** | **0.67915100** | **-0.06435200** |
| **H** | **-7.57335600** | **0.46900300** | **0.11973100** |
| **H** | **-6.63227800** | **5.10621900** | **-0.01994000** |
| **H** | **-7.72272100** | **2.89820800** | **0.07832000** |
| **C** | **-3.99936800** | **5.59303000** | **-0.15023300** |
| **C** | **-2.62546700** | **5.72786500** | **-0.21870900** |
| **C** | **-1.81813100** | **4.57737500** | **-0.22618100** |
| **C** | **-2.39172400** | **3.31874100** | **-0.16373300** |
| **H** | **-4.63746200** | **6.47371600** | **-0.14486900** |
| **H** | **-2.17253600** | **6.71420000** | **-0.26768300** |
| **H** | **-0.73703000** | **4.67328300** | **-0.28362200** |
| **H** | **-1.73941400** | **2.45209500** | **-0.17281100** |
| **Si** | **1.10021500** | **-1.76226900** | **-0.00068900** |

Phosphore Doped Expanded [11]-helicene:

X Y Z

**-----------------------------------------------------------------**

| **H** | **3.55766200** | **6.36126500** | **0.71844900** |
| --- | --- | --- | --- |
| **C** | **3.74993500** | **5.29966600** | **0.59002000** |
| **H** | **1.67222600** | **4.72682500** | **0.79531700** |
| **C** | **2.68831200** | **4.37904100** | **0.62832300** |
| **C** | **5.03995200** | **4.84301100** | **0.39386400** |
| **C** | **5.30991500** | **3.46620000** | **0.23005900** |
| **C** | **4.23597100** | **2.52842000** | **0.25877600** |
| **C** | **2.92939900** | **3.02513100** | **0.46394100** |
| **C** | **6.65914300** | **3.00242600** | **0.04326400** |
| **C** | **6.93975200** | **1.68145100** | **-0.10000300** |
| **C** | **5.89494700** | **0.69518100** | **-0.08176300** |
| **C** | **4.53140400** | **1.10686500** | **0.08540000** |
| **C** | **3.53753600** | **0.12106600** | **0.07750200** |
| **C** | **6.18140500** | **-0.66743300** | **-0.22064600** |
| **C** | **5.18235100** | **-1.64364800** | **-0.21205500** |
| **C** | **3.81193600** | **-1.24523600** | **-0.07339200** |
| **C** | **5.50531900** | **-3.04013000** | **-0.33081200** |
| **C** | **4.53646200** | **-3.98668500** | **-0.30559300** |
| **C** | **3.13845900** | **-3.64011100** | **-0.18269600** |
| **C** | **2.75777400** | **-2.26535400** | **-0.08630000** |
| **C** | **0.17107600** | **-3.26155900** | **-0.01652500** |
| **C** | **0.80720300** | **-4.54070600** | **-0.06816800** |
| **C** | **2.19819600** | **-4.67731500** | **-0.15916300** |
| **C** | **-1.29261000** | **-3.20947400** | **0.03774900** |
| **C** | **-2.04235600** | **-4.42843200** | **0.09454900** |
| **C** | **-1.34952200** | **-5.68746900** | **0.06102900** |
| **C** | **0.00199400** | **-5.73975500** | **-0.02780000** |
| **C** | **-2.00453100** | **-2.00025600** | **0.03313800** |
| **C** | **-3.39889700** | **-1.93096100** | **0.09636500** |
| **C** | **-4.13416700** | **-3.15845000** | **0.18854800** |

| **C** | **-3.43767100** | **-4.36986400** | **0.17726600** |
| --- | --- | --- | --- |
| **C** | **-4.13381300** | **-0.66253500** | **0.07375100** |
| **C** | **-5.56231800** | **-0.68850000** | **0.19241800** |
| **C** | **-6.24802400** | **-1.95087600** | **0.30426400** |
| **C** | **-5.57036200** | **-3.12575900** | **0.29259200** |
| **C** | **-3.50436300** | **0.57834000** | **-0.06794500** |
| **C** | **-4.20325000** | **1.79384500** | **-0.08883400** |
| **C** | **-5.62844800** | **1.75579200** | **0.05571300** |
| **C** | **-6.26713400** | **0.51686400** | **0.18828100** |
| **C** | **-3.54169500** | **3.08677400** | **-0.25462100** |
| **C** | **-4.33214600** | **4.27422000** | **-0.24563200** |
| **C** | **-5.75834900** | **4.18247600** | **-0.08172900** |
| **C** | **-6.37769200** | **2.98202800** | **0.05752200** |
| **H** | **5.87176300** | **5.54313500** | **0.36685900** |
| **H** | **2.08883900** | **2.34075500** | **0.50366900** |
| **H** | **7.45726800** | **3.74071200** | **0.02555400** |
| **H** | **7.96463600** | **1.34378300** | **-0.23322300** |
| **H** | **2.50326300** | **0.41954800** | **0.19626500** |
| **H** | **7.21817600** | **-0.97768400** | **-0.33503300** |
| **H** | **6.55090900** | **-3.32056800** | **-0.43236700** |
| **H** | **4.79318500** | **-5.04020100** | **-0.38456800** |
| **H** | **2.58751200** | **-5.69220300** | **-0.21606100** |
| **H** | **-1.93739600** | **-6.60129600** | **0.10044700** |
| **H** | **0.51509300** | **-6.69762800** | **-0.06316100** |
| **H** | **-1.43524100** | **-1.08062800** | **-0.02088300** |
| **H** | **-3.99934400** | **-5.30024600** | **0.23358200** |
| **H** | **-7.33186100** | **-1.93872200** | **0.39020900** |
| **H** | **-6.10098000** | **-4.07175900** | **0.36778700** |
| **H** | **-2.42772400** | **0.60255200** | **-0.17278200** |
| **H** | **-7.35067300** | **0.49308100** | **0.28648800** |
| **H** | **-6.33471400** | **5.10452100** | **-0.07766900** |
| **H** | **-7.45725500** | **2.92551700** | **0.17398900** |
| **C** | **-3.70733300** | **5.53104200** | **-0.40572300** |
| **C** | **-2.33966600** | **5.63251900** | **-0.57877500** |
| **C** | **-1.55587200** | **4.46557700** | **-0.59528900** |
| **C** | **-2.14670000** | **3.22352100** | **-0.43427500** |
| **H** | **-4.32683500** | **6.42488000** | **-0.39451100** |
| **H** | **-1.87362500** | **6.60584800** | **-0.70507100** |
| **H** | **-0.48112300** | **4.53408500** | **-0.74242600** |
| **H** | **-1.51327600** | **2.34325500** | **-0.45552900** |
| **P** | **1.07736000** | **-1.75296800** | **0.00234000** |

Expanded [12]-helicene:

X Y Z

**-----------------------------------------------------------------**

| **C** | **2.79246400** | **-5.81229200** | **-1.62987200** |
| --- | --- | --- | --- |
| **C** | **3.95795200** | **-5.21453000** | **-1.21634300** |
| **C** | **3.98903200** | **-3.82771800** | **-0.89049500** |
| **C** | **1.59497500** | **-5.05422600** | **-1.73739700** |
| **C** | **1.58951400** | **-3.71525300** | **-1.43026600** |
| **C** | **2.77921300** | **-3.06012200** | **-0.99829600** |
| **C** | **5.16023400** | **-3.17075500** | **-0.47338600** |
| **C** | **5.17893500** | **-1.81245200** | **-0.17036200** |
| **C** | **3.96582200** | **-1.03512000** | **-0.26815100** |
| **C** | **2.80634400** | **-1.68655500** | **-0.67916400** |
| **C** | **6.40762200** | **-1.16928800** | **0.22592300** |
| **C** | **6.45660900** | **0.15833200** | **0.48799700** |
| **C** | **5.27745700** | **0.98346300** | **0.39883900** |
| **C** | **4.01805400** | **0.39915700** | **0.04768100** |
| **C** | **2.89324000** | **1.23222100** | **0.00770400** |
| **C** | **5.34763900** | **2.35719100** | **0.63712800** |
| **C** | **4.22603700** | **3.18857500** | **0.55652900** |

| **C** | **2.94911600** | **2.61100400** | **0.26127300** |
| --- | --- | --- | --- |
| **C** | **4.34896400** | **4.61059200** | **0.74058600** |
| **C** | **3.27506800** | **5.42902800** | **0.61515700** |
| **C** | **1.96430600** | **4.90062700** | **0.34366300** |
| **C** | **1.77451500** | **3.48593500** | **0.20959400** |
| **C** | **0.46674300** | **3.01103500** | **0.03578100** |
| **C** | **-0.64880400** | **3.85457400** | **-0.05748300** |
| **C** | **-0.42869000** | **5.27041400** | **-0.01454700** |
| **C** | **0.86389400** | **5.75124900** | **0.20812100** |
| **C** | **-2.02079500** | **3.36089600** | **-0.20809200** |
| **C** | **-3.07210900** | **4.30662300** | **-0.44060100** |
| **C** | **-2.78411500** | **5.71706000** | **-0.44273500** |
| **C** | **-1.52967500** | **6.17678200** | **-0.21139400** |
| **C** | **-2.36366800** | **2.00521600** | **-0.11409200** |
| **C** | **-3.67449500** | **1.53343000** | **-0.26684900** |
| **C** | **-4.70033300** | **2.48378400** | **-0.57923900** |
| **C** | **-4.37404000** | **3.84082600** | **-0.64338900** |
| **C** | **-4.04356500** | **0.12230100** | **-0.11558900** |
| **C** | **-5.39964400** | **-0.26927700** | **-0.37276400** |
| **C** | **-6.37780000** | **0.72391400** | **-0.73489900** |
| **C** | **-6.04881200** | **2.03648100** | **-0.81539500** |
| **C** | **-3.14013900** | **-0.86462300** | **0.29391400** |
| **C** | **-3.49469600** | **-2.21292100** | **0.45050600** |
| **C** | **-4.84366100** | **-2.59500500** | **0.15391600** |
| **C** | **-5.75688000** | **-1.61273700** | **-0.24552300** |
| **C** | **-2.55501300** | **-3.23547300** | **0.90699700** |
| **C** | **-3.00162300** | **-4.58596000** | **1.01802800** |
| **C** | **-4.36162200** | **-4.91941100** | **0.68938000** |
| **C** | **-5.24201100** | **-3.96970200** | **0.28149700** |
| **H** | **2.78027300** | **-6.87035100** | **-1.87743800** |
| **H** | **4.87678800** | **-5.79040800** | **-1.13350900** |
| **H** | **6.08424400** | **-3.74026900** | **-0.39416100** |
| **H** | **1.87569700** | **-1.13654500** | **-0.78098800** |
| **H** | **7.30362700** | **-1.78112100** | **0.29675400** |
| **H** | **7.39249400** | **0.63336100** | **0.77188400** |
| **H** | **1.93723300** | **0.79544800** | **-0.25275200** |
| **H** | **6.31188500** | **2.79969000** | **0.87927000** |
| **H** | **5.33339800** | **5.01620100** | **0.96066200** |
| **H** | **3.38061800** | **6.50505700** | **0.72954400** |
| **H** | **0.31145400** | **1.94160200** | **-0.03508300** |
| **H** | **1.02020300** | **6.82613300** | **0.27433600** |
| **H** | **-3.60612000** | **6.40810800** | **-0.61250600** |
| **H** | **-1.32512000** | **7.24434600** | **-0.18837400** |
| **H** | **-1.57903400** | **1.29054100** | **0.10047200** |
| **H** | **-5.16334000** | **4.56144900** | **-0.84812000** |
| **H** | **-7.39664200** | **0.39474400** | **-0.92389600** |
| **H** | **-6.79890600** | **2.78197800** | **-1.06778800** |
| **H** | **-2.12261600** | **-0.57131200** | **0.51799100** |
| **H** | **-6.78464000** | **-1.90487100** | **-0.45197400** |
| **H** | **-4.67248500** | **-5.95723900** | **0.78261500** |
| **H** | **-6.27010700** | **-4.23161000** | **0.04355500** |
| **C** | **-2.10457800** | **-5.58513900** | **1.45694900** |
| **C** | **-0.79765000** | **-5.27837600** | **1.78592200** |
| **C** | **-0.35266900** | **-3.94892600** | **1.68495900** |
| **C** | **-1.21495500** | **-2.95391300** | **1.25589200** |
| **H** | **0.67932000** | **-5.54095300** | **-2.06102400** |
| **H** | **0.67154200** | **-3.13826400** | **-1.50561500** |
| **H** | **-2.46436700** | **-6.60866800** | **1.53414100** |
| **H** | **-0.11794800** | **-6.05693400** | **2.12051300** |
| **H** | **0.67303300** | **-3.69837600** | **1.93911000** |
| **H** | **-0.84072100** | **-1.93734500** | **1.19670900** |

Hydroxyl Functionalized Expanded [12]-helicene:

X Y Z

**-----------------------------------------------------------------**

| **C** | **-2.79868500** | **-5.66288200** | **-1.82124900** |
| --- | --- | --- | --- |
| **C** | **-3.92900400** | **-5.09533300** | **-1.28556800** |
| **C** | **-3.95348300** | **-3.71480600** | **-0.93303200** |
| **C** | **-1.63127300** | **-4.87973200** | **-2.03118300** |
| **C** | **-1.61754300** | **-3.54685500** | **-1.69886000** |
| **C** | **-2.77120200** | **-2.92356500** | **-1.13877700** |
| **C** | **-5.09615200** | **-3.08383600** | **-0.40931200** |
| **C** | **-5.11466400** | **-1.72875600** | **-0.08965800** |
| **C** | **-3.92462800** | **-0.93254400** | **-0.26932400** |
| **C** | **-2.79197500** | **-1.55580000** | **-0.78996200** |
| **C** | **-6.32276700** | **-1.10326000** | **0.39116800** |
| **C** | **-6.37584200** | **0.22565400** | **0.64951900** |
| **C** | **-5.21598500** | **1.06767300** | **0.49048800** |
| **C** | **-3.97279000** | **0.49452900** | **0.07117500** |
| **C** | **-2.85401700** | **1.32975900** | **-0.00666000** |
| **C** | **-5.28441900** | **2.44227000** | **0.73096500** |
| **C** | **-4.17055100** | **3.27970900** | **0.60248500** |
| **C** | **-2.90466400** | **2.70399800** | **0.26337200** |
| **C** | **-4.28159900** | **4.70371800** | **0.78423100** |
| **C** | **-3.20814200** | **5.51853100** | **0.62277300** |
| **C** | **-1.90428800** | **4.98671000** | **0.32270900** |
| **C** | **-1.72910100** | **3.57140600** | **0.18849200** |
| **C** | **-0.43174200** | **3.07960300** | **0.00086200** |
| **C** | **0.69111500** | **3.90838900** | **-0.10471500** |
| **C** | **0.48967100** | **5.32691200** | **-0.06594000** |
| **C** | **-0.79576300** | **5.82513500** | **0.16812000** |
| **C** | **2.05159100** | **3.38859600** | **-0.25596000** |
| **C** | **3.12092000** | **4.31338800** | **-0.48423800** |
| **C** | **2.85413700** | **5.72851100** | **-0.49575300** |
| **C** | **1.60668000** | **6.21233300** | **-0.27068400** |
| **C** | **2.36188900** | **2.02582300** | **-0.15960500** |
| **C** | **3.66303400** | **1.52593100** | **-0.29671700** |
| **C** | **4.71414600** | **2.45687800** | **-0.58636100** |
| **C** | **4.41625700** | **3.82049700** | **-0.66539800** |
| **C** | **3.99600700** | **0.10657100** | **-0.13642600** |
| **C** | **5.35620700** | **-0.30221700** | **-0.31438000** |
| **C** | **6.36566000** | **0.66802000** | **-0.65091300** |
| **C** | **6.06151000** | **1.98368400** | **-0.77233100** |
| **C** | **3.04368500** | **-0.86131700** | **0.21140800** |
| **C** | **3.35931100** | **-2.21777700** | **0.41272700** |
| **C** | **4.73430600** | **-2.60386300** | **0.24189800** |
| **C** | **5.68297200** | **-1.64535100** | **-0.12796000** |
| **C** | **2.38903300** | **-3.24232900** | **0.81696200** |
| **C** | **2.86101800** | **-4.56927500** | **1.08264400** |
| **C** | **4.25013500** | **-4.88941700** | **0.89411000** |
| **C** | **5.14204000** | **-3.95852700** | **0.47885100** |
| **H** | **-2.79179300** | **-6.71592000** | **-2.08905400** |
| **H** | **-4.82557500** | **-5.69001200** | **-1.12701100** |
| **H** | **-6.00176400** | **-3.67020800** | **-0.26629700** |
| **H** | **-1.89188100** | **-0.98031600** | **-0.98802000** |
| **H** | **-7.20326100** | **-1.72766700** | **0.52109300** |
| **H** | **-7.30011400** | **0.68668000** | **0.98906300** |
| **H** | **-1.90700100** | **0.89955400** | **-0.30664500** |
| **H** | **-6.23932500** | **2.87867100** | **1.01719800** |
| **H** | **-5.25689100** | **5.11524400** | **1.03226000** |
| **H** | **-3.30932900** | **6.59519800** | **0.73579200** |
| **H** | **-0.28846800** | **2.00862500** | **-0.06150800** |
| **H** | **-0.93782100** | **6.90203400** | **0.23452200** |
| **H** | **3.68761000** | **6.40564600** | **-0.66635200** |
| **H** | **1.42229600** | **7.28372500** | **-0.25452300** |
| **H** | **1.56357800** | **1.32727100** | **0.05507400** |
| **H** | **5.22422400** | **4.52380800** | **-0.85754700** |

| **H** | **7.38632000** | **0.31952800** | **-0.78947300** |
| --- | --- | --- | --- |
| **H** | **6.83302200** | **2.71227200** | **-1.00936100** |
| **H** | **2.02125200** | **-0.55706400** | **0.35613200** |
| **H** | **6.71807900** | **-1.95769400** | **-0.25174100** |
| **H** | **4.56632100** | **-5.91050500** | **1.09182300** |
| **H** | **6.18890800** | **-4.21465200** | **0.33510600** |
| **C** | **1.98830400** | **-5.58738100** | **1.52089100** |
| **C** | **0.64372600** | **-5.33206900** | **1.69825700** |
| **C** | **0.14677500** | **-4.05544000** | **1.41983700** |
| **C** | **0.98671700** | **-3.03802600** | **0.97837600** |
| **H** | **-0.74417500** | **-5.34282800** | **-2.45367200** |
| **H** | **-0.72253700** | **-2.94928900** | **-1.85223200** |
| **H** | **2.39561600** | **-6.57573800** | **1.71529700** |
| **H** | **-0.03189600** | **-6.11118900** | **2.03910700** |
| **H** | **-0.91574700** | **-3.84957800** | **1.52898800** |
| **O** | **0.43797400** | **-1.81457100** | **0.67776800** |
| **H** | **-0.52285500** | **-1.87225200** | **0.81171400** |

Methyl Functionalized Expanded [12]-helicene:

X Y Z

**-----------------------------------------------------------------**

| **C** | **-2.31156100** | **-5.97382900** | **-1.53587600** |
| --- | --- | --- | --- |
| **C** | **-3.53535100** | **-5.45635900** | **-1.18743000** |
| **C** | **-3.68186400** | **-4.07047400** | **-0.89068500** |
| **C** | **-1.16829000** | **-5.13216000** | **-1.60394000** |
| **C** | **-1.27458100** | **-3.79121300** | **-1.32499000** |
| **C** | **-2.52746100** | **-3.21790000** | **-0.96142300** |
| **C** | **-4.91454200** | **-3.49522800** | **-0.53421600** |
| **C** | **-5.04466900** | **-2.13796200** | **-0.25470900** |
| **C** | **-3.88880100** | **-1.27477600** | **-0.31913300** |
| **C** | **-2.66905000** | **-1.84503700** | **-0.67172800** |
| **C** | **-6.33083000** | **-1.58214600** | **0.08767700** |
| **C** | **-6.48533700** | **-0.25963900** | **0.33454900** |
| **C** | **-5.36684500** | **0.64864700** | **0.27802700** |
| **C** | **-4.05750600** | **0.15552700** | **-0.02726400** |
| **C** | **-2.99663400** | **1.06913200** | **-0.04486600** |
| **C** | **-5.54371500** | **2.01470200** | **0.50496100** |
| **C** | **-4.48347000** | **2.92523400** | **0.45319100** |
| **C** | **-3.16036000** | **2.44183300** | **0.19415500** |
| **C** | **-4.71383200** | **4.33435900** | **0.63288200** |
| **C** | **-3.69864400** | **5.22839100** | **0.53812600** |
| **C** | **-2.34693100** | **4.79710000** | **0.29774700** |
| **C** | **-2.05218000** | **3.40044900** | **0.16450100** |
| **C** | **-0.71063900** | **3.02302100** | **0.00971400** |
| **C** | **0.34203600** | **3.94555500** | **-0.06259000** |
| **C** | **0.01940700** | **5.34144000** | **-0.01286800** |
| **C** | **-1.30843900** | **5.72583200** | **0.18845800** |
| **C** | **1.74765300** | **3.55305600** | **-0.20169100** |
| **C** | **2.73057800** | **4.57461300** | **-0.41214200** |
| **C** | **2.34221400** | **5.96073500** | **-0.40163000** |
| **C** | **1.05493100** | **6.32672900** | **-0.18407300** |
| **C** | **2.18569800** | **2.22420400** | **-0.12131300** |
| **C** | **3.52741600** | **1.84775800** | **-0.27345500** |
| **C** | **4.48526800** | **2.87402600** | **-0.56309000** |
| **C** | **4.06393300** | **4.20552400** | **-0.60834200** |
| **C** | **3.99674000** | **0.46454800** | **-0.14418400** |
| **C** | **5.37233800** | **0.17618300** | **-0.41221400** |
| **C** | **6.28296500** | **1.24026400** | **-0.74522100** |
| **C** | **5.86346000** | **2.52840300** | **-0.79760900** |
| **C** | **3.16217900** | **-0.58867800** | **0.25357000** |
| **C** | **3.59419300** | **-1.91903700** | **0.40158200** |
| **C** | **4.95287400** | **-2.19894200** | **0.01748000** |

| **C** | **5.80131600** | **-1.14970800** | **-0.35037500** |
| --- | --- | --- | --- |
| **C** | **2.74371200** | **-3.02314200** | **0.87621400** |
| **C** | **3.25668600** | **-4.35531300** | **0.73041100** |
| **C** | **4.59977800** | **-4.58024200** | **0.26772600** |
| **C** | **5.42795200** | **-3.55106200** | **-0.02931300** |
| **H** | **-2.21137400** | **-7.03223000** | **-1.76113800** |
| **H** | **-4.41267600** | **-6.09683100** | **-1.13383800** |
| **H** | **-5.79691200** | **-4.13012900** | **-0.48176900** |
| **H** | **-1.77751100** | **-1.22962000** | **-0.74537500** |
| **H** | **-7.18219800** | **-2.25691400** | **0.13308600** |
| **H** | **-7.46280300** | **0.14793800** | **0.58067400** |
| **H** | **-2.00421700** | **0.70154800** | **-0.27353300** |
| **H** | **-6.54431300** | **2.38681200** | **0.71606000** |
| **H** | **-5.73050700** | **4.66744200** | **0.82653900** |
| **H** | **-3.88458100** | **6.29358800** | **0.65150600** |
| **H** | **-0.47677300** | **1.96839500** | **-0.06594800** |
| **H** | **-1.54357000** | **6.78617300** | **0.25620500** |
| **H** | **3.11458200** | **6.71085100** | **-0.55283100** |
| **H** | **0.77374600** | **7.37653000** | **-0.15389900** |
| **H** | **1.45218200** | **1.45344800** | **0.07975800** |
| **H** | **4.80166300** | **4.98332700** | **-0.79548300** |
| **H** | **7.32227200** | **0.98584900** | **-0.93820200** |
| **H** | **6.56035200** | **3.33025600** | **-1.02894300** |
| **H** | **2.12966500** | **-0.35739700** | **0.44266500** |
| **H** | **6.83330500** | **-1.38117700** | **-0.60658100** |
| **H** | **4.94568700** | **-5.60847500** | **0.19591900** |
| **H** | **6.45430600** | **-3.72890600** | **-0.34050200** |
| **C** | **2.47747900** | **-5.47745100** | **1.08013700** |
| **C** | **1.21565700** | **-5.31878700** | **1.61181200** |
| **C** | **0.73768300** | **-4.02376500** | **1.84038500** |
| **C** | **1.46253100** | **-2.88068000** | **1.50466300** |
| **H** | **-0.20479500** | **-5.55573000** | **-1.87244000** |
| **H** | **-0.39807100** | **-3.15004400** | **-1.37098200** |
| **H** | **2.90019800** | **-6.46896700** | **0.93713400** |
| **H** | **0.61015200** | **-6.17947600** | **1.88109500** |
| **H** | **-0.23390300** | **-3.89771900** | **2.31055100** |
| **C** | **0.82868900** | **-1.56518400** | **1.91260100** |
| **H** | **1.54511400** | **-0.88650200** | **2.38599500** |
| **H** | **0.37287200** | **-1.03075800** | **1.06847700** |
| **H** | **0.02508400** | **-1.75258900** | **2.63062500** |

Methoxy Functionalized Expanded [12]-helicene:

X Y Z

**-----------------------------------------------------------------**

| **C** | **-2.07152800** | **-5.97080100** | **-1.69525900** |
| --- | --- | --- | --- |
| **C** | **-3.31578700** | **-5.50120200** | **-1.35152500** |
| **C** | **-3.51264100** | **-4.12583400** | **-1.03531200** |
| **C** | **-0.95711200** | **-5.08960400** | **-1.73975400** |
| **C** | **-1.11169100** | **-3.75734200** | **-1.44254700** |
| **C** | **-2.38735300** | **-3.23367300** | **-1.08249400** |
| **C** | **-4.76707700** | **-3.59852900** | **-0.67989700** |
| **C** | **-4.94573700** | **-2.25151200** | **-0.37744500** |
| **C** | **-3.81963200** | **-1.34872400** | **-0.41877500** |
| **C** | **-2.57776700** | **-1.87075000** | **-0.77203700** |
| **C** | **-6.25159000** | **-1.74554300** | **-0.03154600** |
| **C** | **-6.45131300** | **-0.43434400** | **0.24276000** |
| **C** | **-5.36394800** | **0.51225800** | **0.21070900** |
| **C** | **-4.03845500** | **0.06911500** | **-0.10128600** |
| **C** | **-3.00864100** | **1.01728400** | **-0.09916600** |
| **C** | **-5.58598200** | **1.86685900** | **0.46694400** |
| **C** | **-4.55583000** | **2.81279200** | **0.43788900** |
| **C** | **-3.21802700** | **2.37811400** | **0.16959500** |

| **C** | **-4.82912000** | **4.21018400** | **0.64863600** |
| --- | --- | --- | --- |
| **C** | **-3.84178300** | **5.13732000** | **0.57381800** |
| **C** | **-2.47703200** | **4.75486200** | **0.32345400** |
| **C** | **-2.14090200** | **3.37096400** | **0.16087000** |
| **C** | **-0.78997800** | **3.03620100** | **-0.00317500** |
| **C** | **0.23394700** | **3.99069200** | **-0.05968900** |
| **C** | **-0.12840100** | **5.37532100** | **0.01582800** |
| **C** | **-1.46658800** | **5.71648500** | **0.22995500** |
| **C** | **1.64745600** | **3.63844200** | **-0.21340100** |
| **C** | **2.60048300** | **4.68700100** | **-0.42529200** |
| **C** | **2.17391900** | **6.06168800** | **-0.38735900** |
| **C** | **0.87983400** | **6.39023500** | **-0.14764300** |
| **C** | **2.11833000** | **2.32069600** | **-0.14920900** |
| **C** | **3.46568800** | **1.98081900** | **-0.32588000** |
| **C** | **4.39133200** | **3.03046000** | **-0.63428900** |
| **C** | **3.93853700** | **4.35281300** | **-0.65393000** |
| **C** | **3.96543700** | **0.60744100** | **-0.21401000** |
| **C** | **5.33331400** | **0.34554500** | **-0.54213600** |
| **C** | **6.20923800** | **1.43032800** | **-0.90256900** |
| **C** | **5.76669800** | **2.71222600** | **-0.92080600** |
| **C** | **3.16361400** | **-0.45280300** | **0.22616400** |
| **C** | **3.62465800** | **-1.77800600** | **0.33962300** |
| **C** | **4.97932900** | **-2.03238500** | **-0.07526000** |
| **C** | **5.79240900** | **-0.97020100** | **-0.48582500** |
| **C** | **2.82059000** | **-2.90065400** | **0.84011900** |
| **C** | **3.38947800** | **-4.21372100** | **0.80728500** |
| **C** | **4.73277700** | **-4.40873600** | **0.33317900** |
| **C** | **5.50048000** | **-3.36798900** | **-0.06918600** |
| **H** | **-1.93267800** | **-7.02161300** | **-1.93490500** |
| **H** | **-4.17088500** | **-6.17226000** | **-1.31626400** |
| **H** | **-5.62752400** | **-4.26401600** | **-0.64491500** |
| **H** | **-1.70616300** | **-1.22563200** | **-0.82834100** |
| **H** | **-7.08029100** | **-2.44891800** | **-0.00493500** |
| **H** | **-7.44312400** | **-0.06537800** | **0.49242100** |
| **H** | **-2.00543800** | **0.68869000** | **-0.33965200** |
| **H** | **-6.59876300** | **2.20122100** | **0.68315100** |
| **H** | **-5.85544200** | **4.50784400** | **0.84900200** |
| **H** | **-4.06161400** | **6.19334500** | **0.71036100** |
| **H** | **-0.52410400** | **1.99154200** | **-0.10517600** |
| **H** | **-1.73308100** | **6.76792000** | **0.31798400** |
| **H** | **2.92329000** | **6.83531500** | **-0.53634500** |
| **H** | **0.57220700** | **7.43182800** | **-0.09703000** |
| **H** | **1.41052100** | **1.52645000** | **0.05116700** |
| **H** | **4.65283600** | **5.14999200** | **-0.85059500** |
| **H** | **7.24485400** | **1.19769100** | **-1.13876600** |
| **H** | **6.44101700** | **3.52851300** | **-1.16828300** |
| **H** | **2.14773300** | **-0.24855700** | **0.51428400** |
| **H** | **6.82318100** | **-1.18150700** | **-0.76370900** |
| **H** | **5.12547100** | **-5.42238700** | **0.32661700** |
| **H** | **6.52310000** | **-3.52511200** | **-0.40346700** |
| **C** | **2.66752700** | **-5.33998000** | **1.25894800** |
| **C** | **1.39374300** | **-5.19784500** | **1.76372200** |
| **C** | **0.81485000** | **-3.92454700** | **1.83973900** |
| **C** | **1.50273900** | **-2.79853200** | **1.39599700** |
| **H** | **0.02242300** | **-5.47624000** | **-2.00600300** |
| **H** | **-0.25848600** | **-3.08443500** | **-1.47143500** |
| **H** | **3.14009900** | **-6.31718100** | **1.20873800** |
| **H** | **0.83312100** | **-6.06141300** | **2.11018200** |
| **H** | **-0.18585400** | **-3.82505600** | **2.24018500** |
| **O** | **0.94341300** | **-1.55246000** | **1.48571400** |
| **C** | **-0.25170000** | **-1.38056800** | **2.23932500** |
| **H** | **-0.42652000** | **-0.30339900** | **2.27254300** |
| **H** | **-1.10788100** | **-1.86910800** | **1.75962600** |

H -0.13404600 -1.76236700 3.26071800

Amino Functionalized Expanded [12]-helicene:

X Y Z

**-----------------------------------------------------------------**

| **C** | **2.96288000** | **-5.67470700** | **1.67881600** |
| --- | --- | --- | --- |
| **C** | **4.11404200** | **-5.04150100** | **1.27768000** |
| **C** | **4.09902000** | **-3.66213100** | **0.92090300** |
| **C** | **1.73464900** | **-4.96223400** | **1.74023500** |
| **C** | **1.68387900** | **-3.63223200** | **1.39943100** |
| **C** | **2.85781800** | **-2.94065700** | **0.98155600** |
| **C** | **5.25327400** | **-2.96916000** | **0.51509300** |
| **C** | **5.22807800** | **-1.61557300** | **0.19231700** |
| **C** | **3.98582800** | **-0.88164600** | **0.25646400** |
| **C** | **2.83840900** | **-1.57267500** | **0.63700500** |
| **C** | **6.43933700** | **-0.93512500** | **-0.19531300** |
| **C** | **6.44670800** | **0.39024900** | **-0.47272900** |
| **C** | **5.24129000** | **1.17782300** | **-0.39637100** |
| **C** | **3.99780300** | **0.55479400** | **-0.05447100** |
| **C** | **2.85046600** | **1.35612800** | **-0.00504900** |
| **C** | **5.27251200** | **2.55421900** | **-0.62769800** |
| **C** | **4.12849300** | **3.35361700** | **-0.53909200** |
| **C** | **2.86926700** | **2.73880000** | **-0.24331100** |
| **C** | **4.21181700** | **4.78028900** | **-0.70916700** |
| **C** | **3.11749200** | **5.56851000** | **-0.56676000** |
| **C** | **1.82355800** | **5.00264900** | **-0.29010100** |
| **C** | **1.67297500** | **3.58194600** | **-0.17075800** |
| **C** | **0.38068500** | **3.06942400** | **0.01028500** |
| **C** | **-0.75616300** | **3.88165400** | **0.12110900** |
| **C** | **-0.57592400** | **5.30329900** | **0.08945600** |
| **C** | **0.70159600** | **5.82166400** | **-0.13639600** |
| **C** | **-2.11299600** | **3.34787500** | **0.27033900** |
| **C** | **-3.19381400** | **4.26157100** | **0.49660700** |
| **C** | **-2.94472900** | **5.67940900** | **0.51447700** |
| **C** | **-1.70222000** | **6.17625400** | **0.29510700** |
| **C** | **-2.41292500** | **1.98308400** | **0.17154300** |
| **C** | **-3.71165600** | **1.47239900** | **0.30227600** |
| **C** | **-4.77366900** | **2.39291600** | **0.58255600** |
| **C** | **-4.48618600** | **3.75807400** | **0.66745000** |
| **C** | **-4.03478300** | **0.05259500** | **0.13584600** |
| **C** | **-5.39101600** | **-0.37094400** | **0.30385000** |
| **C** | **-6.41327400** | **0.59121400** | **0.62269300** |
| **C** | **-6.11988000** | **1.90924000** | **0.74863600** |
| **C** | **-3.07382800** | **-0.90653300** | **-0.21182900** |
| **C** | **-3.37345100** | **-2.26406800** | **-0.43055900** |
| **C** | **-4.72668900** | **-2.68068700** | **-0.17427600** |
| **C** | **-5.69106900** | **-1.72765900** | **0.16738300** |
| **C** | **-2.39061300** | **-3.26923400** | **-0.85205300** |
| **C** | **-2.77364600** | **-4.64897500** | **-0.80444400** |
| **C** | **-4.12886400** | **-5.00969700** | **-0.48286800** |
| **C** | **-5.07197900** | **-4.07237300** | **-0.22282600** |
| **H** | **2.98614500** | **-6.72630700** | **1.95208700** |
| **H** | **5.05636900** | **-5.58243100** | **1.23027000** |
| **H** | **6.19902700** | **-3.50516100** | **0.46245700** |
| **H** | **1.87958000** | **-1.06544100** | **0.68505800** |
| **H** | **7.35641900** | **-1.51688600** | **-0.24806800** |
| **H** | **7.36939700** | **0.89344300** | **-0.75134400** |
| **H** | **1.90876400** | **0.89162900** | **0.25945900** |
| **H** | **6.22445700** | **3.02553400** | **-0.86425000** |
| **H** | **5.18350000** | **5.21460200** | **-0.93126900** |
| **H** | **3.19378300** | **6.64815900** | **-0.67000300** |
| **H** | **0.25536200** | **1.99538800** | **0.06992900** |

| **H** | **0.82801300** | **6.90104700** | **-0.19309700** |
| --- | --- | --- | --- |
| **H** | **-3.78656400** | **6.34597800** | **0.68520300** |
| **H** | **-1.52772800** | **7.24935700** | **0.28388100** |
| **H** | **-1.60570200** | **1.29351700** | **-0.04106900** |
| **H** | **-5.30062800** | **4.45533300** | **0.85407900** |
| **H** | **-7.43251400** | **0.23480900** | **0.75076900** |
| **H** | **-6.89979000** | **2.63201700** | **0.97560400** |
| **H** | **-2.04200000** | **-0.59910400** | **-0.27086100** |
| **H** | **-6.71225700** | **-2.06040600** | **0.34308200** |
| **H** | **-4.38422000** | **-6.06644800** | **-0.48087300** |
| **H** | **-6.09997700** | **-4.35750400** | **-0.01296700** |
| **C** | **-1.85251100** | **-5.67461700** | **-1.09844200** |
| **C** | **-0.56029500** | **-5.36406500** | **-1.47482500** |
| **C** | **-0.18799600** | **-4.02560200** | **-1.61540900** |
| **C** | **-1.07324900** | **-2.98151200** | **-1.33748500** |
| **H** | **0.83068900** | **-5.47687600** | **2.05316900** |
| **H** | **0.74363500** | **-3.08902300** | **1.43950000** |
| **H** | **-2.18023200** | **-6.70854300** | **-1.03221200** |
| **H** | **0.15836300** | **-6.14828600** | **-1.69509400** |
| **H** | **0.81359900** | **-3.77967100** | **-1.96051300** |
| **N** | **-0.60831700** | **-1.66309800** | **-1.53653400** |
| **H** | **0.25944900** | **-1.65130500** | **-2.06386400** |
| **H** | **-1.28745200** | **-1.06226800** | **-1.99459900** |

Carboxyl Functionalized Expanded [12]-helicene:

X Y Z

**-----------------------------------------------------------------**

| **C** | **-3.12305100** | **-5.60830700** | **-1.78477000** |
| --- | --- | --- | --- |
| **C** | **-4.26412800** | **-4.94785100** | **-1.39898900** |
| **C** | **-4.22194000** | **-3.56863700** | **-1.04350400** |
| **C** | **-1.87783000** | **-4.92439100** | **-1.83153700** |
| **C** | **-1.80173500** | **-3.59467400** | **-1.49402700** |
| **C** | **-2.96479800** | **-2.87526100** | **-1.09333700** |
| **C** | **-5.36300700** | **-2.85033000** | **-0.64439300** |
| **C** | **-5.30736100** | **-1.49975200** | **-0.31279600** |
| **C** | **-4.04828100** | **-0.79408000** | **-0.36770700** |
| **C** | **-2.91677300** | **-1.50824100** | **-0.75028800** |
| **C** | **-6.50306800** | **-0.79590600** | **0.08042200** |
| **C** | **-6.47822900** | **0.52453000** | **0.37870300** |
| **C** | **-5.25368700** | **1.28334900** | **0.31645900** |
| **C** | **-4.02493100** | **0.63835800** | **-0.03768500** |
| **C** | **-2.85911800** | **1.41578400** | **-0.07583100** |
| **C** | **-5.25339100** | **2.65523600** | **0.57281600** |
| **C** | **-4.09265200** | **3.43082200** | **0.49623400** |
| **C** | **-2.84671100** | **2.79542200** | **0.18591500** |
| **C** | **-4.14999900** | **4.85554900** | **0.68968200** |
| **C** | **-3.04335600** | **5.62615300** | **0.55085800** |
| **C** | **-1.76221200** | **5.04076100** | **0.25719200** |
| **C** | **-1.63461800** | **3.61869200** | **0.12130700** |
| **C** | **-0.34948200** | **3.09009300** | **-0.07028100** |
| **C** | **0.79805200** | **3.88872000** | **-0.18295400** |
| **C** | **0.63669500** | **5.31223300** | **-0.14424600** |
| **C** | **-0.63080400** | **5.84546200** | **0.09988000** |
| **C** | **2.14770900** | **3.34185500** | **-0.34864500** |
| **C** | **3.23361400** | **4.24221600** | **-0.60289300** |
| **C** | **3.00106200** | **5.66274600** | **-0.61419800** |
| **C** | **1.76941300** | **6.17274600** | **-0.36619700** |
| **C** | **2.43679200** | **1.97587500** | **-0.24191400** |
| **C** | **3.72531600** | **1.45112700** | **-0.40605700** |
| **C** | **4.78823400** | **2.35520400** | **-0.72789600** |
| **C** | **4.51551100** | **3.72354800** | **-0.80706900** |
| **C** | **4.03593000** | **0.02908700** | **-0.24314700** |

| **C** | **5.37607200** | **-0.42249700** | **-0.47836000** |
| --- | --- | --- | --- |
| **C** | **6.39749600** | **0.52650000** | **-0.83877000** |
| **C** | **6.11936000** | **1.84963200** | **-0.94553000** |
| **C** | **3.08132100** | **-0.91307200** | **0.14937400** |
| **C** | **3.37514200** | **-2.26934600** | **0.34421300** |
| **C** | **4.69183200** | **-2.72349400** | **0.00371300** |
| **C** | **5.65994800** | **-1.78595600** | **-0.37411500** |
| **C** | **2.38712000** | **-3.24142200** | **0.80636200** |
| **C** | **2.68741900** | **-4.62915000** | **0.66343400** |
| **C** | **4.00659500** | **-5.03930000** | **0.26005200** |
| **C** | **4.97892800** | **-4.13037400** | **-0.00505500** |
| **H** | **-3.16728700** | **-6.65962300** | **-2.05660900** |
| **H** | **-5.21878800** | **-5.46749300** | **-1.36125400** |
| **H** | **-6.32117300** | **-3.36441600** | **-0.59900200** |
| **H** | **-1.94704800** | **-1.02457100** | **-0.78549200** |
| **H** | **-7.43402200** | **-1.35598700** | **0.12365100** |
| **H** | **-7.38841000** | **1.04605000** | **0.66456100** |
| **H** | **-1.92989700** | **0.93553800** | **-0.35733500** |
| **H** | **-6.19423300** | **3.14354400** | **0.81898600** |
| **H** | **-5.11270400** | **5.30293700** | **0.92433500** |
| **H** | **-3.09909300** | **6.70544200** | **0.66920900** |
| **H** | **-0.23861900** | **2.01459900** | **-0.14166400** |
| **H** | **-0.74255200** | **6.92590100** | **0.16549400** |
| **H** | **3.84659600** | **6.31997700** | **-0.80162400** |
| **H** | **1.60735700** | **7.24765500** | **-0.34768500** |
| **H** | **1.62979000** | **1.29941300** | **0.00825000** |
| **H** | **5.33216500** | **4.41086100** | **-1.01878700** |
| **H** | **7.40473000** | **0.15592400** | **-1.01235900** |
| **H** | **6.90121900** | **2.56037400** | **-1.20186000** |
| **H** | **2.06207100** | **-0.58271300** | **0.28104200** |
| **H** | **6.66335500** | **-2.13449300** | **-0.61000400** |
| **H** | **4.21300100** | **-6.10491000** | **0.19943100** |
| **H** | **5.98180500** | **-4.45182900** | **-0.27479300** |
| **C** | **1.70363000** | **-5.60202400** | **0.94766900** |
| **C** | **0.44252000** | **-5.23704400** | **1.37467800** |
| **C** | **0.16055700** | **-3.88192900** | **1.60004500** |
| **C** | **1.12107000** | **-2.90115600** | **1.36673500** |
| **H** | **-0.98232000** | **-5.46040500** | **-2.13327000** |
| **H** | **-0.84919000** | **-3.07235300** | **-1.52239300** |
| **H** | **1.95910100** | **-6.65121500** | **0.81989200** |
| **H** | **-0.31721200** | **-5.98793700** | **1.56880200** |
| **H** | **-0.80535300** | **-3.59019600** | **1.99932400** |
| **C** | **0.80940500** | **-1.55464800** | **1.94525200** |
| **O** | **1.45651700** | **-0.99266000** | **2.79933200** |
| **O** | **-0.35794600** | **-1.03757700** | **1.46493500** |
| **H** | **-0.53532200** | **-0.23531100** | **1.99420600** |

Bor Doped Expanded [12]-helicene:

X Y Z

**-----------------------------------------------------------------**

| **C** | **3.29205800** | **-5.85195700** | **-1.20474500** |
| --- | --- | --- | --- |
| **C** | **4.44370900** | **-5.16683300** | **-0.90276200** |
| **C** | **4.41425800** | **-3.76270600** | **-0.66193700** |
| **C** | **2.04846000** | **-5.16802000** | **-1.28162200** |
| **C** | **1.98454600** | **-3.81409700** | **-1.05624200** |
| **C** | **3.15763400** | **-3.07010700** | **-0.73812700** |
| **C** | **5.56849900** | **-3.01870200** | **-0.35959700** |
| **C** | **5.52629300** | **-1.64554000** | **-0.13589400** |
| **C** | **4.26549200** | **-0.94436200** | **-0.19804100** |
| **C** | **3.12361800** | **-1.68069900** | **-0.49978100** |
| **C** | **6.73630700** | **-0.91183500** | **0.14157400** |
| **C** | **6.72067700** | **0.42980400** | **0.32625000** |

| **C** | **5.49154900** | **1.18142600** | **0.27348700** |
| --- | --- | --- | --- |
| **C** | **4.24920600** | **0.50549700** | **0.03737500** |
| **C** | **3.07675000** | **1.27012600** | **0.03120300** |
| **C** | **5.49795200** | **2.56859600** | **0.44459100** |
| **C** | **4.32645000** | **3.32939100** | **0.40165100** |
| **C** | **3.07707700** | **2.66163700** | **0.20856300** |
| **C** | **4.34680600** | **4.76270200** | **0.53244500** |
| **C** | **3.21014400** | **5.50163800** | **0.45981500** |
| **C** | **1.91279300** | **4.89611700** | **0.27794100** |
| **C** | **1.86258700** | **3.44285800** | **0.18058700** |
| **C** | **-0.85129400** | **3.78547300** | **-0.01173600** |
| **C** | **-0.54874100** | **5.21012900** | **0.04339400** |
| **C** | **0.76072100** | **5.70039800** | **0.19850200** |
| **C** | **-2.21568900** | **3.32748200** | **-0.13864200** |
| **C** | **-3.25887000** | **4.29395200** | **-0.29559800** |
| **C** | **-2.93178200** | **5.69568600** | **-0.26182200** |
| **C** | **-1.65703500** | **6.12650400** | **-0.08285500** |
| **C** | **-2.55606800** | **1.96821400** | **-0.10270600** |
| **C** | **-3.87501600** | **1.51371000** | **-0.23315100** |
| **C** | **-4.90183500** | **2.48862500** | **-0.46484700** |
| **C** | **-4.56989700** | **3.84736900** | **-0.47464800** |
| **C** | **-4.25446100** | **0.10066700** | **-0.14149000** |
| **C** | **-5.61779800** | **-0.26797000** | **-0.39433100** |
| **C** | **-6.59599300** | **0.74945500** | **-0.67941900** |
| **C** | **-6.25872100** | **2.06253700** | **-0.68996900** |
| **C** | **-3.35301000** | **-0.90988000** | **0.21101000** |
| **C** | **-3.71602800** | **-2.26156800** | **0.30915900** |
| **C** | **-5.06901000** | **-2.62090500** | **0.00208100** |
| **C** | **-5.98108400** | **-1.61447200** | **-0.33449500** |
| **C** | **-2.78311900** | **-3.30998500** | **0.71778700** |
| **C** | **-3.23548700** | **-4.66253600** | **0.75803000** |
| **C** | **-4.59615500** | **-4.97289700** | **0.41048200** |
| **C** | **-5.47332000** | **-3.99861200** | **0.05748200** |
| **H** | **3.32662400** | **-6.92264300** | **-1.38738300** |
| **H** | **5.39756200** | **-5.68594200** | **-0.84433200** |
| **H** | **6.52727200** | **-3.53097700** | **-0.30779900** |
| **H** | **2.15872400** | **-1.18817400** | **-0.57395300** |
| **H** | **7.67051700** | **-1.46618000** | **0.18634000** |
| **H** | **7.64249900** | **0.97253400** | **0.52088500** |
| **H** | **2.11861300** | **0.78382300** | **-0.12882600** |
| **H** | **6.44836500** | **3.07320200** | **0.60698000** |
| **H** | **5.30810400** | **5.24958100** | **0.67805200** |
| **H** | **3.25203400** | **6.58491800** | **0.54365500** |
| **H** | **0.89289100** | **6.77739600** | **0.25382200** |
| **H** | **-3.74187300** | **6.41260700** | **-0.37188700** |
| **H** | **-1.43624700** | **7.19049100** | **-0.04467000** |
| **H** | **-1.75130200** | **1.25136500** | **0.03371500** |
| **H** | **-5.36070100** | **4.57969800** | **-0.62495700** |
| **H** | **-7.62109700** | **0.43815500** | **-0.86440800** |
| **H** | **-7.00913100** | **2.82572900** | **-0.88125400** |
| **H** | **-2.33150800** | **-0.63231400** | **0.43791100** |
| **H** | **-7.01306800** | **-1.88995500** | **-0.54263300** |
| **H** | **-4.91124700** | **-6.01291800** | **0.44807900** |
| **H** | **-6.50332600** | **-4.24278600** | **-0.19075700** |
| **C** | **-2.34514500** | **-5.68650800** | **1.15128900** |
| **C** | **-1.04073400** | **-5.40179500** | **1.50866200** |
| **C** | **-0.59157200** | **-4.06981300** | **1.48291800** |
| **C** | **-1.44583200** | **-3.05133500** | **1.09410200** |
| **H** | **1.14399700** | **-5.72184600** | **-1.51688900** |
| **H** | **1.03167000** | **-3.29407300** | **-1.11040400** |
| **H** | **-2.70925300** | **-6.71115800** | **1.17333500** |
| **H** | **-0.36700300** | **-6.19828600** | **1.81171400** |
| **H** | **0.43054500** | **-3.83694900** | **1.76610100** |

H -1.07035500 -2.03342000 1.09464600

B 0.45146900 3.17739200 0.06787500

Nitrogene Doped Expanded [12]-helicene:

X Y Z

**-----------------------------------------------------------------**

| **C** | **-5.15321000** | **-3.19008200** | **-1.94140500** |
| --- | --- | --- | --- |
| **C** | **-4.42811000** | **-4.26796100** | **-1.49559300** |
| **C** | **-3.06438900** | **-4.11844400** | **-1.11017200** |
| **C** | **-4.55299100** | **-1.90427300** | **-2.02389000** |
| **C** | **-3.24121700** | **-1.72444600** | **-1.65885800** |
| **C** | **-2.45695300** | **-2.81876000** | **-1.19149600** |
| **C** | **-2.27912700** | **-5.19537400** | **-0.66105000** |
| **C** | **-0.94376800** | **-5.03690400** | **-0.30183200** |
| **C** | **-0.33003900** | **-3.73228900** | **-0.36921900** |
| **C** | **-1.10704400** | **-2.66609600** | **-0.81284000** |
| **C** | **-0.15771300** | **-6.17105500** | **0.11808200** |
| **C** | **1.15589900** | **-6.04723700** | **0.42438000** |
| **C** | **1.82279600** | **-4.76965500** | **0.36540700** |
| **C** | **1.08438600** | **-3.59499900** | **0.00195400** |
| **C** | **1.75667800** | **-2.36800600** | **0.00074400** |
| **C** | **3.18765500** | **-4.66580900** | **0.64695700** |
| **C** | **3.86635200** | **-3.44372300** | **0.59206400** |
| **C** | **3.12284600** | **-2.26197500** | **0.28703300** |
| **C** | **5.28726400** | **-3.36045200** | **0.81065600** |
| **C** | **5.94880900** | **-2.17981200** | **0.69914100** |
| **C** | **5.24351500** | **-0.96161400** | **0.40162600** |
| **C** | **3.81896100** | **-0.97895700** | **0.24544900** |
| **C** | **3.73948500** | **1.31949900** | **-0.05707000** |
| **C** | **5.16781500** | **1.42788900** | **-0.01362900** |
| **C** | **5.89384100** | **0.26406100** | **0.24516500** |
| **C** | **2.94736500** | **2.53522300** | **-0.22483900** |
| **C** | **3.62053400** | **3.77228100** | **-0.47249200** |
| **C** | **5.06030200** | **3.81467600** | **-0.48075100** |
| **C** | **5.79903800** | **2.70186400** | **-0.23684800** |
| **C** | **1.55243500** | **2.52308500** | **-0.12665200** |
| **C** | **0.78152700** | **3.68236000** | **-0.27616200** |
| **C** | **1.45575500** | **4.90329100** | **-0.61359300** |
| **C** | **2.85217100** | **4.92010100** | **-0.68923800** |
| **C** | **-0.67263000** | **3.70585300** | **-0.09646400** |
| **C** | **-1.38581900** | **4.91793100** | **-0.37651800** |
| **C** | **-0.66305700** | **6.09681900** | **-0.77976800** |
| **C** | **0.68996100** | **6.09651300** | **-0.86929900** |
| **C** | **-1.39752200** | **2.60574700** | **0.37427900** |
| **C** | **-2.78684600** | **2.63120200** | **0.56849500** |
| **C** | **-3.49330000** | **3.83344100** | **0.23632200** |
| **C** | **-2.77305300** | **4.94246400** | **-0.22256400** |
| **C** | **-3.53646600** | **1.49562100** | **1.10318600** |
| **C** | **-4.95202100** | **1.60545200** | **1.24421000** |
| **C** | **-5.61576100** | **2.82542100** | **0.87000100** |
| **C** | **-4.92037600** | **3.89048600** | **0.39492300** |
| **H** | **-6.19225600** | **-3.31544800** | **-2.23406800** |
| **H** | **-4.88301600** | **-5.25383200** | **-1.43254400** |
| **H** | **-2.72599200** | **-6.18614800** | **-0.60429400** |
| **H** | **-0.67456000** | **-1.67386600** | **-0.89627000** |
| **H** | **-0.64741600** | **-7.14069000** | **0.16711000** |
| **H** | **1.73918500** | **-6.91521500** | **0.72197600** |
| **H** | **1.23817200** | **-1.44922800** | **-0.24386200** |
| **H** | **3.74241700** | **-5.56722300** | **0.89954900** |
| **H** | **5.82582700** | **-4.27540700** | **1.04483100** |
| **H** | **7.02617700** | **-2.13078700** | **0.83800100** |
| **H** | **6.97875500** | **0.31146100** | **0.32076900** |

| **H** | **5.54526100** | **4.76980800** | **-0.66664700** |
| --- | --- | --- | --- |
| **H** | **6.88527600** | **2.74858500** | **-0.21913100** |
| **H** | **1.08699500** | **1.56690700** | **0.07822200** |
| **H** | **3.35515900** | **5.85842300** | **-0.91430100** |
| **H** | **-1.23128300** | **7.00049000** | **-0.98665000** |
| **H** | **1.22688600** | **7.00041200** | **-1.14645800** |
| **H** | **-0.85517500** | **1.70256000** | **0.62279300** |
| **H** | **-3.31157300** | **5.86050600** | **-0.44958400** |
| **H** | **-6.69563400** | **2.87865900** | **0.98652400** |
| **H** | **-5.43154000** | **4.81179700** | **0.12627900** |
| **C** | **-5.68960600** | **0.51672500** | **1.75966200** |
| **C** | **-5.06295100** | **-0.65603700** | **2.13719000** |
| **C** | **-3.66812500** | **-0.76784200** | **2.00819700** |
| **C** | **-2.92552500** | **0.28594500** | **1.50287700** |
| **H** | **-5.14029800** | **-1.06022500** | **-2.37429700** |
| **H** | **-2.78438400** | **-0.73983300** | **-1.71476200** |
| **H** | **-6.76796300** | **0.61941400** | **1.85786300** |
| **H** | **-5.64288500** | **-1.48601900** | **2.53093900** |
| **H** | **-3.16665300** | **-1.68605500** | **2.29978700** |
| **H** | **-1.85053000** | **0.16655300** | **1.42107800** |
| **N** | **3.10920600** | **0.14164200** | **0.05037200** |

Silicone Doped Expanded [12]-helicene:

X Y Z

**-----------------------------------------------------------------**

| **C** | **-4.24258700** | **5.93860500** | **-0.65926300** |
| --- | --- | --- | --- |
| **C** | **-5.33063000** | **5.12914500** | **-0.44058200** |
| **C** | **-5.17778500** | **3.71705900** | **-0.32357100** |
| **C** | **-2.94144000** | **5.37784400** | **-0.77583800** |
| **C** | **-2.75783500** | **4.02063100** | **-0.66625300** |
| **C** | **-3.86226500** | **3.15027700** | **-0.43324700** |
| **C** | **-6.26376900** | **2.84799200** | **-0.11290000** |
| **C** | **-6.09820500** | **1.46986100** | **-0.00425100** |
| **C** | **-4.77696200** | **0.89622900** | **-0.09442800** |
| **C** | **-3.70403600** | **1.75529600** | **-0.31151500** |
| **C** | **-7.23638000** | **0.60387400** | **0.18640200** |
| **C** | **-7.09535500** | **-0.74096600** | **0.27504300** |
| **C** | **-5.79842500** | **-1.36660800** | **0.20282000** |
| **C** | **-4.62672200** | **-0.55888400** | **0.03627500** |
| **C** | **-3.38487000** | **-1.19827000** | **0.00514700** |
| **C** | **-5.66056700** | **-2.75394900** | **0.29398600** |
| **C** | **-4.41305400** | **-3.38536800** | **0.24255700** |
| **C** | **-3.23111500** | **-2.58962300** | **0.11086900** |
| **C** | **-4.28929800** | **-4.81596700** | **0.31377100** |
| **C** | **-3.07801500** | **-5.42320200** | **0.25356200** |
| **C** | **-1.84628500** | **-4.67291300** | **0.13892000** |
| **C** | **-1.92575600** | **-3.23783900** | **0.08460700** |
| **C** | **1.03841200** | **-3.53576400** | **-0.02270400** |
| **C** | **0.67697200** | **-4.92790500** | **0.00010100** |
| **C** | **-0.64452600** | **-5.40406200** | **0.08666700** |
| **C** | **2.44624600** | **-3.15987600** | **-0.08381500** |
| **C** | **3.44623400** | **-4.18047700** | **-0.17569100** |
| **C** | **3.04164900** | **-5.56038500** | **-0.16660900** |
| **C** | **1.73552800** | **-5.91207500** | **-0.07172500** |
| **C** | **2.87263500** | **-1.82370800** | **-0.05335300** |
| **C** | **4.21680900** | **-1.44374200** | **-0.12599600** |
| **C** | **5.20284600** | **-2.47651200** | **-0.25792600** |
| **C** | **4.79165500** | **-3.81273600** | **-0.26941600** |
| **C** | **4.65726100** | **-0.04590300** | **-0.07610100** |
| **C** | **6.05454600** | **0.24443600** | **-0.21864000** |
| **C** | **6.99863500** | **-0.83253100** | **-0.37362100** |
| **C** | **6.59433300** | **-2.12705800** | **-0.38017100** |

| **C** | **3.77706600** | **1.02483800** | **0.11498600** |
| --- | --- | --- | --- |
| **C** | **4.19418900** | **2.36317100** | **0.15990600** |
| **C** | **5.58927400** | **2.64045300** | **-0.01512300** |
| **C** | **6.47866700** | **1.57448700** | **-0.19408300** |
| **C** | **3.27338900** | **3.47736900** | **0.38000800** |
| **C** | **3.78718600** | **4.80835000** | **0.38657600** |
| **C** | **5.19381100** | **5.03346100** | **0.18710000** |
| **C** | **6.05446900** | **3.99992400** | **0.00111600** |
| **H** | **-4.37203600** | **7.01383700** | **-0.74787300** |
| **H** | **-6.32787100** | **5.55450500** | **-0.35539700** |
| **H** | **-7.26604900** | **3.26548700** | **-0.03900000** |
| **H** | **-2.69639900** | **1.36164400** | **-0.40269700** |
| **H** | **-8.22058900** | **1.06163600** | **0.24882300** |
| **H** | **-7.96458600** | **-1.38032600** | **0.40888900** |
| **H** | **-2.49160200** | **-0.59497100** | **-0.10785600** |
| **H** | **-6.55396900** | **-3.36509300** | **0.40722200** |
| **H** | **-5.19712600** | **-5.40701500** | **0.40948900** |
| **H** | **-3.00773400** | **-6.50715700** | **0.29840300** |
| **H** | **-0.19995700** | **-0.94398600** | **-0.03539300** |
| **H** | **-0.75228300** | **-6.48611700** | **0.11597100** |
| **H** | **3.81400300** | **-6.32329500** | **-0.23048900** |
| **H** | **1.45217400** | **-6.96153700** | **-0.05649600** |
| **H** | **2.11674100** | **-1.05139800** | **0.02886200** |
| **H** | **5.54495100** | **-4.59361300** | **-0.35455600** |
| **H** | **8.05129200** | **-0.58035400** | **-0.47551800** |
| **H** | **7.31728300** | **-2.93225300** | **-0.48610900** |
| **H** | **2.72376700** | **0.81111700** | **0.24226700** |
| **H** | **7.53867500** | **1.79031600** | **-0.31256400** |
| **H** | **5.55576500** | **6.05875400** | **0.19602200** |
| **H** | **7.11702800** | **4.18188900** | **-0.14044600** |
| **C** | **2.91062200** | **5.89565400** | **0.59879500** |
| **C** | **1.55957200** | **5.69429100** | **0.80951700** |
| **C** | **1.04741200** | **4.38501300** | **0.81272100** |
| **C** | **1.88802700** | **3.30497000** | **0.59978700** |
| **H** | **-2.09059000** | **6.02909200** | **-0.95660700** |
| **H** | **-1.76331800** | **3.59102300** | **-0.76293500** |
| **H** | **3.32205200** | **6.90241800** | **0.59875200** |
| **H** | **0.89819900** | **6.53974000** | **0.97785100** |
| **H** | **-0.01203500** | **4.21863900** | **0.98806500** |
| **H** | **1.46188100** | **2.30742200** | **0.61387400** |
| **Si** | **-0.34621800** | **-2.41156000** | **0.00592500** |

Phosphore Doped Expanded [12]-helicene:

X Y Z

**-----------------------------------------------------------------**

| **C** | **-3.79007800** | **5.93010300** | **-0.95460000** |
| --- | --- | --- | --- |
| **C** | **-4.90753100** | **5.17660200** | **-0.68872900** |
| **C** | **-4.80958500** | **3.76678900** | **-0.50605100** |
| **C** | **-2.51375100** | **5.31234800** | **-1.05393000** |
| **C** | **-2.38366700** | **3.95485400** | **-0.88471300** |
| **C** | **-3.51969800** | **3.14174600** | **-0.60309700** |
| **C** | **-5.92636600** | **2.95420400** | **-0.23917000** |
| **C** | **-5.81384800** | **1.57768300** | **-0.06592100** |
| **C** | **-4.51901700** | **0.94546900** | **-0.14866900** |
| **C** | **-3.41505400** | **1.74802600** | **-0.41859700** |
| **C** | **-6.98348200** | **0.77143900** | **0.18594500** |
| **C** | **-6.89835700** | **-0.57210700** | **0.33744900** |
| **C** | **-5.63017300** | **-1.25471800** | **0.26930200** |
| **C** | **-4.42794700** | **-0.50813700** | **0.04417700** |
| **C** | **-3.21542200** | **-1.20161400** | **0.01335300** |
| **C** | **-5.55132300** | **-2.64061500** | **0.41541900** |
| **C** | **-4.33454600** | **-3.32959700** | **0.36060600** |

| **C** | **-3.12033100** | **-2.59449400** | **0.17228400** |
| --- | --- | --- | --- |
| **C** | **-4.28844600** | **-4.76095600** | **0.47783800** |
| **C** | **-3.11274800** | **-5.42985600** | **0.39824400** |
| **C** | **-1.85736400** | **-4.73716300** | **0.22468000** |
| **C** | **-1.84101100** | **-3.30950000** | **0.13952100** |
| **C** | **0.91207300** | **-3.61853900** | **-0.03106100** |
| **C** | **0.61744300** | **-5.01803600** | **-0.00323500** |
| **C** | **-0.68819400** | **-5.50221100** | **0.13781800** |
| **C** | **2.31552400** | **-3.20273600** | **-0.12516800** |
| **C** | **3.33644000** | **-4.19740100** | **-0.27019200** |
| **C** | **2.97937400** | **-5.58952700** | **-0.26691600** |
| **C** | **1.68981900** | **-5.97860700** | **-0.12268200** |
| **C** | **2.71375500** | **-1.85711500** | **-0.07448800** |
| **C** | **4.04682400** | **-1.44703400** | **-0.18143700** |
| **C** | **5.04988400** | **-2.45278800** | **-0.37872400** |
| **C** | **4.66889900** | **-3.79626000** | **-0.40601200** |
| **C** | **4.46059100** | **-0.04193100** | **-0.10222700** |
| **C** | **5.84460600** | **0.28232400** | **-0.29330800** |
| **C** | **6.80361000** | **-0.76826000** | **-0.52062700** |
| **C** | **6.42800800** | **-2.07071500** | **-0.54711800** |
| **C** | **3.56912200** | **1.00242700** | **0.16586700** |
| **C** | **3.96423600** | **2.34657100** | **0.24230000** |
| **C** | **5.34401800** | **2.65902900** | **0.01367200** |
| **C** | **6.24458000** | **1.61864200** | **-0.24220100** |
| **C** | **3.03520400** | **3.43300800** | **0.54836100** |
| **C** | **3.52366200** | **4.77325800** | **0.57867300** |
| **C** | **4.91414200** | **5.03449100** | **0.32001900** |
| **C** | **5.78428500** | **4.02606500** | **0.05681100** |
| **H** | **-3.87722500** | **7.00447500** | **-1.09235000** |
| **H** | **-5.88599600** | **5.64542500** | **-0.61433000** |
| **H** | **-6.90982000** | **3.41523800** | **-0.17127300** |
| **H** | **-2.42565600** | **1.30916800** | **-0.50232800** |
| **H** | **-7.94601500** | **1.27390200** | **0.24337600** |
| **H** | **-7.79059400** | **-1.16677700** | **0.51743400** |
| **H** | **-2.29829500** | **-0.64764800** | **-0.14634500** |
| **H** | **-6.46755000** | **-3.20675000** | **0.57107900** |
| **H** | **-5.22338500** | **-5.29779600** | **0.61873500** |
| **H** | **-3.08884100** | **-6.51432300** | **0.47117300** |
| **H** | **-0.80692600** | **-6.58331000** | **0.18086600** |
| **H** | **3.77255400** | **-6.32584800** | **-0.37106500** |
| **H** | **1.43042500** | **-7.03424500** | **-0.10563500** |
| **H** | **1.94234700** | **-1.10719000** | **0.05243400** |
| **H** | **5.43530100** | **-4.55789500** | **-0.53538100** |
| **H** | **7.84543400** | **-0.48938400** | **-0.65848700** |
| **H** | **7.16249700** | **-2.85666600** | **-0.70481900** |
| **H** | **2.52693800** | **0.76192100** | **0.33125600** |
| **H** | **7.29405800** | **1.85979700** | **-0.39925500** |
| **H** | **5.25659500** | **6.06612600** | **0.34868700** |
| **H** | **6.83535600** | **4.23427100** | **-0.12781300** |
| **C** | **2.63859200** | **5.83421900** | **0.87317300** |
| **C** | **1.30395800** | **5.59727100** | **1.14280300** |
| **C** | **0.81788100** | **4.27803800** | **1.12491300** |
| **C** | **1.66647400** | **3.22343100** | **0.83101200** |
| **H** | **-1.63769200** | **5.91908000** | **-1.26592900** |
| **H** | **-1.40728400** | **3.48315100** | **-0.96315900** |
| **H** | **3.03065900** | **6.84859100** | **0.88951700** |
| **H** | **0.63487600** | **6.42169000** | **1.37337500** |
| **H** | **-0.22839900** | **4.08400700** | **1.34205500** |
| **H** | **1.26050300** | **2.21742000** | **0.83179800** |
| **P** | **-0.34561700** | **-2.38701000** | **0.01427900** |

Expanded [13]-helicene:

X Y Z

**-----------------------------------------------------------------**

| **C** | **-5.08294500** | **3.67069400** | **1.35390200** |
| --- | --- | --- | --- |
| **C** | **-4.31165100** | **4.66603600** | **0.84602700** |
| **C** | **-2.91403800** | **4.46000000** | **0.58325300** |
| **C** | **-4.53259700** | **2.37573100** | **1.65202900** |
| **C** | **-3.15146700** | **2.11748100** | **1.40530700** |
| **C** | **-2.31956500** | **3.18293200** | **0.84827400** |
| **C** | **-2.11300700** | **5.49362600** | **0.08447400** |
| **C** | **-0.75009400** | **5.32670200** | **-0.16774600** |
| **C** | **-0.14769300** | **4.04526500** | **0.06235300** |
| **C** | **-0.95464600** | **3.01939900** | **0.56733000** |
| **C** | **0.05704800** | **6.42711300** | **-0.62846600** |
| **C** | **1.39111100** | **6.28629600** | **-0.82472500** |
| **C** | **2.04682200** | **5.02106300** | **-0.61592600** |
| **C** | **1.28304500** | **3.87783700** | **-0.21179200** |
| **C** | **1.94928900** | **2.65159900** | **-0.08766000** |
| **C** | **3.42713800** | **4.89032200** | **-0.79158100** |
| **C** | **4.09112700** | **3.67337000** | **-0.61257200** |
| **C** | **3.32771000** | **2.50411200** | **-0.29211100** |
| **C** | **5.52423800** | **3.58749500** | **-0.72046800** |
| **C** | **6.17593000** | **2.41813500** | **-0.50198000** |
| **C** | **5.45494400** | **1.20465200** | **-0.21830100** |
| **C** | **4.02289200** | **1.22092900** | **-0.16467600** |
| **C** | **3.35534000** | **0.00007100** | **0.00000000** |
| **C** | **4.02294500** | **-1.22075700** | **0.16467700** |
| **C** | **5.45499600** | **-1.20441700** | **0.21830400** |
| **C** | **6.12962000** | **0.00013100** | **0.00000200** |
| **C** | **3.32781800** | **-2.50397100** | **0.29211100** |
| **C** | **4.09128500** | **-3.67319600** | **0.61257200** |
| **C** | **5.52439200** | **-3.58725800** | **0.72047000** |
| **C** | **6.17603500** | **-2.41786900** | **0.50198300** |
| **C** | **1.94940500** | **-2.65151600** | **0.08765900** |
| **C** | **1.28321300** | **-3.87778300** | **0.21179000** |
| **C** | **2.04704000** | **-5.02097700** | **0.61592300** |
| **C** | **3.42734900** | **-4.89017500** | **0.79157800** |
| **C** | **-0.14751900** | **-4.04527100** | **-0.06235600** |
| **C** | **-0.74986400** | **-5.32673600** | **0.16773900** |
| **C** | **0.05732500** | **-6.42711300** | **0.62845900** |
| **C** | **1.39138200** | **-6.28623700** | **0.82471900** |
| **C** | **-0.95451500** | **-3.01943900** | **-0.56733100** |
| **C** | **-2.31942800** | **-3.18303200** | **-0.84827500** |
| **C** | **-2.91384500** | **-4.46012600** | **-0.58325900** |
| **C** | **-2.11276800** | **-5.49371800** | **-0.08448200** |
| **C** | **-3.15137700** | **-2.11761500** | **-1.40530300** |
| **C** | **-4.53249400** | **-2.37592400** | **-1.65202900** |
| **C** | **-5.08278500** | **-3.67091500** | **-1.35391000** |
| **C** | **-4.31144800** | **-4.66622500** | **-0.84603800** |
| **H** | **-6.13935800** | **3.83689600** | **1.55104400** |
| **H** | **-4.73718600** | **5.64318900** | **0.63073100** |
| **H** | **-2.56582500** | **6.46612500** | **-0.09868100** |
| **H** | **-0.49975900** | **2.05901700** | **0.77312700** |
| **H** | **-0.42978700** | **7.38480900** | **-0.79572800** |
| **H** | **1.99580300** | **7.12926200** | **-1.15010800** |
| **H** | **1.37609600** | **1.77854500** | **0.19793300** |
| **H** | **4.00720700** | **5.76989400** | **-1.06381900** |
| **H** | **6.07610400** | **4.49407200** | **-0.95638600** |
| **H** | **7.26076500** | **2.36953700** | **-0.55545600** |
| **H** | **2.27241300** | **0.00004800** | **-0.00000100** |
| **H** | **7.21784300** | **0.00015500** | **0.00000200** |
| **H** | **6.07629700** | **-4.49381200** | **0.95638700** |
| **H** | **7.26086700** | **-2.36922600** | **0.55546000** |
| **H** | **1.37617300** | **-1.77848700** | **-0.19793400** |

| **H** | **4.00745700** | **-5.76972400** | **1.06381600** |
| --- | --- | --- | --- |
| **H** | **-0.42946700** | **-7.38482900** | **0.79571900** |
| **H** | **1.99611100** | **-7.12917800** | **1.15010100** |
| **H** | **-0.49967200** | **-2.05903700** | **-0.77312500** |
| **H** | **-2.56554500** | **-6.46623700** | **0.09866900** |
| **H** | **-6.13919000** | **-3.83716200** | **-1.55105500** |
| **H** | **-4.73694000** | **-5.64339700** | **-0.63074600** |
| **C** | **-5.34886800** | **-1.35738900** | **-2.19155900** |
| **C** | **-4.83303400** | **-0.10969700** | **-2.48715000** |
| **C** | **-3.47280600** | **0.15094700** | **-2.24849900** |
| **C** | **-2.65467700** | **-0.83334400** | **-1.72076700** |
| **H** | **-6.39931600** | **-1.57409900** | **-2.37192100** |
| **H** | **-5.47298900** | **0.66524100** | **-2.89969300** |
| **H** | **-3.05911600** | **1.12987500** | **-2.47330500** |
| **H** | **-1.60930300** | **-0.59867600** | **-1.55163900** |
| **C** | **-2.65471000** | **0.83323400** | **1.72078100** |
| **C** | **-3.47279700** | **-0.15109000** | **2.24851700** |
| **C** | **-4.83303600** | **0.10949500** | **2.48716300** |
| **C** | **-5.34892500** | **1.35716300** | **2.19156300** |
| **H** | **-6.39938400** | **1.57382500** | **2.37192000** |
| **H** | **-5.47295700** | **-0.66547000** | **2.89970900** |
| **H** | **-3.05906200** | **-1.12999800** | **2.47333100** |
| **H** | **-1.60932500** | **0.59861300** | **1.55165700** |

Hydroxyl Functionalized Expanded [13]-helicene:

X Y Z

**-----------------------------------------------------------------**

| **C** | **3.72578700** | **-4.88568500** | **-1.50718800** |
| --- | --- | --- | --- |
| **C** | **2.68948800** | **-5.63392300** | **-1.05871000** |
| **C** | **1.43775700** | **-5.02412100** | **-0.71381100** |
| **C** | **3.62577500** | **-3.45643300** | **-1.63088800** |
| **C** | **2.40917000** | **-2.78999000** | **-1.27168900** |
| **C** | **1.27070600** | **-3.59936100** | **-0.82170400** |
| **C** | **0.36618100** | **-5.81867400** | **-0.29292700** |
| **C** | **-0.88691700** | **-5.28569900** | **0.01080700** |
| **C** | **-1.07777900** | **-3.87208000** | **-0.10238500** |
| **C** | **0.00300500** | **-3.07721700** | **-0.50721900** |
| **C** | **-1.98238900** | **-6.13244300** | **0.40745500** |
| **C** | **-3.21439600** | **-5.62028800** | **0.65112200** |
| **C** | **-3.47072500** | **-4.20765300** | **0.53552300** |
| **C** | **-2.40387400** | **-3.31692100** | **0.18443800** |
| **C** | **-2.68728600** | **-1.94806200** | **0.11120600** |
| **C** | **-4.75330200** | **-3.68918000** | **0.74058700** |
| **C** | **-5.03442400** | **-2.32445500** | **0.62357800** |
| **C** | **-3.96522800** | **-1.41884600** | **0.32905700** |
| **C** | **-6.37719900** | **-1.82226200** | **0.76200700** |
| **C** | **-6.65957400** | **-0.50597100** | **0.58913500** |
| **C** | **-5.61890100** | **0.45063000** | **0.31311200** |
| **C** | **-4.25738100** | **0.01181100** | **0.23302100** |
| **C** | **-3.26076700** | **0.97894900** | **0.06019700** |
| **C** | **-3.53881400** | **2.34149900** | **-0.09339500** |
| **C** | **-4.90971200** | **2.75528200** | **-0.11803600** |
| **C** | **-5.90897500** | **1.80484200** | **0.11525800** |
| **C** | **-2.49141700** | **3.35011500** | **-0.25277600** |
| **C** | **-2.86703700** | **4.68945500** | **-0.59283600** |
| **C** | **-4.26178400** | **5.04240800** | **-0.66245500** |
| **C** | **-5.23241700** | **4.12981500** | **-0.40238600** |
| **C** | **-1.13169500** | **3.06919100** | **-0.06933300** |
| **C** | **-0.12415100** | **4.02389900** | **-0.24931000** |
| **C** | **-0.50546500** | **5.33015600** | **-0.69822000** |
| **C** | **-1.86372200** | **5.63303700** | **-0.83551700** |
| **C** | **1.29223400** | **3.74758000** | **0.00555000** |
| **C** | **2.26061400** | **4.75173900** | **-0.32584700** |
| **C** | **1.82641600** | **6.02286800** | **-0.84704800** |

| **C** | **0.50909000** | **6.30766400** | **-0.99775400** |
| --- | --- | --- | --- |
| **C** | **1.74598600** | **2.56114300** | **0.59838300** |
| **C** | **3.10114500** | **2.30801600** | **0.87097000** |
| **C** | **4.06129100** | **3.30161900** | **0.48663700** |
| **C** | **3.61351600** | **4.49224200** | **-0.09753700** |
| **C** | **3.56856600** | **1.09496300** | **1.54174100** |
| **C** | **4.96665000** | **0.92830900** | **1.77213300** |
| **C** | **5.88972400** | **1.94502200** | **1.34463200** |
| **C** | **5.45938300** | **3.07802500** | **0.73280500** |
| **H** | **4.66827800** | **-5.35301000** | **-1.78121000** |
| **H** | **2.78090100** | **-6.71331300** | **-0.96468700** |
| **H** | **0.51337000** | **-6.89441700** | **-0.21842600** |
| **H** | **-0.13963100** | **-2.01502000** | **-0.60516600** |
| **H** | **-1.79985700** | **-7.20100600** | **0.49316600** |
| **H** | **-4.03884100** | **-6.26994100** | **0.93493300** |
| **H** | **-1.88535200** | **-1.27146000** | **-0.15315400** |
| **H** | **-5.56412100** | **-4.37430600** | **0.98062200** |
| **H** | **-7.16980100** | **-2.53275700** | **0.98410100** |
| **H** | **-7.68199000** | **-0.14430500** | **0.66690900** |
| **H** | **-2.22823100** | **0.65609600** | **0.02989200** |
| **H** | **-6.94802600** | **2.12789400** | **0.13382700** |
| **H** | **-4.51944000** | **6.07021300** | **-0.90600700** |
| **H** | **-6.28115800** | **4.41550300** | **-0.42988000** |
| **H** | **-0.84977000** | **2.06935300** | **0.23607700** |
| **H** | **-2.14951400** | **6.63960100** | **-1.13449000** |
| **H** | **2.58481000** | **6.76334400** | **-1.08899700** |
| **H** | **0.19120300** | **7.28200100** | **-1.36065900** |
| **H** | **1.01208600** | **1.83034900** | **0.91607700** |
| **H** | **4.34544700** | **5.25363500** | **-0.36005300** |
| **H** | **6.94930000** | **1.78665600** | **1.53055700** |
| **H** | **6.16540500** | **3.84463400** | **0.42302800** |
| **C** | **5.43433000** | **-0.23499500** | **2.42209300** |
| **C** | **4.55922900** | **-1.21930300** | **2.84084100** |
| **C** | **3.18096300** | **-1.06130300** | **2.61652900** |
| **C** | **2.69970400** | **0.07220000** | **1.98376200** |
| **H** | **6.50364200** | **-0.34377200** | **2.58780900** |
| **H** | **4.93271700** | **-2.11065600** | **3.33700400** |
| **H** | **2.48611400** | **-1.83239500** | **2.93655100** |
| **H** | **1.63149300** | **0.15395100** | **1.81890900** |
| **C** | **2.42153800** | **-1.36817200** | **-1.38554500** |
| **C** | **3.53957500** | **-0.68369500** | **-1.85122200** |
| **C** | **4.70492300** | **-1.36551200** | **-2.21297600** |
| **C** | **4.74750200** | **-2.74015500** | **-2.09816700** |
| **H** | **5.64584100** | **-3.29131700** | **-2.36226400** |
| **H** | **5.56590800** | **-0.80905900** | **-2.57187100** |
| **H** | **3.50068600** | **0.40159700** | **-1.91874200** |
| **O** | **1.31243200** | **-0.64476400** | **-1.02136600** |
| **H** | **1.52958800** | **0.29954500** | **-1.09710500** |

Methyl Functionalized Expanded [13]-helicene:

X Y Z

**-----------------------------------------------------------------**

| **C** | **5.56078100** | **-2.83359200** | **-0.95241600** |
| --- | --- | --- | --- |
| **C** | **4.91151100** | **-3.95043700** | **-0.54771300** |
| **C** | **3.48169300** | **-3.96116500** | **-0.44212800** |
| **C** | **4.84681500** | **-1.65673300** | **-1.36845200** |
| **C** | **3.41217600** | **-1.62317000** | **-1.35588100** |
| **C** | **2.71730200** | **-2.78909000** | **-0.78295500** |
| **C** | **2.82956600** | **-5.10606600** | **0.02714600** |
| **C** | **1.44971400** | **-5.15786400** | **0.22384100** |
| **C** | **0.67993600** | **-3.97567200** | **-0.01335700** |
| **C** | **1.33885400** | **-2.84001600** | **-0.50430300** |

| **C** | **0.80257900** | **-6.36795700** | **0.65904200** |
| --- | --- | --- | --- |
| **C** | **-0.54108900** | **-6.41877500** | **0.83241600** |
| **C** | **-1.36435200** | **-5.25567300** | **0.62410800** |
| **C** | **-0.76426200** | **-4.01180700** | **0.23947500** |
| **C** | **-1.59774600** | **-2.89216500** | **0.11146600** |
| **C** | **-2.75140600** | **-5.32365800** | **0.78065700** |
| **C** | **-3.57901200** | **-4.21178100** | **0.60273300** |
| **C** | **-2.98554400** | **-2.94318900** | **0.29998400** |
| **C** | **-5.01067400** | **-4.33213400** | **0.69465300** |
| **C** | **-5.81987200** | **-3.26510500** | **0.48039200** |
| **C** | **-5.27562900** | **-1.95864200** | **0.21656700** |
| **C** | **-3.85532200** | **-1.77041200** | **0.17552800** |
| **C** | **-3.36765700** | **-0.46495900** | **0.02813500** |
| **C** | **-4.20135400** | **0.65073800** | **-0.12797100** |
| **C** | **-5.61630900** | **0.43142700** | **-0.18975100** |
| **C** | **-6.11345100** | **-0.85976800** | **0.00762800** |
| **C** | **-3.69627400** | **2.02195700** | **-0.23548400** |
| **C** | **-4.61954400** | **3.07589200** | **-0.53529500** |
| **C** | **-6.02542400** | **2.78815600** | **-0.65049900** |
| **C** | **-6.50305200** | **1.53358700** | **-0.45724400** |
| **C** | **-2.35210300** | **2.36138900** | **-0.03132000** |
| **C** | **-1.86806200** | **3.67231400** | **-0.13338500** |
| **C** | **-2.79007800** | **4.70311600** | **-0.50864900** |
| **C** | **-4.13765700** | **4.37908100** | **-0.68748700** |
| **C** | **-0.47301400** | **4.03607900** | **0.13483000** |
| **C** | **-0.06337400** | **5.39687900** | **-0.06115900** |
| **C** | **-1.02426700** | **6.38169100** | **-0.48707300** |
| **C** | **-2.32492200** | **6.05468100** | **-0.68539000** |
| **C** | **0.47987500** | **3.12215300** | **0.59840800** |
| **C** | **1.81159100** | **3.47080300** | **0.86887700** |
| **C** | **2.21216700** | **4.82834800** | **0.64365100** |
| **C** | **1.26412100** | **5.75096600** | **0.18668100** |
| **C** | **2.79771000** | **2.51654300** | **1.37289000** |
| **C** | **4.13143600** | **2.96193300** | **1.61297900** |
| **C** | **4.48399100** | **4.33345100** | **1.36123900** |
| **C** | **3.56931700** | **5.22476900** | **0.90021100** |
| **H** | **6.64671000** | **-2.81104900** | **-1.00145000** |
| **H** | **5.45829100** | **-4.84758800** | **-0.26827000** |
| **H** | **3.42620300** | **-5.98810000** | **0.25151700** |
| **H** | **0.74618000** | **-1.95915700** | **-0.66869900** |
| **H** | **1.41850400** | **-7.24772700** | **0.82879300** |
| **H** | **-1.02669300** | **-7.34166800** | **1.13999800** |
| **H** | **-1.15255000** | **-1.94455600** | **-0.16491600** |
| **H** | **-3.20409800** | **-6.27930400** | **1.03772200** |
| **H** | **-5.42986500** | **-5.31055900** | **0.91618500** |
| **H** | **-6.90107400** | **-3.37124500** | **0.52294200** |
| **H** | **-2.29573900** | **-0.31109600** | **0.03758600** |
| **H** | **-7.19058200** | **-1.01457500** | **0.00026200** |
| **H** | **-6.70093000** | **3.61079900** | **-0.87197400** |
| **H** | **-7.56953100** | **1.33076400** | **-0.51707600** |
| **H** | **-1.65883700** | **1.57408600** | **0.23651700** |
| **H** | **-4.83892100** | **5.17179300** | **-0.94065700** |
| **H** | **-0.68185000** | **7.40387500** | **-0.62875700** |
| **H** | **-3.04683300** | **6.80963300** | **-0.98709000** |
| **H** | **0.17106100** | **2.10003900** | **0.77550200** |
| **H** | **1.57003200** | **6.78372100** | **0.03114000** |
| **H** | **5.50845300** | **4.64338900** | **1.55348800** |
| **H** | **3.84624400** | **6.26031400** | **0.71791600** |
| **C** | **5.09594600** | **2.05125000** | **2.09815900** |
| **C** | **4.77140800** | **0.73068900** | **2.34490000** |
| **C** | **3.45864000** | **0.28565100** | **2.11296900** |
| **C** | **2.49714500** | **1.16232800** | **1.64011700** |
| **H** | **6.10734100** | **2.41036900** | **2.27454700** |

| **H** | **5.52419300** | **0.03990900** | **2.71411900** |
| --- | --- | --- | --- |
| **H** | **3.19497600** | **-0.75147200** | **2.29902200** |
| **H** | **1.49285000** | **0.78629700** | **1.47682800** |
| **C** | **2.78176700** | **-0.46955900** | **-1.92885800** |
| **C** | **3.57259500** | **0.58886700** | **-2.37440900** |
| **C** | **4.96975100** | **0.57238100** | **-2.30843300** |
| **C** | **5.59817000** | **-0.55500300** | **-1.82598700** |
| **H** | **6.68321100** | **-0.61967400** | **-1.80168900** |
| **H** | **5.54370100** | **1.42521400** | **-2.65965500** |
| **H** | **3.07694600** | **1.45705800** | **-2.80078300** |
| **C** | **1.29243700** | **-0.30474700** | **-2.15622000** |
| **H** | **0.83412800** | **-1.19791000** | **-2.59275800** |
| **H** | **0.74757300** | **-0.06632900** | **-1.23316400** |
| **H** | **1.11563700** | **0.52686800** | **-2.84434000** |

Methoxy Functionalized Expanded [13]-helicene:

X Y Z

**-----------------------------------------------------------------**

| **C** | **3.49429000** | **-5.11105300** | **-1.63687800** |
| --- | --- | --- | --- |
| **C** | **2.42824900** | **-5.81604100** | **-1.17841500** |
| **C** | **1.19627400** | **-5.15871900** | **-0.83917900** |
| **C** | **3.43940300** | **-3.68127000** | **-1.78350300** |
| **C** | **2.24546500** | **-2.97819000** | **-1.44410500** |
| **C** | **1.08654700** | **-3.73618700** | **-0.97536600** |
| **C** | **0.08786700** | **-5.88685400** | **-0.39192700** |
| **C** | **-1.13092500** | **-5.27941000** | **-0.08381800** |
| **C** | **-1.25866400** | **-3.85691800** | **-0.22021400** |
| **C** | **-0.14286500** | **-3.13570600** | **-0.66223600** |
| **C** | **-2.26015900** | **-6.06444800** | **0.34404200** |
| **C** | **-3.46061700** | **-5.48826800** | **0.59836000** |
| **C** | **-3.64913100** | **-4.06650600** | **0.46660900** |
| **C** | **-2.54961400** | **-3.23063000** | **0.08407800** |
| **C** | **-2.77021300** | **-1.84890900** | **0.00376800** |
| **C** | **-4.90100500** | **-3.48627300** | **0.68837500** |
| **C** | **-5.12125200** | **-2.11171700** | **0.56240500** |
| **C** | **-4.01767300** | **-1.25580100** | **0.24232300** |
| **C** | **-6.44011700** | **-1.55560500** | **0.71798600** |
| **C** | **-6.66867000** | **-0.23090400** | **0.53745100** |
| **C** | **-5.59235200** | **0.67833500** | **0.24164200** |
| **C** | **-4.24918100** | **0.18773800** | **0.14516600** |
| **C** | **-3.21914500** | **1.11958700** | **-0.03912900** |
| **C** | **-3.44851400** | **2.49399600** | **-0.18610400** |
| **C** | **-4.80542200** | **2.95473700** | **-0.19845500** |
| **C** | **-5.83475800** | **2.04086200** | **0.04443100** |
| **C** | **-2.37020200** | **3.47222300** | **-0.34437500** |
| **C** | **-2.70960600** | **4.82566400** | **-0.66910400** |
| **C** | **-4.09235500** | **5.22196200** | **-0.73280600** |
| **C** | **-5.08946700** | **4.33905100** | **-0.47461800** |
| **C** | **-1.01741800** | **3.15360400** | **-0.16871700** |
| **C** | **0.01510400** | **4.08550000** | **-0.33590500** |
| **C** | **-0.33350300** | **5.40862900** | **-0.76245000** |
| **C** | **-1.68313000** | **5.74663500** | **-0.89798700** |
| **C** | **1.42543200** | **3.77084200** | **-0.08933800** |
| **C** | **2.41368100** | **4.75812400** | **-0.39859500** |
| **C** | **2.01330300** | **6.05169400** | **-0.88869400** |
| **C** | **0.70341900** | **6.36967400** | **-1.03840900** |
| **C** | **1.83962700** | **2.55483800** | **0.46854200** |
| **C** | **3.18707000** | **2.23869200** | **0.72933100** |
| **C** | **4.16844800** | **3.22290200** | **0.35257000** |
| **C** | **3.75657500** | **4.44574500** | **-0.18864300** |
| **C** | **3.64619900** | **0.99175700** | **1.35627900** |
| **C** | **5.05396400** | **0.78923100** | **1.51726100** |

| **C** | **5.98480700** | **1.79691000** | **1.08714900** |
| --- | --- | --- | --- |
| **C** | **5.56577600** | **2.96297500** | **0.54059500** |
| **H** | **4.41959600** | **-5.61836900** | **-1.89947200** |
| **H** | **2.48300100** | **-6.89627400** | **-1.06738600** |
| **H** | **0.17518400** | **-6.96722700** | **-0.29420800** |
| **H** | **-0.23752600** | **-2.06567700** | **-0.79497700** |
| **H** | **-2.12859800** | **-7.13907500** | **0.44420700** |
| **H** | **-4.31147500** | **-6.09160000** | **0.90520000** |
| **H** | **-1.94532300** | **-1.20969400** | **-0.28491400** |
| **H** | **-5.73848600** | **-4.13006100** | **0.94997300** |
| **H** | **-7.25704300** | **-2.23185900** | **0.95754300** |
| **H** | **-7.67326200** | **0.17538400** | **0.62615700** |
| **H** | **-2.19773400** | **0.76197000** | **-0.08117600** |
| **H** | **-6.86113600** | **2.40127800** | **0.07488100** |
| **H** | **-4.31818700** | **6.25920000** | **-0.96812100** |
| **H** | **-6.12949300** | **4.65533400** | **-0.49478600** |
| **H** | **-0.75687000** | **2.14409000** | **0.12318000** |
| **H** | **-1.94396100** | **6.76457800** | **-1.18108100** |
| **H** | **2.78978800** | **6.77935400** | **-1.11231600** |
| **H** | **0.40809800** | **7.35832900** | **-1.38135600** |
| **H** | **1.09324000** | **1.83116400** | **0.74321000** |
| **H** | **4.51512300** | **5.18459900** | **-0.43987900** |
| **H** | **7.04468300** | **1.59963600** | **1.22685700** |
| **H** | **6.27888400** | **3.72425800** | **0.23374600** |
| **C** | **5.56635600** | **-0.38489300** | **2.11011100** |
| **C** | **4.71496100** | **-1.36945900** | **2.56007100** |
| **C** | **3.33040200** | **-1.20186000** | **2.43218500** |
| **C** | **2.79936700** | **-0.05533700** | **1.84827900** |
| **H** | **6.64309600** | **-0.49335500** | **2.20746100** |
| **H** | **5.10424700** | **-2.27595200** | **3.01494900** |
| **C** | **2.24748900** | **-1.57155200** | **-1.57531700** |
| **C** | **3.36247900** | **-0.88882300** | **-2.03110100** |
| **C** | **4.53025900** | **-1.58910600** | **-2.37921300** |
| **C** | **4.56284000** | **-2.96502600** | **-2.25227900** |
| **H** | **5.46279900** | **-3.51930300** | **-2.50884200** |
| **H** | **5.40315200** | **-1.05009600** | **-2.73663400** |
| **H** | **1.37085000** | **-0.99666300** | **-1.29768300** |
| **H** | **2.67197900** | **-1.98470200** | **2.78616100** |
| **O** | **1.44598200** | **0.11010300** | **1.72936100** |
| **C** | **0.57417700** | **-0.82495700** | **2.35387300** |
| **H** | **-0.43432300** | **-0.43433200** | **2.20466800** |
| **H** | **0.64745200** | **-1.81701400** | **1.89309200** |
| **H** | **0.77781800** | **-0.90170000** | **3.42887300** |
| **H** | **3.33332900** | **0.19406100** | **-2.10821400** |

Amino Functionalized Expanded [13]-helicene:

X Y Z

**-----------------------------------------------------------------**

| **C** | **3.34170200** | **-5.21398100** | **-1.14640900** |
| --- | --- | --- | --- |
| **C** | **2.20574900** | **-5.88469900** | **-0.83680600** |
| **C** | **0.96994200** | **-5.18105100** | **-0.64608400** |
| **C** | **3.34359500** | **-3.79344900** | **-1.37553500** |
| **C** | **2.12142600** | **-3.05144800** | **-1.28687200** |
| **C** | **0.92045200** | **-3.75307600** | **-0.81892400** |
| **C** | **-0.17900400** | **-5.87025200** | **-0.24477100** |
| **C** | **-1.38676800** | **-5.22394800** | **0.02695100** |
| **C** | **-1.43785100** | **-3.79784100** | **-0.06995800** |
| **C** | **-0.28397100** | **-3.11304700** | **-0.47367600** |
| **C** | **-2.56083000** | **-5.96150500** | **0.41512200** |
| **C** | **-3.73300800** | **-5.32948600** | **0.67347600** |
| **C** | **-3.84626100** | **-3.89703100** | **0.57500800** |
| **C** | **-2.69879500** | **-3.11297400** | **0.22568600** |

| **C** | **-2.84790700** | **-1.72196200** | **0.14948200** |
| --- | --- | --- | --- |
| **C** | **-5.07014300** | **-3.25502700** | **0.78644000** |
| **C** | **-5.21735400** | **-1.86980800** | **0.66948200** |
| **C** | **-4.06761100** | **-1.06958200** | **0.36752300** |
| **C** | **-6.50749100** | **-1.24609200** | **0.80967600** |
| **C** | **-6.66608900** | **0.08851800** | **0.62613300** |
| **C** | **-5.54193700** | **0.94096600** | **0.33788800** |
| **C** | **-4.22456000** | **0.38282200** | **0.25886300** |
| **C** | **-3.14596800** | **1.25831500** | **0.07392500** |
| **C** | **-3.30321700** | **2.64073700** | **-0.09083300** |
| **C** | **-4.63508600** | **3.17004100** | **-0.11258500** |
| **C** | **-5.71202700** | **2.31253700** | **0.12958200** |
| **C** | **-2.17540700** | **3.56209300** | **-0.25284500** |
| **C** | **-2.44661000** | **4.93251000** | **-0.57120700** |
| **C** | **-3.80698700** | **5.39820800** | **-0.64383700** |
| **C** | **-4.84818800** | **4.56566500** | **-0.39427300** |
| **C** | **-0.83860800** | **3.17542400** | **-0.08283600** |
| **C** | **0.23908700** | **4.05898100** | **-0.23289500** |
| **C** | **-0.04533400** | **5.40556300** | **-0.63333200** |
| **C** | **-1.37595200** | **5.80599100** | **-0.78076500** |
| **C** | **1.63266700** | **3.67843100** | **0.02300000** |
| **C** | **2.66621000** | **4.64654800** | **-0.20819200** |
| **C** | **2.32480800** | **5.96630100** | **-0.67306800** |
| **C** | **1.03324300** | **6.33242300** | **-0.86080900** |
| **C** | **2.00457700** | **2.42418500** | **0.52181700** |
| **C** | **3.33308300** | **2.07758900** | **0.81463000** |
| **C** | **4.35557300** | **3.05023600** | **0.56280800** |
| **C** | **3.99360200** | **4.30197800** | **0.05242000** |
| **C** | **3.71210600** | **0.78445200** | **1.38224100** |
| **C** | **5.08614800** | **0.53224600** | **1.67151700** |
| **C** | **6.07384700** | **1.53868200** | **1.38865600** |
| **C** | **5.72729700** | **2.73875000** | **0.85644100** |
| **H** | **4.28504400** | **-5.74418400** | **-1.25154000** |
| **H** | **2.21475600** | **-6.96244500** | **-0.69331000** |
| **H** | **-0.12358300** | **-6.95105500** | **-0.12937800** |
| **H** | **-0.30287800** | **-2.03526300** | **-0.47686300** |
| **H** | **-2.48667600** | **-7.04382000** | **0.48902800** |
| **H** | **-4.61775400** | **-5.89556900** | **0.95454200** |
| **H** | **-1.98712600** | **-1.12443100** | **-0.12237700** |
| **H** | **-5.94414300** | **-3.85729000** | **1.02678800** |
| **H** | **-7.36103900** | **-1.87898100** | **1.04011100** |
| **H** | **-7.64947900** | **0.54573400** | **0.70386900** |
| **H** | **-2.14443700** | **0.84696300** | **0.05098600** |
| **H** | **-6.71832200** | **2.72632100** | **0.15051000** |
| **H** | **-3.97845900** | **6.44594200** | **-0.87833200** |
| **H** | **-5.87110100** | **4.93314500** | **-0.42146100** |
| **H** | **-0.63110800** | **2.15069300** | **0.19925800** |
| **H** | **-1.58616600** | **6.83919200** | **-1.05022400** |
| **H** | **3.13257200** | **6.67287900** | **-0.84770700** |
| **H** | **0.78355200** | **7.33904400** | **-1.18719800** |
| **H** | **1.22990300** | **1.69413700** | **0.71717200** |
| **H** | **4.77261300** | **5.04064700** | **-0.12632600** |
| **H** | **7.11370700** | **1.31502800** | **1.61522200** |
| **H** | **6.48208500** | **3.49386300** | **0.65044200** |
| **C** | **5.46672800** | **-0.70668500** | **2.23275300** |
| **C** | **4.52915600** | **-1.68501600** | **2.50461000** |
| **C** | **3.17492000** | **-1.44544700** | **2.21520300** |
| **C** | **2.77915800** | **-0.23716900** | **1.66744700** |
| **H** | **6.51899600** | **-0.87903800** | **2.44742400** |
| **H** | **4.83570200** | **-2.63485500** | **2.93380200** |
| **H** | **2.43251300** | **-2.21320300** | **2.41312200** |
| **H** | **1.72855700** | **-0.09260600** | **1.44222500** |
| **C** | **2.17198800** | **-1.67475200** | **-1.67990700** |

| **C** | **3.39703900** | **-1.08373200** | **-1.99624400** |
| --- | --- | --- | --- |
| **C** | **4.58885400** | **-1.81092500** | **-1.98887300** |
| **C** | **4.55877500** | **-3.16345800** | **-1.71010700** |
| **H** | **5.46611800** | **-3.76015400** | **-1.74768200** |
| **H** | **5.52354300** | **-1.31551800** | **-2.23584400** |
| **H** | **3.41118900** | **-0.03009100** | **-2.26622700** |
| **N** | **1.01875800** | **-0.86113700** | **-1.74631900** |
| **H** | **1.21357100** | **0.01868900** | **-2.21470300** |
| **H** | **0.23394900** | **-1.31629300** | **-2.20390300** |

Carboxyl Functionalized Expanded [13]-helicene:

X Y Z

**-----------------------------------------------------------------**

| **C** | **6.29277900** | **-0.10336600** | **-1.51646900** |
| --- | --- | --- | --- |
| **C** | **6.23844700** | **-1.36240100** | **-1.01096400** |
| **C** | **4.97977300** | **-1.98465900** | **-0.70523500** |
| **C** | **5.09656600** | **0.65836300** | **-1.75674200** |
| **C** | **3.82209300** | **0.09170900** | **-1.45666500** |
| **C** | **3.75811700** | **-1.26761400** | **-0.92234300** |
| **C** | **4.92230300** | **-3.29220300** | **-0.21018000** |
| **C** | **3.71313300** | **-3.93146800** | **0.06893800** |
| **C** | **2.48163800** | **-3.22410700** | **-0.13700500** |
| **C** | **2.54897800** | **-1.91388300** | **-0.62370300** |
| **C** | **3.69159400** | **-5.29354400** | **0.53579600** |
| **C** | **2.52172900** | **-5.94172800** | **0.75497700** |
| **C** | **1.25606900** | **-5.28562900** | **0.55081900** |
| **C** | **1.21573700** | **-3.91299700** | **0.13972300** |
| **C** | **-0.04102900** | **-3.30486700** | **-0.00010000** |
| **C** | **0.05675900** | **-5.98033100** | **0.72605000** |
| **C** | **-1.19022200** | **-5.38139300** | **0.52965400** |
| **C** | **-1.25031300** | **-3.98938400** | **0.19437200** |
| **C** | **-2.40138200** | **-6.15350800** | **0.62529800** |
| **C** | **-3.61081200** | **-5.59439600** | **0.37429900** |
| **C** | **-3.73392700** | **-4.19257200** | **0.07165600** |
| **C** | **-2.56599900** | **-3.36152300** | **0.03621500** |
| **C** | **-2.74549400** | **-1.98278200** | **-0.14138500** |
| **C** | **-4.00346100** | **-1.39421200** | **-0.33486700** |
| **C** | **-5.14726900** | **-2.25341800** | **-0.41195400** |
| **C** | **-4.98431000** | **-3.62187500** | **-0.18130300** |
| **C** | **-4.20277800** | **0.05105900** | **-0.46364600** |
| **C** | **-5.50390400** | **0.54488100** | **-0.80501600** |
| **C** | **-6.60394100** | **-0.37347800** | **-0.94395200** |
| **C** | **-6.43986700** | **-1.70226500** | **-0.72638900** |
| **C** | **-3.18432900** | **0.98250100** | **-0.23041300** |
| **C** | **-3.37123100** | **2.36539700** | **-0.34642800** |
| **C** | **-4.66005600** | **2.84293400** | **-0.74834900** |
| **C** | **-5.69033000** | **1.92197500** | **-0.95979400** |
| **C** | **-2.31871600** | **3.33887300** | **-0.04883500** |
| **C** | **-2.59455200** | **4.73545600** | **-0.21388100** |
| **C** | **-3.89910900** | **5.15832600** | **-0.65411000** |
| **C** | **-4.88314600** | **4.25792400** | **-0.90140400** |
| **C** | **-1.05160500** | **2.96448100** | **0.40654500** |
| **C** | **-0.04983800** | **3.88625000** | **0.73856600** |
| **C** | **-0.30842000** | **5.27332300** | **0.48116000** |
| **C** | **-1.57719000** | **5.65996100** | **0.03332900** |
| **C** | **1.25677200** | **3.49112100** | **1.26129900** |
| **C** | **2.30177500** | **4.46274700** | **1.26650100** |
| **C** | **2.00667400** | **5.83551200** | **0.95061600** |
| **C** | **0.75077100** | **6.23237300** | **0.62370700** |
| **H** | **7.24990300** | **0.35621600** | **-1.75177500** |
| **H** | **7.14929500** | **-1.92946000** | **-0.83446800** |
| **H** | **5.85212000** | **-3.83535800** | **-0.05282600** |

| **H** | **1.62635000** | **-1.37478300** | **-0.78696100** |
| --- | --- | --- | --- |
| **H** | **4.64251100** | **-5.79740400** | **0.69094700** |
| **H** | **2.51415400** | **-6.97688100** | **1.08736700** |
| **H** | **-0.08069300** | **-2.26693100** | **-0.30747600** |
| **H** | **0.09565200** | **-7.03175000** | **1.00397600** |
| **H** | **-2.31847500** | **-7.20836000** | **0.87515200** |
| **H** | **-4.51817500** | **-6.19188200** | **0.41536600** |
| **H** | **-1.87197800** | **-1.34187400** | **-0.12724400** |
| **H** | **-5.86157700** | **-4.26556900** | **-0.19626500** |
| **H** | **-7.58071800** | **0.03000700** | **-1.19921800** |
| **H** | **-7.28327100** | **-2.38433600** | **-0.80062600** |
| **H** | **-2.21905400** | **0.61542200** | **0.09269900** |
| **H** | **-6.67698700** | **2.29075300** | **-1.23311100** |
| **H** | **-4.07992800** | **6.22355300** | **-0.77514700** |
| **H** | **-5.86843000** | **4.58938600** | **-1.22006100** |
| **H** | **-0.82644800** | **1.91150900** | **0.47798500** |
| **H** | **-1.77067700** | **6.71593800** | **-0.14417000** |
| **H** | **2.81849700** | **6.55652900** | **1.00509300** |
| **H** | **0.53339700** | **7.27761000** | **0.41843200** |
| **C** | **3.61957100** | **4.08818500** | **1.60863400** |
| **C** | **3.92438100** | **2.78585600** | **1.94969500** |
| **C** | **2.89369400** | **1.83890500** | **2.02723200** |
| **C** | **1.57717400** | **2.18546800** | **1.73511800** |
| **H** | **4.39621400** | **4.84897000** | **1.59274100** |
| **H** | **4.94402500** | **2.49878100** | **2.18795500** |
| **C** | **2.67578400** | **0.88175900** | **-1.69700300** |
| **C** | **2.77456400** | **2.16227100** | **-2.21419900** |
| **C** | **4.03279700** | **2.71164000** | **-2.51535800** |
| **C** | **5.17362200** | **1.96581800** | **-2.28580700** |
| **H** | **6.15446200** | **2.37862300** | **-2.51095700** |
| **H** | **4.10644100** | **3.71632600** | **-2.92239300** |
| **C** | **0.54936300** | **1.18056100** | **2.15653000** |
| **O** | **-0.32278900** | **1.36917000** | **2.97409600** |
| **O** | **0.74298200** | **-0.03866800** | **1.57485700** |
| **H** | **0.11927800** | **-0.64990500** | **2.01382300** |
| **H** | **3.11180700** | **0.82785300** | **2.35575300** |
| **H** | **1.87297300** | **2.74481600** | **-2.38113900** |
| **H** | **1.69123200** | **0.49205000** | **-1.46444400** |

Bor Doped Expanded [13]-helicene:

X Y Z

**-----------------------------------------------------------------**

| **C** | **-4.04590600** | **-5.09119700** | **1.06560400** |
| --- | --- | --- | --- |
| **C** | **-5.02401600** | **-4.26977400** | **0.60510000** |
| **C** | **-4.78221800** | **-2.86717400** | **0.40707500** |
| **C** | **-2.73569100** | **-4.58965100** | **1.38277700** |
| **C** | **-2.44268900** | **-3.20510500** | **1.20160700** |
| **C** | **-3.48641500** | **-2.32102200** | **0.68517600** |
| **C** | **-5.79920900** | **-2.01543800** | **-0.03894100** |
| **C** | **-5.59656800** | **-0.64757800** | **-0.23115400** |
| **C** | **-4.29266000** | **-0.09541500** | **-0.00177300** |
| **C** | **-3.28482400** | **-0.95154000** | **0.45619000** |
| **C** | **-6.68141500** | **0.21369100** | **-0.62499400** |
| **C** | **-6.50333600** | **1.55157300** | **-0.75534000** |
| **C** | **-5.21332000** | **2.15843900** | **-0.55213400** |
| **C** | **-4.08312200** | **1.33858000** | **-0.22146500** |
| **C** | **-2.83172500** | **1.95887000** | **-0.11581700** |
| **C** | **-5.04703400** | **3.54235600** | **-0.67315400** |
| **C** | **-3.80446400** | **4.15910600** | **-0.50928000** |
| **C** | **-2.65742400** | **3.34187800** | **-0.25972200** |
| **C** | **-3.64759500** | **5.58912100** | **-0.57336500** |
| **C** | **-2.44063500** | **6.18409000** | **-0.39108000** |

| **C** | **-1.23479800** | **5.42133700** | **-0.17046500** |
| --- | --- | --- | --- |
| **C** | **-1.36264200** | **3.97024600** | **-0.14267600** |
| **C** | **1.36264300** | **3.97024600** | **0.14267600** |
| **C** | **1.23479900** | **5.42133700** | **0.17046500** |
| **C** | **0.00000100** | **6.07509500** | **0.00000000** |
| **C** | **2.65742400** | **3.34187700** | **0.25972200** |
| **C** | **3.80446500** | **4.15910500** | **0.50928000** |
| **C** | **3.64759600** | **5.58912100** | **0.57336500** |
| **C** | **2.44063600** | **6.18409000** | **0.39108000** |
| **C** | **2.83172600** | **1.95886900** | **0.11581700** |
| **C** | **4.08312300** | **1.33858000** | **0.22146400** |
| **C** | **5.21332100** | **2.15843800** | **0.55213400** |
| **C** | **5.04703400** | **3.54235600** | **0.67315300** |
| **C** | **4.29266000** | **-0.09541600** | **0.00177300** |
| **C** | **5.59656800** | **-0.64757900** | **0.23115400** |
| **C** | **6.68141500** | **0.21369000** | **0.62499300** |
| **C** | **6.50333700** | **1.55157100** | **0.75534000** |
| **C** | **3.28482400** | **-0.95154100** | **-0.45619000** |
| **C** | **3.48641400** | **-2.32102300** | **-0.68517600** |
| **C** | **4.78221700** | **-2.86717500** | **-0.40707500** |
| **C** | **5.79920800** | **-2.01543900** | **0.03894100** |
| **C** | **2.44268900** | **-3.20510500** | **-1.20160700** |
| **C** | **2.73569000** | **-4.58965200** | **-1.38277700** |
| **C** | **4.04590500** | **-5.09119700** | **-1.06560500** |
| **C** | **5.02401600** | **-4.26977500** | **-0.60510100** |
| **H** | **-4.23893400** | **-6.15123100** | **1.21247100** |
| **H** | **-6.01396300** | **-4.65822900** | **0.37850800** |
| **H** | **-6.78721600** | **-2.43153700** | **-0.22602600** |
| **H** | **-2.30942400** | **-0.53205900** | **0.66730800** |
| **H** | **-7.65768600** | **-0.23472400** | **-0.79190500** |
| **H** | **-7.33516900** | **2.19713100** | **-1.02644800** |
| **H** | **-1.95021700** | **1.35982600** | **0.09443400** |
| **H** | **-5.91664600** | **4.15788900** | **-0.89497600** |
| **H** | **-4.53327500** | **6.19308700** | **-0.75550800** |
| **H** | **-2.35180100** | **7.26738000** | **-0.42150400** |
| **H** | **0.00000100** | **7.16161400** | **0.00000000** |
| **H** | **4.53327600** | **6.19308700** | **0.75550900** |
| **H** | **2.35180300** | **7.26737900** | **0.42150400** |
| **H** | **1.95021800** | **1.35982600** | **-0.09443400** |
| **H** | **5.91664700** | **4.15788800** | **0.89497500** |
| **H** | **7.65768600** | **-0.23472500** | **0.79190500** |
| **H** | **7.33517000** | **2.19713000** | **1.02644700** |
| **H** | **2.30942400** | **-0.53206000** | **-0.66730800** |
| **H** | **6.78721500** | **-2.43153800** | **0.22602500** |
| **H** | **4.23893300** | **-6.15123200** | **-1.21247200** |
| **H** | **6.01396200** | **-4.65823000** | **-0.37850900** |
| **C** | **1.73751900** | **-5.45629900** | **-1.88022800** |
| **C** | **0.47786500** | **-4.98623200** | **-2.20082400** |
| **C** | **0.18470600** | **-3.62241300** | **-2.03159300** |
| **C** | **1.14780600** | **-2.75575600** | **-1.54347600** |
| **H** | **1.98027600** | **-6.50856700** | **-2.00973000** |
| **H** | **-0.28093000** | **-5.66432100** | **-2.58104200** |
| **H** | **-0.80317200** | **-3.24438000** | **-2.27863500** |
| **H** | **0.88923900** | **-1.70854700** | **-1.42882700** |
| **C** | **-1.14780700** | **-2.75575600** | **1.54347700** |
| **C** | **-0.18470700** | **-3.62241300** | **2.03159400** |
| **C** | **-0.47786700** | **-4.98623200** | **2.20082500** |
| **C** | **-1.73752000** | **-5.45629800** | **1.88022800** |
| **H** | **-1.98027700** | **-6.50856700** | **2.00973000** |
| **H** | **0.28092900** | **-5.66432100** | **2.58104300** |
| **H** | **0.80317100** | **-3.24438000** | **2.27863600** |
| **H** | **-0.88923900** | **-1.70854700** | **1.42882800** |
| **B** | **0.00000000** | **3.52963400** | **0.00000000** |

Nitrogene Doped Expanded [13]-helicene:

X Y Z

**-----------------------------------------------------------------**

| **C** | **-5.06629700** | **3.08937200** | **1.62499100** |
| --- | --- | --- | --- |
| **C** | **-4.36817200** | **4.12594400** | **1.09434600** |
| **C** | **-2.97323600** | **3.99383800** | **0.77549700** |
| **C** | **-4.43913600** | **1.82260000** | **1.89053600** |
| **C** | **-3.05825500** | **1.63648400** | **1.58537100** |
| **C** | **-2.30458100** | **2.74686600** | **1.00583000** |
| **C** | **-2.24570000** | **5.07238100** | **0.25874700** |
| **C** | **-0.88534300** | **4.97880800** | **-0.04069900** |
| **C** | **-0.21121500** | **3.72823800** | **0.15370300** |
| **C** | **-0.94422000** | **2.65580700** | **0.67399900** |
| **C** | **-0.14794600** | **6.12437600** | **-0.50850600** |
| **C** | **1.18718600** | **6.05776800** | **-0.73616300** |
| **C** | **1.91528900** | **4.82732800** | **-0.55845300** |
| **C** | **1.21769000** | **3.63818400** | **-0.15968900** |
| **C** | **1.94555500** | **2.44534900** | **-0.07407500** |
| **C** | **3.29846600** | **4.77928800** | **-0.76159900** |
| **C** | **4.03039100** | **3.59797000** | **-0.60670500** |
| **C** | **3.32579600** | **2.39438400** | **-0.29231600** |
| **C** | **5.46503200** | **3.57340400** | **-0.73479200** |
| **C** | **6.17135500** | **2.43120900** | **-0.53235700** |
| **C** | **5.50493400** | **1.19028700** | **-0.23627400** |
| **C** | **4.07428300** | **1.14643300** | **-0.17259400** |
| **C** | **4.07428300** | **-1.14643300** | **0.17259400** |
| **C** | **5.50493400** | **-1.19028700** | **0.23627400** |
| **C** | **6.19603600** | **0.00000000** | **0.00000000** |
| **C** | **3.32579600** | **-2.39438400** | **0.29231600** |
| **C** | **4.03039100** | **-3.59797000** | **0.60670500** |
| **C** | **5.46503200** | **-3.57340400** | **0.73479200** |
| **C** | **6.17135500** | **-2.43120900** | **0.53235700** |
| **C** | **1.94555500** | **-2.44534900** | **0.07407500** |
| **C** | **1.21769000** | **-3.63818400** | **0.15968900** |
| **C** | **1.91528800** | **-4.82732800** | **0.55845300** |
| **C** | **3.29846600** | **-4.77928800** | **0.76159900** |
| **C** | **-0.21121500** | **-3.72823800** | **-0.15370300** |
| **C** | **-0.88534300** | **-4.97880800** | **0.04069900** |
| **C** | **-0.14794600** | **-6.12437600** | **0.50850600** |
| **C** | **1.18718600** | **-6.05776800** | **0.73616300** |
| **C** | **-0.94422000** | **-2.65580700** | **-0.67399900** |
| **C** | **-2.30458100** | **-2.74686600** | **-1.00583000** |
| **C** | **-2.97323600** | **-3.99383800** | **-0.77549700** |
| **C** | **-2.24570000** | **-5.07238100** | **-0.25874700** |
| **C** | **-3.05825500** | **-1.63648400** | **-1.58537100** |
| **C** | **-4.43913600** | **-1.82260000** | **-1.89053700** |
| **C** | **-5.06629700** | **-3.08937200** | **-1.62499000** |
| **C** | **-4.36817200** | **-4.12594400** | **-1.09434600** |
| **H** | **-6.12105000** | **3.19995800** | **1.86545800** |
| **H** | **-4.85145600** | **5.08124500** | **0.90413100** |
| **H** | **-2.75438200** | **6.02190100** | **0.10351000** |
| **H** | **-0.42893100** | **1.72123200** | **0.85428200** |
| **H** | **-0.68771300** | **7.05738300** | **-0.65176600** |
| **H** | **1.73779500** | **6.93654600** | **-1.06286900** |
| **H** | **1.45889800** | **1.51226300** | **0.18145200** |
| **H** | **3.82166600** | **5.69396700** | **-1.03316200** |
| **H** | **5.97570800** | **4.50305700** | **-0.97354500** |
| **H** | **7.25656600** | **2.42960300** | **-0.60111900** |
| **H** | **7.28462000** | **0.00000000** | **0.00000000** |
| **H** | **5.97570800** | **-4.50305700** | **0.97354500** |
| **H** | **7.25656600** | **-2.42960300** | **0.60111900** |

| **H** | **1.45889800** | **-1.51226300** | **-0.18145200** |
| --- | --- | --- | --- |
| **H** | **3.82166600** | **-5.69396700** | **1.03316200** |
| **H** | **-0.68771300** | **-7.05738300** | **0.65176700** |
| **H** | **1.73779500** | **-6.93654600** | **1.06286900** |
| **H** | **-0.42893100** | **-1.72123200** | **-0.85428200** |
| **H** | **-2.75438200** | **-6.02190100** | **-0.10351000** |
| **H** | **-6.12105000** | **-3.19995800** | **-1.86545800** |
| **H** | **-4.85145600** | **-5.08124500** | **-0.90413100** |
| **C** | **-5.17990800** | **-0.76068400** | **-2.45478400** |
| **C** | **-4.58955100** | **0.46097400** | **-2.71811200** |
| **C** | **-3.22928300** | **0.65087700** | **-2.42009100** |
| **C** | **-2.48428500** | **-0.37687300** | **-1.86737400** |
| **H** | **-6.23164600** | **-0.92245400** | **-2.67987000** |
| **H** | **-5.17179200** | **1.26996900** | **-3.15046700** |
| **H** | **-2.75739000** | **1.60902000** | **-2.61783900** |
| **H** | **-1.43739700** | **-0.19497100** | **-1.65024200** |
| **C** | **-2.48428500** | **0.37687300** | **1.86737400** |
| **C** | **-3.22928300** | **-0.65087700** | **2.42009100** |
| **C** | **-4.58955100** | **-0.46097400** | **2.71811100** |
| **C** | **-5.17990800** | **0.76068400** | **2.45478400** |
| **H** | **-6.23164600** | **0.92245400** | **2.67987000** |
| **H** | **-5.17179200** | **-1.26996900** | **3.15046700** |
| **H** | **-2.75739000** | **-1.60902000** | **2.61783900** |
| **H** | **-1.43739700** | **0.19497100** | **1.65024200** |
| **N** | **3.40217300** | **0.00000000** | **0.00000000** |

Silicone Doped Expanded [13]-helicene:

X Y Z

**-----------------------------------------------------------------**

| **C** | **5.02719000** | **5.09694700** | **0.78057800** |
| --- | --- | --- | --- |
| **C** | **5.93820000** | **4.16225400** | **0.40687500** |
| **C** | **5.56388900** | **2.78425600** | **0.24669500** |
| **C** | **3.65610200** | **4.74439000** | **1.03433900** |
| **C** | **3.23176200** | **3.39023500** | **0.88537700** |
| **C** | **4.20729600** | **2.38223600** | **0.47386300** |
| **C** | **6.50796000** | **1.81807600** | **-0.11836600** |
| **C** | **6.17615300** | **0.47101500** | **-0.27277000** |
| **C** | **4.81658000** | **0.05741800** | **-0.07455800** |
| **C** | **3.88062300** | **1.02924800** | **0.29623800** |
| **C** | **7.18299000** | **-0.50280200** | **-0.60722100** |
| **C** | **6.87628600** | **-1.81844100** | **-0.72014100** |
| **C** | **5.52776100** | **-2.28898000** | **-0.53971400** |
| **C** | **4.47591000** | **-1.35915100** | **-0.24687300** |
| **C** | **3.17171000** | **-1.85599800** | **-0.13231000** |
| **C** | **5.22474300** | **-3.64909600** | **-0.64143000** |
| **C** | **3.92484600** | **-4.13739700** | **-0.48824800** |
| **C** | **2.85152700** | **-3.21740900** | **-0.25540300** |
| **C** | **3.64639800** | **-5.54638200** | **-0.54552800** |
| **C** | **2.38968400** | **-6.02216400** | **-0.37099900** |
| **C** | **1.25871100** | **-5.14393400** | **-0.16528200** |
| **C** | **1.48695400** | **-3.72350100** | **-0.14151100** |
| **C** | **-1.48695400** | **-3.72350100** | **0.14151100** |
| **C** | **-1.25871100** | **-5.14393400** | **0.16528200** |
| **C** | **0.00000000** | **-5.74931900** | **0.00000000** |
| **C** | **-2.85152600** | **-3.21740900** | **0.25540300** |
| **C** | **-3.92484600** | **-4.13739700** | **0.48824800** |
| **C** | **-3.64639800** | **-5.54638200** | **0.54552800** |
| **C** | **-2.38968400** | **-6.02216400** | **0.37099900** |
| **C** | **-3.17170900** | **-1.85599800** | **0.13231000** |
| **C** | **-4.47591000** | **-1.35915100** | **0.24687300** |
| **C** | **-5.52776000** | **-2.28898100** | **0.53971400** |
| **C** | **-5.22474300** | **-3.64909600** | **0.64143000** |

| **C** | **-4.81658000** | **0.05741800** | **0.07455800** |
| --- | --- | --- | --- |
| **C** | **-6.17615300** | **0.47101500** | **0.27277000** |
| **C** | **-7.18299000** | **-0.50280200** | **0.60722200** |
| **C** | **-6.87628600** | **-1.81844100** | **0.72014100** |
| **C** | **-3.88062300** | **1.02924800** | **-0.29623800** |
| **C** | **-4.20729600** | **2.38223500** | **-0.47386300** |
| **C** | **-5.56388900** | **2.78425600** | **-0.24669500** |
| **C** | **-6.50796000** | **1.81807600** | **0.11836600** |
| **C** | **-3.23176200** | **3.39023500** | **-0.88537700** |
| **C** | **-3.65610200** | **4.74439000** | **-1.03433900** |
| **C** | **-5.02719000** | **5.09694700** | **-0.78057800** |
| **C** | **-5.93820000** | **4.16225400** | **-0.40687500** |
| **H** | **5.32038200** | **6.13716100** | **0.90055100** |
| **H** | **6.97352000** | **4.43882600** | **0.22302500** |
| **H** | **7.53959200** | **2.12625900** | **-0.27646700** |
| **H** | **2.85780200** | **0.72046900** | **0.47001100** |
| **H** | **8.20332800** | **-0.15633200** | **-0.75175200** |
| **H** | **7.64623300** | **-2.54917000** | **-0.95546600** |
| **H** | **2.36433700** | **-1.16076300** | **0.06896700** |
| **H** | **6.03024400** | **-4.35364000** | **-0.83942300** |
| **H** | **4.47618900** | **-6.22821100** | **-0.71611800** |
| **H** | **2.20090100** | **-7.09242800** | **-0.39627000** |
| **H** | **0.00000000** | **-1.26765600** | **0.00000000** |
| **H** | **0.00000000** | **-6.83703700** | **0.00000000** |
| **H** | **-4.47618900** | **-6.22821100** | **0.71611800** |
| **H** | **-2.20090100** | **-7.09242800** | **0.39627000** |
| **H** | **-2.36433700** | **-1.16076300** | **-0.06896700** |
| **H** | **-6.03024300** | **-4.35364000** | **0.83942300** |
| **H** | **-8.20332800** | **-0.15633200** | **0.75175200** |
| **H** | **-7.64623300** | **-2.54917000** | **0.95546600** |
| **H** | **-2.85780200** | **0.72046900** | **-0.47001100** |
| **H** | **-7.53959200** | **2.12625900** | **0.27646700** |
| **H** | **-5.32038200** | **6.13716100** | **-0.90055100** |
| **H** | **-6.97352000** | **4.43882600** | **-0.22302500** |
| **C** | **-2.72550000** | **5.72899200** | **-1.43450100** |
| **C** | **-1.40585900** | **5.40497200** | **-1.68720500** |
| **C** | **-0.98128200** | **4.07256600** | **-1.54469800** |
| **C** | **-1.87682600** | **3.09153800** | **-1.15293300** |
| **H** | **-3.06925500** | **6.75524200** | **-1.54240200** |
| **H** | **-0.70018000** | **6.17217800** | **-1.99302000** |
| **H** | **0.05492100** | **3.81004600** | **-1.73570100** |
| **H** | **-1.51690100** | **2.07268900** | **-1.05631100** |
| **C** | **1.87682600** | **3.09153800** | **1.15293300** |
| **C** | **0.98128200** | **4.07256600** | **1.54469800** |
| **C** | **1.40585900** | **5.40497200** | **1.68720400** |
| **C** | **2.72550000** | **5.72899200** | **1.43450100** |
| **H** | **3.06925500** | **6.75524200** | **1.54240200** |
| **H** | **0.70018000** | **6.17217800** | **1.99302000** |
| **H** | **-0.05492100** | **3.81004600** | **1.73570100** |
| **H** | **1.51690100** | **2.07268900** | **1.05631100** |
| **Si** | **0.00000000** | **-2.74362100** | **0.00000000** |

Phosphore Doped Expanded [13]-helicene:

X Y Z

**-----------------------------------------------------------------**

| **C** | **4.59274800** | **5.11087300** | **1.00961200** |
| --- | --- | --- | --- |
| **C** | **5.53136700** | **4.23029000** | **0.57714900** |
| **C** | **5.20849500** | **2.84574700** | **0.36869200** |
| **C** | **3.24310400** | **4.69309300** | **1.27890500** |
| **C** | **2.86958500** | **3.32976300** | **1.08422600** |
| **C** | **3.87486700** | **2.38000500** | **0.61028700** |
| **C** | **6.18109300** | **1.93432100** | **-0.05799100** |

| **C** | **5.89867800** | **0.58228300** | **-0.25922000** |
| --- | --- | --- | --- |
| **C** | **4.56251500** | **0.10649400** | **-0.04532300** |
| **C** | **3.59733200** | **1.02297100** | **0.38564300** |
| **C** | **6.93369100** | **-0.33693200** | **-0.65732200** |
| **C** | **6.67793800** | **-1.65884300** | **-0.81548200** |
| **C** | **5.35420100** | **-2.19050700** | **-0.61947500** |
| **C** | **4.27492200** | **-1.31517400** | **-0.26489300** |
| **C** | **2.99470700** | **-1.86519900** | **-0.13591700** |
| **C** | **5.10223800** | **-3.55676200** | **-0.76184300** |
| **C** | **3.82671500** | **-4.10238300** | **-0.59045700** |
| **C** | **2.72469200** | **-3.23352200** | **-0.30040300** |
| **C** | **3.61152600** | **-5.52032200** | **-0.68117400** |
| **C** | **2.38314600** | **-6.05300100** | **-0.47590900** |
| **C** | **1.23191000** | **-5.22116000** | **-0.21295200** |
| **C** | **1.37762000** | **-3.79889400** | **-0.16967900** |
| **C** | **-1.37762000** | **-3.79889400** | **0.16967900** |
| **C** | **-1.23191000** | **-5.22116000** | **0.21295200** |
| **C** | **0.00000000** | **-5.85015700** | **0.00000000** |
| **C** | **-2.72469200** | **-3.23352200** | **0.30040300** |
| **C** | **-3.82671500** | **-4.10238300** | **0.59045700** |
| **C** | **-3.61152600** | **-5.52032200** | **0.68117400** |
| **C** | **-2.38314600** | **-6.05300200** | **0.47590800** |
| **C** | **-2.99470700** | **-1.86519900** | **0.13591700** |
| **C** | **-4.27492200** | **-1.31517500** | **0.26489300** |
| **C** | **-5.35420100** | **-2.19050700** | **0.61947500** |
| **C** | **-5.10223800** | **-3.55676300** | **0.76184300** |
| **C** | **-4.56251500** | **0.10649400** | **0.04532300** |
| **C** | **-5.89867800** | **0.58228300** | **0.25922000** |
| **C** | **-6.93369000** | **-0.33693200** | **0.65732200** |
| **C** | **-6.67793800** | **-1.65884300** | **0.81548200** |
| **C** | **-3.59733200** | **1.02297100** | **-0.38564300** |
| **C** | **-3.87486700** | **2.38000400** | **-0.61028700** |
| **C** | **-5.20849500** | **2.84574700** | **-0.36869200** |
| **C** | **-6.18109300** | **1.93432100** | **0.05799100** |
| **C** | **-2.86958500** | **3.32976300** | **-1.08422600** |
| **C** | **-3.24310400** | **4.69309300** | **-1.27890500** |
| **C** | **-4.59274800** | **5.11087300** | **-1.00961200** |
| **C** | **-5.53136700** | **4.23028900** | **-0.57714900** |
| **H** | **4.84708200** | **6.15652400** | **1.16588900** |
| **H** | **6.55007100** | **4.55628600** | **0.38174000** |
| **H** | **7.19534700** | **2.29037800** | **-0.22764300** |
| **H** | **2.59225700** | **0.66586600** | **0.56928600** |
| **H** | **7.93542700** | **0.05608000** | **-0.81307900** |
| **H** | **7.47029400** | **-2.34736800** | **-1.09861000** |
| **H** | **2.16510500** | **-1.21355400** | **0.11103200** |
| **H** | **5.92891100** | **-4.22172800** | **-1.00396400** |
| **H** | **4.46452900** | **-6.15896800** | **-0.89712900** |
| **H** | **2.23323500** | **-7.12890300** | **-0.51926100** |
| **H** | **0.00000000** | **-6.93860500** | **0.00000000** |
| **H** | **-4.46452900** | **-6.15896800** | **0.89712900** |
| **H** | **-2.23323500** | **-7.12890300** | **0.51926100** |
| **H** | **-2.16510500** | **-1.21355400** | **-0.11103200** |
| **H** | **-5.92891100** | **-4.22172800** | **1.00396400** |
| **H** | **-7.93542700** | **0.05608000** | **0.81308000** |
| **H** | **-7.47029400** | **-2.34736900** | **1.09861000** |
| **H** | **-2.59225700** | **0.66586500** | **-0.56928600** |
| **H** | **-7.19534700** | **2.29037800** | **0.22764300** |
| **H** | **-4.84708200** | **6.15652400** | **-1.16588900** |
| **H** | **-6.55007100** | **4.55628600** | **-0.38174000** |
| **C** | **-2.28354300** | **5.62217100** | **-1.73848700** |
| **C** | **-0.98394600** | **5.23430600** | **-2.00506700** |
| **C** | **-0.60992600** | **3.89270400** | **-1.81717700** |
| **C** | **-1.53438700** | **2.96480600** | **-1.36798800** |

| **H** | **-2.58812000** | **6.65661400** | **-1.88054500** |
| --- | --- | --- | --- |
| **H** | **-0.25525400** | **5.95967300** | **-2.35578700** |
| **H** | **0.41056300** | **3.58030600** | **-2.01839500** |
| **H** | **-1.21215400** | **1.93744900** | **-1.23622200** |
| **C** | **1.53438700** | **2.96480600** | **1.36798700** |
| **C** | **0.60992600** | **3.89270400** | **1.81717700** |
| **C** | **0.98394600** | **5.23430600** | **2.00506700** |
| **C** | **2.28354200** | **5.62217100** | **1.73848700** |
| **H** | **2.58812000** | **6.65661400** | **1.88054500** |
| **H** | **0.25525400** | **5.95967300** | **2.35578600** |
| **H** | **-0.41056300** | **3.58030600** | **2.01839500** |
| **H** | **1.21215400** | **1.93744900** | **1.23622200** |
| **P** | **0.00000000** | **-2.71355100** | **0.00000000** |

Expanded [14]-helicene:

X Y Z

**-----------------------------------------------------------------**

| **C** | **-3.40456100** | **-5.15381500** | **-0.93368300** |
| --- | --- | --- | --- |
| **C** | **-2.32569600** | **-5.83444500** | **-0.47832100** |
| **C** | **-1.05175100** | **-5.18362300** | **-0.29651200** |
| **C** | **-3.32874000** | **-3.74988700** | **-1.25571600** |
| **C** | **-2.08144100** | **-3.04503000** | **-1.07898700** |
| **C** | **-0.91459300** | **-3.78687100** | **-0.58225300** |
| **C** | **0.06371500** | **-5.90210700** | **0.13861400** |
| **C** | **1.31920400** | **-5.30787200** | **0.30390000** |
| **C** | **1.46610700** | **-3.90627400** | **0.04957900** |
| **C** | **0.33916300** | **-3.19485700** | **-0.38824300** |
| **C** | **2.46110300** | **-6.09170600** | **0.69518200** |
| **C** | **3.69331100** | **-5.53426900** | **0.79809400** |
| **C** | **3.89811900** | **-4.13026400** | **0.55700100** |
| **C** | **2.78444700** | **-3.29123900** | **0.22397300** |
| **C** | **3.01888800** | **-1.91791600** | **0.06838900** |
| **C** | **5.17635100** | **-3.56885200** | **0.62962500** |
| **C** | **5.40719900** | **-2.20747200** | **0.41636200** |
| **C** | **4.29151500** | **-1.34153200** | **0.17273000** |
| **C** | **6.74457900** | **-1.67398500** | **0.41170400** |
| **C** | **6.97609300** | **-0.36185000** | **0.15841300** |
| **C** | **5.88968900** | **0.56068600** | **-0.04616300** |
| **C** | **4.53585400** | **0.09453600** | **0.01540700** |
| **C** | **3.50780400** | **1.04266500** | **-0.06750100** |
| **C** | **3.74272600** | **2.41094700** | **-0.25816200** |
| **C** | **5.09745800** | **2.84499700** | **-0.43089400** |
| **C** | **6.13171300** | **1.91454900** | **-0.29485500** |
| **C** | **2.67306700** | **3.41103200** | **-0.29339000** |
| **C** | **3.00127400** | **4.76079300** | **-0.64422100** |
| **C** | **4.37376500** | **5.12815900** | **-0.87671800** |
| **C** | **5.37487000** | **4.22245700** | **-0.74471000** |
| **C** | **1.34171200** | **3.11866600** | **0.03107100** |
| **C** | **0.31794000** | **4.07457300** | **0.00160900** |
| **C** | **0.64760200** | **5.40105600** | **-0.42902900** |
| **C** | **1.97764800** | **5.70902000** | **-0.72815200** |
| **C** | **-1.06248300** | **3.78403700** | **0.40184500** |
| **C** | **-2.05070900** | **4.81615700** | **0.27534500** |
| **C** | **-1.67154100** | **6.11664300** | **-0.21425600** |
| **C** | **-0.38493600** | **6.39959500** | **-0.53489800** |
| **C** | **-1.46185200** | **2.55130000** | **0.93086800** |
| **C** | **-2.77783900** | **2.27737100** | **1.33279500** |
| **C** | **-3.75878500** | **3.31073400** | **1.17837800** |
| **C** | **-3.36735400** | **4.54828400** | **0.65476000** |
| **C** | **-3.18462600** | **0.99567200** | **1.90607000** |
| **C** | **-4.54676300** | **0.80947500** | **2.28631500** |
| **C** | **-5.49348700** | **1.87630800** | **2.10190000** |

| **C** | **-5.11887400** | **3.06973900** | **1.57413700** |
| --- | --- | --- | --- |
| **H** | **-4.35832500** | **-5.65751400** | **-1.07139800** |
| **H** | **-2.39647800** | **-6.89407400** | **-0.24509800** |
| **H** | **-0.04273100** | **-6.96646400** | **0.33896800** |
| **H** | **0.45069100** | **-2.14186500** | **-0.61375700** |
| **H** | **2.31469200** | **-7.15220500** | **0.88504200** |
| **H** | **4.55497800** | **-6.13917000** | **1.06958900** |
| **H** | **2.18028600** | **-1.27315800** | **-0.16311500** |
| **H** | **6.02289400** | **-4.21785700** | **0.84499500** |
| **H** | **7.57030800** | **-2.35852300** | **0.58964900** |
| **H** | **7.99128400** | **0.02592100** | **0.12532500** |
| **H** | **2.48376500** | **0.70208000** | **0.02302600** |
| **H** | **7.16098400** | **2.25660500** | **-0.38328800** |
| **H** | **4.59157200** | **6.16162400** | **-1.13480000** |
| **H** | **6.41135100** | **4.51635800** | **-0.89105900** |
| **H** | **1.09837800** | **2.10988500** | **0.33997300** |
| **H** | **2.22749100** | **6.72677600** | **-1.02141700** |
| **H** | **-2.44441200** | **6.87639300** | **-0.30190400** |
| **H** | **-0.10526200** | **7.39103100** | **-0.88270100** |
| **H** | **-0.71525100** | **1.77798800** | **1.05802600** |
| **H** | **-4.11328400** | **5.33425100** | **0.55343200** |
| **H** | **-6.52503100** | **1.70373900** | **2.39945900** |
| **H** | **-5.84271000** | **3.87041400** | **1.44287400** |
| **C** | **-4.95508000** | **-0.42256200** | **2.84316800** |
| **C** | **-4.05653400** | **-1.45664800** | **3.02663900** |
| **C** | **-2.71294800** | **-1.27999400** | **2.65435100** |
| **C** | **-2.29112200** | **-0.07958100** | **2.10851400** |
| **H** | **-5.99811600** | **-0.54478100** | **3.12604800** |
| **H** | **-4.38507800** | **-2.40019000** | **3.45334700** |
| **H** | **-2.00027100** | **-2.08864500** | **2.78919200** |
| **H** | **-1.24670400** | **0.02279900** | **1.83419300** |
| **C** | **-2.04688800** | **-1.69105900** | **-1.39846000** |
| **C** | **-3.17317700** | **-0.99650000** | **-1.88418000** |
| **C** | **-4.40897100** | **-1.70654100** | **-2.06373900** |
| **C** | **-4.44586100** | **-3.07402800** | **-1.73794300** |
| **H** | **-5.37725100** | **-3.62189400** | **-1.86758800** |
| **H** | **-1.12933100** | **-1.12300300** | **-1.28084200** |
| **C** | **-3.12757100** | **0.39142900** | **-2.20355700** |
| **C** | **-4.24086800** | **1.04082500** | **-2.67837300** |
| **C** | **-5.46149800** | **0.33591300** | **-2.86137700** |
| **C** | **-5.54304400** | **-1.00177200** | **-2.56092700** |
| **H** | **-6.47587400** | **-1.54410200** | **-2.69692200** |
| **H** | **-6.33295900** | **0.86400600** | **-3.23899800** |
| **H** | **-4.19338400** | **2.10055400** | **-2.91270300** |
| **H** | **-2.19617700** | **0.93262400** | **-2.05665500** |

Hydroxyl Functionalized Expanded [14]-helicene:

X Y Z

**-----------------------------------------------------------------**

| **C** | **-4.07033700** | **-4.55390700** | **-0.88716600** |
| --- | --- | --- | --- |
| **C** | **-3.11027800** | **-5.35072900** | **-0.36001000** |
| **C** | **-1.75474300** | **-4.88449700** | **-0.20105700** |
| **C** | **-3.78306600** | **-3.20462700** | **-1.30804300** |
| **C** | **-2.44800300** | **-2.67828700** | **-1.15192500** |
| **C** | **-1.40907200** | **-3.54614900** | **-0.57912400** |
| **C** | **-0.75885700** | **-5.73311900** | **0.28746200** |
| **C** | **0.57371400** | **-5.32812000** | **0.42131500** |
| **C** | **0.92765900** | **-3.98176600** | **0.08861600** |
| **C** | **-0.07977000** | **-3.13590000** | **-0.40474000** |
| **C** | **1.59099000** | **-6.25509900** | **0.84393000** |
| **C** | **2.89648600** | **-5.88928500** | **0.89641700** |
| **C** | **3.30721400** | **-4.54538900** | **0.58517200** |
| **C** | **2.32349600** | **-3.56399400** | **0.23335100** |
| **C** | **2.75067600** | **-2.24502800** | **0.03615200** |

| **C** | **4.65640500** | **-4.17719900** | **0.61396700** |
| --- | --- | --- | --- |
| **C** | **5.07884400** | **-2.87031500** | **0.35345100** |
| **C** | **4.09425100** | **-1.85851100** | **0.11118500** |
| **C** | **6.47764100** | **-2.52931400** | **0.30642100** |
| **C** | **6.88480800** | **-1.26523200** | **0.02614500** |
| **C** | **5.93562500** | **-0.19828700** | **-0.16205700** |
| **C** | **4.53353100** | **-0.47439900** | **-0.06705700** |
| **C** | **3.64118600** | **0.60132900** | **-0.12658800** |
| **C** | **4.05374500** | **1.92495900** | **-0.31791600** |
| **C** | **5.44973000** | **2.17578300** | **-0.51958900** |
| **C** | **6.35285600** | **1.11233500** | **-0.41552500** |
| **C** | **3.12262200** | **3.05366000** | **-0.31412300** |
| **C** | **3.61643600** | **4.35855500** | **-0.63577400** |
| **C** | **5.02008800** | **4.54523800** | **-0.89898800** |
| **C** | **5.89652900** | **3.51193800** | **-0.81844700** |
| **C** | **1.77093500** | **2.91997700** | **0.02585000** |
| **C** | **0.87837600** | **3.99798200** | **0.05015400** |
| **C** | **1.37181900** | **5.28960100** | **-0.32814600** |
| **C** | **2.72365300** | **5.43459100** | **-0.65600100** |
| **C** | **-0.52104600** | **3.86358800** | **0.46552000** |
| **C** | **-1.35585400** | **5.02563800** | **0.44981400** |
| **C** | **-0.82110700** | **6.29243800** | **0.02120100** |
| **C** | **0.47900900** | **6.41979900** | **-0.34370500** |
| **C** | **-1.06653800** | **2.64881800** | **0.90235300** |
| **C** | **-2.39602500** | **2.51246700** | **1.34105100** |
| **C** | **-3.21362400** | **3.69586500** | **1.33227400** |
| **C** | **-2.67799900** | **4.90604500** | **0.87913700** |
| **C** | **-2.98126300** | **1.25688700** | **1.82481100** |
| **C** | **-4.32752300** | **1.27128700** | **2.31505500** |
| **C** | **-5.08906300** | **2.49081800** | **2.28804200** |
| **C** | **-4.56568800** | **3.64440300** | **1.80845000** |
| **H** | **-5.08674200** | **-4.92027600** | **-1.00941300** |
| **H** | **-3.34007400** | **-6.36843200** | **-0.05414100** |
| **H** | **-1.02383800** | **-6.75588400** | **0.54841800** |
| **H** | **0.19751400** | **-2.13898200** | **-0.72602700** |
| **H** | **1.28929200** | **-7.26978300** | **1.09172600** |
| **H** | **3.66140500** | **-6.60606400** | **1.18484500** |
| **H** | **2.01060500** | **-1.48990100** | **-0.19743300** |
| **H** | **5.40286400** | **-4.93666100** | **0.83811800** |
| **H** | **7.20432700** | **-3.31981400** | **0.47709000** |
| **H** | **7.94380100** | **-1.02664800** | **-0.03577100** |
| **H** | **2.58431500** | **0.40256600** | **-0.00467900** |
| **H** | **7.41667100** | **1.31362100** | **-0.52558200** |
| **H** | **5.36777900** | **5.54708900** | **-1.13928600** |
| **H** | **6.95823800** | **3.67204600** | **-0.98967000** |
| **H** | **1.40368200** | **1.94306300** | **0.31144700** |
| **H** | **3.09746600** | **6.42302800** | **-0.91620900** |
| **H** | **-1.48589500** | **7.15296000** | **0.01146500** |
| **H** | **0.87693800** | **7.38400000** | **-0.65076200** |
| **H** | **-0.43858600** | **1.77518300** | **0.92825500** |
| **H** | **-3.31349400** | **5.78957100** | **0.88039800** |
| **H** | **-6.10975600** | **2.45879300** | **2.66068800** |
| **H** | **-5.15530900** | **4.55772700** | **1.78756600** |
| **C** | **-4.93995400** | **0.10496600** | **2.81920800** |
| **C** | **-4.25569300** | **-1.09332300** | **2.84328800** |
| **C** | **-2.94767200** | **-1.14513800** | **2.35308500** |
| **C** | **-2.32337600** | **-0.00836400** | **1.84988800** |
| **H** | **-5.96165400** | **0.16764100** | **3.18348000** |
| **H** | **-4.72308300** | **-1.99460900** | **3.22925400** |
| **H** | **-2.40494000** | **-2.08823600** | **2.34849600** |
| **C** | **-2.20923100** | **-1.36973200** | **-1.56129300** |
| **C** | **-3.21635000** | **-0.55428600** | **-2.11586400** |
| **C** | **-4.53962700** | **-1.08863400** | **-2.27952600** |

| **C** | **-4.78175900** | **-2.40977500** | **-1.86283200** |
| --- | --- | --- | --- |
| **H** | **-5.78190100** | **-2.82353500** | **-1.97609300** |
| **H** | **-1.22562200** | **-0.92697500** | **-1.44625900** |
| **C** | **-2.96324200** | **0.78822900** | **-2.52099100** |
| **C** | **-3.96224500** | **1.55752300** | **-3.06564300** |
| **C** | **-5.26944800** | **1.02530900** | **-3.23453600** |
| **C** | **-5.55077800** | **-0.26282700** | **-2.84999200** |
| **H** | **-6.55078000** | **-0.67196400** | **-2.97355500** |
| **H** | **-6.04824900** | **1.64713300** | **-3.66774700** |
| **H** | **-3.75760800** | **2.58107500** | **-3.36681500** |
| **H** | **-1.96556000** | **1.19719500** | **-2.38285400** |
| **O** | **-1.04545500** | **-0.12331700** | **1.35889200** |
| **H** | **-0.79271900** | **-1.06116000** | **1.39329400** |

Methyl Functionalized Expanded [14]-helicene:

X Y Z

**-----------------------------------------------------------------**

| **C** | **-3.53288500** | **-5.03687200** | **-0.92377200** |
| --- | --- | --- | --- |
| **C** | **-2.46681700** | **-5.75270200** | **-0.49304200** |
| **C** | **-1.17029100** | **-5.14207000** | **-0.33303200** |
| **C** | **-3.41920900** | **-3.63510600** | **-1.24392900** |
| **C** | **-2.14664600** | **-2.97069500** | **-1.09249700** |
| **C** | **-0.99510100** | **-3.74924800** | **-0.61669700** |
| **C** | **-0.07036800** | **-5.89593300** | **0.08116200** |
| **C** | **1.20441200** | **-5.34017000** | **0.23120400** |
| **C** | **1.38914900** | **-3.94190000** | **-0.01705200** |
| **C** | **0.27841400** | **-3.19579100** | **-0.43820800** |
| **C** | **2.32747400** | **-6.15984800** | **0.60287200** |
| **C** | **3.57625200** | **-5.63892400** | **0.69584600** |
| **C** | **3.81831200** | **-4.23920800** | **0.46470900** |
| **C** | **2.72622300** | **-3.36589900** | **0.14876500** |
| **C** | **2.99821600** | **-1.99813800** | **0.00422800** |
| **C** | **5.11217300** | **-3.71462000** | **0.53306100** |
| **C** | **5.37956200** | **-2.35801500** | **0.33308200** |
| **C** | **4.28718600** | **-1.45834400** | **0.10566300** |
| **C** | **6.73148500** | **-1.86281300** | **0.32822200** |
| **C** | **6.99861900** | **-0.55444900** | **0.09185500** |
| **C** | **5.93760400** | **0.40055700** | **-0.09564200** |
| **C** | **4.57116500** | **-0.02773400** | **-0.03643300** |
| **C** | **3.57031800** | **0.95049700** | **-0.10632100** |
| **C** | **3.84368000** | **2.31421900** | **-0.27929600** |
| **C** | **5.21052300** | **2.71178800** | **-0.44577300** |
| **C** | **6.21767600** | **1.75028300** | **-0.32518800** |
| **C** | **2.80293000** | **3.34534600** | **-0.30233500** |
| **C** | **3.17219800** | **4.69054100** | **-0.62982500** |
| **C** | **4.55509800** | **5.02188200** | **-0.85397500** |
| **C** | **5.52895000** | **4.08521800** | **-0.73717200** |
| **C** | **1.46170200** | **3.08694000** | **0.01070000** |
| **C** | **0.46483000** | **4.07188900** | **-0.01093300** |
| **C** | **0.83770100** | **5.39666600** | **-0.41243300** |
| **C** | **2.17792900** | **5.67017000** | **-0.69904300** |
| **C** | **-0.92822200** | **3.81719200** | **0.37024600** |
| **C** | **-1.87913100** | **4.87933300** | **0.25291500** |
| **C** | **-1.45885000** | **6.18041600** | **-0.19785700** |
| **C** | **-0.16125300** | **6.43023900** | **-0.50133800** |
| **C** | **-1.36736500** | **2.58280200** | **0.86876800** |
| **C** | **-2.69136500** | **2.31987800** | **1.26590100** |
| **C** | **-3.64363700** | **3.37924600** | **1.05465100** |
| **C** | **-3.21064900** | **4.62103800** | **0.57811200** |
| **C** | **-3.15537000** | **1.04252100** | **1.83448800** |
| **C** | **-4.57205300** | **0.86789400** | **1.98184900** |
| **C** | **-5.48172400** | **1.94494500** | **1.69957000** |

| **C** | **-5.03962300** | **3.15832600** | **1.29428100** |
| --- | --- | --- | --- |
| **H** | **-4.50460600** | **-5.50989900** | **-1.04367500** |
| **H** | **-2.56584700** | **-6.81055200** | **-0.26202200** |
| **H** | **-0.20575500** | **-6.95754400** | **0.27862500** |
| **H** | **0.41814200** | **-2.14536800** | **-0.65996100** |
| **H** | **2.15248700** | **-7.21709800** | **0.78657800** |
| **H** | **4.42319500** | **-6.27030200** | **0.95302400** |
| **H** | **2.17617600** | **-1.32783900** | **-0.21378400** |
| **H** | **5.94168500** | **-4.38911900** | **0.73603100** |
| **H** | **7.53822300** | **-2.57277800** | **0.49317300** |
| **H** | **8.02405800** | **-0.19456800** | **0.05960700** |
| **H** | **2.53705200** | **0.63807900** | **-0.01887600** |
| **H** | **7.25618400** | **2.06408700** | **-0.41034000** |
| **H** | **4.80308700** | **6.05284900** | **-1.09423900** |
| **H** | **6.57382400** | **4.35057500** | **-0.87823400** |
| **H** | **1.18914800** | **2.08126200** | **0.30515700** |
| **H** | **2.45952100** | **6.68555400** | **-0.97105300** |
| **H** | **-2.20827000** | **6.96443800** | **-0.27487500** |
| **H** | **0.15301400** | **7.42012700** | **-0.82309500** |
| **H** | **-0.64151400** | **1.79418100** | **0.93916900** |
| **H** | **-3.94620200** | **5.41286400** | **0.45016200** |
| **H** | **-6.54327000** | **1.76314400** | **1.84801300** |
| **H** | **-5.73219200** | **3.97663600** | **1.11356100** |
| **C** | **-5.11302900** | **-0.34945500** | **2.44329600** |
| **C** | **-4.28889900** | **-1.39520800** | **2.79804000** |
| **C** | **-2.90352600** | **-1.21360100** | **2.73138400** |
| **C** | **-2.31637000** | **-0.03316300** | **2.27725900** |
| **H** | **-6.19344100** | **-0.44084200** | **2.52294900** |
| **H** | **-4.70048100** | **-2.33671800** | **3.15059700** |
| **H** | **-2.25162600** | **-2.02013000** | **3.05654300** |
| **C** | **-2.07446700** | **-1.61918800** | **-1.41639100** |
| **C** | **-3.18767900** | **-0.88864500** | **-1.87908700** |
| **C** | **-4.44992400** | **-1.55775400** | **-2.02949400** |
| **C** | **-4.52395000** | **-2.92327600** | **-1.70225100** |
| **H** | **-5.47490900** | **-3.44098200** | **-1.81249300** |
| **H** | **-1.13546800** | **-1.08226800** | **-1.32321400** |
| **C** | **-3.10331100** | **0.49624000** | **-2.20375500** |
| **C** | **-4.20516600** | **1.18151700** | **-2.65410500** |
| **C** | **-5.45241400** | **0.51725300** | **-2.80715700** |
| **C** | **-5.57115300** | **-0.81661000** | **-2.50229900** |
| **H** | **-6.52409600** | **-1.32820300** | **-2.61635700** |
| **H** | **-6.31434400** | **1.07341800** | **-3.16591100** |
| **H** | **-4.12793400** | **2.23870100** | **-2.89178800** |
| **H** | **-2.15140100** | **1.00649700** | **-2.07998000** |
| **C** | **-0.80313100** | **0.00669700** | **2.35464900** |
| **H** | **-0.32601100** | **-0.10314200** | **1.37171200** |
| **H** | **-0.43113500** | **0.93290400** | **2.80439600** |
| **H** | **-0.44453400** | **-0.82491900** | **2.96791500** |

Methoxy Functionalized Expanded [14]-helicene:

X Y Z

**-----------------------------------------------------------------**

| **C** | **-3.18217900** | **-5.26006500** | **-1.22371000** |
| --- | --- | --- | --- |
| **C** | **-2.08602600** | **-5.91221600** | **-0.76811000** |
| **C** | **-0.84805500** | **-5.21415000** | **-0.52384000** |
| **C** | **-3.16719800** | **-3.83811800** | **-1.46481400** |
| **C** | **-1.96329700** | **-3.08438600** | **-1.20711000** |
| **C** | **-0.76761200** | **-3.80107900** | **-0.74295200** |
| **C** | **0.28988800** | **-5.90250200** | **-0.09858600** |
| **C** | **1.51735400** | **-5.26498900** | **0.11016600** |
| **C** | **1.61457500** | **-3.85169500** | **-0.10116000** |
| **C** | **0.46298800** | **-3.16905700** | **-0.52335100** |

| **C** | **2.68041400** | **-6.01667700** | **0.50186000** |
| --- | --- | --- | --- |
| **C** | **3.88786300** | **-5.41639000** | **0.64635900** |
| **C** | **4.04509000** | **-4.00078400** | **0.44042600** |
| **C** | **2.90892100** | **-3.19456000** | **0.10150800** |
| **C** | **3.10075600** | **-1.81180900** | **-0.03408100** |
| **C** | **5.30129400** | **-3.39728000** | **0.54836900** |
| **C** | **5.49049000** | **-2.02607500** | **0.35932800** |
| **C** | **4.35186700** | **-1.19461000** | **0.10136100** |
| **C** | **6.80961600** | **-1.44977500** | **0.38961300** |
| **C** | **7.00334100** | **-0.12871900** | **0.15206400** |
| **C** | **5.89231700** | **0.75948700** | **-0.07068400** |
| **C** | **4.55295600** | **0.25008800** | **-0.04025600** |
| **C** | **3.49748600** | **1.16604500** | **-0.14001300** |
| **C** | **3.69381800** | **2.54195100** | **-0.32036400** |
| **C** | **5.03703500** | **3.01881200** | **-0.46770000** |
| **C** | **6.09689700** | **2.12110500** | **-0.31085800** |
| **C** | **2.59394400** | **3.50722500** | **-0.37456400** |
| **C** | **2.88330700** | **4.86335400** | **-0.73481400** |
| **C** | **4.24750300** | **5.27383800** | **-0.94295100** |
| **C** | **5.27563800** | **4.40351300** | **-0.78209300** |
| **C** | **1.27008200** | **3.17458300** | **-0.06020900** |
| **C** | **0.21626500** | **4.09579700** | **-0.11722200** |
| **C** | **0.50526900** | **5.42345500** | **-0.57328300** |
| **C** | **1.82931300** | **5.77408400** | **-0.85272400** |
| **C** | **-1.15742600** | **3.76549100** | **0.27373300** |
| **C** | **-2.18089700** | **4.74720900** | **0.08373300** |
| **C** | **-1.84595400** | **6.04792800** | **-0.43562400** |
| **C** | **-0.56318400** | **6.37709400** | **-0.72813600** |
| **C** | **-1.50119200** | **2.53800400** | **0.85431500** |
| **C** | **-2.81171000** | **2.20373500** | **1.24655600** |
| **C** | **-3.83408300** | **3.18567900** | **0.99541200** |
| **C** | **-3.49085700** | **4.42076300** | **0.43410000** |
| **C** | **-3.19431300** | **0.93639300** | **1.88281500** |
| **C** | **-4.57669200** | **0.71455000** | **2.17961100** |
| **C** | **-5.55361900** | **1.72521600** | **1.87872600** |
| **C** | **-5.20181900** | **2.90917500** | **1.32374700** |
| **H** | **-4.10617600** | **-5.79987000** | **-1.41655700** |
| **H** | **-2.11265200** | **-6.98433000** | **-0.58913300** |
| **H** | **0.22513200** | **-6.97756100** | **0.05764100** |
| **H** | **0.53590500** | **-2.10695400** | **-0.72055100** |
| **H** | **2.57034800** | **-7.08670600** | **0.66011900** |
| **H** | **4.76618500** | **-5.99535600** | **0.92099200** |
| **H** | **2.24759000** | **-1.19284000** | **-0.28274900** |
| **H** | **6.16517800** | **-4.02093500** | **0.76973500** |
| **H** | **7.65303000** | **-2.10904400** | **0.57951000** |
| **H** | **8.00581300** | **0.29210700** | **0.14394800** |
| **H** | **2.48181300** | **0.79465200** | **-0.07900600** |
| **H** | **7.11664600** | **2.49497200** | **-0.37868100** |
| **H** | **4.43640100** | **6.31192600** | **-1.20548000** |
| **H** | **6.30464700** | **4.73089200** | **-0.90879200** |
| **H** | **1.05278800** | **2.16307400** | **0.25875800** |
| **H** | **2.04882600** | **6.79483000** | **-1.15977900** |
| **H** | **-2.64754200** | **6.77107300** | **-0.56608800** |
| **H** | **-0.31494100** | **7.37013200** | **-1.09491000** |
| **H** | **-0.72502900** | **1.81643500** | **1.03659200** |
| **H** | **-4.27716800** | **5.15681200** | **0.27757400** |
| **H** | **-6.59186000** | **1.51366000** | **2.12149500** |
| **H** | **-5.94828000** | **3.67114600** | **1.11298300** |
| **C** | **-5.01753400** | **-0.48263000** | **2.78366500** |
| **C** | **-4.11790600** | **-1.47244800** | **3.11174100** |
| **C** | **-2.75451700** | **-1.28591500** | **2.85091200** |
| **C** | **-2.29368500** | **-0.11525500** | **2.25511300** |
| **H** | **-6.07803000** | **-0.60552800** | **2.98564000** |

| **H** | **-4.45251800** | **-2.39725000** | **3.57327200** |
| --- | --- | --- | --- |
| **H** | **-2.05770900** | **-2.07204500** | **3.11195200** |
| **C** | **-1.99671100** | **-1.70894000** | **-1.41675700** |
| **C** | **-3.14527200** | **-1.04215600** | **-1.88998100** |
| **C** | **-4.33219600** | **-1.80262000** | **-2.16698200** |
| **C** | **-4.30489300** | **-3.18944300** | **-1.93584700** |
| **H** | **-5.20250700** | **-3.77395200** | **-2.12848900** |
| **H** | **-1.12355700** | **-1.09941100** | **-1.20606000** |
| **C** | **-3.16794100** | **0.36640000** | **-2.10393900** |
| **C** | **-4.29967900** | **0.98788300** | **-2.57255500** |
| **C** | **-5.47094100** | **0.23341700** | **-2.85388200** |
| **C** | **-5.48696900** | **-1.12539600** | **-2.65464400** |
| **H** | **-6.38236100** | **-1.70579000** | **-2.86494400** |
| **H** | **-6.35757900** | **0.74024600** | **-3.22537600** |
| **H** | **-4.30458600** | **2.06345700** | **-2.72448100** |
| **H** | **-2.27559500** | **0.94508400** | **-1.88029800** |
| **O** | **-0.95960400** | **0.07085600** | **2.01191500** |
| **C** | **-0.02294000** | **-0.86930300** | **2.52469300** |
| **H** | **-0.12886900** | **-1.84935300** | **2.04512200** |
| **H** | **0.96293200** | **-0.46186400** | **2.29244300** |
| **H** | **-0.12337400** | **-0.97797000** | **3.61150800** |

Amino Functionalized Expanded [14]-helicene:

X Y Z

**-----------------------------------------------------------------**

| **C** | **-4.04385000** | **-4.66598200** | **-0.91161000** |
| --- | --- | --- | --- |
| **C** | **-3.06621000** | **-5.46525100** | **-0.42176900** |
| **C** | **-1.72350300** | **-4.97452200** | **-0.23203700** |
| **C** | **-3.78934000** | **-3.28854300** | **-1.25523500** |
| **C** | **-2.46973300** | **-2.73728100** | **-1.05888100** |
| **C** | **-1.40955900** | **-3.61046300** | **-0.53661800** |
| **C** | **-0.71019600** | **-5.82140200** | **0.22140000** |
| **C** | **0.61175600** | **-5.39132700** | **0.37558300** |
| **C** | **0.93805600** | **-4.02488400** | **0.09642800** |
| **C** | **-0.09042300** | **-3.17894600** | **-0.34669500** |
| **C** | **1.64414700** | **-6.31232700** | **0.77215800** |
| **C** | **2.94116000** | **-5.92300500** | **0.84568100** |
| **C** | **3.32744300** | **-4.56470700** | **0.56814800** |
| **C** | **2.32994500** | **-3.58835100** | **0.24013600** |
| **C** | **2.74440000** | **-2.26205400** | **0.05104800** |
| **C** | **4.67166000** | **-4.18242800** | **0.59730700** |
| **C** | **5.08050500** | **-2.87078300** | **0.34404300** |
| **C** | **4.08718100** | **-1.86454600** | **0.10964600** |
| **C** | **6.47791400** | **-2.52774200** | **0.29050500** |
| **C** | **6.88037600** | **-1.26482100** | **0.00344800** |
| **C** | **5.92570500** | **-0.20419100** | **-0.18796000** |
| **C** | **4.52280600** | **-0.47886900** | **-0.08407200** |
| **C** | **3.63274300** | **0.60026800** | **-0.15591900** |
| **C** | **4.04946600** | **1.92229900** | **-0.36283300** |
| **C** | **5.44609800** | **2.16622900** | **-0.56930700** |
| **C** | **6.34510400** | **1.10166200** | **-0.45726000** |
| **C** | **3.12800900** | **3.06070300** | **-0.36504600** |
| **C** | **3.63414200** | **4.36194100** | **-0.68729400** |
| **C** | **5.03709900** | **4.53679600** | **-0.95986200** |
| **C** | **5.90343400** | **3.49594600** | **-0.87959200** |
| **C** | **1.77540700** | **2.94440700** | **-0.02284000** |
| **C** | **0.89697600** | **4.03502900** | **0.00996200** |
| **C** | **1.40657600** | **5.32415300** | **-0.35300100** |
| **C** | **2.75699100** | **5.45062200** | **-0.69282300** |
| **C** | **-0.50086100** | **3.91958200** | **0.43254400** |
| **C** | **-1.31579000** | **5.09464800** | **0.45364800** |
| **C** | **-0.76156200** | **6.36147700** | **0.05197100** |

| **C** | **0.53502200** | **6.47073300** | **-0.33210600** |
| --- | --- | --- | --- |
| **C** | **-1.06625600** | **2.70533900** | **0.84417700** |
| **C** | **-2.38890300** | **2.57928500** | **1.30661500** |
| **C** | **-3.20876700** | **3.76110000** | **1.25916900** |
| **C** | **-2.64954900** | **4.97630200** | **0.85042600** |
| **C** | **-2.98103200** | **1.32064700** | **1.77403500** |
| **C** | **-4.39595200** | **1.28323000** | **1.99404800** |
| **C** | **-5.17280700** | **2.48994400** | **1.89273500** |
| **C** | **-4.60471500** | **3.67960800** | **1.58030600** |
| **H** | **-5.04982700** | **-5.05103600** | **-1.06071300** |
| **H** | **-3.27138800** | **-6.50341600** | **-0.17195100** |
| **H** | **-0.95168300** | **-6.86035400** | **0.43747300** |
| **H** | **0.15288200** | **-2.15167800** | **-0.58596500** |
| **H** | **1.36051700** | **-7.34003600** | **0.98564000** |
| **H** | **3.71919200** | **-6.63234000** | **1.11704400** |
| **H** | **1.99419400** | **-1.51282900** | **-0.17008600** |
| **H** | **5.42711800** | **-4.93697200** | **0.80762300** |
| **H** | **7.20629700** | **-3.31695500** | **0.45999900** |
| **H** | **7.93788900** | **-1.02176300** | **-0.06520200** |
| **H** | **2.57462300** | **0.40410500** | **-0.03471000** |
| **H** | **7.40915600** | **1.29811100** | **-0.57340600** |
| **H** | **5.39168500** | **5.53491900** | **-1.20526800** |
| **H** | **6.96586600** | **3.64432600** | **-1.05672300** |
| **H** | **1.39970000** | **1.97035500** | **0.26307900** |
| **H** | **3.14458300** | **6.43559300** | **-0.94578800** |
| **H** | **-1.40957800** | **7.23451700** | **0.06950000** |
| **H** | **0.94710900** | **7.43344200** | **-0.62471000** |
| **H** | **-0.47341100** | **1.80924700** | **0.75809600** |
| **H** | **-3.28475500** | **5.85983600** | **0.82882000** |
| **H** | **-6.23882300** | **2.42307900** | **2.09537000** |
| **H** | **-5.20059100** | **4.58778400** | **1.53188900** |
| **C** | **-5.05154600** | **0.08339500** | **2.33449900** |
| **C** | **-4.33112100** | **-1.08482500** | **2.48965400** |
| **C** | **-2.94053400** | **-1.05624300** | **2.36703900** |
| **C** | **-2.25410800** | **0.11549700** | **2.04168400** |
| **H** | **-6.12916600** | **0.09576400** | **2.47313700** |
| **H** | **-4.83073300** | **-2.01651000** | **2.73952400** |
| **H** | **-2.36628600** | **-1.96382600** | **2.53941300** |
| **C** | **-2.26527200** | **-1.40029100** | **-1.38609500** |
| **C** | **-3.28863100** | **-0.58349400** | **-1.90815900** |
| **C** | **-4.59539400** | **-1.14306800** | **-2.11517500** |
| **C** | **-4.80548700** | **-2.49065400** | **-1.77294500** |
| **H** | **-5.79341500** | **-2.92381100** | **-1.91760300** |
| **H** | **-1.29502200** | **-0.93962200** | **-1.23165600** |
| **C** | **-3.06862000** | **0.78474100** | **-2.24001300** |
| **C** | **-4.08245200** | **1.55532300** | **-2.75473100** |
| **C** | **-5.37321000** | **0.99861900** | **-2.96555600** |
| **C** | **-5.62293800** | **-0.31511900** | **-2.65242600** |
| **H** | **-6.61003600** | **-0.74397900** | **-2.80931200** |
| **H** | **-6.16428400** | **1.62145100** | **-3.37460900** |
| **H** | **-3.90201700** | **2.59818300** | **-3.00004000** |
| **H** | **-2.08349600** | **1.21212900** | **-2.07132800** |
| **N** | **-0.84365100** | **0.04709900** | **1.97294900** |
| **H** | **-0.38387800** | **0.82601900** | **2.43621800** |
| **H** | **-0.49765800** | **-0.82386900** | **2.36452600** |

Carboxyl Functionalized Expanded [14]-helicene:

X Y Z

----------------------------------------------------------------- C -3.83744000 -4.97703000 -1.00617100

C -2.81998600 -5.72638100 -0.51829800

C -1.50987000 -5.16280400 -0.30446200

| **C** | **-3.66009600** | **-3.58009900** | **-1.31924500** |
| --- | --- | --- | --- |
| **C** | **-2.37633400** | **-2.95784500** | **-1.09660700** |
| **C** | **-1.27040300** | **-3.77932200** | **-0.58555800** |
| **C** | **-0.45551100** | **-5.95613500** | **0.15114300** |
| **C** | **0.83720800** | **-5.45180400** | **0.32668000** |
| **C** | **1.09036700** | **-4.06645000** | **0.06229800** |
| **C** | **0.02101600** | **-3.27568800** | **-0.38396200** |
| **C** | **1.91231500** | **-6.31781900** | **0.73184400** |
| **C** | **3.18406000** | **-5.85743700** | **0.82436900** |
| **C** | **3.49767600** | **-4.47904600** | **0.55511900** |
| **C** | **2.45514400** | **-3.55313300** | **0.22140700** |
| **C** | **2.80261400** | **-2.20450900** | **0.03720100** |
| **C** | **4.81985200** | **-4.03055600** | **0.59511600** |
| **C** | **5.16485700** | **-2.70274000** | **0.33661400** |
| **C** | **4.12656400** | **-1.74319900** | **0.09322700** |
| **C** | **6.54717300** | **-2.30631400** | **0.28121900** |
| **C** | **6.89828600** | **-1.03521300** | **-0.02988500** |
| **C** | **5.89975100** | **-0.02069700** | **-0.24190900** |
| **C** | **4.50637700** | **-0.34099000** | **-0.12285600** |
| **C** | **3.57997800** | **0.70985700** | **-0.21353300** |
| **C** | **3.95586600** | **2.03987400** | **-0.46582300** |
| **C** | **5.34357200** | **2.31863100** | **-0.69111900** |
| **C** | **6.27540900** | **1.28827700** | **-0.55044500** |
| **C** | **3.00794200** | **3.15909000** | **-0.50239200** |
| **C** | **3.48279900** | **4.46108900** | **-0.87310600** |
| **C** | **4.87803000** | **4.66283300** | **-1.16221600** |
| **C** | **5.76779800** | **3.64634800** | **-1.05060600** |
| **C** | **1.65868000** | **3.03022700** | **-0.14907300** |
| **C** | **0.76044900** | **4.10704100** | **-0.15159400** |
| **C** | **1.24117400** | **5.39334100** | **-0.55541200** |
| **C** | **2.58586400** | **5.53292700** | **-0.90839100** |
| **C** | **-0.63328200** | **3.98408200** | **0.27906000** |
| **C** | **-1.47317600** | **5.14453800** | **0.28383900** |
| **C** | **-0.94205900** | **6.40871600** | **-0.15666200** |
| **C** | **0.34873400** | **6.52462900** | **-0.55821600** |
| **C** | **-1.18035100** | **2.77157000** | **0.70517600** |
| **C** | **-2.48887500** | **2.64168800** | **1.18817900** |
| **C** | **-3.33765800** | **3.79599800** | **1.12966100** |
| **C** | **-2.80370900** | **5.01399800** | **0.69102000** |
| **C** | **-3.03259900** | **1.37989600** | **1.68681100** |
| **C** | **-4.44004000** | **1.28850800** | **1.90416300** |
| **C** | **-5.26005500** | **2.46567200** | **1.78989400** |
| **C** | **-4.72929900** | **3.67081300** | **1.46150200** |
| **H** | **-4.81739900** | **-5.41728700** | **-1.17493700** |
| **H** | **-2.96742200** | **-6.77907800** | **-0.28891500** |
| **H** | **-0.63895500** | **-7.00977400** | **0.35289900** |
| **H** | **0.20594600** | **-2.23278500** | **-0.59635600** |
| **H** | **1.68273300** | **-7.36079700** | **0.93638200** |
| **H** | **3.99751800** | **-6.52325600** | **1.10227600** |
| **H** | **2.01425400** | **-1.49278000** | **-0.17059700** |
| **H** | **5.61083800** | **-4.74609100** | **0.81117900** |
| **H** | **7.30538100** | **-3.06381600** | **0.46427600** |
| **H** | **7.94437700** | **-0.74890600** | **-0.10589700** |
| **H** | **2.53041000** | **0.48156600** | **-0.06839300** |
| **H** | **7.33209600** | **1.51457600** | **-0.67867400** |
| **H** | **5.20552600** | **5.66026800** | **-1.44482100** |
| **H** | **6.82569900** | **3.81158800** | **-1.23955100** |
| **H** | **1.30724600** | **2.05942700** | **0.17709300** |
| **H** | **2.95518900** | **6.51570300** | **-1.19474000** |
| **H** | **-1.60206200** | **7.27269800** | **-0.15620600** |
| **H** | **0.74213900** | **7.48507500** | **-0.88232800** |
| **H** | **-0.57185300** | **1.88701500** | **0.61128000** |
| **H** | **-3.45344300** | **5.88606100** | **0.65200700** |

| **H** | **-6.32309000** | **2.36703500** | **1.99465800** |
| --- | --- | --- | --- |
| **H** | **-5.35637500** | **4.55708100** | **1.40467800** |
| **C** | **-5.02545900** | **0.05335300** | **2.25637900** |
| **C** | **-4.25713500** | **-1.08480400** | **2.40192400** |
| **C** | **-2.86550200** | **-0.99664500** | **2.25861400** |
| **C** | **-2.25706200** | **0.21440300** | **1.94770000** |
| **H** | **-6.10166800** | **0.01259300** | **2.40509100** |
| **H** | **-4.71691100** | **-2.03649300** | **2.65014100** |
| **H** | **-2.24586200** | **-1.87420600** | **2.41600900** |
| **C** | **-2.24767400** | **-1.60249000** | **-1.38649900** |
| **C** | **-3.31219600** | **-0.83578400** | **-1.90305700** |
| **C** | **-4.58218400** | **-1.46511500** | **-2.13787000** |
| **C** | **-4.71671800** | **-2.83034700** | **-1.82834600** |
| **H** | **-5.67653400** | **-3.31659300** | **-1.99294000** |
| **H** | **-1.31080800** | **-1.09039700** | **-1.19587800** |
| **C** | **-3.16995300** | **0.55047600** | **-2.20191200** |
| **C** | **-4.22196400** | **1.27346500** | **-2.70952100** |
| **C** | **-5.47625300** | **0.64805200** | **-2.94629800** |
| **C** | **-5.65119400** | **-0.68531600** | **-2.66660000** |
| **H** | **-6.60972300** | **-1.16735000** | **-2.84538200** |
| **H** | **-6.29840100** | **1.23297400** | **-3.35014900** |
| **H** | **-4.09939200** | **2.33001700** | **-2.93112100** |
| **H** | **-2.21138200** | **1.02925200** | **-2.01802800** |
| **C** | **-0.75933600** | **0.19684500** | **2.00162700** |
| **O** | **-0.02598700** | **-0.40159900** | **1.23510000** |
| **O** | **-0.28453000** | **0.88279900** | **3.06253300** |
| **H** | **0.68898100** | **0.80393500** | **3.03104900** |

Bor Doped Expanded [14]-helicene:

X Y Z

**-----------------------------------------------------------------**

| **C** | **-1.57766000** | **-6.16746400** | **-0.64306100** |
| --- | --- | --- | --- |
| **C** | **-0.32634100** | **-6.47115400** | **-0.22286700** |
| **C** | **0.69193200** | **-5.45655700** | **-0.10737900** |
| **C** | **-1.94555300** | **-4.81958200** | **-1.00150200** |
| **C** | **-0.96873900** | **-3.76205700** | **-0.89323800** |
| **C** | **0.38194900** | **-4.09331700** | **-0.41915100** |
| **C** | **1.99011100** | **-5.78710300** | **0.28684100** |
| **C** | **3.00624500** | **-4.83156500** | **0.39310500** |
| **C** | **2.69955900** | **-3.45777300** | **0.13073100** |
| **C** | **1.39481500** | **-3.13810800** | **-0.27305400** |
| **C** | **4.35187800** | **-5.21636000** | **0.72770900** |
| **C** | **5.35352400** | **-4.30215300** | **0.76660100** |
| **C** | **5.10093600** | **-2.90628300** | **0.52460500** |
| **C** | **3.76373000** | **-2.45948800** | **0.25532600** |
| **C** | **3.54906400** | **-1.08208500** | **0.11401300** |
| **C** | **6.14590000** | **-1.97534200** | **0.54726400** |
| **C** | **5.92989200** | **-0.61060100** | **0.34527900** |
| **C** | **4.58944800** | **-0.14521600** | **0.16251400** |
| **C** | **7.01360400** | **0.33719500** | **0.30512400** |
| **C** | **6.79821900** | **1.66030800** | **0.08819300** |
| **C** | **5.47035700** | **2.20680400** | **-0.06428900** |
| **C** | **4.35574600** | **1.27171000** | **0.01301700** |
| **C** | **2.77785500** | **3.49748400** | **-0.23123500** |
| **C** | **4.03396700** | **4.22166400** | **-0.36988900** |
| **C** | **5.28674900** | **3.58766500** | **-0.26694600** |
| **C** | **1.51511200** | **4.19521700** | **-0.27492500** |
| **C** | **1.50592800** | **5.59626000** | **-0.56167800** |
| **C** | **2.75884900** | **6.28373700** | **-0.73931700** |
| **C** | **3.94951900** | **5.64002700** | **-0.62661600** |
| **C** | **0.29826900** | **3.54871300** | **-0.02162100** |
| **C** | **-0.93289900** | **4.21528100** | **-0.04917800** |

| **C** | **-0.93732000** | **5.60289900** | **-0.41447800** |
| --- | --- | --- | --- |
| **C** | **0.27775200** | **6.25653400** | **-0.64806400** |
| **C** | **-2.20477100** | **3.56948200** | **0.28709900** |
| **C** | **-3.42042300** | **4.31640200** | **0.14084500** |
| **C** | **-3.37067000** | **5.68948600** | **-0.29082000** |
| **C** | **-2.18798000** | **6.30677800** | **-0.53425400** |
| **C** | **-2.28823800** | **2.26223500** | **0.77987800** |
| **C** | **-3.50154200** | **1.64680000** | **1.12235700** |
| **C** | **-4.71160800** | **2.39128500** | **0.93214900** |
| **C** | **-4.63606700** | **3.70319600** | **0.45010900** |
| **C** | **-3.58184000** | **0.29414400** | **1.67087100** |
| **C** | **-4.86304600** | **-0.25313300** | **1.97683000** |
| **C** | **-6.04818500** | **0.52902500** | **1.74859600** |
| **C** | **-5.97712700** | **1.79140000** | **1.25389700** |
| **H** | **-2.33543100** | **-6.94255700** | **-0.72889400** |
| **H** | **-0.06045800** | **-7.49385800** | **0.03320400** |
| **H** | **2.22512600** | **-6.82812700** | **0.49974300** |
| **H** | **1.16907300** | **-2.10637700** | **-0.51174700** |
| **H** | **4.55206600** | **-6.26696900** | **0.92307900** |
| **H** | **6.37286200** | **-4.60661600** | **0.99114800** |
| **H** | **2.54206200** | **-0.70853000** | **-0.04898500** |
| **H** | **7.16018100** | **-2.32953900** | **0.72072400** |
| **H** | **8.02584300** | **-0.03785200** | **0.43585800** |
| **H** | **7.63543000** | **2.35241900** | **0.03923500** |
| **H** | **6.17482800** | **4.20851600** | **-0.34703200** |
| **H** | **2.73172100** | **7.35016700** | **-0.94999500** |
| **H** | **4.88242500** | **6.18664300** | **-0.74173500** |
| **H** | **0.33164100** | **2.48895100** | **0.21472600** |
| **H** | **0.26426900** | **7.31603300** | **-0.89629300** |
| **H** | **-4.30897100** | **6.22902800** | **-0.39373000** |
| **H** | **-2.16020200** | **7.35159000** | **-0.83392900** |
| **H** | **-1.37013200** | **1.70845500** | **0.92913800** |
| **H** | **-5.55660600** | **4.27089500** | **0.32876400** |
| **H** | **-7.01065000** | **0.08389600** | **1.98933500** |
| **H** | **-6.87953500** | **2.37647900** | **1.09377900** |
| **C** | **-4.95715500** | **-1.55858300** | **2.50737100** |
| **C** | **-3.82652600** | **-2.31902900** | **2.73925200** |
| **C** | **-2.56074500** | **-1.78420200** | **2.44481600** |
| **C** | **-2.44594700** | **-0.50650700** | **1.92397600** |
| **H** | **-5.94331900** | **-1.95838200** | **2.73210500** |
| **H** | **-3.91367400** | **-3.32279900** | **3.14551400** |
| **H** | **-1.66661700** | **-2.37533000** | **2.62101700** |
| **H** | **-1.45357200** | **-0.12319400** | **1.71188500** |
| **C** | **-1.35623500** | **-2.47688300** | **-1.26075400** |
| **C** | **-2.65234500** | **-2.17870600** | **-1.72797700** |
| **C** | **-3.61856800** | **-3.23647400** | **-1.83262100** |
| **C** | **-3.22866400** | **-4.53517700** | **-1.45983700** |
| **H** | **-3.95157000** | **-5.34508100** | **-1.53694100** |
| **H** | **-0.65065700** | **-1.65388000** | **-1.20168300** |
| **C** | **-3.03858600** | **-0.85905700** | **-2.10152100** |
| **C** | **-4.30841800** | **-0.59935700** | **-2.55608100** |
| **C** | **-5.26216000** | **-1.64770600** | **-2.66336800** |
| **C** | **-4.92585200** | **-2.93158100** | **-2.31008100** |
| **H** | **-5.65318500** | **-3.73628700** | **-2.38901700** |
| **H** | **-6.26243800** | **-1.42594000** | **-3.02546700** |
| **H** | **-4.59091000** | **0.41264900** | **-2.83202200** |
| **H** | **-2.31141900** | **-0.05547700** | **-2.01392200** |
| **B** | **3.20672200** | **2.13246000** | **-0.07865000** |

Nitrogene Doped Expanded [14]-helicene:

X Y Z

**-----------------------------------------------------------------**

| **C** | **-4.00968100** | **-4.27797700** | **-1.20903200** |
| --- | --- | --- | --- |
| **C** | **-3.05164000** | **-5.11455500** | **-0.74331900** |
| **C** | **-1.70313800** | **-4.65642200** | **-0.51663700** |
| **C** | **-3.72588200** | **-2.89365700** | **-1.49796200** |
| **C** | **-2.39709300** | **-2.37732300** | **-1.27230800** |
| **C** | **-1.36209700** | **-3.28731400** | **-0.76388900** |
| **C** | **-0.71159500** | **-5.53770300** | **-0.07952400** |
| **C** | **0.61217600** | **-5.13295000** | **0.12198800** |
| **C** | **0.96023100** | **-3.76052100** | **-0.08829900** |
| **C** | **-0.04253600** | **-2.88362600** | **-0.52727500** |
| **C** | **1.62789100** | **-6.08057000** | **0.49916000** |
| **C** | **2.92694700** | **-5.70877000** | **0.62103200** |
| **C** | **3.33365800** | **-4.34219700** | **0.42136200** |
| **C** | **2.34874100** | **-3.34309600** | **0.11692600** |
| **C** | **2.77156400** | **-2.01171500** | **0.01447800** |
| **C** | **4.68131000** | **-3.97643500** | **0.51108100** |
| **C** | **5.10444700** | **-2.65592400** | **0.33498300** |
| **C** | **4.11600800** | **-1.64440800** | **0.12295100** |
| **C** | **6.50057000** | **-2.30036700** | **0.34174700** |
| **C** | **6.90372000** | **-1.02239200** | **0.12148900** |
| **C** | **5.94545800** | **0.03486100** | **-0.06837800** |
| **C** | **4.54348000** | **-0.25422600** | **-0.00837200** |
| **C** | **3.98502400** | **1.98304200** | **-0.24947800** |
| **C** | **5.35446700** | **2.36008600** | **-0.43544200** |
| **C** | **6.31950100** | **1.35793000** | **-0.31285300** |
| **C** | **2.96293400** | **3.02535700** | **-0.24072600** |
| **C** | **3.33905500** | **4.36868900** | **-0.55320700** |
| **C** | **4.72140500** | **4.68139200** | **-0.81125400** |
| **C** | **5.68664600** | **3.72931900** | **-0.72980800** |
| **C** | **1.63640800** | **2.74689100** | **0.10231700** |
| **C** | **0.65066100** | **3.73983000** | **0.14777500** |
| **C** | **1.01550000** | **5.07146800** | **-0.24411000** |
| **C** | **2.34524300** | **5.35219000** | **-0.57583400** |
| **C** | **-0.72237000** | **3.48400000** | **0.59242000** |
| **C** | **-1.67810300** | **4.55168900** | **0.53874400** |
| **C** | **-1.27108900** | **5.85355800** | **0.07556300** |
| **C** | **0.01300700** | **6.10561300** | **-0.28038900** |
| **C** | **-1.13752200** | **2.25003700** | **1.10565700** |
| **C** | **-2.44272200** | **2.00966700** | **1.56067500** |
| **C** | **-3.39404500** | **3.07848400** | **1.47725600** |
| **C** | **-2.98552400** | **4.31676900** | **0.96825300** |
| **C** | **-2.86525300** | **0.72841700** | **2.12318500** |
| **C** | **-4.21277200** | **0.57875300** | **2.56647300** |
| **C** | **-5.13040700** | **1.68018200** | **2.45149900** |
| **C** | **-4.74133100** | **2.87260000** | **1.93198700** |
| **H** | **-5.02131400** | **-4.63752700** | **-1.38140200** |
| **H** | **-3.27891900** | **-6.15764200** | **-0.53688700** |
| **H** | **-0.97276800** | **-6.58084200** | **0.08805600** |
| **H** | **0.22756900** | **-1.85386300** | **-0.72347900** |
| **H** | **1.33108900** | **-7.11449300** | **0.65809700** |
| **H** | **3.69055000** | **-6.43928600** | **0.87672300** |
| **H** | **2.06207800** | **-1.21405500** | **-0.16792600** |
| **H** | **5.42287300** | **-4.74794900** | **0.70845500** |
| **H** | **7.23096700** | **-3.08952900** | **0.50230500** |
| **H** | **7.96108900** | **-0.76980100** | **0.09751300** |
| **H** | **7.37409100** | **1.61118300** | **-0.40617600** |
| **H** | **4.98013600** | **5.71053800** | **-1.04783500** |
| **H** | **6.73129000** | **3.98203300** | **-0.89494300** |
| **H** | **1.40471800** | **1.71882000** | **0.35152600** |
| **H** | **2.61612100** | **6.37187900** | **-0.84229200** |
| **H** | **-2.01948000** | **6.64168400** | **0.04062000** |
| **H** | **0.31316300** | **7.09937100** | **-0.60370000** |
| **H** | **-0.40900800** | **1.45299100** | **1.17905600** |

| **H** | **-3.70828200** | **5.12913000** | **0.92240100** |
| --- | --- | --- | --- |
| **H** | **-6.15189200** | **1.53471800** | **2.79485300** |
| **H** | **-5.44332800** | **3.69910300** | **1.85269400** |
| **C** | **-4.63521200** | **-0.65165300** | **3.11641700** |
| **C** | **-3.76429100** | **-1.71864400** | **3.23249400** |
| **C** | **-2.43561200** | **-1.57829100** | **2.79644300** |
| **C** | **-2.00002300** | **-0.38041400** | **2.25584600** |
| **H** | **-5.66665300** | **-0.74584700** | **3.44842400** |
| **H** | **-4.10350300** | **-2.66030800** | **3.65516200** |
| **H** | **-1.74525000** | **-2.41303200** | **2.87648100** |
| **H** | **-0.96845200** | **-0.30814300** | **1.92890500** |
| **C** | **-2.16007100** | **-1.03669200** | **-1.56029300** |
| **C** | **-3.16097300** | **-0.18072900** | **-2.06284600** |
| **C** | **-4.47910800** | **-0.70374900** | **-2.29223300** |
| **C** | **-4.72055000** | **-2.05715600** | **-1.99609000** |
| **H** | **-5.71617000** | **-2.46393500** | **-2.16270900** |
| **H** | **-1.17566900** | **-0.60713200** | **-1.40358600** |
| **C** | **-2.90737200** | **1.19126400** | **-2.35284000** |
| **C** | **-3.90105900** | **2.00038600** | **-2.84738500** |
| **C** | **-5.20357900** | **1.48060600** | **-3.07940200** |
| **C** | **-5.48488600** | **0.16401100** | **-2.80761800** |
| **H** | **-6.48089900** | **-0.23680900** | **-2.98125500** |
| **H** | **-5.97839700** | **2.13364700** | **-3.47219100** |
| **H** | **-3.69510300** | **3.04559800** | **-3.06063600** |
| **H** | **-1.91317200** | **1.58987500** | **-2.16772000** |
| **N** | **3.61306100** | **0.70770200** | **-0.07270200** |

Silicone Doped Expanded [14]-helicene:

X Y Z

**-----------------------------------------------------------------**

| **C** | **-4.91569000** | **4.92527900** | **-0.30451100** |
| --- | --- | --- | --- |
| **C** | **-5.82593700** | **3.99852300** | **0.07878900** |
| **C** | **-5.49215000** | **2.59656700** | **0.12892100** |
| **C** | **-3.57751300** | **4.55082400** | **-0.69140200** |
| **C** | **-3.18662800** | **3.16146200** | **-0.65130100** |
| **C** | **-4.17074400** | **2.16280800** | **-0.21228300** |
| **C** | **-6.44776100** | **1.64599600** | **0.49350500** |
| **C** | **-6.16039200** | **0.27811000** | **0.54630200** |
| **C** | **-4.83099200** | **-0.16630100** | **0.25308900** |
| **C** | **-3.88262500** | **0.79574000** | **-0.12388000** |
| **C** | **-7.18571500** | **-0.68005600** | **0.86547900** |
| **C** | **-6.92424300** | **-2.01112400** | **0.87218200** |
| **C** | **-5.60260400** | **-2.51251000** | **0.60423100** |
| **C** | **-4.53083400** | **-1.59842800** | **0.33291200** |
| **C** | **-3.24583500** | **-2.12587500** | **0.15393400** |
| **C** | **-5.34203100** | **-3.88593600** | **0.60354800** |
| **C** | **-4.06346900** | **-4.40205600** | **0.38020900** |
| **C** | **-2.96650200** | **-3.50067100** | **0.18676200** |
| **C** | **-3.82712800** | **-5.81933900** | **0.32942400** |
| **C** | **-2.58877700** | **-6.31843800** | **0.09658100** |
| **C** | **-1.43288800** | **-5.46205200** | **-0.05858700** |
| **C** | **-1.61947000** | **-4.03787500** | **0.02288200** |
| **C** | **1.35093700** | **-4.10216500** | **-0.25887300** |
| **C** | **1.08331200** | **-5.50888200** | **-0.39546300** |
| **C** | **-0.19274000** | **-6.08936000** | **-0.27638300** |
| **C** | **2.72894900** | **-3.62692000** | **-0.32231700** |
| **C** | **3.78019800** | **-4.55297800** | **-0.62055700** |
| **C** | **3.46292600** | **-5.94416300** | **-0.79568100** |
| **C** | **2.19198900** | **-6.39770300** | **-0.66758800** |
| **C** | **3.08153900** | **-2.28949300** | **-0.08475100** |
| **C** | **4.39847900** | **-1.81926400** | **-0.14483400** |
| **C** | **5.43042700** | **-2.74899000** | **-0.50158200** |

| **C** | **5.09427400** | **-4.08795600** | **-0.71944100** |
| --- | --- | --- | --- |
| **C** | **4.76955500** | **-0.43061500** | **0.14677400** |
| **C** | **6.14098800** | **-0.03575100** | **0.00172300** |
| **C** | **7.12877000** | **-1.00464900** | **-0.39832400** |
| **C** | **6.79237600** | **-2.29852800** | **-0.62480000** |
| **C** | **3.84970200** | **0.53012700** | **0.58085200** |
| **C** | **4.20292100** | **1.85655000** | **0.87041700** |
| **C** | **5.57214400** | **2.24273400** | **0.69697100** |
| **C** | **6.50051300** | **1.28636100** | **0.26985800** |
| **C** | **3.24145300** | **2.85138200** | **1.34253700** |
| **C** | **3.69164400** | **4.18007600** | **1.60152000** |
| **C** | **5.07512700** | **4.51854300** | **1.40083300** |
| **C** | **5.97319100** | **3.59499200** | **0.97164600** |
| **H** | **-5.18033500** | **5.97932900** | **-0.33928200** |
| **H** | **-6.83476800** | **4.29413200** | **0.35632200** |
| **H** | **-7.45607400** | **1.97935700** | **0.73104100** |
| **H** | **-2.88470700** | **0.46226400** | **-0.37922200** |
| **H** | **-8.18493700** | **-0.30985000** | **1.08117700** |
| **H** | **-7.71046500** | **-2.72955000** | **1.09097100** |
| **H** | **-2.42180100** | **-1.44324300** | **-0.02140600** |
| **H** | **-6.16438300** | **-4.57691900** | **0.77878600** |
| **H** | **-4.67447900** | **-6.48716100** | **0.46573300** |
| **H** | **-2.43353900** | **-7.39288600** | **0.03956700** |
| **H** | **-0.06468800** | **-1.62205500** | **0.07086300** |
| **H** | **-0.22397500** | **-7.17351800** | **-0.35911500** |
| **H** | **4.27474000** | **-6.63316900** | **-1.01663400** |
| **H** | **1.97500400** | **-7.45689700** | **-0.78090500** |
| **H** | **2.28975300** | **-1.59296800** | **0.16684700** |
| **H** | **5.88423400** | **-4.79471500** | **-0.96625300** |
| **H** | **8.15944400** | **-0.67377700** | **-0.49994600** |
| **H** | **7.54848600** | **-3.02596200** | **-0.90998100** |
| **H** | **2.81751300** | **0.23305600** | **0.71447600** |
| **H** | **7.54132100** | **1.58175800** | **0.15247000** |
| **H** | **5.38852800** | **5.53933300** | **1.60642100** |
| **H** | **7.01763700** | **3.86122100** | **0.82844000** |
| **C** | **2.77420700** | **5.15292500** | **2.05638200** |
| **C** | **1.44281800** | **4.84122100** | **2.25722000** |
| **C** | **0.99316900** | **3.53340200** | **2.00733100** |
| **C** | **1.87561300** | **2.56410400** | **1.56153000** |
| **H** | **3.13691400** | **6.16040400** | **2.24703700** |
| **H** | **0.74733000** | **5.60025500** | **2.60394800** |
| **H** | **-0.05258600** | **3.28094000** | **2.15756900** |
| **H** | **1.49604100** | **1.56388200** | **1.38167200** |
| **C** | **-1.89328600** | **2.83648200** | **-1.05075700** |
| **C** | **-0.96713400** | **3.80564900** | **-1.48766000** |
| **C** | **-1.36219300** | **5.18654900** | **-1.51946200** |
| **C** | **-2.66784300** | **5.51598300** | **-1.11401400** |
| **H** | **-2.98005700** | **6.55829800** | **-1.13858700** |
| **H** | **-1.56236000** | **1.80235200** | **-1.04659200** |
| **C** | **0.35156700** | **3.46142700** | **-1.90365300** |
| **C** | **1.23156700** | **4.42736200** | **-2.32626400** |
| **C** | **0.83943700** | **5.79324300** | **-2.35660800** |
| **C** | **-0.42360900** | **6.16237700** | **-1.96349900** |
| **H** | **-0.72782100** | **7.20628000** | **-1.98619700** |
| **H** | **1.54765300** | **6.54552300** | **-2.69314600** |
| **H** | **2.23543000** | **4.15116400** | **-2.63610200** |
| **H** | **0.65211800** | **2.41684100** | **-1.87786300** |
| **Si** | **-0.10668100** | **-3.09257300** | **-0.04723700** |

Phosphore Doped Expanded [14]-helicene:

X Y Z

**-----------------------------------------------------------------**

| **C** | **-3.45328100** | **5.74193500** | **-0.55431500** |
| --- | --- | --- | --- |
| **C** | **-4.55042100** | **5.06674100** | **-0.13581700** |
| **C** | **-4.54734400** | **3.62907800** | **-0.02329300** |
| **C** | **-2.23624600** | **5.05476000** | **-0.91109300** |
| **C** | **-2.17335800** | **3.61636200** | **-0.80465200** |
| **C** | **-3.36223100** | **2.88967300** | **-0.33895100** |
| **C** | **-5.69410000** | **2.93936300** | **0.37639000** |
| **C** | **-5.72893800** | **1.54510000** | **0.48248400** |
| **C** | **-4.54171400** | **0.79534300** | **0.20364300** |
| **C** | **-3.39762900** | **1.49697600** | **-0.20229300** |
| **C** | **-6.94324200** | **0.86080000** | **0.84211300** |
| **C** | **-6.99718900** | **-0.49350000** | **0.89978300** |
| **C** | **-5.83159600** | **-1.29640000** | **0.63980400** |
| **C** | **-4.58246500** | **-0.66417100** | **0.32724700** |
| **C** | **-3.45820300** | **-1.47790300** | **0.14722700** |
| **C** | **-5.89555100** | **-2.69173200** | **0.68042300** |
| **C** | **-4.77560700** | **-3.49668700** | **0.45411200** |
| **C** | **-3.50424600** | **-2.87892900** | **0.21745600** |
| **C** | **-4.88341400** | **-4.92995500** | **0.43701100** |
| **C** | **-3.80335000** | **-5.70777100** | **0.18408000** |
| **C** | **-2.49093600** | **-5.13871300** | **-0.01872400** |
| **C** | **-2.31605500** | **-3.72071300** | **0.04468900** |
| **C** | **0.37524100** | **-4.30902500** | **-0.27721600** |
| **C** | **-0.07992300** | **-5.65555400** | **-0.43430400** |
| **C** | **-1.42543200** | **-6.00894700** | **-0.27731300** |
| **C** | **1.81615700** | **-4.04821200** | **-0.34725100** |
| **C** | **2.70912100** | **-5.11373600** | **-0.69341900** |
| **C** | **2.18855000** | **-6.43695500** | **-0.90276600** |
| **C** | **0.86735800** | **-6.69835500** | **-0.75404000** |
| **C** | **2.37284300** | **-2.79025900** | **-0.06730500** |
| **C** | **3.74508500** | **-2.52742200** | **-0.13236000** |
| **C** | **4.61829900** | **-3.58921000** | **-0.54065000** |
| **C** | **4.07833600** | **-4.85120000** | **-0.80041000** |
| **C** | **4.32623300** | **-1.22465600** | **0.20815800** |
| **C** | **5.74084900** | **-1.03953700** | **0.06243200** |
| **C** | **6.56410800** | **-2.13102700** | **-0.39161500** |
| **C** | **6.03232200** | **-3.34761100** | **-0.66698800** |
| **C** | **3.56658700** | **-0.15376500** | **0.69114200** |
| **C** | **4.12243900** | **1.08824200** | **1.03306100** |
| **C** | **5.53451500** | **1.26551800** | **0.86294000** |
| **C** | **6.30213300** | **0.19781100** | **0.38305400** |
| **C** | **3.32780300** | **2.19708200** | **1.55825700** |
| **C** | **3.97993700** | **3.42538200** | **1.87567300** |
| **C** | **5.39862100** | **3.55426400** | **1.67748700** |
| **C** | **6.14139700** | **2.52480600** | **1.19557300** |
| **H** | **-3.46994300** | **6.82592200** | **-0.63831100** |
| **H** | **-5.46359900** | **5.59796000** | **0.12128300** |
| **H** | **-6.59673000** | **3.50518000** | **0.59864300** |
| **H** | **-2.50527700** | **0.93271200** | **-0.44126900** |
| **H** | **-7.82717300** | **1.45938200** | **1.04842400** |
| **H** | **-7.92508800** | **-1.00150000** | **1.15075500** |
| **H** | **-2.50181300** | **-1.01387100** | **-0.06199000** |
| **H** | **-6.85180500** | **-3.16861600** | **0.88626800** |
| **H** | **-5.85972400** | **-5.37598500** | **0.61030000** |
| **H** | **-3.89929100** | **-6.79002000** | **0.14550300** |
| **H** | **-1.66673000** | **-7.06700100** | **-0.36143400** |
| **H** | **2.88516000** | **-7.23071900** | **-1.16130500** |
| **H** | **0.48612500** | **-7.70788500** | **-0.88628700** |
| **H** | **1.69823000** | **-1.99394200** | **0.22308100** |
| **H** | **4.74727700** | **-5.66137000** | **-1.08391500** |
| **H** | **7.63268200** | **-1.95807100** | **-0.49407400** |
| **H** | **6.66594700** | **-4.16895100** | **-0.99256700** |
| **H** | **2.50164800** | **-0.29419400** | **0.82326800** |

| **H** | **7.37555900** | **0.33414100** | **0.26670200** |
| --- | --- | --- | --- |
| **H** | **5.86725800** | **4.50313800** | **1.92756500** |
| **H** | **7.21405800** | **2.63275500** | **1.05404300** |
| **C** | **3.22616200** | **4.50594300** | **2.38461800** |
| **C** | **1.86264800** | **4.39556300** | **2.58140800** |
| **C** | **1.21414300** | **3.18765000** | **2.27204200** |
| **C** | **1.93385600** | **2.11512300** | **1.77347700** |
| **H** | **3.74160100** | **5.43437500** | **2.61966100** |
| **H** | **1.29435500** | **5.23575900** | **2.97043100** |
| **H** | **0.14195000** | **3.09378400** | **2.41848600** |
| **H** | **1.40203800** | **1.19708900** | **1.54766400** |
| **C** | **-0.98564600** | **2.98639400** | **-1.16558400** |
| **C** | **0.13964800** | **3.69782500** | **-1.62926000** |
| **C** | **0.06890400** | **5.12880700** | **-1.73387900** |
| **C** | **-1.12904700** | **5.76619600** | **-1.36448300** |
| **H** | **-1.19470200** | **6.84991800** | **-1.44044400** |
| **H** | **-0.89720800** | **1.90603300** | **-1.10514900** |
| **C** | **1.34908700** | **3.04268400** | **-2.00148700** |
| **C** | **2.42828400** | **3.76162100** | **-2.45387900** |
| **C** | **2.35611600** | **5.17729900** | **-2.55947200** |
| **C** | **1.20723800** | **5.84264200** | **-2.20806300** |
| **H** | **1.14836400** | **6.92577900** | **-2.28657600** |
| **H** | **3.21880500** | **5.73143100** | **-2.91943600** |
| **H** | **3.34578500** | **3.24980400** | **-2.73024600** |
| **H** | **1.40511800** | **1.96015700** | **-1.91734300** |
| **P** | **-0.73026600** | **-2.95956100** | **-0.03474200** |

Expanded [15]-helicene:

X Y Z

**-----------------------------------------------------------------**

| **C** | **-2.41039100** | **-5.71968100** | **-0.58456300** |
| --- | --- | --- | --- |
| **C** | **-1.22127600** | **-6.20918100** | **-0.15504100** |
| **C** | **-0.05222700** | **-5.37103300** | **-0.08213000** |
| **C** | **-2.54796900** | **-4.34748100** | **-0.99982300** |
| **C** | **-1.41852200** | **-3.46544300** | **-0.93979300** |
| **C** | **-0.14011200** | **-3.98716700** | **-0.44541900** |
| **C** | **1.18024200** | **-5.89290100** | **0.32038500** |
| **C** | **2.33640000** | **-5.11053200** | **0.39172400** |
| **C** | **2.25103900** | **-3.71240000** | **0.09069000** |
| **C** | **1.01432200** | **-3.20154000** | **-0.32603200** |
| **C** | **3.60773000** | **-5.69631900** | **0.72830800** |
| **C** | **4.74317400** | **-4.95438700** | **0.73351300** |
| **C** | **4.71152500** | **-3.54124500** | **0.46008500** |
| **C** | **3.46134000** | **-2.89318700** | **0.19319000** |
| **C** | **3.46155100** | **-1.50055700** | **0.03681800** |
| **C** | **5.88682800** | **-2.78451000** | **0.44517000** |
| **C** | **5.88324300** | **-1.40479900** | **0.22236000** |
| **C** | **4.62963300** | **-0.72773200** | **0.06921500** |
| **C** | **7.11441400** | **-0.66458100** | **0.12453600** |
| **C** | **7.11399400** | **0.66888800** | **-0.12414800** |
| **C** | **5.88235700** | **1.40832100** | **-0.22202300** |
| **C** | **4.62917100** | **0.73046200** | **-0.06890300** |
| **C** | **3.46059100** | **1.50254000** | **-0.03657900** |
| **C** | **3.45947900** | **2.89515900** | **-0.19306900** |
| **C** | **4.70927100** | **3.54399600** | **-0.45992600** |
| **C** | **5.88506400** | **2.78802300** | **-0.44488100** |
| **C** | **2.24863300** | **3.71360700** | **-0.09078600** |
| **C** | **2.33314400** | **5.11176800** | **-0.39193600** |
| **C** | **3.60413400** | **5.69834200** | **-0.72842900** |
| **C** | **4.74005000** | **4.95713400** | **-0.73346300** |
| **C** | **1.01218000** | **3.20201800** | **0.32583800** |
| **C** | **-0.14274800** | **3.98694500** | **0.44503900** |

| **C** | **-0.05570500** | **5.37081900** | **0.08157900** |
| --- | --- | --- | --- |
| **C** | **1.17648300** | **5.89341500** | **-0.32084100** |
| **C** | **-1.42086200** | **3.46449100** | **0.93938800** |
| **C** | **-2.55089000** | **4.34580700** | **0.99912500** |
| **C** | **-2.41416400** | **5.71801700** | **0.58361500** |
| **C** | **-1.22530400** | **6.20822400** | **0.15419300** |
| **C** | **-1.59038500** | **2.14639700** | **1.37702600** |
| **C** | **-2.80732000** | **1.64974500** | **1.86638300** |
| **C** | **-3.92878300** | **2.54053500** | **1.91458300** |
| **C** | **-3.76897300** | **3.86043000** | **1.47808200** |
| **C** | **-2.97624500** | **0.27391700** | **2.33060200** |
| **C** | **-4.25006700** | **-0.13845000** | **2.82282300** |
| **C** | **-5.34305700** | **0.79600200** | **2.85085100** |
| **C** | **-5.19157700** | **2.07317900** | **2.41596800** |
| **H** | **-3.28692900** | **-6.36092100** | **-0.63724000** |
| **H** | **-1.12436300** | **-7.25079700** | **0.14131400** |
| **H** | **1.24645000** | **-6.95074300** | **0.56717000** |
| **H** | **0.95691800** | **-2.15516500** | **-0.59791600** |
| **H** | **3.63954500** | **-6.76008500** | **0.95081900** |
| **H** | **5.70374300** | **-5.41212800** | **0.95680700** |
| **H** | **2.51651000** | **-0.99831500** | **-0.12941400** |
| **H** | **6.83811500** | **-3.28894200** | **0.60272100** |
| **H** | **8.04979100** | **-1.20895900** | **0.22841600** |
| **H** | **8.04902900** | **1.21385700** | **-0.22801100** |
| **H** | **2.51588700** | **0.99968900** | **0.12972400** |
| **H** | **6.83603200** | **3.29306900** | **-0.60238100** |
| **H** | **3.63529100** | **6.76211200** | **-0.95101400** |
| **H** | **5.70034700** | **5.41546400** | **-0.95671700** |
| **H** | **0.95534900** | **2.15561400** | **0.59773800** |
| **H** | **1.24205400** | **6.95126600** | **-0.56775800** |
| **H** | **-3.29113100** | **6.35869000** | **0.63605200** |
| **H** | **-1.12902700** | **7.24985200** | **-0.14232600** |
| **H** | **-0.73872300** | **1.47921100** | **1.35017700** |
| **H** | **-4.62239900** | **4.53427200** | **1.52170200** |
| **H** | **-6.30233000** | **0.45210000** | **3.23032900** |
| **H** | **-6.02537300** | **2.77059900** | **2.44082200** |
| **C** | **-4.42853000** | **-1.46396500** | **3.27681200** |
| **C** | **-3.38932800** | **-2.37484600** | **3.24929500** |
| **C** | **-2.13258400** | **-1.97644000** | **2.76239800** |
| **C** | **-1.93512100** | **-0.68074500** | **2.31629300** |
| **H** | **-5.40721600** | **-1.75847500** | **3.64885800** |
| **H** | **-3.54182400** | **-3.39247800** | **3.59787000** |
| **H** | **-1.31188300** | **-2.68717800** | **2.72793600** |
| **H** | **-0.95283700** | **-0.40766400** | **1.94617100** |
| **C** | **-1.58887800** | **-2.14734900** | **-1.37712000** |
| **C** | **-2.80610100** | **-1.65136900** | **-1.86643400** |
| **C** | **-3.92697900** | **-2.54287800** | **-1.91492600** |
| **C** | **-3.76633400** | **-3.86278100** | **-1.47875600** |
| **H** | **-4.61932100** | **-4.53716400** | **-1.52259400** |
| **H** | **-0.73767000** | **-1.47959800** | **-1.34999100** |
| **C** | **-5.34236900** | **-0.79898600** | **-2.85070700** |
| **C** | **-5.19006400** | **-2.07619700** | **-2.41620900** |
| **H** | **-6.02340700** | **-2.77415000** | **-2.44128200** |
| **H** | **-6.30186400** | **-0.45558800** | **-3.23007700** |
| **C** | **-4.24998200** | **0.13616400** | **-2.82239500** |
| **C** | **-2.97589600** | **-0.27552800** | **-2.33029700** |
| **C** | **-4.42929500** | **1.46170100** | **-3.27598700** |
| **C** | **-1.93536700** | **0.67978100** | **-2.31575100** |
| **C** | **-3.39067300** | **2.37324000** | **-3.24820500** |
| **H** | **-5.40817200** | **1.75569600** | **-3.64793700** |
| **C** | **-2.13366000** | **1.97548500** | **-2.76146500** |
| **H** | **-0.95288600** | **0.40719500** | **-1.94578400** |
| **H** | **-3.54382500** | **3.39088200** | **-3.59646600** |

H -1.31340600 2.68673100 -2.72682800

Bor Doped Expanded [15]-helicene:

X Y Z

**-----------------------------------------------------------------**

| **C** | **-0.47084400** | **-6.36802700** | **-0.26643600** |
| --- | --- | --- | --- |
| **C** | **0.82248300** | **-6.46881400** | **0.12808100** |
| **C** | **1.69113100** | **-5.31989100** | **0.13923700** |
| **C** | **-1.02055800** | **-5.11405600** | **-0.71373300** |
| **C** | **-0.20099400** | **-3.93689700** | **-0.72081300** |
| **C** | **1.18406700** | **-4.03690500** | **-0.24980400** |
| **C** | **3.03425900** | **-5.43882500** | **0.50715400** |
| **C** | **3.90424000** | **-4.34465900** | **0.52511700** |
| **C** | **3.39216000** | **-3.04392800** | **0.21169400** |
| **C** | **2.04985300** | **-2.93772800** | **-0.17585600** |
| **C** | **5.30364000** | **-4.51302200** | **0.81718400** |
| **C** | **6.16162200** | **-3.46325900** | **0.76699800** |
| **C** | **5.69683400** | **-2.13023400** | **0.48548300** |
| **C** | **4.29836900** | **-1.89498200** | **0.26675300** |
| **C** | **3.87198600** | **-0.56975800** | **0.11055500** |
| **C** | **6.59108300** | **-1.05527900** | **0.42741800** |
| **C** | **6.16357900** | **0.25561500** | **0.20436500** |
| **C** | **4.76083100** | **0.51297600** | **0.08987900** |
| **C** | **7.09037300** | **1.35107200** | **0.08159200** |
| **C** | **6.66819300** | **2.62221000** | **-0.14202200** |
| **C** | **5.26711100** | **2.96385800** | **-0.21310800** |
| **C** | **4.30985500** | **1.87664600** | **-0.05843600** |
| **C** | **2.40775100** | **3.84517100** | **-0.18537400** |
| **C** | **3.53244800** | **4.74325500** | **-0.41280200** |
| **C** | **4.86853400** | **4.30004900** | **-0.40422800** |
| **C** | **1.05862900** | **4.35580600** | **-0.11782200** |
| **C** | **0.82929100** | **5.74413200** | **-0.37385800** |
| **C** | **1.95190600** | **6.60276900** | **-0.64951700** |
| **C** | **3.22653300** | **6.13451800** | **-0.64791000** |
| **C** | **-0.03098100** | **3.54232700** | **0.22160500** |
| **C** | **-1.34046900** | **4.03092600** | **0.32033400** |
| **C** | **-1.56634100** | **5.41238200** | **0.00327900** |
| **C** | **-0.47986200** | **6.22907600** | **-0.32859900** |
| **C** | **-2.47749100** | **3.20942600** | **0.74688700** |
| **C** | **-3.78626200** | **3.79607800** | **0.76758400** |
| **C** | **-3.96102300** | **5.17490200** | **0.39092600** |
| **C** | **-2.90301700** | **5.94686800** | **0.03993900** |
| **C** | **-2.34419500** | **1.88036900** | **1.16366000** |
| **C** | **-3.42624000** | **1.09789900** | **1.59265200** |
| **C** | **-4.73061700** | **1.69146700** | **1.58918000** |
| **C** | **-4.87293900** | **3.02111400** | **1.17678800** |
| **C** | **-3.27922400** | **-0.28386900** | **2.04627200** |
| **C** | **-4.43865500** | **-1.00154200** | **2.46535600** |
| **C** | **-5.72605800** | **-0.36105100** | **2.43571100** |
| **C** | **-5.86643900** | **0.92322800** | **2.01839100** |
| **H** | **-1.11862300** | **-7.24134500** | **-0.26941500** |
| **H** | **1.23275500** | **-7.42447400** | **0.44518400** |
| **H** | **3.42147500** | **-6.42162000** | **0.76880900** |
| **H** | **1.67425400** | **-1.96276900** | **-0.45996700** |
| **H** | **5.66327800** | **-5.51250500** | **1.04891700** |
| **H** | **7.22303900** | **-3.60629900** | **0.95434400** |
| **H** | **2.81293500** | **-0.35561000** | **-0.00300800** |
| **H** | **7.65402800** | **-1.24912000** | **0.55732400** |
| **H** | **8.15299500** | **1.13257100** | **0.15542400** |
| **H** | **7.38869200** | **3.42890800** | **-0.25355000** |
| **H** | **5.64771600** | **5.04412200** | **-0.54475400** |
| **H** | **1.75600200** | **7.65508300** | **-0.84097800** |
| **H** | **4.05971400** | **6.80797900** | **-0.83384900** |
| **H** | **0.17032400** | **2.49611200** | **0.43354000** |

| **H** | **-0.66073100** | **7.28041600** | **-0.54387400** |
| --- | --- | --- | --- |
| **H** | **-4.96616600** | **5.58890600** | **0.41279700** |
| **H** | **-3.04306700** | **6.99253900** | **-0.22311400** |
| **H** | **-1.35609300** | **1.43897400** | **1.17200600** |
| **H** | **-5.86409000** | **3.47039500** | **1.18761500** |
| **H** | **-6.59160700** | **-0.93401700** | **2.75982400** |
| **H** | **-6.84445600** | **1.39811700** | **2.00293600** |
| **C** | **-4.31261200** | **-2.33699400** | **2.90750400** |
| **C** | **-3.08212500** | **-2.96559700** | **2.94120500** |
| **C** | **-1.93554000** | **-2.26446600** | **2.53027200** |
| **C** | **-2.03674200** | **-0.95406200** | **2.09492200** |
| **H** | **-5.20877500** | **-2.86690500** | **3.22225900** |
| **H** | **-2.99997700** | **-3.99437800** | **3.28046400** |
| **H** | **-0.96510800** | **-2.75205500** | **2.54672800** |
| **H** | **-1.13203900** | **-0.44220000** | **1.78507100** |
| **C** | **-0.76055600** | **-2.74801500** | **-1.20143500** |
| **C** | **-2.08024600** | **-2.65276300** | **-1.66625500** |
| **C** | **-2.89214100** | **-3.83311400** | **-1.63769900** |
| **C** | **-2.33970400** | **-5.02805100** | **-1.16262900** |
| **H** | **-2.95737700** | **-5.92400700** | **-1.15302700** |
| **H** | **-0.14139700** | **-1.86085000** | **-1.23058300** |
| **C** | **-4.78138600** | **-2.62071100** | **-2.57637800** |
| **C** | **-4.25001600** | **-3.77735200** | **-2.10394400** |
| **H** | **-4.84365500** | **-4.68766400** | **-2.07001800** |
| **H** | **-5.81019100** | **-2.58851300** | **-2.92701800** |
| **C** | **-4.00864300** | **-1.40881900** | **-2.62873100** |
| **C** | **-2.65554600** | **-1.41020900** | **-2.17740900** |
| **C** | **-4.57931300** | **-0.21527000** | **-3.12346300** |
| **C** | **-1.93610000** | **-0.19650600** | **-2.24888000** |
| **C** | **-3.84979500** | **0.95741900** | **-3.17694800** |
| **H** | **-5.61273600** | **-0.23475900** | **-3.46191900** |
| **C** | **-2.51580600** | **0.96324200** | **-2.73451200** |
| **H** | **-0.90348300** | **-0.15791200** | **-1.91869600** |
| **H** | **-4.30293500** | **1.86927500** | **-3.55557700** |
| **H** | **-1.93621100** | **1.88142900** | **-2.76720600** |
| **B** | **3.04236300** | **2.55825800** | **-0.07379200** |

Nitrogene Doped Expanded [15]-helicene:

X Y Z

**-----------------------------------------------------------------**

| **C** | **3.32007900** | **4.88503800** | **-0.95296900** |
| --- | --- | --- | --- |
| **C** | **2.23738000** | **5.59182600** | **-0.54563300** |
| **C** | **0.94463900** | **4.96924600** | **-0.42067600** |
| **C** | **3.21716300** | **3.48941100** | **-1.29296200** |
| **C** | **1.95669600** | **2.81528000** | **-1.17561800** |
| **C** | **0.79262900** | **3.57206300** | **-0.70373200** |
| **C** | **-0.17669200** | **5.71610600** | **-0.04771900** |
| **C** | **-1.44814400** | **5.14709400** | **0.06782800** |
| **C** | **-1.60248400** | **3.73969300** | **-0.14741200** |
| **C** | **-0.47608800** | **3.00101300** | **-0.53413600** |
| **C** | **-2.60192400** | **5.95799300** | **0.35830600** |
| **C** | **-3.84789900** | **5.42243500** | **0.39218400** |
| **C** | **-4.05883300** | **4.01050200** | **0.20221900** |
| **C** | **-2.93288400** | **3.14465100** | **-0.00450100** |
| **C** | **-3.16531800** | **1.76534400** | **-0.06736400** |
| **C** | **-5.34915100** | **3.46982000** | **0.21619100** |
| **C** | **-5.57962000** | **2.09835500** | **0.07188000** |
| **C** | **-4.45296200** | **1.22275200** | **-0.02105200** |
| **C** | **-6.91266700** | **1.55647100** | **0.00231500** |
| **C** | **-7.12476800** | **0.22612700** | **-0.17022400** |
| **C** | **-6.02337800** | **-0.69900400** | **-0.22500100** |
| **C** | **-4.68078500** | **-0.21775300** | **-0.08958300** |

| **C** | **-3.81540000** | **-2.36838200** | **-0.12166700** |
| --- | --- | --- | --- |
| **C** | **-5.10420800** | **-2.94017600** | **-0.37588100** |
| **C** | **-6.19906300** | **-2.07339100** | **-0.39941500** |
| **C** | **-2.67144000** | **-3.25911000** | **0.04987900** |
| **C** | **-2.84483700** | **-4.66094300** | **-0.17030000** |
| **C** | **-4.14739900** | **-5.17545700** | **-0.50781000** |
| **C** | **-5.23031500** | **-4.35912300** | **-0.58189500** |
| **C** | **-1.42560100** | **-2.77937000** | **0.46658900** |
| **C** | **-0.32927100** | **-3.62451300** | **0.67827200** |
| **C** | **-0.48947700** | **-5.02160600** | **0.38884500** |
| **C** | **-1.73759600** | **-5.50195000** | **-0.02210800** |
| **C** | **0.95771300** | **-3.15184600** | **1.19659200** |
| **C** | **2.03505700** | **-4.08789700** | **1.33186600** |
| **C** | **1.83333800** | **-5.46862200** | **0.97546400** |
| **C** | **0.62990900** | **-5.91607000** | **0.53920700** |
| **C** | **1.17744000** | **-1.82895700** | **1.59505000** |
| **C** | **2.40097700** | **-1.37819200** | **2.11226500** |
| **C** | **3.47399600** | **-2.32149700** | **2.22692400** |
| **C** | **3.26111400** | **-3.64736800** | **1.83275300** |
| **C** | **2.62197800** | **0.00057600** | **2.54482800** |
| **C** | **3.89773500** | **0.36487200** | **3.06906900** |
| **C** | **4.94238900** | **-0.61964000** | **3.15840700** |
| **C** | **4.74197900** | **-1.90057800** | **2.75564100** |
| **H** | **4.29165900** | **5.36430600** | **-1.04589600** |
| **H** | **2.32211000** | **6.64946900** | **-0.30798900** |
| **H** | **-0.05962200** | **6.78258700** | **0.13486200** |
| **H** | **-0.60409000** | **1.94637400** | **-0.74246100** |
| **H** | **-2.45296700** | **7.02318600** | **0.51799000** |
| **H** | **-4.71600600** | **6.05067900** | **0.57604100** |
| **H** | **-2.34362100** | **1.06578100** | **-0.16006700** |
| **H** | **-6.19854400** | **4.13983500** | **0.33326600** |
| **H** | **-7.75138700** | **2.24525100** | **0.06672200** |
| **H** | **-8.13439300** | **-0.16934900** | **-0.25178600** |
| **H** | **-7.20032700** | **-2.47419000** | **-0.54717500** |
| **H** | **-4.25018600** | **-6.24488000** | **-0.67470900** |
| **H** | **-6.21553000** | **-4.76200700** | **-0.80424100** |
| **H** | **-1.35037800** | **-1.71364300** | **0.64352300** |
| **H** | **-1.85367200** | **-6.56724900** | **-0.21155400** |
| **H** | **2.67167700** | **-6.15269500** | **1.08325700** |
| **H** | **0.48431900** | **-6.96531400** | **0.29399600** |
| **H** | **0.35819500** | **-1.12591200** | **1.51882700** |
| **H** | **4.07641100** | **-4.36139700** | **1.93131300** |
| **H** | **5.90498000** | **-0.31124800** | **3.55948000** |
| **H** | **5.53907000** | **-2.63650700** | **2.82837000** |
| **C** | **4.12535000** | **1.69194200** | **3.49553400** |
| **C** | **3.13214400** | **2.64950800** | **3.41140800** |
| **C** | **1.87405900** | **2.29824000** | **2.89271800** |
| **C** | **1.62863000** | **1.00235500** | **2.47140600** |
| **H** | **5.10464300** | **1.94954400** | **3.89262200** |
| **H** | **3.32209700** | **3.66749800** | **3.74015400** |
| **H** | **1.08974500** | **3.04539500** | **2.81364700** |
| **H** | **0.64705400** | **0.76759500** | **2.07435100** |
| **C** | **1.89458500** | **1.46619200** | **-1.54201100** |
| **C** | **3.00206600** | **0.74799100** | **-2.01687200** |
| **C** | **4.25559500** | **1.43418300** | **-2.12442600** |
| **C** | **4.32775700** | **2.78227400** | **-1.75637700** |
| **H** | **5.28105200** | **3.29979400** | **-1.84426300** |
| **H** | **0.94400600** | **0.95392000** | **-1.47056900** |
| **C** | **5.33826100** | **-0.56508900** | **-2.99083800** |
| **C** | **5.41198700** | **0.73789200** | **-2.61676700** |
| **H** | **6.35005800** | **1.28329500** | **-2.68645000** |
| **H** | **6.21816300** | **-1.08255800** | **-3.36580200** |
| **C** | **4.10499300** | **-1.29988200** | **-2.90288500** |

| **C** | **2.92917900** | **-0.65761900** | **-2.41254200** |
| --- | --- | --- | --- |
| **C** | **4.04939000** | **-2.65540000** | **-3.29577900** |
| **C** | **1.74422500** | **-1.42272500** | **-2.33411800** |
| **C** | **2.87332200** | **-3.37643200** | **-3.20940600** |
| **H** | **4.95644300** | **-3.12612300** | **-3.66833000** |
| **C** | **1.71286200** | **-2.75171800** | **-2.72133300** |
| **H** | **0.83109800** | **-0.97554000** | **-1.95696700** |
| **H** | **2.84552600** | **-4.41951900** | **-3.51229700** |
| **H** | **0.78625900** | **-3.31235500** | **-2.63864700** |
| **N** | **-3.62918400** | **-1.04509000** | **-0.01928500** |

Silicone Doped Expanded [15]-helicene:

X Y Z

**-----------------------------------------------------------------**

| **C** | **-4.14584700** | **5.53742000** | **-0.01389900** |
| --- | --- | --- | --- |
| **C** | **-5.20885600** | **4.77080900** | **0.33406900** |
| **C** | **-5.13080800** | **3.33262800** | **0.31482900** |
| **C** | **-2.90383000** | **4.94469600** | **-0.43957000** |
| **C** | **-2.77116400** | **3.51703100** | **-0.47889900** |
| **C** | **-3.90716800** | **2.68949600** | **-0.06208200** |
| **C** | **-6.23811100** | **2.54735500** | **0.64832400** |
| **C** | **-6.19237800** | **1.14995200** | **0.64400900** |
| **C** | **-4.95766500** | **0.49514900** | **0.32939000** |
| **C** | **-3.85987500** | **1.29027000** | **-0.02328800** |
| **C** | **-7.36736600** | **0.36712200** | **0.92580800** |
| **C** | **-7.33630100** | **-0.98847600** | **0.87804100** |
| **C** | **-6.11652400** | **-1.69805100** | **0.59458700** |
| **C** | **-4.90389400** | **-0.96853300** | **0.36293600** |
| **C** | **-3.72222400** | **-1.69582100** | **0.17871600** |
| **C** | **-6.08913000** | **-3.09499000** | **0.54342400** |
| **C** | **-4.91073700** | **-3.80951100** | **0.31180900** |
| **C** | **-3.67595200** | **-3.09777800** | **0.16575500** |
| **C** | **-4.91053800** | **-5.24358000** | **0.20725500** |
| **C** | **-3.76718500** | **-5.93209500** | **-0.03027100** |
| **C** | **-2.48223000** | **-5.27481600** | **-0.13408200** |
| **C** | **-2.43306300** | **-3.84384900** | **0.00192100** |
| **C** | **0.49251200** | **-4.38664100** | **-0.22477600** |
| **C** | **-0.00020400** | **-5.72315000** | **-0.42766500** |
| **C** | **-1.35708200** | **-6.08920200** | **-0.35728200** |
| **C** | **1.93165400** | **-4.14497000** | **-0.23428300** |
| **C** | **2.82324100** | **-5.22190700** | **-0.54531500** |
| **C** | **2.28534200** | **-6.53187700** | **-0.79291700** |
| **C** | **0.95358700** | **-6.77215800** | **-0.71571500** |
| **C** | **2.49413600** | **-2.89586200** | **0.07264200** |
| **C** | **3.87301000** | **-2.65254000** | **0.07508100** |
| **C** | **4.74718800** | **-3.73078600** | **-0.28652300** |
| **C** | **4.19939800** | **-4.98240200** | **-0.58041600** |
| **C** | **4.46170000** | **-1.35996500** | **0.44360700** |
| **C** | **5.88743800** | **-1.20910900** | **0.38970300** |
| **C** | **6.71304400** | **-2.31753100** | **-0.01530100** |
| **C** | **6.17073200** | **-3.51975200** | **-0.32943300** |
| **C** | **3.69947300** | **-0.26530900** | **0.86682900** |
| **C** | **4.26197400** | **0.96457000** | **1.24062900** |
| **C** | **5.68689600** | **1.10185500** | **1.17518300** |
| **C** | **6.45698300** | **0.01371000** | **0.74909300** |
| **C** | **3.46254700** | **2.09962900** | **1.69803600** |
| **C** | **4.12470600** | **3.30751500** | **2.06899600** |
| **C** | **5.55780300** | **3.39336100** | **1.98297900** |
| **C** | **6.30379300** | **2.34280400** | **1.55495300** |
| **H** | **-4.21485200** | **6.62232800** | **0.00749800** |
| **H** | **-6.14678200** | **5.22912800** | **0.63802900** |
| **H** | **-7.17258300** | **3.04109200** | **0.90766300** |

| **H** | **-2.93579400** | **0.79745700** | **-0.29682400** |
| --- | --- | --- | --- |
| **H** | **-8.29084200** | **0.89248700** | **1.15653900** |
| **H** | **-8.23538600** | **-1.56996700** | **1.06696200** |
| **H** | **-2.79302300** | **-1.15493000** | **0.03885900** |
| **H** | **-7.01748800** | **-3.64473100** | **0.68620600** |
| **H** | **-5.85874300** | **-5.76675400** | **0.30612800** |
| **H** | **-3.79096800** | **-7.01448300** | **-0.12876200** |
| **H** | **-0.50418300** | **-1.72144100** | **0.18145300** |
| **H** | **-1.56367000** | **-7.14935200** | **-0.48627200** |
| **H** | **2.97843900** | **-7.33701700** | **-1.02521800** |
| **H** | **0.56787400** | **-7.77485400** | **-0.88193300** |
| **H** | **1.82161300** | **-2.08787300** | **0.33796300** |
| **H** | **4.86881800** | **-5.80271700** | **-0.83229000** |
| **H** | **7.78950700** | **-2.16864900** | **-0.05041400** |
| **H** | **6.80362400** | **-4.35463300** | **-0.62032300** |
| **H** | **2.62424300** | **-0.37432500** | **0.92187900** |
| **H** | **7.53910400** | **0.12153300** | **0.70680100** |
| **H** | **6.03402900** | **4.32746000** | **2.27149000** |
| **H** | **7.38681100** | **2.41880300** | **1.49484500** |
| **C** | **3.36642300** | **4.41179300** | **2.51677500** |
| **C** | **1.98837500** | **4.34570900** | **2.59956800** |
| **C** | **1.32929200** | **3.15979100** | **2.23289000** |
| **C** | **2.05367500** | **2.06444000** | **1.79426200** |
| **H** | **3.89024700** | **5.32334200** | **2.79549800** |
| **H** | **1.41699100** | **5.20388200** | **2.94205200** |
| **H** | **0.24587200** | **3.10206200** | **2.28584400** |
| **H** | **1.51368700** | **1.16641100** | **1.51526000** |
| **C** | **-1.56256200** | **2.98007000** | **-0.93611600** |
| **C** | **-0.47850200** | **3.76846600** | **-1.34896600** |
| **C** | **-0.61766200** | **5.19341500** | **-1.28341700** |
| **C** | **-1.82519800** | **5.73948500** | **-0.83266100** |
| **H** | **-1.92967100** | **6.82214700** | **-0.79609900** |
| **H** | **-1.46267900** | **1.90371100** | **-0.98784200** |
| **C** | **1.64126100** | **5.50493000** | **-2.13226600** |
| **C** | **0.47418800** | **6.03622800** | **-1.68597200** |
| **H** | **0.34276100** | **7.11379400** | **-1.62401000** |
| **H** | **2.46272100** | **6.15103200** | **-2.43243400** |
| **C** | **1.82868000** | **4.08200600** | **-2.22575800** |
| **C** | **0.77486100** | **3.20106600** | **-1.84222800** |
| **C** | **3.04881300** | **3.54897900** | **-2.69727800** |
| **C** | **1.00098500** | **1.81136800** | **-1.96193000** |
| **C** | **3.24201800** | **2.18423500** | **-2.79645000** |
| **H** | **3.84137400** | **4.23732700** | **-2.98144900** |
| **C** | **2.20524600** | **1.31011400** | **-2.42657500** |
| **H** | **0.21922800** | **1.10944500** | **-1.69098000** |
| **H** | **4.18727800** | **1.78780600** | **-3.15589100** |
| **H** | **2.34906600** | **0.23593800** | **-2.50274000** |
| **Si** | **-0.78429700** | **-3.15902100** | **-0.00086200** |

Phosphore Doped Expanded [15]-helicene:

X Y Z

**-----------------------------------------------------------------**

| **C** | **-2.55618100** | **6.19419600** | **-0.20344300** |
| --- | --- | --- | --- |
| **C** | **-3.75996500** | **5.70963400** | **0.19011400** |
| **C** | **-4.02307200** | **4.29368900** | **0.21371100** |
| **C** | **-1.49887100** | **5.31419700** | **-0.63067900** |
| **C** | **-1.70439500** | **3.89464600** | **-0.62137500** |
| **C** | **-2.99494800** | **3.37005400** | **-0.16467800** |
| **C** | **-5.27695600** | **3.80143900** | **0.58750300** |
| **C** | **-5.56395200** | **2.43360900** | **0.61674100** |
| **C** | **-4.52856300** | **1.49778000** | **0.29474500** |
| **C** | **-3.28031000** | **2.00046000** | **-0.09294100** |

| **C** | **-6.88480000** | **1.95864900** | **0.93787900** |
| --- | --- | --- | --- |
| **C** | **-7.18104300** | **0.63489200** | **0.91783900** |
| **C** | **-6.17318000** | **-0.34984700** | **0.62314800** |
| **C** | **-4.82673000** | **0.06448600** | **0.35498600** |
| **C** | **-3.85730200** | **-0.92529200** | **0.15968800** |
| **C** | **-6.48262600** | **-1.71206400** | **0.59214400** |
| **C** | **-5.51567900** | **-2.69109700** | **0.34505600** |
| **C** | **-4.14906100** | **-2.29807900** | **0.16655800** |
| **C** | **-5.87284100** | **-4.08032400** | **0.25333200** |
| **C** | **-4.93778100** | **-5.02474600** | **-0.01009000** |
| **C** | **-3.53916800** | **-4.68881200** | **-0.14525800** |
| **C** | **-3.12060100** | **-3.32811300** | **-0.01012600** |
| **C** | **-0.56160200** | **-4.37320400** | **-0.25332000** |
| **C** | **-1.24024100** | **-5.60954900** | **-0.49186200** |
| **C** | **-2.63293300** | **-5.72293500** | **-0.40686800** |
| **C** | **0.90482100** | **-4.37508800** | **-0.23982700** |
| **C** | **1.61000400** | **-5.57490800** | **-0.58047700** |
| **C** | **0.87403000** | **-6.77206800** | **-0.88135900** |
| **C** | **-0.47894800** | **-6.79382500** | **-0.81523600** |
| **C** | **1.66240900** | **-3.24969800** | **0.12350400** |
| **C** | **3.06151400** | **-3.24364600** | **0.15536400** |
| **C** | **3.75102200** | **-4.44017900** | **-0.23261300** |
| **C** | **3.00803200** | **-5.56888900** | **-0.58615500** |
| **C** | **3.84810600** | **-2.08173500** | **0.58438100** |
| **C** | **5.27938400** | **-2.17407600** | **0.57356500** |
| **C** | **5.91714600** | **-3.39222600** | **0.14443100** |
| **C** | **5.19066000** | **-4.47232000** | **-0.23497200** |
| **C** | **3.26774700** | **-0.88643800** | **1.02193200** |
| **C** | **4.01812900** | **0.21825800** | **1.45171900** |
| **C** | **5.44700300** | **0.11123400** | **1.43628100** |
| **C** | **6.03602000** | **-1.07912400** | **0.99443800** |
| **C** | **3.40656200** | **1.46184900** | **1.91587700** |
| **C** | **4.25002700** | **2.52773500** | **2.34864700** |
| **C** | **5.67891300** | **2.36755700** | **2.31625700** |
| **C** | **6.25120800** | **1.21669000** | **1.87862200** |
| **H** | **-2.36820200** | **7.26507900** | **-0.21737300** |
| **H** | **-4.55591300** | **6.38411200** | **0.49623900** |
| **H** | **-6.06156200** | **4.50846900** | **0.84977300** |
| **H** | **-2.50673500** | **1.29596600** | **-0.36978700** |
| **H** | **-7.65012900** | **2.69351400** | **1.17545800** |
| **H** | **-8.18838100** | **0.28889200** | **1.13601000** |
| **H** | **-2.82857700** | **-0.62813400** | **-0.00535800** |
| **H** | **-7.51218400** | **-2.02190000** | **0.76011400** |
| **H** | **-6.91753000** | **-4.35401300** | **0.37922300** |
| **H** | **-5.22054500** | **-6.07019300** | **-0.10508900** |
| **H** | **-3.05250100** | **-6.71705900** | **-0.54984800** |
| **H** | **1.43131300** | **-7.66938600** | **-1.13905600** |
| **H** | **-1.02693600** | **-7.71139500** | **-1.01450300** |
| **H** | **1.12664800** | **-2.35351100** | **0.41251200** |
| **H** | **3.53515600** | **-6.48128400** | **-0.85805500** |
| **H** | **7.00392100** | **-3.42770900** | **0.14226600** |
| **H** | **5.68263300** | **-5.39072300** | **-0.54576200** |
| **H** | **2.18850000** | **-0.81169500** | **1.03930900** |
| **H** | **7.12156100** | **-1.15618400** | **0.98675300** |
| **H** | **6.29586000** | **3.19785100** | **2.65176800** |
| **H** | **7.33285300** | **1.10763000** | **1.85730200** |
| **C** | **3.67419600** | **3.73480700** | **2.80288600** |
| **C** | **2.30295900** | **3.90544100** | **2.83139500** |
| **C** | **1.46622600** | **2.86087600** | **2.40212100** |
| **C** | **2.00950100** | **1.66769600** | **1.95704400** |
| **H** | **4.33429300** | **4.53520600** | **3.12943900** |
| **H** | **1.87321300** | **4.84067000** | **3.17922000** |
| **H** | **0.38776800** | **2.98986900** | **2.41163600** |

| **H** | **1.33529100** | **0.88350300** | **1.63067800** |
| --- | --- | --- | --- |
| **C** | **-0.66369400** | **3.07485200** | **-1.07193700** |
| **C** | **0.56452700** | **3.57451400** | **-1.52855200** |
| **C** | **0.75996800** | **4.99421600** | **-1.52170400** |
| **C** | **-0.27544600** | **5.82193000** | **-1.07219500** |
| **H** | **-0.12450900** | **6.89971800** | **-1.07608200** |
| **H** | **-0.81680200** | **2.00366800** | **-1.08255800** |
| **C** | **3.00379200** | **4.74179300** | **-2.42784300** |
| **C** | **2.00459300** | **5.54549000** | **-1.98159100** |
| **H** | **2.12681900** | **6.62578700** | **-1.96461500** |
| **H** | **3.94224700** | **5.16841300** | **-2.77391800** |
| **C** | **2.85717400** | **3.31130700** | **-2.45812500** |
| **C** | **1.64181500** | **2.71313400** | **-2.01177200** |
| **C** | **3.90840100** | **2.49252300** | **-2.92663900** |
| **C** | **1.54249000** | **1.30450800** | **-2.06110900** |
| **C** | **3.78209000** | **1.11664300** | **-2.95958000** |
| **H** | **4.82777600** | **2.96764200** | **-3.26116000** |
| **C** | **2.58667200** | **0.52059300** | **-2.52218600** |
| **H** | **0.63314400** | **0.81127300** | **-1.73446700** |
| **H** | **4.60043100** | **0.49852800** | **-3.31789900** |
| **H** | **2.48004400** | **-0.56028600** | **-2.54034500** |
| **P** | **-1.42309800** | **-2.85805800** | **0.00225300** |

Expanded [16]-helicene:

X Y Z

**-----------------------------------------------------------------**

| **C** | **4.16782800** | **-4.09551100** | **1.55635900** |
| --- | --- | --- | --- |
| **C** | **3.30993100** | **-5.04251900** | **1.10396100** |
| **C** | **1.92283100** | **-4.73860900** | **0.86420300** |
| **C** | **3.72400600** | **-2.75238900** | **1.82627400** |
| **C** | **2.35501900** | **-2.39149500** | **1.59643400** |
| **C** | **1.43244400** | **-3.41026000** | **1.08531500** |
| **C** | **1.03562300** | **-5.73009000** | **0.43589200** |
| **C** | **-0.31932200** | **-5.47362200** | **0.20674100** |
| **C** | **-0.81320300** | **-4.13889400** | **0.37249700** |
| **C** | **0.08134000** | **-3.15466600** | **0.81515100** |
| **C** | **-1.22323300** | **-6.53379100** | **-0.15621800** |
| **C** | **-2.55063800** | **-6.30244400** | **-0.31200700** |
| **C** | **-3.09765800** | **-4.97784300** | **-0.17426300** |
| **C** | **-2.23046300** | **-3.87329600** | **0.11261800** |
| **C** | **-2.78792300** | **-2.58786600** | **0.13873200** |
| **C** | **-4.47020400** | **-4.74841100** | **-0.30780400** |
| **C** | **-5.02583500** | **-3.46958000** | **-0.21193200** |
| **C** | **-4.15583100** | **-2.34409500** | **-0.03968800** |
| **C** | **-6.45145200** | **-3.27275400** | **-0.25799300** |
| **C** | **-6.99413200** | **-2.03693100** | **-0.12226300** |
| **C** | **-6.16323900** | **-0.86649800** | **-0.00818400** |
| **C** | **-4.73675400** | **-0.99982900** | **-0.02919100** |
| **C** | **-3.96450200** | **0.16894000** | **-0.05678900** |
| **C** | **-4.52148400** | **1.45383600** | **-0.01096500** |
| **C** | **-5.94261900** | **1.56734500** | **0.13600600** |
| **C** | **-6.72396200** | **0.40863000** | **0.10689800** |
| **C** | **-3.72330200** | **2.67871900** | **-0.10703700** |
| **C** | **-4.36934300** | **3.94061400** | **0.10311300** |
| **C** | **-5.79072300** | **3.99112800** | **0.32735600** |
| **C** | **-6.54442300** | **2.86367400** | **0.31071800** |
| **C** | **-2.36078400** | **2.68069200** | **-0.43375800** |
| **C** | **-1.59914400** | **3.85319900** | **-0.54171400** |
| **C** | **-2.24290200** | **5.10164900** | **-0.25303100** |
| **C** | **-3.60719900** | **5.11056600** | **0.05229500** |
| **C** | **-0.19241500** | **3.86287000** | **-0.95191500** |
| **C** | **0.50701800** | **5.11166200** | **-0.98890600** |

| **C** | **-0.17594100** | **6.32958500** | **-0.64045700** |
| --- | --- | --- | --- |
| **C** | **-1.48917000** | **6.32685700** | **-0.30103400** |
| **C** | **0.50249300** | **2.70740300** | **-1.33771700** |
| **C** | **1.83924200** | **2.71850300** | **-1.75169500** |
| **C** | **2.52859500** | **3.97342500** | **-1.77139400** |
| **C** | **1.84729600** | **5.13028700** | **-1.38837200** |
| **C** | **2.55491100** | **1.50755400** | **-2.17534200** |
| **C** | **3.92773100** | **1.62696200** | **-2.60547400** |
| **C** | **4.56633500** | **2.92020500** | **-2.59607100** |
| **C** | **3.90461800** | **4.03287500** | **-2.19814100** |
| **H** | **5.21321300** | **-4.33490600** | **1.73527400** |
| **H** | **3.65249700** | **-6.05698900** | **0.91513400** |
| **H** | **1.40870200** | **-6.74290700** | **0.29644300** |
| **H** | **-0.29857400** | **-2.15522400** | **0.98509800** |
| **H** | **-0.81762100** | **-7.53563700** | **-0.27447000** |
| **H** | **-3.23059100** | **-7.11544400** | **-0.55444900** |
| **H** | **-2.13469200** | **-1.74364500** | **0.32192900** |
| **H** | **-5.13000500** | **-5.59660000** | **-0.47946500** |
| **H** | **-7.08656100** | **-4.14744600** | **-0.37471800** |
| **H** | **-8.07294800** | **-1.90192900** | **-0.12333500** |
| **H** | **-2.88767800** | **0.07396500** | **-0.12517100** |
| **H** | **-7.80625100** | **0.50322500** | **0.16947200** |
| **H** | **-6.25162200** | **4.96401600** | **0.47955700** |
| **H** | **-7.62206500** | **2.91483800** | **0.44539800** |
| **H** | **-1.88171800** | **1.73237300** | **-0.64269900** |
| **H** | **-4.09712300** | **6.06479900** | **0.23590500** |
| **H** | **0.38585500** | **7.25992800** | **-0.67360400** |
| **H** | **-2.00212700** | **7.25458500** | **-0.05948300** |
| **H** | **-0.02573500** | **1.76256100** | **-1.33396900** |
| **H** | **2.37363600** | **6.08244200** | **-1.41344200** |
| **H** | **5.60263200** | **2.97919900** | **-2.91996400** |
| **H** | **4.39891800** | **5.00129100** | **-2.19699200** |
| **C** | **4.62455900** | **0.49757400** | **-3.02520300** |
| **C** | **2.67340000** | **-0.90821100** | **-2.60797200** |
| **C** | **1.97541100** | **0.24244500** | **-2.18862500** |
| **H** | **5.65808600** | **0.60665700** | **-3.34829400** |
| **H** | **0.94690600** | **0.10687000** | **-1.86886200** |
| **C** | **1.96161000** | **-1.08133900** | **1.89039200** |
| **C** | **2.83847300** | **-0.11111000** | **2.39748900** |
| **C** | **4.20375900** | **-0.48590500** | **2.61902800** |
| **C** | **4.60710600** | **-1.79321000** | **2.32485700** |
| **H** | **5.64433600** | **-2.07353300** | **2.49754100** |
| **H** | **0.92788500** | **-0.80351900** | **1.73077600** |
| **C** | **3.38036900** | **2.16842800** | **3.23684900** |
| **C** | **4.73839000** | **1.74098500** | **3.44098000** |
| **C** | **5.13266600** | **0.47608100** | **3.14460800** |
| **H** | **6.16237100** | **0.16436200** | **3.30198800** |
| **H** | **5.44837500** | **2.46134200** | **3.84036000** |
| **C** | **2.41950400** | **1.25353300** | **2.71263500** |
| **C** | **1.10006600** | **1.72111900** | **2.52289900** |
| **C** | **0.73950400** | **3.02158100** | **2.83219300** |
| **H** | **0.34103100** | **1.05781200** | **2.12243800** |
| **C** | **2.99020200** | **3.48949300** | **3.54917800** |
| **C** | **1.69051700** | **3.91642000** | **3.35187900** |
| **H** | **-0.28220700** | **3.34985500** | **2.66445900** |
| **H** | **3.73760900** | **4.17133400** | **3.94854900** |
| **H** | **1.40508300** | **4.93662100** | **3.59282100** |
| **C** | **4.03631500** | **-0.77964000** | **-3.04351200** |
| **C** | **4.73586000** | **-1.94473700** | **-3.47169400** |
| **C** | **2.07114500** | **-2.19961000** | **-2.61310700** |
| **C** | **4.12228200** | **-3.17354200** | **-3.46575300** |
| **H** | **5.76685000** | **-1.84205700** | **-3.80238200** |
| **C** | **2.77578900** | **-3.30282000** | **-3.02919800** |

| **H** | **1.04370000** | **-2.30025600** | **-2.27252900** |
| --- | --- | --- | --- |
| **H** | **4.66579200** | **-4.05576700** | **-3.79330200** |
| **H** | **2.30574400** | **-4.28214100** | **-3.02078200** |

Transition State of Expanded [11]-helicene:

X Y Z

**-----------------------------------------------------------------**

| **C** | **0.73605200** | **-3.32637700** | **0.21310500** |
| --- | --- | --- | --- |
| **C** | **-0.73722500** | **-3.32616700** | **0.21398600** |
| **C** | **1.41061000** | **-4.59178700** | **0.29602700** |
| **C** | **2.80082000** | **-4.63783300** | **0.20450000** |
| **C** | **3.57753900** | **-3.49370000** | **0.03988800** |
| **C** | **2.93917700** | **-2.20938700** | **0.02304200** |
| **C** | **1.53430600** | **-2.17207800** | **0.10068800** |
| **C** | **-1.53523800** | **-2.17165500** | **0.10219300** |
| **C** | **-2.94016500** | **-2.20856800** | **0.02547400** |
| **C** | **-3.57894300** | **-3.49265400** | **0.04353100** |
| **C** | **-2.80242900** | **-4.63698000** | **0.20782100** |
| **C** | **-1.41208700** | **-4.59137100** | **0.29765700** |
| **C** | **3.79494700** | **-1.01876800** | **-0.10037400** |
| **C** | **-3.79556100** | **-1.01776100** | **-0.09851900** |
| **C** | **5.20020800** | **-1.20653700** | **-0.33951500** |
| **C** | **6.02041200** | **-0.09537100** | **-0.52596900** |
| **C** | **5.53927200** | **1.21233500** | **-0.43147000** |
| **C** | **4.15997100** | **1.42359600** | **-0.10636100** |
| **C** | **3.32743900** | **0.29657100** | **0.02269700** |
| **C** | **-3.32748800** | **0.29748000** | **0.02325500** |
| **C** | **-4.15954200** | **1.42470800** | **-0.10686900** |
| **C** | **-5.53890300** | **1.21376000** | **-0.43188600** |
| **C** | **-6.02070200** | **-0.09383500** | **-0.52465200** |

| **C** | **-5.20096100** | **-1.20517700** | **-0.33714500** |
| --- | --- | --- | --- |
| **C** | **3.69776300** | **2.79700800** | **0.08954300** |
| **C** | **4.61017000** | **3.87535100** | **-0.12255100** |
| **C** | **-4.60821700** | **3.87668000** | **-0.12659900** |
| **C** | **-3.69678100** | **2.79804800** | **0.08806700** |
| **C** | **4.99885500** | **-3.61744400** | **-0.14600200** |
| **C** | **5.76974300** | **-2.52717200** | **-0.36118800** |
| **C** | **6.41649700** | **2.33060500** | **-0.63800400** |
| **C** | **5.96860100** | **3.60396900** | **-0.50537600** |
| **C** | **-5.96646400** | **3.60558400** | **-0.51032600** |
| **C** | **-6.41525000** | **2.33229400** | **-0.64075500** |
| **C** | **-5.77100200** | **-2.52561700** | **-0.35721900** |
| **C** | **-5.00042500** | **-3.61598200** | **-0.14136100** |
| **C** | **-0.67677800** | **-5.81838700** | **0.43570000** |
| **C** | **0.67506800** | **-5.81859500** | **0.43474700** |
| **C** | **4.18255700** | **5.20718200** | **0.06994100** |
| **C** | **2.89606400** | **5.48996500** | **0.48531300** |
| **C** | **2.39081000** | **3.11952700** | **0.51537800** |
| **C** | **-2.39022200** | **3.12019800** | **0.51536100** |
| **C** | **-2.89394400** | **5.49090300** | **0.48185800** |
| **C** | **-4.18000400** | **5.20846900** | **0.06487900** |
| **H** | **3.29464900** | **-5.60682800** | **0.23597600** |
| **H** | **1.04151600** | **-1.20788700** | **0.03881000** |
| **H** | **-1.04219300** | **-1.20762400** | **0.03989600** |
| **H** | **-3.29654800** | **-5.60579800** | **0.24016600** |
| **H** | **7.07651900** | **-0.25269600** | **-0.73541200** |
| **H** | **2.27779100** | **0.45053700** | **0.24392600** |
| **H** | **-2.27773300** | **0.45126200** | **0.24409100** |
| **H** | **-7.07692700** | **-0.25090500** | **-0.73370400** |
| **H** | **5.43075600** | **-4.61500500** | **-0.13572100** |
| **H** | **6.83928700** | **-2.62213300** | **-0.53094100** |
| **H** | **7.45118500** | **2.12905900** | **-0.90414500** |
| **H** | **6.63619100** | **4.44695300** | **-0.66607900** |
| **H** | **-6.63326500** | **4.44876200** | **-0.67326900** |
| **H** | **-7.44996800** | **2.13101800** | **-0.90698600** |
| **H** | **-6.84060600** | **-2.62037200** | **-0.52671600** |
| **H** | **-5.43272200** | **-4.61336000** | **-0.12994400** |
| **H** | **-1.23802400** | **-6.74524900** | **0.52275600** |
| **H** | **1.23613300** | **-6.74563100** | **0.52112700** |
| **H** | **4.89380400** | **6.01126200** | **-0.10399700** |
| **H** | **2.58027800** | **6.51790600** | **0.63991100** |
| **H** | **1.67449400** | **2.32832700** | **0.71084300** |
| **H** | **-1.67479300** | **2.32875100** | **0.71306400** |
| **H** | **-2.57795800** | **6.51878200** | **0.63645700** |
| **H** | **-4.89049400** | **6.01280400** | **-0.11097100** |
| **C** | **2.00101500** | **4.43258200** | **0.71597700** |
| **H** | **0.99672000** | **4.64636800** | **1.06441400** |
| **C** | **-1.99991500** | **4.43322200** | **0.71512200** |
| **H** | **-0.99598300** | **4.64670100** | **1.06480600** |

Transition State of Bor Doped Expanded [11]-helicene:

X Y Z

**-----------------------------------------------------------------**

| **C** | **1.09722200** | **-3.29664000** | **0.00005000** |
| --- | --- | --- | --- |
| **C** | **-0.35259700** | **-3.34673900** | **-0.00002000** |
| **C** | **1.82503200** | **-4.53103300** | **0.00021400** |
| **C** | **3.21860100** | **-4.49847100** | **0.00018900** |
| **C** | **3.93716400** | **-3.30178800** | **0.00002900** |
| **C** | **3.23015600** | **-2.05250900** | **-0.00006200** |
| **C** | **1.82553000** | **-2.09477100** | **-0.00009800** |
| **C** | **-2.89461600** | **-2.28651500** | **-0.00003200** |
| **C** | **-3.33561400** | **-3.67592200** | **-0.00000100** |
| **C** | **-2.43796900** | **-4.75509700** | **0.00005500** |
| **C** | **-1.03845500** | **-4.63557700** | **0.00008500** |

| **C** | **4.01595200** | **-0.81064100** | **-0.00014800** |
| --- | --- | --- | --- |
| **C** | **-3.87289700** | **-1.21628600** | **-0.00002000** |
| **C** | **5.44991400** | **-0.90140600** | **-0.00029500** |
| **C** | **6.21110000** | **0.26715100** | **-0.00028000** |
| **C** | **5.63043100** | **1.53810000** | **-0.00014800** |
| **C** | **4.20198200** | **1.65058700** | **-0.00009200** |
| **C** | **3.44345100** | **0.46768000** | **-0.00002200** |
| **C** | **-3.51677700** | **0.13882000** | **0.00003600** |
| **C** | **-4.46546400** | **1.17597400** | **0.00000000** |
| **C** | **-5.85487300** | **0.81874900** | **-0.00002700** |
| **C** | **-6.21475100** | **-0.53304700** | **-0.00006800** |
| **C** | **-5.26704500** | **-1.55428100** | **-0.00006700** |
| **C** | **3.60741700** | **2.98631800** | **-0.00003600** |
| **C** | **4.46395500** | **4.12861300** | **0.00030500** |
| **C** | **-5.15340500** | **3.57168900** | **0.00004800** |
| **C** | **-4.11172600** | **2.59482200** | **-0.00001700** |
| **C** | **5.37567100** | **-3.32974400** | **-0.00012500** |
| **C** | **6.09858800** | **-2.18536000** | **-0.00032700** |
| **C** | **6.44828500** | **2.71903200** | **0.00007800** |
| **C** | **5.89117300** | **3.95613500** | **0.00037400** |
| **C** | **-6.52910200** | **3.15613300** | **0.00008700** |
| **C** | **-6.86329500** | **1.84114900** | **0.00002800** |
| **C** | **-5.66760100** | **-2.93638500** | **-0.00008000** |
| **C** | **-4.75474800** | **-3.93719600** | **-0.00003800** |
| **C** | **-0.23401600** | **-5.83221700** | **0.00025000** |
| **C** | **1.11955600** | **-5.78387100** | **0.00033200** |
| **C** | **3.90516000** | **5.42554600** | **0.00046500** |
| **C** | **2.53644300** | **5.61316700** | **0.00022900** |
| **C** | **2.21355300** | **3.21200700** | **-0.00035800** |
| **C** | **-2.77767600** | **3.05870000** | **-0.00012400** |
| **C** | **-3.51248500** | **5.36772500** | **-0.00006100** |
| **C** | **-4.82773000** | **4.94586000** | **0.00003100** |
| **H** | **3.76613800** | **-5.43880900** | **0.00024100** |
| **H** | **1.26227300** | **-1.16558500** | **-0.00033400** |
| **H** | **-2.85617300** | **-5.75768200** | **0.00012600** |
| **H** | **7.29618200** | **0.18571400** | **-0.00030700** |
| **H** | **2.36337700** | **0.54766100** | **0.00027400** |
| **H** | **-2.45801500** | **0.38153500** | **0.00015400** |
| **H** | **-7.27094900** | **-0.79492900** | **-0.00006800** |
| **H** | **5.86746700** | **-4.29932700** | **-0.00011400** |
| **H** | **7.18536700** | **-2.21145400** | **-0.00048700** |
| **H** | **7.52849600** | **2.59570500** | **0.00006300** |
| **H** | **6.51649200** | **4.84560300** | **0.00062200** |
| **H** | **-7.29867000** | **3.92412700** | **0.00015900** |
| **H** | **-7.90594100** | **1.53290300** | **0.00004700** |
| **H** | **-6.73153100** | **-3.16043700** | **-0.00011200** |
| **H** | **-5.07328200** | **-4.97667100** | **-0.00003600** |
| **H** | **-0.75436300** | **-6.78673600** | **0.00032100** |
| **H** | **1.70476400** | **-6.70016800** | **0.00046200** |
| **H** | **4.57708600** | **6.28065700** | **0.00075800** |
| **H** | **2.11821200** | **6.61585200** | **0.00035400** |
| **H** | **1.52838200** | **2.37086400** | **-0.00079100** |
| **H** | **-1.95762300** | **2.34820600** | **-0.00019300** |
| **H** | **-3.27426400** | **6.42771300** | **-0.00007100** |
| **H** | **-5.63780600** | **5.67143100** | **0.00009600** |
| **C** | **1.68904200** | **4.49289100** | **-0.00021800** |
| **H** | **0.61269900** | **4.63041100** | **-0.00049100** |
| **C** | **-2.48347300** | **4.41146500** | **-0.00014100** |
| **H** | **-1.44831400** | **4.73733300** | **-0.00023300** |
| **B** | **-1.45487400** | **-2.41216500** | **-0.00006400** |

Transition State of Nitrogene Doped Expanded [11]-helicene:

X Y Z

**-----------------------------------------------------------------**

| **C** | **0.23550000** | **-3.37011500** | **0.37406900** |
| --- | --- | --- | --- |
| **C** | **-1.22789400** | **-3.24060800** | **0.33844100** |
| **C** | **0.78972600** | **-4.68402300** | **0.53059100** |
| **C** | **2.16711900** | **-4.85961800** | **0.40653300** |
| **C** | **3.02811700** | **-3.79992300** | **0.13114900** |
| **C** | **2.50930400** | **-2.46141200** | **0.07715000** |
| **C** | **1.11698200** | **-2.28925200** | **0.18866000** |
| **C** | **-3.13480600** | **-1.95104500** | **-0.01815100** |
| **C** | **-3.97647400** | **-3.11260400** | **-0.04366100** |
| **C** | **-3.38654100** | **-4.33991500** | **0.23823700** |
| **C** | **-2.01311500** | **-4.43942200** | **0.44371200** |
| **C** | **3.46856800** | **-1.36709700** | **-0.14199200** |
| **C** | **-3.77298100** | **-0.64379400** | **-0.20651400** |
| **C** | **4.81262200** | **-1.70501300** | **-0.52464900** |
| **C** | **5.70907600** | **-0.69016800** | **-0.85213500** |
| **C** | **5.38249100** | **0.66146700** | **-0.72213900** |
| **C** | **4.09959500** | **1.01802200** | **-0.19276700** |
| **C** | **3.16403200** | **-0.01032700** | **0.03357700** |
| **C** | **-3.09712400** | **0.56261100** | **-0.00085800** |
| **C** | **-3.72342500** | **1.81096200** | **-0.16317400** |
| **C** | **-5.07122300** | **1.83705100** | **-0.65292900** |
| **C** | **-5.75348600** | **0.63165800** | **-0.83960000** |
| **C** | **-5.15692700** | **-0.60253700** | **-0.58589100** |
| **C** | **3.84945400** | **2.42626700** | **0.11149300** |
| **C** | **4.82699400** | **3.40176000** | **-0.25443000** |
| **C** | **-3.77077400** | **4.30212800** | **-0.10829300** |
| **C** | **-3.08352600** | **3.08169300** | **0.16765600** |
| **C** | **4.41144200** | **-4.07008500** | **-0.15977800** |
| **C** | **5.24987300** | **-3.07525600** | **-0.52924300** |
| **C** | **6.32898400** | **1.67963400** | **-1.08299000** |
| **C** | **6.05142800** | **2.99243300** | **-0.88565300** |
| **C** | **-5.08642500** | **4.26897300** | **-0.68622200** |
| **C** | **-5.71649700** | **3.09146200** | **-0.92554600** |
| **C** | **-5.92735300** | **-1.81458600** | **-0.67431900** |
| **C** | **-5.37193600** | **-3.01278800** | **-0.37593300** |
| **C** | **-1.40106400** | **-5.71376100** | **0.69603500** |
| **C** | **-0.05389900** | **-5.82895700** | **0.74161200** |
| **C** | **4.61103900** | **4.76338800** | **0.04971800** |
| **C** | **3.48529500** | **5.16900100** | **0.73924100** |
| **C** | **2.70961100** | **2.87291500** | **0.81518700** |
| **C** | **-1.82838900** | **3.16288600** | **0.80738000** |
| **C** | **-1.94156600** | **5.58370100** | **0.85208300** |
| **C** | **-3.17206400** | **5.53637800** | **0.22590200** |
| **H** | **2.57668900** | **-5.86521100** | **0.47512400** |
| **H** | **0.67606200** | **-1.30460900** | **0.07490300** |
| **H** | **-3.99962400** | **-5.23878100** | **0.26764000** |
| **H** | **6.70702000** | **-0.95988600** | **-1.19181100** |
| **H** | **2.17072000** | **0.25187300** | **0.38059500** |
| **H** | **-2.06008700** | **0.49843800** | **0.30701300** |
| **H** | **-6.79023700** | **0.65898000** | **-1.16867100** |
| **H** | **4.75008300** | **-5.10233000** | **-0.12098900** |
| **H** | **6.28214500** | **-3.28377900** | **-0.79905900** |
| **H** | **7.27536700** | **1.36930500** | **-1.51902100** |
| **H** | **6.76851600** | **3.76030800** | **-1.16545800** |
| **H** | **-5.57968000** | **5.21460100** | **-0.89786600** |
| **H** | **-6.72622200** | **3.07269100** | **-1.32826400** |
| **H** | **-6.97458100** | **-1.73898600** | **-0.95588000** |
| **H** | **-5.95911600** | **-3.92737600** | **-0.40653300** |
| **H** | **-2.04593600** | **-6.58061600** | **0.81760900** |
| **H** | **0.42078600** | **-6.79346900** | **0.90267500** |
| **H** | **5.36435800** | **5.48841100** | **-0.24954400** |

| **H** | **3.33834100** | **6.21661700** | **0.98671900** |
| --- | --- | --- | --- |
| **H** | **1.96428300** | **2.15659600** | **1.14531500** |
| **H** | **-1.29374300** | **2.25390400** | **1.06189200** |
| **H** | **-1.49675300** | **6.53803600** | **1.12001300** |
| **H** | **-3.70943200** | **6.45403400** | **-0.00188400** |
| **C** | **2.53924600** | **4.20951000** | **1.13496400** |
| **H** | **1.67507500** | **4.51879600** | **1.71221300** |
| **C** | **-1.27610600** | **4.38384100** | **1.15306600** |
| **H** | **-0.32529700** | **4.41113000** | **1.67227100** |
| **N** | **-1.80373600** | **-2.03732500** | **0.15763600** |

Transition State of Silicone Doped Expanded [11]-helicene:

X Y Z

**-----------------------------------------------------------------**

| **C** | **1.44921000** | **-3.13707700** | **0.00854100** |
| --- | --- | --- | --- |
| **C** | **-0.00932900** | **-3.17479300** | **-0.00539700** |
| **C** | **2.18615300** | **-4.36509300** | **0.02092500** |
| **C** | **3.58299000** | **-4.32505500** | **0.04268500** |
| **C** | **4.29544700** | **-3.12273900** | **0.04802300** |
| **C** | **3.57426100** | **-1.88335400** | **0.02599700** |
| **C** | **2.17605000** | **-1.93654800** | **0.00953500** |
| **C** | **-2.83061500** | **-2.20037300** | **-0.02133600** |
| **C** | **-3.08401100** | **-3.61588300** | **-0.04484300** |
| **C** | **-2.08178000** | **-4.60303100** | **-0.04083600** |
| **C** | **-0.68350300** | **-4.44525000** | **-0.01993300** |
| **C** | **4.32977200** | **-0.62599700** | **0.02192400** |
| **C** | **-3.95557700** | **-1.26964300** | **-0.01884100** |
| **C** | **5.76294200** | **-0.67973500** | **0.05353900** |
| **C** | **6.48915600** | **0.51265100** | **0.05421600** |
| **C** | **5.86890000** | **1.76676800** | **0.02038300** |
| **C** | **4.43813900** | **1.83605700** | **-0.01839100** |
| **C** | **3.71847400** | **0.63204100** | **-0.01432800** |
| **C** | **-3.78659100** | **0.12102200** | **0.01720400** |
| **C** | **-4.85609900** | **1.02638100** | **0.01903300** |
| **C** | **-6.18847800** | **0.49728300** | **-0.02137800** |
| **C** | **-6.36888800** | **-0.89034000** | **-0.05473200** |
| **C** | **-5.29457800** | **-1.78144100** | **-0.05236600** |
| **C** | **3.79483200** | **3.14812100** | **-0.06142100** |
| **C** | **4.60979100** | **4.31920000** | **-0.05774800** |
| **C** | **-5.82359100** | **3.32033500** | **0.05368200** |
| **C** | **-4.67324300** | **2.47657400** | **0.06085900** |
| **C** | **5.73493600** | **-3.11614500** | **0.07579600** |
| **C** | **6.43358300** | **-1.95411600** | **0.08126100** |
| **C** | **6.64312100** | **2.97729700** | **0.02239200** |
| **C** | **6.04205600** | **4.19417200** | **-0.01371000** |
| **C** | **-7.13821300** | **2.73825100** | **0.00839200** |
| **C** | **-7.31235100** | **1.39200900** | **-0.02607000** |
| **C** | **-5.50191100** | **-3.20454700** | **-0.08065500** |
| **C** | **-4.45848400** | **-4.06903200** | **-0.07439700** |
| **C** | **0.11788100** | **-5.64981800** | **-0.01232300** |
| **C** | **1.47275700** | **-5.61311100** | **0.00991000** |
| **C** | **4.00286400** | **5.59405300** | **-0.09943600** |
| **C** | **2.62820400** | **5.73048200** | **-0.14572900** |
| **C** | **2.39330800** | **3.32107600** | **-0.10944900** |
| **C** | **-3.40319900** | **3.09346800** | **0.11061800** |
| **C** | **-4.40519100** | **5.29706500** | **0.14069900** |
| **C** | **-5.66174100** | **4.72306000** | **0.09322200** |
| **H** | **4.13492400** | **-5.26295000** | **0.05574300** |
| **H** | **1.61940100** | **-1.00662800** | **-0.00293800** |
| **H** | **-0.61900300** | **-0.36744800** | **0.01881300** |
| **H** | **-2.43675600** | **-5.63112200** | **-0.05539300** |
| **H** | **7.57604500** | **0.46568500** | **0.08043700** |

| **H** | **2.63766500** | **0.68028300** | **-0.04277200** |
| --- | --- | --- | --- |
| **H** | **-2.77563800** | **0.51073700** | **0.04612300** |
| **H** | **-7.38166300** | **-1.28781000** | **-0.08235100** |
| **H** | **6.25109600** | **-4.07293200** | **0.09399400** |
| **H** | **7.52053800** | **-1.96076100** | **0.10419800** |
| **H** | **7.72681500** | **2.89483100** | **0.05371000** |
| **H** | **6.63691500** | **5.10436100** | **-0.01156400** |
| **H** | **-7.99580300** | **3.40664600** | **0.00391100** |
| **H** | **-8.31093800** | **0.96301600** | **-0.05834900** |
| **H** | **-6.52420600** | **-3.57457800** | **-0.10515500** |
| **H** | **-4.63675800** | **-5.14127500** | **-0.09337400** |
| **H** | **-0.40307900** | **-6.60392400** | **-0.02283600** |
| **H** | **2.04913500** | **-6.53524100** | **0.01794500** |
| **H** | **4.64170800** | **6.47420100** | **-0.09546300** |
| **H** | **2.17538400** | **6.71753600** | **-0.17850500** |
| **H** | **1.74018300** | **2.45502300** | **-0.11595300** |
| **H** | **-2.50516300** | **2.48502100** | **0.12043600** |
| **H** | **-4.29628700** | **6.37762900** | **0.17155700** |
| **H** | **-6.55094700** | **5.34921500** | **0.08653000** |
| **C** | **1.81993700** | **4.58065400** | **-0.15123700** |
| **H** | **0.73813400** | **4.67787800** | **-0.19013200** |
| **C** | **-3.26816900** | **4.47096700** | **0.15013100** |
| **H** | **-2.27636800** | **4.91368400** | **0.19026000** |
| **Si** | **-1.10076200** | **-1.76187800** | **-0.00109500** |

Transition State of Phosphore Doped Expanded [11]-helicene:

X Y Z

**-----------------------------------------------------------------**

| **C** | **1.27653300** | **-3.19711800** | **-0.00019400** |
| --- | --- | --- | --- |
| **C** | **-0.19067900** | **-3.24095300** | **-0.00009800** |
| **C** | **2.01587800** | **-4.42500900** | **-0.00016400** |
| **C** | **3.41287700** | **-4.38496800** | **-0.00013600** |
| **C** | **4.12497100** | **-3.18436400** | **-0.00008900** |
| **C** | **3.40368300** | **-1.94457500** | **-0.00012600** |
| **C** | **2.00488400** | **-1.99548600** | **-0.00023900** |
| **C** | **-2.78668300** | **-2.24517700** | **0.00008400** |
| **C** | **-3.16644000** | **-3.62403400** | **0.00017500** |
| **C** | **-2.22401100** | **-4.65768600** | **0.00020000** |
| **C** | **-0.83144400** | **-4.51987800** | **0.00005800** |
| **C** | **4.16252300** | **-0.68819500** | **-0.00003300** |
| **C** | **-3.84857400** | **-1.23047200** | **0.00011400** |
| **C** | **5.59607700** | **-0.74706400** | **0.00003700** |
| **C** | **6.32809200** | **0.44110200** | **0.00019200** |
| **C** | **5.71309600** | **1.69785200** | **0.00020600** |
| **C** | **4.28218900** | **1.77331900** | **0.00003300** |
| **C** | **3.55568000** | **0.57251300** | **0.00000800** |
| **C** | **-3.58471000** | **0.14713600** | **0.00022800** |
| **C** | **-4.59136300** | **1.12210100** | **0.00015100** |
| **C** | **-5.95746800** | **0.68583000** | **-0.00002200** |
| **C** | **-6.23230500** | **-0.68508300** | **-0.00004900** |
| **C** | **-5.22148100** | **-1.64786500** | **0.00003000** |
| **C** | **3.64581600** | **3.08932000** | **-0.00011400** |
| **C** | **4.46720900** | **4.25616100** | **0.00014200** |
| **C** | **-5.40158500** | **3.47674800** | **-0.00006000** |
| **C** | **-4.31042000** | **2.55757300** | **0.00019900** |
| **C** | **5.56471300** | **-3.18300900** | **-0.00003100** |
| **C** | **6.26478300** | **-2.02257900** | **0.00002100** |
| **C** | **6.49426200** | **2.90372700** | **0.00044200** |
| **C** | **5.89925600** | **4.12391000** | **0.00045900** |
| **C** | **-6.75321700** | **2.98462500** | **-0.00023400** |
| **C** | **-7.01929600** | **1.65351300** | **-0.00018400** |
| **C** | **-5.54160500** | **-3.04924600** | **0.00007700** |

| **C** | **-4.56520100** | **-3.98667200** | **0.00019000** |
| --- | --- | --- | --- |
| **C** | **-0.03539000** | **-5.72483800** | **0.00001100** |
| **C** | **1.31789100** | **-5.68064500** | **-0.00011500** |
| **C** | **3.86720000** | **5.53485600** | **0.00000500** |
| **C** | **2.49272700** | **5.67893700** | **-0.00042500** |
| **C** | **2.24460900** | **3.26970200** | **-0.00058700** |
| **C** | **-3.00127700** | **3.08787900** | **0.00047200** |
| **C** | **-3.85455000** | **5.35495600** | **0.00011400** |
| **C** | **-5.14723700** | **4.86591300** | **-0.00011200** |
| **H** | **3.96391900** | **-5.32331400** | **-0.00015700** |
| **H** | **1.44601800** | **-1.06733600** | **-0.00041500** |
| **H** | **-2.61378300** | **-5.67388100** | **0.00028600** |
| **H** | **7.41503100** | **0.38866300** | **0.00033300** |
| **H** | **2.47462900** | **0.62489300** | **0.00011000** |
| **H** | **-2.54876700** | **0.46395300** | **0.00037100** |
| **H** | **-7.26976100** | **-1.01346800** | **-0.00011300** |
| **H** | **6.07816000** | **-4.14133800** | **-0.00003400** |
| **H** | **7.35201000** | **-2.03074700** | **0.00005800** |
| **H** | **7.57788400** | **2.81470800** | **0.00064000** |
| **H** | **6.49867600** | **5.03111200** | **0.00067500** |
| **H** | **-7.56333300** | **3.70990300** | **-0.00038000** |
| **H** | **-8.04480000** | **1.29235000** | **-0.00027300** |
| **H** | **-6.58960600** | **-3.33872800** | **0.00004500** |
| **H** | **-4.81505000** | **-5.04471500** | **0.00026300** |
| **H** | **-0.55728900** | **-6.67849800** | **0.00005700** |
| **H** | **1.90372200** | **-6.59655500** | **-0.00018200** |
| **H** | **4.51113100** | **6.41129400** | **0.00022700** |
| **H** | **2.04428500** | **6.66854400** | **-0.00054100** |
| **H** | **1.58619800** | **2.40768400** | **-0.00089100** |
| **H** | **-2.14605900** | **2.42067200** | **0.00075500** |
| **H** | **-3.67260300** | **6.42606600** | **0.00006900** |
| **H** | **-5.99305100** | **5.54954100** | **-0.00031700** |
| **C** | **1.67896900** | **4.53323900** | **-0.00074200** |
| **H** | **0.59738300** | **4.63636800** | **-0.00115400** |
| **C** | **-2.77613900** | **4.45420100** | **0.00042900** |
| **H** | **-1.75719700** | **4.83147700** | **0.00065200** |
| **P** | **-1.10098700** | **-1.73132200** | **-0.00013500** |

Transition State of Expanded [12]-helicene:

X Y Z

**-----------------------------------------------------------------**

| **C** | **-0.34743100** | **-5.16126700** | **0.74112700** |
| --- | --- | --- | --- |
| **C** | **-0.59308900** | **-3.76879600** | **0.48631400** |
| **C** | **0.52404000** | **-2.94406700** | **0.25252400** |
| **C** | **1.84205800** | **-3.43460900** | **0.18727300** |
| **C** | **2.04435500** | **-4.84385500** | **0.36433400** |
| **C** | **0.95573700** | **-5.65253400** | **0.68348000** |
| **C** | **-1.99286600** | **-3.31719400** | **0.40833700** |
| **C** | **3.01852300** | **-2.60674300** | **-0.11259900** |
| **C** | **-3.02843800** | **-4.30548100** | **0.52900200** |
| **C** | **-4.35302200** | **-3.94958300** | **0.27556500** |
| **C** | **-4.72363000** | **-2.65030100** | **-0.06308300** |
| **C** | **-3.73104100** | **-1.61613000** | **-0.04469600** |
| **C** | **-2.39129900** | **-1.98851600** | **0.16810900** |
| **C** | **3.02047800** | **-1.20206900** | **-0.08694600** |
| **C** | **4.14946200** | **-0.43521000** | **-0.41624600** |
| **C** | **5.31388900** | **-1.11575200** | **-0.89602500** |
| **C** | **5.34351200** | **-2.50870900** | **-0.86252700** |
| **C** | **4.25175500** | **-3.26674700** | **-0.43324100** |
| **C** | **-4.17685000** | **-0.23673300** | **-0.28452500** |
| **C** | **4.21289500** | **1.02489700** | **-0.27031500** |
| **C** | **-5.51314400** | **-0.02351100** | **-0.76842000** |

| **C** | **-5.92377800** | **1.26735800** | **-1.09743800** |
| --- | --- | --- | --- |
| **C** | **-5.11486300** | **2.38530800** | **-0.87583300** |
| **C** | **-3.83622300** | **2.20502900** | **-0.25516500** |
| **C** | **-3.38927300** | **0.89139100** | **-0.02368100** |
| **C** | **3.24876600** | **1.76487000** | **0.40597200** |
| **C** | **3.35650400** | **3.15955500** | **0.58665400** |
| **C** | **4.47446900** | **3.85923800** | **0.01830100** |
| **C** | **5.45099100** | **3.11080800** | **-0.66128400** |
| **C** | **5.36426400** | **1.72818200** | **-0.78823000** |
| **C** | **-3.08128100** | **3.39439400** | **0.13292200** |
| **C** | **-3.57722000** | **4.68270000** | **-0.23171900** |
| **C** | **-1.43177600** | **-6.07545100** | **0.97635600** |
| **C** | **-2.71592100** | **-5.66903500** | **0.85949100** |
| **C** | **-6.07450700** | **-2.36692100** | **-0.47105400** |
| **C** | **-6.43628700** | **-1.12320700** | **-0.86382200** |
| **C** | **-5.56672000** | **3.70189200** | **-1.23073100** |
| **C** | **-4.81943200** | **4.79799000** | **-0.94585200** |
| **C** | **3.62765900** | **5.95142700** | **0.92531400** |
| **C** | **4.57280600** | **5.26893700** | **0.19928900** |
| **C** | **6.44930700** | **0.98457200** | **-1.37846900** |
| **C** | **6.44222900** | **-0.36871300** | **-1.39328600** |
| **C** | **4.37731800** | **-4.69170400** | **-0.29101300** |
| **C** | **3.33502400** | **-5.43827500** | **0.14308200** |
| **C** | **-2.86691900** | **5.84189200** | **0.14991800** |
| **C** | **-1.71531900** | **5.75474500** | **0.90777600** |
| **C** | **-1.24248000** | **4.49132200** | **1.29979400** |
| **C** | **-1.90368800** | **3.33926900** | **0.91007800** |
| **H** | **0.35855000** | **-1.89181500** | **0.04637200** |
| **H** | **1.11997100** | **-6.71732600** | **0.83541700** |
| **H** | **-5.11759100** | **-4.72305700** | **0.30838300** |
| **H** | **-1.62853900** | **-1.21976100** | **0.10199100** |
| **H** | **2.11419500** | **-0.69000200** | **0.21766700** |
| **H** | **6.25314000** | **-3.02404800** | **-1.16425800** |
| **H** | **-6.92028100** | **1.41288500** | **-1.50960700** |
| **H** | **-2.40464300** | **0.74272800** | **0.40489300** |
| **H** | **2.39583400** | **1.26521400** | **0.85615800** |
| **H** | **6.32114600** | **3.62321400** | **-1.06684900** |
| **H** | **-1.18950600** | **-7.10969700** | **1.20725800** |
| **H** | **-3.53937900** | **-6.36606100** | **0.99284200** |
| **H** | **-6.78473900** | **-3.18961600** | **-0.49506000** |
| **H** | **-7.44423600** | **-0.91870900** | **-1.21602100** |
| **H** | **-6.52580900** | **3.79751400** | **-1.73381700** |
| **H** | **-5.16453000** | **5.79157100** | **-1.22133000** |
| **H** | **3.71515500** | **7.02571400** | **1.06275400** |
| **H** | **5.41805000** | **5.79331700** | **-0.24021700** |
| **H** | **7.29749900** | **1.54072300** | **-1.76991300** |
| **H** | **7.28623800** | **-0.92522000** | **-1.79326700** |
| **H** | **5.33936100** | **-5.14743000** | **-0.51139200** |
| **H** | **3.43571200** | **-6.51140500** | **0.28451400** |
| **H** | **-3.25703800** | **6.81228300** | **-0.14819700** |
| **H** | **-1.51900300** | **2.37968000** | **1.23984100** |
| **C** | **2.40390000** | **3.89969100** | **1.34148700** |
| **C** | **2.53460500** | **5.25672100** | **1.50987700** |
| **H** | **-0.35809400** | **4.41799500** | **1.92277600** |
| **H** | **1.57268900** | **3.36219100** | **1.78944300** |
| **H** | **-1.18547800** | **6.65434800** | **1.20829700** |
| **H** | **1.80303600** | **5.80831700** | **2.09425500** |

Transition State of Hydroxyl Functionalized Expanded [12]-helicene:

X Y Z

----------------------------------------------------------------- C 0.18601900 5.22851900 0.71936000

C 0.46914000 3.84190200 0.47107500

C -0.62397200 2.98987900 0.22254500

| **C** | **-1.95196900** | **3.44859100** | **0.13559800** |
| --- | --- | --- | --- |
| **C** | **-2.19047500** | **4.85348500** | **0.30081300** |
| **C** | **-1.12693000** | **5.68908600** | **0.63487900** |
| **C** | **1.88002600** | **3.42293500** | **0.41602700** |
| **C** | **-3.10487000** | **2.59041200** | **-0.17015800** |
| **C** | **2.88992900** | **4.43386500** | **0.56358100** |
| **C** | **4.22712000** | **4.10980100** | **0.33553300** |
| **C** | **4.63279700** | **2.82293700** | **-0.01097700** |
| **C** | **3.66300900** | **1.76687300** | **-0.02620200** |
| **C** | **2.31190000** | **2.10607900** | **0.16977500** |
| **C** | **-3.07235400** | **1.18649000** | **-0.13112800** |
| **C** | **-4.18162200** | **0.38867800** | **-0.45383400** |
| **C** | **-5.36268500** | **1.03575400** | **-0.93911100** |
| **C** | **-5.42399600** | **2.42802100** | **-0.92686000** |
| **C** | **-4.35162500** | **3.21717600** | **-0.50510100** |
| **C** | **4.14066900** | **0.40214300** | **-0.28759800** |
| **C** | **-4.21082100** | **-1.07063900** | **-0.28845600** |
| **C** | **5.48515000** | **0.22998500** | **-0.75611900** |
| **C** | **5.91397200** | **-1.04452400** | **-1.12013700** |
| **C** | **5.11766000** | **-2.17832300** | **-0.94358700** |
| **C** | **3.83022200** | **-2.04892400** | **-0.31764400** |
| **C** | **3.36388700** | **-0.74247400** | **-0.06081300** |
| **C** | **-3.21827600** | **-1.78143400** | **0.37821700** |
| **C** | **-3.29872700** | **-3.17341900** | **0.58904900** |
| **C** | **-4.41808000** | **-3.90317800** | **0.06311800** |
| **C** | **-5.41800400** | **-3.18614400** | **-0.61576500** |
| **C** | **-5.35664600** | **-1.80541800** | **-0.77461100** |
| **C** | **3.10720000** | **-3.27853600** | **0.02039700** |
| **C** | **3.63330900** | **-4.53677200** | **-0.42000100** |
| **C** | **1.24394700** | **6.16685200** | **0.97876400** |
| **C** | **2.53909600** | **5.78863900** | **0.89192200** |
| **C** | **5.99699100** | **2.57499800** | **-0.39700400** |
| **C** | **6.39027800** | **1.34684700** | **-0.80875300** |
| **C** | **5.59833100** | **-3.46127600** | **-1.36797700** |
| **C** | **4.87187500** | **-4.58260400** | **-1.14766700** |
| **C** | **-3.52343600** | **-5.96064900** | **1.00466400** |
| **C** | **-4.49238100** | **-5.30901900** | **0.28183000** |
| **C** | **-6.46123800** | **-1.09536700** | **-1.36971600** |
| **C** | **-6.47843900** | **0.25699700** | **-1.41534800** |
| **C** | **-4.51170600** | **4.64036200** | **-0.38063700** |
| **C** | **-3.49162700** | **5.41517700** | **0.05683300** |
| **C** | **2.98859200** | **-5.75302000** | **-0.11121300** |
| **C** | **1.85157500** | **-5.76520300** | **0.66972400** |
| **C** | **1.33807900** | **-4.55602600** | **1.14827500** |
| **C** | **1.93611200** | **-3.34261000** | **0.82777200** |
| **H** | **-0.42947800** | **1.94091300** | **0.02550800** |
| **H** | **-1.31896600** | **6.75016200** | **0.78027300** |
| **H** | **4.97345200** | **4.89967700** | **0.39137200** |
| **H** | **1.57003300** | **1.31944400** | **0.08276100** |
| **H** | **-2.15455500** | **0.70139900** | **0.18266900** |
| **H** | **-6.34529400** | **2.91762600** | **-1.23592000** |
| **H** | **6.91343400** | **-1.16657100** | **-1.53283900** |
| **H** | **2.38103000** | **-0.62244000** | **0.36602200** |
| **H** | **-2.36138400** | **-1.25945000** | **0.79425400** |
| **H** | **-6.28577200** | **-3.72167700** | **-0.99558800** |
| **H** | **0.97347000** | **7.19513700** | **1.20500700** |
| **H** | **3.34349100** | **6.50355100** | **1.04586200** |
| **H** | **6.69078300** | **3.41200800** | **-0.39098100** |
| **H** | **7.40810900** | **1.16896200** | **-1.14705200** |
| **H** | **6.55872200** | **-3.50879800** | **-1.87543400** |
| **H** | **5.23203700** | **-5.55302500** | **-1.47933800** |
| **H** | **-3.59360400** | **-7.03179100** | **1.17230700** |
| **H** | **-5.33935600** | **-5.85566500** | **-0.12594300** |

| **H** | **-7.30232300** | **-1.67559500** | **-1.74089500** |
| --- | --- | --- | --- |
| **H** | **-7.33556700** | **0.78891900** | **-1.82084800** |
| **H** | **-5.48180100** | **5.07113600** | **-0.61537800** |
| **H** | **-3.61909400** | **6.48697100** | **0.18657100** |
| **H** | **3.41890700** | **-6.68017900** | **-0.47935300** |
| **C** | **-2.31895500** | **-3.88402300** | **1.33675300** |
| **C** | **-2.42629000** | **-5.23720500** | **1.54504600** |
| **H** | **0.45499000** | **-4.55649500** | **1.78077100** |
| **H** | **-1.48187000** | **-3.32304400** | **1.74450000** |
| **H** | **1.35841300** | **-6.69937700** | **0.92248200** |
| **H** | **-1.67481100** | **-5.76482400** | **2.12640600** |
| **O** | **1.39988000** | **-2.17980000** | **1.32393400** |
| **H** | **0.69741300** | **-2.41273300** | **1.95052700** |

Transition State of Methyl Functionalized Expanded [12]-helicene:

X Y Z

**-----------------------------------------------------------------**

| **C** | **0.40760300** | **5.22775100** | **0.66555300** |
| --- | --- | --- | --- |
| **C** | **0.63401300** | **3.82818800** | **0.43463500** |
| **C** | **-0.49254500** | **3.01686000** | **0.20052300** |
| **C** | **-1.80194300** | **3.52546000** | **0.11263400** |
| **C** | **-1.98363300** | **4.94110800** | **0.25927300** |
| **C** | **-0.88636800** | **5.73873800** | **0.57754600** |
| **C** | **2.02659800** | **3.35207100** | **0.38747800** |
| **C** | **-2.98841000** | **2.70823500** | **-0.17719400** |
| **C** | **3.07847400** | **4.31881200** | **0.53778800** |
| **C** | **4.40326600** | **3.93220900** | **0.33472800** |
| **C** | **4.75687000** | **2.62464200** | **0.00783500** |
| **C** | **3.73904200** | **1.61539800** | **-0.02429400** |
| **C** | **2.40258300** | **2.01745900** | **0.14988100** |
| **C** | **-3.01071200** | **1.30419200** | **-0.12323900** |
| **C** | **-4.14998400** | **0.54622900** | **-0.43827700** |
| **C** | **-5.30533800** | **1.23495300** | **-0.92914500** |
| **C** | **-5.31342100** | **2.62855200** | **-0.92752700** |
| **C** | **-4.21045200** | **3.37966300** | **-0.51574500** |
| **C** | **4.15749500** | **0.22963700** | **-0.27112400** |
| **C** | **-4.23400300** | **-0.91078700** | **-0.26626500** |
| **C** | **5.50370800** | **-0.01255900** | **-0.70009300** |
| **C** | **5.86756600** | **-1.30532600** | **-1.07378200** |
| **C** | **5.00472400** | **-2.39502200** | **-0.93305800** |
| **C** | **3.72525700** | **-2.20740800** | **-0.30467900** |
| **C** | **3.32159800** | **-0.87789100** | **-0.07110100** |
| **C** | **-3.27279400** | **-1.65696400** | **0.40835800** |
| **C** | **-3.40221900** | **-3.04663000** | **0.61573600** |
| **C** | **-4.54337000** | **-3.73470600** | **0.07979500** |
| **C** | **-5.51416000** | **-2.98133600** | **-0.60193600** |
| **C** | **-5.40311600** | **-1.60367900** | **-0.75842600** |
| **C** | **2.92749500** | **-3.39584600** | **0.02712900** |
| **C** | **3.32708100** | **-4.64334600** | **-0.55741400** |
| **C** | **1.50320200** | **6.12448900** | **0.91617900** |
| **C** | **2.78223800** | **5.69136500** | **0.84574800** |
| **C** | **6.11748100** | **2.30762500** | **-0.33859700** |
| **C** | **6.46375200** | **1.05815600** | **-0.72902100** |
| **C** | **5.38467300** | **-3.68411200** | **-1.43411400** |
| **C** | **4.54895000** | **-4.74237400** | **-1.30951800** |
| **C** | **-3.72787900** | **-5.82543100** | **1.01727200** |
| **C** | **-4.66942100** | **-5.13779000** | **0.29177400** |
| **C** | **-6.48011100** | **-0.85494400** | **-1.35651300** |
| **C** | **-6.44903700** | **0.49698900** | **-1.40387600** |
| **C** | **-4.31450900** | **4.80939600** | **-0.40769300** |
| **C** | **-3.26316100** | **5.55007200** | **0.01453800** |
| **C** | **2.57597700** | **-5.81710700** | **-0.34499600** |

| **C** | **1.48172600** | **-5.80644000** | **0.49347600** |
| --- | --- | --- | --- |
| **C** | **1.15018100** | **-4.61950100** | **1.15584700** |
| **C** | **1.83349800** | **-3.42244700** | **0.94902100** |
| **H** | **-0.34167500** | **1.95808300** | **0.01851700** |
| **H** | **-1.03620200** | **6.80826400** | **0.70995800** |
| **H** | **5.18372600** | **4.68777300** | **0.39776500** |
| **H** | **1.62438400** | **1.26810800** | **0.05237800** |
| **H** | **-2.11249300** | **0.78598900** | **0.19455100** |
| **H** | **-6.21560500** | **3.15042300** | **-1.24017000** |
| **H** | **6.86095300** | **-1.47485100** | **-1.48434900** |
| **H** | **2.32086300** | **-0.69049700** | **0.28280600** |
| **H** | **-2.40361200** | **-1.16554900** | **0.83621600** |
| **H** | **-6.39881900** | **-3.48504000** | **-0.98647700** |
| **H** | **1.27490800** | **7.16619900** | **1.12696700** |
| **H** | **3.61445000** | **6.37400800** | **0.99801000** |
| **H** | **6.85055000** | **3.11019600** | **-0.31873400** |
| **H** | **7.48100400** | **0.82960100** | **-1.03702600** |
| **H** | **6.34010400** | **-3.77969800** | **-1.94388300** |
| **H** | **4.81321000** | **-5.71137100** | **-1.72559500** |
| **H** | **-3.83673900** | **-6.89421600** | **1.18014700** |
| **H** | **-5.53304500** | **-5.65211500** | **-0.12320000** |
| **H** | **-7.34066700** | **-1.40520200** | **-1.72881700** |
| **H** | **-7.28548600** | **1.05913600** | **-1.81175000** |
| **H** | **-5.26844000** | **5.27438100** | **-0.64345800** |
| **H** | **-3.34837500** | **6.62754800** | **0.13091500** |
| **H** | **2.89697300** | **-6.73615300** | **-0.82905200** |
| **C** | **-2.45050500** | **-3.79275600** | **1.36681000** |
| **C** | **-2.60949100** | **-5.14269400** | **1.56660400** |
| **H** | **0.33998900** | **-4.63157300** | **1.87800800** |
| **H** | **-1.59614700** | **-3.26817400** | **1.78543500** |
| **H** | **0.90642800** | **-6.71063100** | **0.67143200** |
| **H** | **-1.88043300** | **-5.69843400** | **2.15021500** |
| **C** | **1.39700500** | **-2.25030200** | **1.80467400** |
| **H** | **2.24629300** | **-1.68771100** | **2.20411700** |
| **H** | **0.76004500** | **-1.54002700** | **1.26087200** |
| **H** | **0.81100900** | **-2.61357700** | **2.65495100** |

Transition State of Methoxy Functionalized Expanded [12]-helicene:

X Y Z

**-----------------------------------------------------------------**

| **C** | **-0.17125900** | **5.27083600** | **0.66022400** |
| --- | --- | --- | --- |
| **C** | **0.18972000** | **3.90250500** | **0.41083000** |
| **C** | **-0.85367600** | **2.99133900** | **0.15895100** |
| **C** | **-2.20555200** | **3.37493300** | **0.07225700** |
| **C** | **-2.52191600** | **4.76478000** | **0.23483900** |
| **C** | **-1.50689200** | **5.65839500** | **0.56900300** |
| **C** | **1.62245300** | **3.56298600** | **0.36438500** |
| **C** | **-3.31040500** | **2.45180700** | **-0.22162800** |
| **C** | **2.57268100** | **4.62680600** | **0.53600100** |
| **C** | **3.92878700** | **4.37935100** | **0.32652300** |
| **C** | **4.41033200** | **3.12196700** | **-0.02939400** |
| **C** | **3.50088900** | **2.01417900** | **-0.07796700** |
| **C** | **2.13037500** | **2.27529600** | **0.10517600** |
| **C** | **-3.19525800** | **1.05247600** | **-0.18347800** |
| **C** | **-4.26321000** | **0.18998100** | **-0.47800400** |
| **C** | **-5.49479200** | **0.76658400** | **-0.92577200** |
| **C** | **-5.63281100** | **2.15340600** | **-0.92443400** |
| **C** | **-4.59626300** | **3.00512100** | **-0.53697900** |
| **C** | **4.05948000** | **0.68310300** | **-0.35670000** |
| **C** | **-4.19893700** | **-1.26863100** | **-0.31417400** |
| **C** | **5.42049900** | **0.59740100** | **-0.80490700** |
| **C** | **5.92810000** | **-0.64003100** | **-1.19438700** |

| **C** | **5.19795100** | **-1.82262300** | **-1.05740200** |
| --- | --- | --- | --- |
| **C** | **3.90211600** | **-1.78126900** | **-0.43950600** |
| **C** | **3.35033300** | **-0.51175000** | **-0.16695900** |
| **C** | **-3.12620900** | **-1.92182700** | **0.28391800** |
| **C** | **-3.11759300** | **-3.31495800** | **0.50266000** |
| **C** | **-4.23334200** | **-4.10499400** | **0.06330300** |
| **C** | **-5.30973900** | **-3.44768700** | **-0.55624300** |
| **C** | **-5.32899400** | **-2.06766500** | **-0.73075100** |
| **C** | **3.24972900** | **-3.05830000** | **-0.13009700** |
| **C** | **3.83153000** | **-4.26803700** | **-0.63530000** |
| **C** | **0.82989800** | **6.26511500** | **0.93481700** |
| **C** | **2.14469100** | **5.95847500** | **0.86710300** |
| **C** | **5.79285700** | **2.95902300** | **-0.39409700** |
| **C** | **6.26251800** | **1.76349900** | **-0.82004800** |
| **C** | **5.74715500** | **-3.06292600** | **-1.52482400** |
| **C** | **5.07170900** | **-4.22523300** | **-1.36090000** |
| **C** | **-3.17183000** | **-6.10126900** | **0.95738400** |
| **C** | **-4.22246200** | **-5.50965400** | **0.30026300** |
| **C** | **-6.49690000** | **-1.42467700** | **-1.27901100** |
| **C** | **-6.58588800** | **-0.07618300** | **-1.34670800** |
| **C** | **-4.83374700** | **4.41814200** | **-0.42158000** |
| **C** | **-3.85368100** | **5.25239200** | **-0.00274900** |
| **C** | **3.23714700** | **-5.52254500** | **-0.38464600** |
| **C** | **2.10424100** | **-5.62650000** | **0.39644000** |
| **C** | **1.55307500** | **-4.46536200** | **0.94959900** |
| **C** | **2.09878100** | **-3.21603600** | **0.69136600** |
| **H** | **-0.60153600** | **1.95399600** | **-0.03414700** |
| **H** | **-1.75803200** | **6.70692200** | **0.71535200** |
| **H** | **4.62937500** | **5.20837800** | **0.40309300** |
| **H** | **1.43372000** | **1.45086100** | **-0.00484300** |
| **H** | **-2.24541400** | **0.62227000** | **0.11373300** |
| **H** | **-6.58879100** | **2.58774600** | **-1.20998400** |
| **H** | **6.93638000** | **-0.69128300** | **-1.60037400** |
| **H** | **2.34499100** | **-0.47161500** | **0.22618900** |
| **H** | **-2.26791000** | **-1.35789700** | **0.63666000** |
| **H** | **-6.16994500** | **-4.03104800** | **-0.87879100** |
| **H** | **0.50018400** | **7.27599800** | **1.16092700** |
| **H** | **2.90695500** | **6.71482000** | **1.03639500** |
| **H** | **6.43720000** | **3.83403000** | **-0.36088400** |
| **H** | **7.29410000** | **1.64924100** | **-1.14361600** |
| **H** | **6.71149800** | **-3.04231700** | **-2.02638700** |
| **H** | **5.47698600** | **-5.16209800** | **-1.73453600** |
| **H** | **-3.17629600** | **-7.17215400** | **1.14190300** |
| **H** | **-5.06814000** | **-6.10292400** | **-0.03962500** |
| **H** | **-7.32298100** | **-2.05355600** | **-1.60174900** |
| **H** | **-7.48615200** | **0.40412400** | **-1.72210200** |
| **H** | **-5.82949300** | **4.79203900** | **-0.64636300** |
| **H** | **-4.03913100** | **6.31636000** | **0.12129200** |
| **H** | **3.70327400** | **-6.41050600** | **-0.80372000** |
| **C** | **-2.04423100** | **-3.96296000** | **1.17661200** |
| **C** | **-2.07356000** | **-5.31774500** | **1.40385400** |
| **H** | **0.68480000** | **-4.51835200** | **1.59445100** |
| **H** | **-1.20269500** | **-3.35673600** | **1.50133000** |
| **H** | **1.65101800** | **-6.59356700** | **0.59451400** |
| **H** | **-1.25563700** | **-5.80349500** | **1.92953000** |
| **O** | **1.46599100** | **-2.13020400** | **1.28186400** |
| **C** | **1.94622400** | **-1.82480200** | **2.59596600** |
| **H** | **3.00333500** | **-1.53345400** | **2.57488500** |
| **H** | **1.34520400** | **-0.98763300** | **2.95811900** |
| **H** | **1.82046200** | **-2.68389500** | **3.26687500** |

Transition State of Amino Functionalized Expanded [12]-helicene:

X Y Z

**-----------------------------------------------------------------**

| **C** | **0.35507200** | **5.23123200** | **0.64696000** |
| --- | --- | --- | --- |
| **C** | **0.59451900** | **3.83102800** | **0.43260700** |
| **C** | **-0.52204500** | **3.01161800** | **0.18011600** |
| **C** | **-1.83146100** | **3.51314400** | **0.05729200** |
| **C** | **-2.02287300** | **4.92972800** | **0.17943600** |
| **C** | **-0.93764900** | **5.73597600** | **0.51635900** |
| **C** | **1.99053700** | **3.36207500** | **0.42278400** |
| **C** | **-3.00956600** | **2.68549400** | **-0.23633300** |
| **C** | **3.03192100** | **4.33435000** | **0.60815400** |
| **C** | **4.36423500** | **3.95686500** | **0.44299300** |
| **C** | **4.73494900** | **2.65519000** | **0.11200900** |
| **C** | **3.72449100** | **1.64024500** | **0.03870300** |
| **C** | **2.38083200** | **2.03129500** | **0.18414400** |
| **C** | **-3.02372100** | **1.28292300** | **-0.15716900** |
| **C** | **-4.15931900** | **0.51278000** | **-0.45510400** |
| **C** | **-5.32166800** | **1.18600200** | **-0.95084400** |
| **C** | **-5.33302700** | **2.57915300** | **-0.98808300** |
| **C** | **-4.23241800** | **3.34411400** | **-0.59630600** |
| **C** | **4.15738400** | **0.26168000** | **-0.22454700** |
| **C** | **-4.23410500** | **-0.94047300** | **-0.25023100** |
| **C** | **5.51402200** | **0.03937600** | **-0.63286700** |
| **C** | **5.89508400** | **-1.23939600** | **-1.03936300** |
| **C** | **5.03953500** | **-2.33860300** | **-0.94645200** |
| **C** | **3.75192800** | **-2.17683700** | **-0.32882300** |
| **C** | **3.32625700** | **-0.85759100** | **-0.06388700** |
| **C** | **-3.24489300** | **-1.67124300** | **0.40022900** |
| **C** | **-3.37092800** | **-3.05293000** | **0.65417700** |
| **C** | **-4.53913100** | **-3.75106200** | **0.19593100** |
| **C** | **-5.53168000** | **-3.01604700** | **-0.47409900** |
| **C** | **-5.42001900** | **-1.64511700** | **-0.68206800** |
| **C** | **2.96567300** | **-3.38067000** | **-0.04787800** |
| **C** | **3.36293000** | **-4.61095000** | **-0.66473600** |
| **C** | **1.43768100** | **6.13402000** | **0.92969100** |
| **C** | **2.71984900** | **5.70557200** | **0.90644700** |
| **C** | **6.10609900** | **2.35350600** | **-0.20368200** |
| **C** | **6.46853100** | **1.11479800** | **-0.61302500** |
| **C** | **5.42813700** | **-3.61150200** | **-1.48318900** |
| **C** | **4.59451200** | **-4.67673900** | **-1.40558800** |
| **C** | **-3.69366600** | **-5.81182400** | **1.17228200** |
| **C** | **-4.66371900** | **-5.14433000** | **0.46578700** |
| **C** | **-6.50917100** | **-0.91443800** | **-1.28030000** |
| **C** | **-6.47366400** | **0.43433400** | **-1.38184500** |
| **C** | **-4.34252200** | **4.77552200** | **-0.52181800** |
| **C** | **-3.29982200** | **5.52882200** | **-0.10046500** |
| **C** | **2.60726400** | **-5.78824500** | **-0.49894900** |
| **C** | **1.50324700** | **-5.79181500** | **0.33179200** |
| **C** | **1.16682200** | **-4.63423600** | **1.03521600** |
| **C** | **1.87289100** | **-3.43984200** | **0.87246400** |
| **H** | **-0.36215500** | **1.95104000** | **0.01753500** |
| **H** | **-1.09558000** | **6.80603200** | **0.63446300** |
| **H** | **5.13785000** | **4.71662900** | **0.53396900** |
| **H** | **1.61107300** | **1.27740800** | **0.05767400** |
| **H** | **-2.12377900** | **0.77708600** | **0.17456400** |
| **H** | **-6.23853500** | **3.08991400** | **-1.30939100** |
| **H** | **6.89618800** | **-1.38739700** | **-1.43958700** |
| **H** | **2.30625600** | **-0.69920400** | **0.26014600** |
| **H** | **-2.35017600** | **-1.17668900** | **0.76665800** |
| **H** | **-6.43134700** | **-3.52777800** | **-0.81017800** |
| **H** | **1.19792100** | **7.17553000** | **1.12850500** |
| **H** | **3.54375800** | **6.39173100** | **1.08575400** |
| **H** | **6.83329100** | **3.15977600** | **-0.14904900** |

| **H** | **7.49389600** | **0.89833700** | **-0.90243700** |
| --- | --- | --- | --- |
| **H** | **6.39015000** | **-3.68822000** | **-1.98388100** |
| **H** | **4.86820100** | **-5.62922300** | **-1.85236600** |
| **H** | **-3.80172500** | **-6.87274700** | **1.38073800** |
| **H** | **-5.54853500** | **-5.66725000** | **0.11046900** |
| **H** | **-7.37972800** | **-1.47470800** | **-1.61229700** |
| **H** | **-7.31627400** | **0.98382200** | **-1.79432000** |
| **H** | **-5.29508900** | **5.23175700** | **-0.77927000** |
| **H** | **-3.39076400** | **6.60806700** | **-0.00683000** |
| **H** | **2.92554000** | **-6.69387400** | **-1.00784600** |
| **C** | **-2.38618500** | **-3.77924100** | **1.38113700** |
| **C** | **-2.54424500** | **-5.11919900** | **1.63954400** |
| **H** | **0.34642200** | **-4.65915200** | **1.74577500** |
| **H** | **-1.50567700** | **-3.24346600** | **1.72489500** |
| **H** | **0.91879200** | **-6.69661800** | **0.47380900** |
| **H** | **-1.79108100** | **-5.66134800** | **2.20531200** |
| **N** | **1.47218400** | **-2.33073700** | **1.64143900** |
| **H** | **0.93361100** | **-2.61106700** | **2.45469300** |
| **H** | **2.24079700** | **-1.73428900** | **1.93080300** |

Transition State of Carboxyl Functionalized Expanded [12]-helicene:

X Y Z

**-----------------------------------------------------------------**

| **C** | **0.05052300** | **5.28880400** | **0.64396900** |
| --- | --- | --- | --- |
| **C** | **0.35079600** | **3.90849800** | **0.38348700** |
| **C** | **-0.73244000** | **3.03722100** | **0.15933100** |
| **C** | **-2.07049800** | **3.47199000** | **0.10822800** |
| **C** | **-2.32915500** | **4.87182500** | **0.29018200** |
| **C** | **-1.27227200** | **5.72525300** | **0.59938000** |
| **C** | **1.76739500** | **3.51601500** | **0.29368400** |
| **C** | **-3.21420100** | **2.59492000** | **-0.18012700** |
| **C** | **2.76371200** | **4.54346300** | **0.42125100** |
| **C** | **4.10196400** | **4.24012900** | **0.17154600** |
| **C** | **4.52324500** | **2.95863600** | **-0.17592500** |
| **C** | **3.56888400** | **1.88909900** | **-0.18010900** |
| **C** | **2.21603100** | **2.20664700** | **0.03818700** |
| **C** | **-3.15486800** | **1.19085300** | **-0.16529800** |
| **C** | **-4.25601300** | **0.37801300** | **-0.48077600** |
| **C** | **-5.45965700** | **1.01297200** | **-0.92667400** |
| **C** | **-5.54640500** | **2.40313900** | **-0.88666400** |
| **C** | **-4.47874400** | **3.20442500** | **-0.47746700** |
| **C** | **4.06735600** | **0.53140500** | **-0.43684400** |
| **C** | **-4.25276100** | **-1.08584300** | **-0.34976500** |
| **C** | **5.41786100** | **0.37146000** | **-0.90028000** |
| **C** | **5.86461600** | **-0.89595600** | **-1.27239300** |
| **C** | **5.08373500** | **-2.04077500** | **-1.09488900** |
| **C** | **3.81314600** | **-1.92024500** | **-0.44201400** |
| **C** | **3.31220900** | **-0.62620600** | **-0.21409200** |
| **C** | **-3.22888600** | **-1.79395500** | **0.27185100** |
| **C** | **-3.28299700** | **-3.19296100** | **0.44941900** |
| **C** | **-4.40313300** | **-3.92949600** | **-0.06454100** |
| **C** | **-5.43243300** | **-3.21448900** | **-0.69988900** |
| **C** | **-5.39777700** | **-1.82951500** | **-0.82517900** |
| **C** | **3.10430200** | **-3.14580800** | **-0.07980900** |
| **C** | **3.53808500** | **-4.37663700** | **-0.66335100** |
| **C** | **1.09798300** | **6.24560600** | **0.87668700** |
| **C** | **2.39753600** | **5.89215000** | **0.75769000** |
| **C** | **5.88913200** | **2.72829400** | **-0.56639800** |
| **C** | **6.30395500** | **1.50267400** | **-0.96437400** |
| **C** | **5.52761700** | **-3.31713500** | **-1.57871100** |
| **C** | **4.75547600** | **-4.42158600** | **-1.42793400** |
| **C** | **-3.44772800** | **-5.98900000** | **0.80574900** |

| **C** | **-4.44760000** | **-5.34115900** | **0.12261200** |
| --- | --- | --- | --- |
| **C** | **-6.52966300** | **-1.12722900** | **-1.37610700** |
| **C** | **-6.57301200** | **0.22498900** | **-1.39277100** |
| **C** | **-4.66214900** | **4.62246100** | **-0.33005000** |
| **C** | **-3.64611600** | **5.41260800** | **0.08825500** |
| **C** | **2.81090600** | **-5.56602700** | **-0.44447200** |
| **C** | **1.69379900** | **-5.57908500** | **0.36622100** |
| **C** | **1.31500500** | **-4.40130700** | **1.02152300** |
| **C** | **2.01284000** | **-3.21169400** | **0.83289200** |
| **H** | **-0.52619700** | **1.99178000** | **-0.04390200** |
| **H** | **-1.47991100** | **6.78169700** | **0.75650900** |
| **H** | **4.83682300** | **5.04130100** | **0.21634700** |
| **H** | **1.48167600** | **1.41119400** | **-0.03227100** |
| **H** | **-2.22411400** | **0.71541100** | **0.12427500** |
| **H** | **-6.48368400** | **2.88080300** | **-1.16502600** |
| **H** | **6.85734700** | **-0.99840600** | **-1.70568600** |
| **H** | **2.29956200** | **-0.51498600** | **0.15259500** |
| **H** | **-2.36492200** | **-1.27607900** | **0.67772900** |
| **H** | **-6.30105800** | **-3.75508500** | **-1.07097300** |
| **H** | **0.81458600** | **7.26857500** | **1.11117600** |
| **H** | **3.19239600** | **6.62127700** | **0.89280000** |
| **H** | **6.56921200** | **3.57644600** | **-0.57079000** |
| **H** | **7.32397700** | **1.33899600** | **-1.30260400** |
| **H** | **6.47842500** | **-3.36776600** | **-2.10320900** |
| **H** | **5.06601400** | **-5.37856400** | **-1.83930600** |
| **H** | **-3.49515600** | **-7.06492900** | **0.95185400** |
| **H** | **-5.29551800** | **-5.89415500** | **-0.27496000** |
| **H** | **-7.36922300** | **-1.71518800** | **-1.73890800** |
| **H** | **-7.44905900** | **0.74989700** | **-1.76579900** |
| **H** | **-5.64627400** | **5.03690000** | **-0.53413500** |
| **H** | **-3.78949500** | **6.48045000** | **0.23284500** |
| **H** | **3.15755700** | **-6.48063500** | **-0.91924200** |
| **C** | **-2.26690400** | **-3.89646200** | **1.15616600** |
| **C** | **-2.35025000** | **-5.25667600** | **1.33399700** |
| **H** | **0.49210100** | **-4.41655700** | **1.72561700** |
| **H** | **1.13327800** | **-6.49498500** | **0.52664500** |
| **H** | **-1.57752600** | **-5.78486600** | **1.88730500** |
| **H** | **-1.43354400** | **-3.32353300** | **1.55106800** |
| **C** | **1.59188000** | **-2.10411500** | **1.74745700** |
| **O** | **0.45134400** | **-1.69959600** | **1.88070400** |
| **O** | **2.60843600** | **-1.64365200** | **2.51015700** |
| **H** | **2.22854600** | **-0.94413400** | **3.07597800** |

Transition State of Bor Doped Expanded [12]-helicene:

X Y Z

**-----------------------------------------------------------------**

| **C** | **-0.44058100** | **-5.12935500** | **0.48211100** |
| --- | --- | --- | --- |
| **C** | **-0.77446300** | **-3.71224500** | **0.34586100** |
| **C** | **1.95597900** | **-3.36938700** | **0.13775100** |
| **C** | **2.01647000** | **-4.82264900** | **0.24368200** |
| **C** | **0.87487200** | **-5.61446200** | **0.43722400** |
| **C** | **-2.16918400** | **-3.30408400** | **0.29266900** |
| **C** | **3.17939600** | **-2.61591900** | **-0.06644300** |
| **C** | **-3.17835100** | **-4.32062100** | **0.36630300** |
| **C** | **-4.51630400** | **-3.96871100** | **0.20322600** |
| **C** | **-4.91921000** | **-2.65230700** | **-0.01851800** |
| **C** | **-3.93874900** | **-1.60421400** | **-0.00963900** |
| **C** | **-2.58737600** | **-1.96957400** | **0.13345600** |
| **C** | **3.22989200** | **-1.21176800** | **-0.06130100** |
| **C** | **4.41545100** | **-0.49395700** | **-0.28155300** |
| **C** | **5.60268400** | **-1.22979000** | **-0.60030900** |
| **C** | **5.56888100** | **-2.62386300** | **-0.56730900** |

| **C** | **4.40265600** | **-3.33305400** | **-0.27548600** |
| --- | --- | --- | --- |
| **C** | **-4.41066000** | **-0.22188000** | **-0.17775400** |
| **C** | **4.51026800** | **0.96870500** | **-0.18681100** |
| **C** | **-5.79205600** | **-0.00384400** | **-0.51062600** |
| **C** | **-6.24375300** | **1.29312200** | **-0.74900500** |
| **C** | **-5.41615100** | **2.40950500** | **-0.60828400** |
| **C** | **-4.06312600** | **2.22182900** | **-0.17547800** |
| **C** | **-3.59684000** | **0.90560600** | **-0.00510900** |
| **C** | **3.47933800** | **1.76815600** | **0.29511200** |
| **C** | **3.60108900** | **3.16780900** | **0.41408200** |
| **C** | **4.81615600** | **3.80790700** | **-0.00516200** |
| **C** | **5.86125700** | **2.99952000** | **-0.48423900** |
| **C** | **5.74813700** | **1.61470900** | **-0.55963600** |
| **C** | **-3.24816000** | **3.40762500** | **0.08365000** |
| **C** | **-3.79323800** | **4.69935800** | **-0.18814100** |
| **C** | **-1.51676500** | **-6.07754100** | **0.62526400** |
| **C** | **-2.81336000** | **-5.69638200** | **0.56544900** |
| **C** | **-6.30227500** | **-2.36641800** | **-0.29214000** |
| **C** | **-6.71321500** | **-1.10687100** | **-0.56679800** |
| **C** | **-5.91681400** | **3.72981400** | **-0.87135600** |
| **C** | **-5.13509800** | **4.82313100** | **-0.68813300** |
| **C** | **3.89611200** | **5.96875900** | **0.62976100** |
| **C** | **4.92629900** | **5.22380400** | **0.11002300** |
| **C** | **6.87762400** | **0.81512700** | **-0.96277600** |
| **C** | **6.81682300** | **-0.53697400** | **-0.95139100** |
| **C** | **4.42208300** | **-4.76682200** | **-0.17810900** |
| **C** | **3.29699800** | **-5.46828000** | **0.09832100** |
| **C** | **-3.02231800** | **5.85505500** | **0.06419100** |
| **C** | **-1.75204000** | **5.76183800** | **0.59836900** |
| **C** | **-1.22032800** | **4.49530100** | **0.89176000** |
| **C** | **-1.94851900** | **3.34687200** | **0.63220600** |
| **H** | **1.01588300** | **-6.68836300** | **0.51927700** |
| **H** | **-5.27064700** | **-4.75268600** | **0.22045600** |
| **H** | **-1.82126600** | **-1.19991100** | **0.08538800** |
| **H** | **2.30580900** | **-0.67207800** | **0.12685900** |
| **H** | **6.48334600** | **-3.17781700** | **-0.76977000** |
| **H** | **-7.28315700** | **1.44226600** | **-1.03431100** |
| **H** | **-2.56596200** | **0.75436200** | **0.29335900** |
| **H** | **2.54944600** | **1.31666900** | **0.62836600** |
| **H** | **6.79864500** | **3.46761800** | **-0.77813600** |
| **H** | **-1.24992500** | **-7.12272200** | **0.75963100** |
| **H** | **-3.61126800** | **-6.42991600** | **0.65037200** |
| **H** | **-7.00306100** | **-3.19747500** | **-0.30252000** |
| **H** | **-7.75248400** | **-0.89665300** | **-0.80642300** |
| **H** | **-6.94095500** | **3.83005100** | **-1.22226000** |
| **H** | **-5.51800100** | **5.81989700** | **-0.89304900** |
| **H** | **3.99210500** | **7.04756700** | **0.71679300** |
| **H** | **5.84664800** | **5.70335200** | **-0.21487200** |
| **H** | **7.79408200** | **1.32833500** | **-1.24312200** |
| **H** | **7.68466200** | **-1.13430100** | **-1.21999400** |
| **H** | **5.37189500** | **-5.27708600** | **-0.31784800** |
| **H** | **3.32613900** | **-6.55130400** | **0.18779200** |
| **H** | **-3.45565800** | **6.82738700** | **-0.15823400** |
| **H** | **-1.51045600** | **2.38534700** | **0.87925000** |
| **C** | **2.56103400** | **3.97212400** | **0.95804100** |
| **C** | **2.70137700** | **5.33412200** | **1.06536200** |
| **H** | **-0.23358800** | **4.41695100** | **1.33388700** |
| **H** | **1.65354800** | **3.47820600** | **1.29377600** |
| **H** | **-1.17188800** | **6.65853500** | **0.79776900** |
| **H** | **1.90010500** | **5.93545300** | **1.48656300** |
| **B** | **0.53486800** | **-3.10492800** | **0.23735700** |

Transition State of Nitrogene Doped Expanded [12]-helicene:

X Y Z

**-----------------------------------------------------------------**

| **C** | **0.04581300** | **-5.21503300** | **0.89233900** |
| --- | --- | --- | --- |
| **C** | **-0.24878300** | **-3.83994700** | **0.59771400** |
| **C** | **1.99944600** | **-3.37433900** | **0.20272200** |
| **C** | **2.36261700** | **-4.75282600** | **0.37623300** |
| **C** | **1.36753900** | **-5.63605900** | **0.78178200** |
| **C** | **-1.66098600** | **-3.44293400** | **0.51634900** |
| **C** | **3.06343700** | **-2.43232400** | **-0.16164600** |
| **C** | **-2.65742600** | **-4.46275100** | **0.67870600** |
| **C** | **-3.98671300** | **-4.17024600** | **0.37212100** |
| **C** | **-4.38561100** | **-2.90578600** | **-0.05592500** |
| **C** | **-3.43368600** | **-1.83165600** | **-0.05194300** |
| **C** | **-2.08565100** | **-2.13966200** | **0.20315200** |
| **C** | **2.90154300** | **-1.03885800** | **-0.13311000** |
| **C** | **3.92934400** | **-0.15948100** | **-0.50899400** |
| **C** | **5.12480200** | **-0.71422800** | **-1.07247800** |
| **C** | **5.30860300** | **-2.09553500** | **-1.05281000** |
| **C** | **4.33569800** | **-2.96446300** | **-0.55338900** |
| **C** | **-3.93084900** | **-0.48438500** | **-0.35914600** |
| **C** | **3.86795900** | **1.29644800** | **-0.32580000** |
| **C** | **-5.23650900** | **-0.35337100** | **-0.94420200** |
| **C** | **-5.68071100** | **0.90449800** | **-1.34857500** |
| **C** | **-4.95494800** | **2.06890600** | **-1.08023200** |
| **C** | **-3.73811700** | **1.97004400** | **-0.33070300** |
| **C** | **-3.23072300** | **0.68763400** | **-0.05110200** |
| **C** | **2.90831300** | **1.92079100** | **0.46374200** |
| **C** | **2.91896200** | **3.31140000** | **0.70368200** |
| **C** | **3.91519400** | **4.12854900** | **0.06880200** |
| **C** | **4.88123800** | **3.49707300** | **-0.73336400** |
| **C** | **4.90363300** | **2.11779100** | **-0.90949000** |
| **C** | **-3.11774400** | **3.20398200** | **0.14381800** |
| **C** | **-3.64628800** | **4.45865600** | **-0.28557000** |
| **C** | **-0.99732500** | **-6.15438300** | **1.19813900** |
| **C** | **-2.29649600** | **-5.79523700** | **1.08117400** |
| **C** | **-5.72066900** | **-2.70057200** | **-0.55483300** |
| **C** | **-6.10224500** | **-1.49837900** | **-1.04688500** |
| **C** | **-5.43846600** | **3.35371200** | **-1.50395000** |
| **C** | **-4.79412200** | **4.49456300** | **-1.15032600** |
| **C** | **3.00751700** | **6.09772100** | **1.16999500** |
| **C** | **3.92082100** | **5.53155900** | **0.31484700** |
| **C** | **6.00157500** | **1.49478000** | **-1.60585300** |
| **C** | **6.12984200** | **0.14819200** | **-1.64306200** |
| **C** | **4.61875500** | **-4.36765700** | **-0.42021500** |
| **C** | **3.68866300** | **-5.21744900** | **0.07659300** |
| **C** | **-3.07375700** | **5.65932900** | **0.18822000** |
| **C** | **-2.03892800** | **5.64217800** | **1.10328200** |
| **C** | **-1.54432300** | **4.40983900** | **1.56210600** |
| **C** | **-2.06093300** | **3.21965400** | **1.07992700** |
| **H** | **1.61396400** | **-6.68187500** | **0.95565900** |
| **H** | **-4.72512500** | **-4.96783100** | **0.42111600** |
| **H** | **-1.32211400** | **-1.37537200** | **0.10317800** |
| **H** | **1.94371900** | **-0.65613000** | **0.20184100** |
| **H** | **6.24585400** | **-2.50885300** | **-1.41968200** |
| **H** | **-6.64416700** | **0.98798000** | **-1.84749300** |
| **H** | **-2.27791100** | **0.59668000** | **0.45853900** |
| **H** | **2.14841000** | **1.32671300** | **0.96317100** |
| **H** | **5.66430800** | **4.09955400** | **-1.18949100** |
| **H** | **-0.71467900** | **-7.16835300** | **1.47028400** |
| **H** | **-3.09408700** | **-6.51241700** | **1.25759500** |
| **H** | **-6.39635700** | **-3.55200400** | **-0.57318900** |
| **H** | **-7.09141200** | **-1.35817400** | **-1.47566300** |

| **H** | **-6.33815200** | **3.38863000** | **-2.11346000** |
| --- | --- | --- | --- |
| **H** | **-5.16275000** | **5.46386400** | **-1.47714900** |
| **H** | **3.02569600** | **7.16762100** | **1.35891000** |
| **H** | **4.67283300** | **6.14408400** | **-0.17698300** |
| **H** | **6.75617800** | **2.13802400** | **-2.05180300** |
| **H** | **6.99177800** | **-0.31612400** | **-2.11581600** |
| **H** | **5.60752000** | **-4.72193400** | **-0.70030700** |
| **H** | **3.90675600** | **-6.27391400** | **0.21230800** |
| **H** | **-3.48275000** | **6.60428100** | **-0.16188600** |
| **H** | **-1.66727300** | **2.28408600** | **1.46366600** |
| **C** | **1.99486200** | **3.93368300** | **1.58895100** |
| **C** | **2.03967100** | **5.28712300** | **1.82120100** |
| **H** | **-0.75939500** | **4.38984500** | **2.30992000** |
| **H** | **1.25688400** | **3.31006100** | **2.08588500** |
| **H** | **-1.61976200** | **6.57248600** | **1.47631600** |
| **H** | **1.33428600** | **5.74753900** | **2.50762500** |
| **N** | **0.72638800** | **-2.95310300** | **0.32070700** |

Transition State of Silicone Doped Expanded [12]-helicene:

X Y Z

**-----------------------------------------------------------------**

| **C** | **-0.61247000** | **-4.90145400** | **-0.00123000** |
| --- | --- | --- | --- |
| **C** | **-0.99322100** | **-3.51257400** | **-0.00084900** |
| **C** | **1.98580900** | **-3.19228900** | **-0.00055900** |
| **C** | **1.91209600** | **-4.62967900** | **-0.00085500** |
| **C** | **0.71423700** | **-5.36496600** | **-0.00124300** |
| **C** | **-2.41328600** | **-3.16346200** | **-0.00058100** |
| **C** | **3.29716400** | **-2.54820300** | **-0.00008000** |
| **C** | **-3.39030700** | **-4.21245700** | **-0.00063300** |
| **C** | **-4.74777600** | **-3.88788800** | **-0.00008800** |
| **C** | **-5.19985000** | **-2.56732600** | **0.00044200** |
| **C** | **-4.24178500** | **-1.49955900** | **0.00026700** |
| **C** | **-2.88094100** | **-1.83779100** | **-0.00020500** |
| **C** | **3.46613800** | **-1.15277800** | **-0.00002600** |
| **C** | **4.71962900** | **-0.52959800** | **0.00060300** |
| **C** | **5.88733800** | **-1.36003000** | **0.00138900** |
| **C** | **5.73408900** | **-2.74689400** | **0.00118800** |
| **C** | **4.47798200** | **-3.35947400** | **0.00040200** |
| **C** | **-4.73647000** | **-0.11650200** | **0.00060900** |
| **C** | **4.89318700** | **0.92991500** | **0.00050200** |
| **C** | **-6.15446400** | **0.10943000** | **0.00145300** |
| **C** | **-6.63847700** | **1.41793300** | **0.00194100** |
| **C** | **-5.79019600** | **2.52946800** | **0.00146300** |
| **C** | **-4.37220800** | **2.32340700** | **0.00036100** |
| **C** | **-3.89521100** | **1.00259100** | **0.00008900** |
| **C** | **3.82830300** | **1.82526100** | **-0.00068400** |
| **C** | **4.01305700** | **3.22286600** | **-0.00079400** |
| **C** | **5.34694500** | **3.75509600** | **0.00041500** |
| **C** | **6.42364900** | **2.85109000** | **0.00157000** |
| **C** | **6.23189600** | **1.47254200** | **0.00158900** |
| **C** | **-3.49361100** | **3.49207500** | **-0.00046300** |
| **C** | **-4.07382700** | **4.79621100** | **0.00016400** |
| **C** | **-1.64962100** | **-5.90913400** | **-0.00150600** |
| **C** | **-2.96369100** | **-5.58425400** | **-0.00120000** |
| **C** | **-6.60995700** | **-2.28022600** | **0.00120300** |
| **C** | **-7.06581700** | **-1.00469200** | **0.00175700** |
| **C** | **-6.32346900** | **3.86345300** | **0.00206100** |
| **C** | **-5.50397500** | **4.94520600** | **0.00150200** |
| **C** | **4.44351800** | **6.01421200** | **-0.00094600** |
| **C** | **5.52594100** | **5.16874000** | **0.00032300** |
| **C** | **7.36386200** | **0.57923100** | **0.00259100** |
| **C** | **7.19995500** | **-0.76487200** | **0.00241800** |

| **C** | **4.35598900** | **-4.79070700** | **0.00006800** |
| --- | --- | --- | --- |
| **C** | **3.14171300** | **-5.39037400** | **-0.00060000** |
| **C** | **-3.23885700** | **5.93526500** | **-0.00064100** |
| **C** | **-1.86266400** | **5.81199800** | **-0.00210300** |
| **C** | **-1.28551000** | **4.53094800** | **-0.00280000** |
| **C** | **-2.08404400** | **3.40008800** | **-0.00198900** |
| **H** | **0.83056600** | **-6.44629500** | **-0.00151500** |
| **H** | **-5.47938200** | **-4.69352100** | **-0.00005200** |
| **H** | **-2.14588600** | **-1.04022200** | **-0.00026500** |
| **H** | **2.57622200** | **-0.53307200** | **-0.00044600** |
| **H** | **6.62305800** | **-3.37464600** | **0.00168700** |
| **H** | **-7.71464000** | **1.57914700** | **0.00271300** |
| **H** | **-2.82438400** | **0.84253300** | **-0.00052700** |
| **H** | **2.80573300** | **1.46017900** | **-0.00161700** |
| **H** | **7.43965900** | **3.24091900** | **0.00244100** |
| **H** | **-1.34190300** | **-6.95165100** | **-0.00194400** |
| **H** | **-3.72597900** | **-6.35971500** | **-0.00138500** |
| **H** | **-7.30258800** | **-3.11827100** | **0.00138000** |
| **H** | **-8.13248400** | **-0.79464100** | **0.00239000** |
| **H** | **-7.40389200** | **3.98509100** | **0.00300700** |
| **H** | **-5.91566800** | **5.95155400** | **0.00200100** |
| **H** | **4.59165400** | **7.09063500** | **-0.00100700** |
| **H** | **6.53816900** | **5.56621200** | **0.00126400** |
| **H** | **8.35925400** | **1.01647900** | **0.00343600** |
| **H** | **8.06126500** | **-1.42841600** | **0.00312500** |
| **H** | **5.26665000** | **-5.38497300** | **0.00033300** |
| **H** | **3.06553400** | **-6.47471100** | **-0.00089600** |
| **H** | **-3.70221200** | **6.91914700** | **-0.00011100** |
| **H** | **-1.60445400** | **2.42703100** | **-0.00261000** |
| **C** | **2.91627900** | **4.13118800** | **-0.00211300** |
| **C** | **3.12268900** | **5.48898000** | **-0.00219600** |
| **H** | **-0.20470400** | **4.42556800** | **-0.00402100** |
| **H** | **1.90819700** | **3.72394900** | **-0.00307900** |
| **H** | **-1.23194700** | **6.69668500** | **-0.00273200** |
| **H** | **2.27616800** | **6.17038000** | **-0.00322100** |
| **Si** | **0.39119300** | **-2.38020300** | **-0.00069500** |
| **H** | **0.23272600** | **-0.91260800** | **-0.00052100** |

Transition State of Phosphore Doped Expanded [12]-helicene:

X Y Z

**-----------------------------------------------------------------**

| **C** | **0.46866700** | **-4.98009600** | **0.21890200** |
| --- | --- | --- | --- |
| **C** | **0.80316800** | **-3.58833500** | **0.15069700** |
| **C** | **-1.96819500** | **-3.23384400** | **0.03850800** |
| **C** | **-2.00638100** | **-4.66400300** | **0.07897200** |
| **C** | **-0.84961900** | **-5.43930300** | **0.18211100** |
| **C** | **2.23017300** | **-3.22289100** | **0.14029600** |
| **C** | **-3.24919000** | **-2.51701200** | **-0.04820600** |
| **C** | **3.21078200** | **-4.26977800** | **0.19729500** |
| **C** | **4.56679400** | **-3.95080800** | **0.13315700** |
| **C** | **5.02036300** | **-2.63881300** | **0.02094200** |
| **C** | **4.06671600** | **-1.56611700** | **0.00592800** |
| **C** | **2.70252000** | **-1.89724300** | **0.05823600** |
| **C** | **-3.35693900** | **-1.11366200** | **-0.03280700** |
| **C** | **-4.57969000** | **-0.43621800** | **-0.12922200** |
| **C** | **-5.77551900** | **-1.21104000** | **-0.27961100** |
| **C** | **-5.68553300** | **-2.60117200** | **-0.27756100** |
| **C** | **-4.46570800** | **-3.27050100** | **-0.15180400** |
| **C** | **4.57812900** | **-0.18889700** | **-0.07821700** |
| **C** | **-4.69883600** | **1.02828000** | **-0.07763500** |
| **C** | **5.99359000** | **0.00972100** | **-0.22796400** |
| **C** | **6.49733300** | **1.30374300** | **-0.34723800** |

| **C** | **5.67643800** | **2.43271700** | **-0.29294900** |
| --- | --- | --- | --- |
| **C** | **4.26819500** | **2.26008700** | **-0.09278800** |
| **C** | **3.76470800** | **0.94938800** | **-0.00681400** |
| **C** | **-3.61628900** | **1.87479000** | **0.13468100** |
| **C** | **-3.75395900** | **3.27684700** | **0.19418900** |
| **C** | **-5.05072100** | **3.86738600** | **0.01879300** |
| **C** | **-6.14570800** | **3.01203500** | **-0.19272300** |
| **C** | **-6.00388300** | **1.62850900** | **-0.23499200** |
| **C** | **3.43146400** | **3.45393500** | **0.02128200** |
| **C** | **4.03552300** | **4.74132500** | **-0.11125200** |
| **C** | **1.50276500** | **-5.98224700** | **0.30917800** |
| **C** | **2.81061400** | **-5.64419700** | **0.29800000** |
| **C** | **6.42963700** | **-2.37648500** | **-0.09918300** |
| **C** | **6.89296900** | **-1.11306000** | **-0.23833500** |
| **C** | **6.23483600** | **3.74945000** | **-0.42350700** |
| **C** | **5.44923800** | **4.85242100** | **-0.34506200** |
| **C** | **-4.08059500** | **6.08011400** | **0.30339100** |
| **C** | **-5.17771200** | **5.28530600** | **0.07698100** |
| **C** | **-7.16019400** | **0.78752000** | **-0.41910000** |
| **C** | **-7.05333400** | **-0.56155100** | **-0.42889300** |
| **C** | **-4.43559800** | **-4.70488100** | **-0.12177300** |
| **C** | **-3.26382800** | **-5.36774100** | **0.00300600** |
| **C** | **3.24365500** | **5.90486600** | **0.00191100** |
| **C** | **1.88809100** | **5.82260900** | **0.25316400** |
| **C** | **1.29026700** | **4.55954400** | **0.39563400** |
| **C** | **2.04365800** | **3.40412000** | **0.27786000** |
| **H** | **-0.98934700** | **-6.51764400** | **0.22354400** |
| **H** | **5.29540700** | **-4.75853100** | **0.15664800** |
| **H** | **1.96819400** | **-1.10018200** | **0.01430300** |
| **H** | **-2.44300400** | **-0.53776900** | **0.05863000** |
| **H** | **-6.59643000** | **-3.18857300** | **-0.37350600** |
| **H** | **7.56917300** | **1.43938400** | **-0.47673000** |
| **H** | **2.69905300** | **0.81245400** | **0.13152300** |
| **H** | **-2.62036300** | **1.46546000** | **0.27416400** |
| **H** | **-7.13693900** | **3.44352800** | **-0.31709300** |
| **H** | **1.19743700** | **-7.02331700** | **0.37519400** |
| **H** | **3.58599500** | **-6.40398200** | **0.35524900** |
| **H** | **7.11043100** | **-3.22396600** | **-0.09128900** |
| **H** | **7.95677000** | **-0.91684300** | **-0.34647600** |
| **H** | **7.30590500** | **3.83923500** | **-0.58719700** |
| **H** | **5.87725400** | **5.84677500** | **-0.44630200** |
| **H** | **-4.18872800** | **7.16040700** | **0.34698200** |
| **H** | **-6.16215200** | **5.72692000** | **-0.05877900** |
| **H** | **-8.12795400** | **1.26858500** | **-0.53766700** |
| **H** | **-7.93285700** | **-1.18804100** | **-0.55505700** |
| **H** | **-5.37754600** | **-5.24296400** | **-0.19280900** |
| **H** | **-3.24072500** | **-6.45397200** | **0.03629500** |
| **H** | **3.72550000** | **6.87378700** | **-0.10632100** |
| **H** | **1.54825700** | **2.44621300** | **0.39638300** |
| **C** | **-2.64250500** | **4.13301000** | **0.43141900** |
| **C** | **-2.79748700** | **5.49649000** | **0.48534200** |
| **H** | **0.22870200** | **4.48884000** | **0.60546100** |
| **H** | **-1.66634500** | **3.67742100** | **0.57132000** |
| **H** | **1.28982500** | **6.72502600** | **0.34402200** |
| **H** | **-1.93944000** | **6.13791100** | **0.66812400** |
| **P** | **-0.44221900** | **-2.33613900** | **0.05986300** |

Transition State of Expanded [13]-helicene:

X Y Z

----------------------------------------------------------------- C -5.28009100 1.10807000 1.22088900

C -3.91493500 0.66606000 1.23643100

| **C** | **-3.26657300** | **0.48810600** | **0.00000000** |
| --- | --- | --- | --- |
| **C** | **-3.91493500** | **0.66606000** | **-1.23643100** |
| **C** | **-5.28009100** | **1.10807000** | **-1.22088900** |
| **C** | **-5.90378300** | **1.36295200** | **0.00000000** |
| **C** | **-3.31989700** | **0.30804700** | **2.53171100** |
| **C** | **-3.31989700** | **0.30804700** | **-2.53171100** |
| **C** | **-4.19116200** | **0.20280500** | **3.66816000** |
| **C** | **-3.71728700** | **-0.38818400** | **4.84125200** |
| **C** | **-2.39099700** | **-0.79555900** | **4.98228300** |
| **C** | **-1.45740900** | **-0.47463000** | **3.94417600** |
| **C** | **-1.95816300** | **0.02197000** | **2.72917600** |
| **C** | **-1.95816300** | **0.02197000** | **-2.72917600** |
| **C** | **-1.45740900** | **-0.47463000** | **-3.94417600** |
| **C** | **-2.39099700** | **-0.79555900** | **-4.98228300** |
| **C** | **-3.71728700** | **-0.38818400** | **-4.84125200** |
| **C** | **-4.19116200** | **0.20280500** | **-3.66816000** |
| **C** | **-0.03419400** | **-0.69590200** | **4.21505400** |
| **C** | **-0.03419400** | **-0.69590200** | **-4.21505400** |
| **C** | **0.33325900** | **-1.50789700** | **5.34048900** |
| **C** | **1.68061800** | **-1.79455900** | **5.56025500** |
| **C** | **2.69844300** | **-1.20665200** | **4.80093900** |
| **C** | **2.34851100** | **-0.25860600** | **3.78532800** |
| **C** | **0.98759100** | **-0.08260800** | **3.48271600** |
| **C** | **0.98759100** | **-0.08260800** | **-3.48271600** |
| **C** | **2.34851100** | **-0.25860600** | **-3.78532800** |
| **C** | **2.69844300** | **-1.20665200** | **-4.80093900** |
| **C** | **1.68061800** | **-1.79455900** | **-5.56025500** |
| **C** | **0.33325900** | **-1.50789700** | **-5.34048900** |
| **C** | **3.41568400** | **0.51383600** | **3.15573800** |
| **C** | **4.77360700** | **0.20381100** | **3.46570800** |
| **C** | **4.77360700** | **0.20381100** | **-3.46570800** |
| **C** | **3.41568400** | **0.51383600** | **-3.15573800** |
| **C** | **-6.04831600** | **1.16673500** | **2.43621000** |
| **C** | **-5.54497400** | **0.68259700** | **3.59576800** |
| **C** | **-1.96360700** | **-1.54638300** | **6.13468600** |
| **C** | **-0.67610500** | **-1.94808100** | **6.26856400** |
| **C** | **4.08084100** | **-1.50024500** | **5.06085300** |
| **C** | **5.07151700** | **-0.84507300** | **4.40319000** |
| **C** | **5.07151700** | **-0.84507300** | **-4.40319000** |
| **C** | **4.08084100** | **-1.50024500** | **-5.06085300** |
| **C** | **-0.67610500** | **-1.94808100** | **-6.26856400** |
| **C** | **-1.96360700** | **-1.54638300** | **-6.13468600** |
| **C** | **-5.54497400** | **0.68259700** | **-3.59576800** |
| **C** | **-6.04831600** | **1.16673500** | **-2.43621000** |
| **C** | **5.81389400** | **0.96493100** | **2.88861300** |
| **C** | **5.53852100** | **2.03569200** | **2.05971300** |
| **C** | **4.20173600** | **2.36626400** | **1.77934800** |
| **C** | **3.16666500** | **1.61243900** | **2.30425100** |
| **C** | **3.16666500** | **1.61243900** | **-2.30425100** |
| **C** | **4.20173600** | **2.36626400** | **-1.77934800** |
| **C** | **5.53852100** | **2.03569200** | **-2.05971300** |
| **C** | **5.81389400** | **0.96493100** | **-2.88861300** |
| **H** | **-2.25654000** | **0.09158300** | **0.00000000** |
| **H** | **-6.94510400** | **1.67853700** | **0.00000000** |
| **H** | **-4.41033400** | **-0.55014600** | **5.66440700** |
| **H** | **-1.26675000** | **0.16993900** | **1.90570200** |
| **H** | **-1.26675000** | **0.16993900** | **-1.90570200** |
| **H** | **-4.41033400** | **-0.55014600** | **-5.66440700** |
| **H** | **1.95208900** | **-2.45562600** | **6.38092800** |
| **H** | **0.70972500** | **0.59593400** | **2.68429000** |
| **H** | **0.70972500** | **0.59593400** | **-2.68429000** |
| **H** | **1.95208900** | **-2.45562600** | **-6.38092800** |
| **H** | **-7.06841600** | **1.53838200** | **2.38116500** |

| **H** | **-6.14952400** | **0.65173800** | **4.49880100** |
| --- | --- | --- | --- |
| **H** | **-2.71183900** | **-1.81672400** | **6.87567500** |
| **H** | **-0.36639600** | **-2.55448300** | **7.11609000** |
| **H** | **4.31450300** | **-2.25788600** | **5.80487700** |
| **H** | **6.11520500** | **-1.07329300** | **4.60548600** |
| **H** | **6.11520500** | **-1.07329300** | **-4.60548600** |
| **H** | **4.31450300** | **-2.25788600** | **-5.80487700** |
| **H** | **-0.36639600** | **-2.55448300** | **-7.11609000** |
| **H** | **-2.71183900** | **-1.81672400** | **-6.87567500** |
| **H** | **-6.14952400** | **0.65173800** | **-4.49880100** |
| **H** | **-7.06841600** | **1.53838200** | **-2.38116500** |
| **H** | **6.84259100** | **0.70677100** | **3.12930600** |
| **H** | **6.34769900** | **2.62586800** | **1.63862700** |
| **H** | **3.97544700** | **3.22597600** | **1.15665800** |
| **H** | **2.14540300** | **1.90204800** | **2.08036000** |
| **H** | **2.14540300** | **1.90204800** | **-2.08036000** |
| **H** | **3.97544700** | **3.22597600** | **-1.15665800** |
| **H** | **6.34769900** | **2.62586800** | **-1.63862700** |
| **H** | **6.84259100** | **0.70677100** | **-3.12930600** |

Transition State of Hydroxyl Functionalized Expanded [13]-helicene:

X Y Z

**-----------------------------------------------------------------**

| **C** | **-2.46080300** | **4.88765000** | **1.11551900** |
| --- | --- | --- | --- |
| **C** | **-2.16479000** | **3.55813900** | **0.66425600** |
| **C** | **-0.81407900** | **3.21468600** | **0.46804800** |
| **C** | **0.24032200** | **4.13169200** | **0.63461400** |
| **C** | **-0.08611900** | **5.45557500** | **1.08231700** |
| **C** | **-1.41478300** | **5.77768400** | **1.35716800** |
| **C** | **-3.29105400** | **2.68038400** | **0.31685800** |
| **C** | **1.63344700** | **3.85287500** | **0.25822000** |
| **C** | **-4.60144100** | **3.26212800** | **0.23671800** |
| **C** | **-5.64079100** | **2.53085000** | **-0.34237700** |
| **C** | **-5.47386400** | **1.21178200** | **-0.76331800** |
| **C** | **-4.24008800** | **0.54595400** | **-0.46967000** |
| **C** | **-3.16864600** | **1.31297300** | **0.01679400** |
| **C** | **2.13784700** | **2.57365300** | **-0.03163300** |
| **C** | **3.42892900** | **2.36816300** | **-0.54667400** |
| **C** | **4.21670500** | **3.51798000** | **-0.87966000** |
| **C** | **3.77680200** | **4.77598200** | **-0.46823500** |
| **C** | **2.53387600** | **4.96490800** | **0.13893500** |
| **C** | **-4.17147500** | **-0.89836800** | **-0.70701300** |
| **C** | **4.01999500** | **1.04618800** | **-0.77519100** |
| **C** | **-5.19173500** | **-1.51341200** | **-1.50809100** |
| **C** | **-5.09005000** | **-2.87110200** | **-1.81225500** |
| **C** | **-4.10044600** | **-3.68574200** | **-1.25060900** |
| **C** | **-3.18194800** | **-3.11516800** | **-0.31084800** |
| **C** | **-3.20767700** | **-1.72365600** | **-0.11883300** |
| **C** | **3.54312200** | **-0.11375300** | **-0.15212200** |
| **C** | **4.14993000** | **-1.37501500** | **-0.32010800** |
| **C** | **5.21092900** | **-1.47065400** | **-1.28570900** |
| **C** | **5.70823500** | **-0.30829700** | **-1.88306700** |
| **C** | **5.19021300** | **0.95360100** | **-1.59640000** |
| **C** | **-2.30530800** | **-4.01063600** | **0.43788400** |
| **C** | **-2.28536600** | **-5.39896100** | **0.10957600** |
| **C** | **4.44751900** | **-3.81424000** | **0.12111100** |
| **C** | **3.80905800** | **-2.57462400** | **0.44964600** |
| **C** | **-3.82071100** | **5.35090200** | **1.19739200** |
| **C** | **-4.83911100** | **4.59291100** | **0.72748200** |
| **C** | **-6.50587400** | **0.53139200** | **-1.50266100** |
| **C** | **-6.33957300** | **-0.74740000** | **-1.92011400** |
| **C** | **-4.02679300** | **-5.08638400** | **-1.56293700** |
| **C** | **-3.14017800** | **-5.89913400** | **-0.93302700** |
| **C** | **5.40615100** | **-3.86556000** | **-0.94881200** |

| **C** | **5.79163000** | **-2.74348600** | **-1.60257600** |
| --- | --- | --- | --- |
| **C** | **5.85503100** | **2.14857000** | **-2.04640200** |
| **C** | **5.42850300** | **3.37048300** | **-1.64374100** |
| **C** | **2.15529700** | **6.26493700** | **0.62366400** |
| **C** | **0.91805800** | **6.48534800** | **1.12673400** |
| **C** | **-1.46502300** | **-6.28037000** | **0.84785200** |
| **C** | **-0.71295200** | **-5.82904200** | **1.91543100** |
| **C** | **-0.75917600** | **-4.46887800** | **2.26577700** |
| **C** | **-1.52536500** | **-3.57934000** | **1.53335100** |
| **C** | **2.93682400** | **-2.60177900** | **1.57580700** |
| **C** | **2.74148200** | **-3.76159100** | **2.31698900** |
| **C** | **3.35871000** | **-4.96348300** | **1.95634000** |
| **C** | **4.19594200** | **-4.98970700** | **0.86006600** |
| **H** | **-0.58360500** | **2.23330100** | **0.06670900** |
| **H** | **-1.65235900** | **6.78983000** | **1.67832500** |
| **H** | **-6.60579900** | **3.01313100** | **-0.48493800** |
| **H** | **-2.20336200** | **0.83320800** | **0.14330400** |
| **H** | **1.50282400** | **1.70879100** | **0.13104300** |
| **H** | **4.41468300** | **5.64094800** | **-0.63910700** |
| **H** | **-5.83344000** | **-3.32216600** | **-2.46655300** |
| **H** | **-2.48540700** | **-1.27126000** | **0.55102400** |
| **H** | **2.71803900** | **-0.02432500** | **0.53558800** |
| **H** | **6.56048600** | **-0.38992700** | **-2.55482400** |
| **H** | **-3.99901600** | **6.35432900** | **1.57578800** |
| **H** | **-5.85859700** | **4.97028900** | **0.71528600** |
| **H** | **-7.40701100** | **1.08613300** | **-1.75216200** |
| **H** | **-7.10064100** | **-1.24218000** | **-2.51835200** |
| **H** | **-4.70537300** | **-5.48186400** | **-2.31480400** |
| **H** | **-3.09007300** | **-6.95770500** | **-1.17618400** |
| **H** | **5.84928200** | **-4.82851200** | **-1.18940300** |
| **H** | **6.55557500** | **-2.78299400** | **-2.37526300** |
| **H** | **6.74549400** | **2.04272600** | **-2.66124800** |
| **H** | **5.97147100** | **4.27058100** | **-1.92149400** |
| **H** | **2.89254200** | **7.06289600** | **0.58162800** |
| **H** | **0.63294000** | **7.46476900** | **1.50255400** |
| **H** | **-1.45473500** | **-7.33307100** | **0.57507500** |
| **H** | **-0.10176800** | **-6.52105100** | **2.48792800** |
| **H** | **-0.20189800** | **-4.11465700** | **3.12764200** |
| **H** | **-1.55190700** | **-2.53795100** | **1.83665000** |
| **H** | **2.09608800** | **-3.72260900** | **3.19172000** |
| **H** | **3.18148000** | **-5.85994200** | **2.54354500** |
| **H** | **4.69637600** | **-5.90823600** | **0.56646200** |
| **O** | **2.29720300** | **-1.45012400** | **1.95643000** |
| **H** | **1.78404600** | **-1.63713800** | **2.75784300** |

Transition State of Methyl Functionalized Expanded [13]-helicene:

X Y Z

**-----------------------------------------------------------------**

| **C** | **-2.24157600** | **5.02001800** | **1.02688800** |
| --- | --- | --- | --- |
| **C** | **-2.00285700** | **3.67328800** | **0.59349200** |
| **C** | **-0.66730900** | **3.25911500** | **0.43420700** |
| **C** | **0.42955800** | **4.11853900** | **0.63155400** |
| **C** | **0.16055800** | **5.45637700** | **1.07656000** |
| **C** | **-1.15729800** | **5.85195100** | **1.30318100** |
| **C** | **-3.16434400** | **2.84892000** | **0.23230200** |
| **C** | **1.81685300** | **3.76798100** | **0.29394900** |
| **C** | **-4.44118300** | **3.49466000** | **0.11288000** |
| **C** | **-5.50708100** | **2.80153700** | **-0.46461300** |
| **C** | **-5.40331900** | **1.46293900** | **-0.84247400** |
| **C** | **-4.20806600** | **0.74464200** | **-0.51489200** |
| **C** | **-3.10594400** | **1.47090900** | **-0.03429900** |
| **C** | **2.25806800** | **2.46866500** | **-0.01193800** |

| **C** | **3.55050900** | **2.20013700** | **-0.49419600** |
| --- | --- | --- | --- |
| **C** | **4.41439200** | **3.31026200** | **-0.76975500** |
| **C** | **4.03316400** | **4.58108800** | **-0.34062400** |
| **C** | **2.78236700** | **4.82886900** | **0.22747500** |
| **C** | **-4.21005600** | **-0.70823900** | **-0.70524200** |
| **C** | **4.07297300** | **0.85373100** | **-0.74724700** |
| **C** | **-5.26370700** | **-1.30059600** | **-1.47951400** |
| **C** | **-5.23809100** | **-2.67386700** | **-1.72376200** |
| **C** | **-4.28818500** | **-3.51601400** | **-1.13485000** |
| **C** | **-3.32469700** | **-2.95516700** | **-0.23490800** |
| **C** | **-3.28180200** | **-1.55806300** | **-0.09545400** |
| **C** | **3.52472700** | **-0.30124400** | **-0.17310400** |
| **C** | **4.06333700** | **-1.58916500** | **-0.36455500** |
| **C** | **5.09448200** | **-1.71400800** | **-1.35979800** |
| **C** | **5.67988900** | **-0.56260200** | **-1.89474800** |
| **C** | **5.25312800** | **0.71840400** | **-1.54768800** |
| **C** | **-2.47248900** | **-3.86101800** | **0.52969100** |
| **C** | **-2.53953800** | **-5.26240000** | **0.27043400** |
| **C** | **4.09507300** | **-4.05906800** | **-0.13078700** |
| **C** | **3.65706400** | **-2.79109700** | **0.37783500** |
| **C** | **-3.57647000** | **5.55616700** | **1.06091100** |
| **C** | **-4.61999100** | **4.84570200** | **0.57212000** |
| **C** | **-6.46568900** | **0.81076400** | **-1.56395500** |
| **C** | **-6.36820100** | **-0.49164100** | **-1.92625200** |
| **C** | **-4.29978600** | **-4.93183800** | **-1.37967800** |
| **C** | **-3.44894100** | **-5.76277600** | **-0.72472100** |
| **C** | **4.99388700** | **-4.13009300** | **-1.25125700** |
| **C** | **5.52216000** | **-3.01027900** | **-1.79973100** |
| **C** | **6.00292200** | **1.88377300** | **-1.93595800** |
| **C** | **5.63926500** | **3.11483000** | **-1.50119000** |
| **C** | **2.46046300** | **6.14197700** | **0.71752200** |
| **C** | **1.21778400** | **6.42774300** | **1.17106100** |
| **C** | **-1.74432500** | **-6.15188400** | **1.02624700** |
| **C** | **-0.92553000** | **-5.69110400** | **2.03936600** |
| **C** | **-0.87671000** | **-4.31414800** | **2.31662200** |
| **C** | **-1.62474100** | **-3.42122600** | **1.56994600** |
| **C** | **2.94358800** | **-2.79185200** | **1.61961100** |
| **C** | **2.64787300** | **-4.00898900** | **2.23114300** |
| **C** | **2.99946600** | **-5.24355000** | **1.67444400** |
| **C** | **3.72602600** | **-5.26273400** | **0.50339700** |
| **H** | **-0.47984900** | **2.26545400** | **0.04074000** |
| **H** | **-1.34954000** | **6.87586600** | **1.61717000** |
| **H** | **-6.44478100** | **3.32709100** | **-0.63387100** |
| **H** | **-2.16845100** | **0.94741200** | **0.12292600** |
| **H** | **1.56674600** | **1.63997800** | **0.10451600** |
| **H** | **4.72402400** | **5.41167600** | **-0.47092700** |
| **H** | **-6.01126500** | **-3.11201300** | **-2.35181700** |
| **H** | **-2.53308300** | **-1.11781100** | **0.55269000** |
| **H** | **2.66673300** | **-0.17798700** | **0.46756100** |
| **H** | **6.51738400** | **-0.67307500** | **-2.58073200** |
| **H** | **-3.71189800** | **6.57295500** | **1.42088000** |
| **H** | **-5.61732400** | **5.27593100** | **0.52596600** |
| **H** | **-7.33462100** | **1.40258100** | **-1.84097000** |
| **H** | **-7.15389900** | **-0.96906100** | **-2.50652200** |
| **H** | **-5.01481700** | **-5.32352600** | **-2.09905500** |
| **H** | **-3.46533200** | **-6.83326600** | **-0.91474500** |
| **H** | **5.29123600** | **-5.11472800** | **-1.60337600** |
| **H** | **6.26068500** | **-3.06888900** | **-2.59537500** |
| **H** | **6.90133300** | **1.74405400** | **-2.53201000** |
| **H** | **6.24267200** | **3.98928100** | **-1.73190100** |
| **H** | **3.24317900** | **6.89647500** | **0.71570000** |
| **H** | **0.97293700** | **7.41811800** | **1.54648800** |
| **H** | **-1.80395400** | **-7.21585800** | **0.80890200** |

| **H** | **-0.33211700** | **-6.38794700** | **2.62468700** |
| --- | --- | --- | --- |
| **H** | **-0.25457100** | **-3.94533200** | **3.12576000** |
| **H** | **-1.57605500** | **-2.36642800** | **1.81885600** |
| **H** | **2.13913600** | **-3.99153400** | **3.19100600** |
| **H** | **2.73245200** | **-6.16756400** | **2.17958700** |
| **H** | **4.05879800** | **-6.20294600** | **0.07069100** |
| **C** | **2.53810200** | **-1.55047100** | **2.38763300** |
| **H** | **3.33326200** | **-0.79899500** | **2.41553800** |
| **H** | **1.64436300** | **-1.06834600** | **1.97075800** |
| **H** | **2.29808500** | **-1.81871300** | **3.42119000** |

Transition State of Methoxy Functionalized Expanded [13]-helicene:

X Y Z

**-----------------------------------------------------------------**

| **C** | **-1.83091000** | **5.18562900** | **1.08944400** |
| --- | --- | --- | --- |
| **C** | **-1.70519700** | **3.83171300** | **0.63056600** |
| **C** | **-0.40870500** | **3.32522800** | **0.42294400** |
| **C** | **0.75231300** | **4.10378000** | **0.58696800** |
| **C** | **0.59588700** | **5.45479400** | **1.04456600** |
| **C** | **-0.68114600** | **5.93762400** | **1.32903100** |
| **C** | **-2.93370100** | **3.10229700** | **0.28552900** |
| **C** | **2.09801100** | **3.65612000** | **0.20190700** |
| **C** | **-4.15983600** | **3.84570200** | **0.20943300** |
| **C** | **-5.28225100** | **3.25589100** | **-0.37562900** |
| **C** | **-5.28274400** | **1.92925500** | **-0.80540900** |
| **C** | **-4.14631500** | **1.10860000** | **-0.50956200** |
| **C** | **-2.98650700** | **1.73061400** | **-0.01787800** |
| **C** | **2.43419900** | **2.32661600** | **-0.10230000** |
| **C** | **3.68874800** | **1.96191000** | **-0.61831700** |
| **C** | **4.62040700** | **3.00251500** | **-0.93711600** |
| **C** | **4.34355400** | **4.30336400** | **-0.51609900** |
| **C** | **3.13307400** | **4.64507800** | **0.09078000** |
| **C** | **-4.26477300** | **-0.33182000** | **-0.75403100** |
| **C** | **4.10121200** | **0.57692700** | **-0.86059300** |
| **C** | **-5.34633300** | **-0.80229200** | **-1.57277000** |
| **C** | **-5.41743400** | **-2.15862700** | **-1.89046500** |
| **C** | **-4.55166400** | **-3.10023900** | **-1.32324400** |
| **C** | **-3.58427900** | **-2.66332300** | **-0.36102500** |
| **C** | **-3.42793500** | **-1.28134100** | **-0.15794600** |
| **C** | **3.47005900** | **-0.51429700** | **-0.25337200** |
| **C** | **3.89710000** | **-1.84486600** | **-0.43679200** |
| **C** | **4.92582400** | **-2.07127700** | **-1.41619500** |
| **C** | **5.58655800** | **-0.97954300** | **-1.98976700** |
| **C** | **5.25158900** | **0.33767000** | **-1.67962800** |
| **C** | **-2.85128300** | **-3.67421300** | **0.39588500** |
| **C** | **-3.00500500** | **-5.04973400** | **0.04863800** |
| **C** | **3.79353700** | **-4.30932000** | **-0.09070000** |
| **C** | **3.38767800** | **-2.99802000** | **0.31093400** |
| **C** | **-3.12197500** | **5.81457300** | **1.17996000** |
| **C** | **-4.22806500** | **5.19284400** | **0.70840200** |
| **C** | **-6.38757700** | **1.39352900** | **-1.55820900** |
| **C** | **-6.38165700** | **0.10832800** | **-1.98842000** |
| **C** | **-4.65502400** | **-4.49501200** | **-1.65283200** |
| **C** | **-3.89409500** | **-5.42299100** | **-1.01806500** |
| **C** | **4.72184000** | **-4.47537600** | **-1.17585100** |
| **C** | **5.29409000** | **-3.40781200** | **-1.78297200** |
| **C** | **6.07230400** | **1.43949300** | **-2.10991900** |
| **C** | **5.80746300** | **2.70326200** | **-1.69634100** |
| **C** | **2.92106100** | **5.97911700** | **0.58451700** |
| **C** | **1.72157900** | **6.35015600** | **1.09094100** |
| **C** | **-2.32546100** | **-6.03932200** | **0.79264500** |
| **C** | **-1.54834900** | **-5.70270700** | **1.88436300** |

| **C** | **-1.42634800** | **-4.35255600** | **2.25448500** |
| --- | --- | --- | --- |
| **C** | **-2.05144000** | **-3.36190200** | **1.51714300** |
| **C** | **2.56894400** | **-2.91816300** | **1.48247500** |
| **C** | **2.17283000** | **-4.06327400** | **2.16562200** |
| **C** | **2.54663700** | **-5.33573800** | **1.71275300** |
| **C** | **3.34667100** | **-5.45791600** | **0.59821000** |
| **H** | **-0.30490900** | **2.32548400** | **0.01417500** |
| **H** | **-0.78966200** | **6.96955400** | **1.65673000** |
| **H** | **-6.17748900** | **3.85794500** | **-0.51784200** |
| **H** | **-2.09079100** | **1.13055500** | **0.10831300** |
| **H** | **1.69234300** | **1.54920900** | **0.04859200** |
| **H** | **5.08739700** | **5.08102200** | **-0.67808300** |
| **H** | **-6.20299500** | **-2.50310500** | **-2.56010000** |
| **H** | **-2.66233800** | **-0.93281900** | **0.52608800** |
| **H** | **2.65894200** | **-0.32386700** | **0.42926800** |
| **H** | **6.41527300** | **-1.16901500** | **-2.66922700** |
| **H** | **-3.17237300** | **6.83012200** | **1.56460200** |
| **H** | **-5.19231700** | **5.69491300** | **0.70067500** |
| **H** | **-7.20780300** | **2.06162600** | **-1.80899200** |
| **H** | **-7.19334600** | **-0.27949500** | **-2.59887700** |
| **H** | **-5.36423600** | **-4.79019200** | **-2.42228900** |
| **H** | **-3.97652300** | **-6.47633300** | **-1.27474400** |
| **H** | **4.99238300** | **-5.48834300** | **-1.46284300** |
| **H** | **6.03984700** | **-3.54221700** | **-2.56272200** |
| **H** | **6.94502500** | **1.22437600** | **-2.72174100** |
| **H** | **6.46630300** | **3.52637000** | **-1.96204700** |
| **H** | **3.75252600** | **6.67860600** | **0.54654100** |
| **H** | **1.56245700** | **7.35539800** | **1.47303000** |
| **H** | **-2.44644900** | **-7.08110100** | **0.50495200** |
| **H** | **-1.04865300** | **-6.47560000** | **2.46165100** |
| **H** | **2.21255700** | **-6.21601500** | **2.25479600** |
| **H** | **3.66763200** | **-6.43526600** | **0.24838100** |
| **O** | **2.22255300** | **-1.67010900** | **1.92152100** |
| **C** | **1.56624000** | **-1.52719900** | **3.17065300** |
| **H** | **1.48011300** | **-0.45181900** | **3.33763900** |
| **H** | **0.56307200** | **-1.97178700** | **3.15371200** |
| **H** | **2.14918700** | **-1.97638500** | **3.98456700** |
| **H** | **1.56642200** | **-3.97933600** | **3.05765900** |
| **H** | **-1.95247200** | **-2.32974700** | **1.83710100** |
| **H** | **-0.84995400** | **-4.08434200** | **3.13479700** |

Transition State of Amino Functionalized Expanded [13]-helicene:

X Y Z

**-----------------------------------------------------------------**

| **C** | **-1.63183300** | **5.24182000** | **1.03812500** |
| --- | --- | --- | --- |
| **C** | **-1.55050200** | **3.87733100** | **0.60168300** |
| **C** | **-0.27163200** | **3.31172000** | **0.44232000** |
| **C** | **0.91689500** | **4.03924300** | **0.63999300** |
| **C** | **0.80439000** | **5.39857800** | **1.08651600** |
| **C** | **-0.45884300** | **5.94269600** | **1.31567000** |
| **C** | **-2.79932200** | **3.19458200** | **0.23550800** |
| **C** | **2.25427500** | **3.53199300** | **0.30003800** |
| **C** | **-3.99088600** | **3.98639300** | **0.11368300** |
| **C** | **-5.12764900** | **3.42594400** | **-0.47216000** |
| **C** | **-5.18008800** | **2.08604900** | **-0.85540700** |
| **C** | **-4.08045200** | **1.22987000** | **-0.52338000** |
| **C** | **-2.90244400** | **1.81959400** | **-0.03505400** |
| **C** | **2.54233200** | **2.19050900** | **-0.00503500** |
| **C** | **3.79481900** | **1.77574900** | **-0.48961100** |
| **C** | **4.78116800** | **2.77820700** | **-0.76645600** |
| **C** | **4.54872100** | **4.08501700** | **-0.33822700** |
| **C** | **3.33531600** | **4.47505800** | **0.23101800** |

| **C** | **-4.25583300** | **-0.21245600** | **-0.71668000** |
| --- | --- | --- | --- |
| **C** | **4.15696400** | **0.37771700** | **-0.74049200** |
| **C** | **-5.36908100** | **-0.67215500** | **-1.49792300** |
| **C** | **-5.51174400** | **-2.03835400** | **-1.74033000** |
| **C** | **-4.67870500** | **-2.99026600** | **-1.14219700** |
| **C** | **-3.65725700** | **-2.55107100** | **-0.23850600** |
| **C** | **-3.44203200** | **-1.16934300** | **-0.10144300** |
| **C** | **3.47486100** | **-0.70405600** | **-0.16599700** |
| **C** | **3.86504900** | **-2.04545400** | **-0.36060900** |
| **C** | **4.87462000** | **-2.29408800** | **-1.35347100** |
| **C** | **5.59143100** | **-1.21885700** | **-1.88558400** |
| **C** | **5.31564700** | **0.10412400** | **-1.53772900** |
| **C** | **-2.93166200** | **-3.55425900** | **0.53580200** |
| **C** | **-3.18254800** | **-4.93720100** | **0.28926700** |
| **C** | **3.57435500** | **-4.50439900** | **-0.13299500** |
| **C** | **3.31412000** | **-3.18920200** | **0.36968300** |
| **C** | **-2.89528600** | **5.92952600** | **1.07254000** |
| **C** | **-4.01285300** | **5.34716400** | **0.57831100** |
| **C** | **-6.30782100** | **1.56803800** | **-1.58623800** |
| **C** | **-6.36468500** | **0.26421600** | **-1.95142000** |
| **C** | **-4.87090400** | **-4.39434000** | **-1.37915100** |
| **C** | **-4.14185800** | **-5.32346700** | **-0.70970200** |
| **C** | **4.46726000** | **-4.68208700** | **-1.24730400** |
| **C** | **5.13754600** | **-3.63599600** | **-1.78891500** |
| **C** | **6.19631500** | **1.17412900** | **-1.92511200** |
| **C** | **5.97633300** | **2.44070000** | **-1.49498700** |
| **C** | **3.16706700** | **5.81630100** | **0.72192700** |
| **C** | **1.96644400** | **6.24212500** | **1.17894400** |
| **C** | **-2.51883700** | **-5.91691200** | **1.06013300** |
| **C** | **-1.65271500** | **-5.55876000** | **2.07530500** |
| **C** | **-1.41892200** | **-4.19775900** | **2.33565500** |
| **C** | **-2.03474100** | **-3.22050200** | **1.57414300** |
| **C** | **2.61159300** | **-3.09460100** | **1.61317900** |
| **C** | **2.12100700** | **-4.25220500** | **2.22242400** |
| **C** | **2.29597300** | **-5.51527800** | **1.65377400** |
| **C** | **3.03108900** | **-5.64486700** | **0.49123900** |
| **H** | **-0.19983400** | **2.30313600** | **0.04881600** |
| **H** | **-0.53181400** | **6.98149200** | **1.63122000** |
| **H** | **-5.99595900** | **4.05892200** | **-0.64401800** |
| **H** | **-2.03418400** | **1.18852600** | **0.12523500** |
| **H** | **1.76182500** | **1.44602900** | **0.11590500** |
| **H** | **5.33052300** | **4.83056800** | **-0.46917300** |
| **H** | **-6.32977900** | **-2.37892000** | **-2.37205600** |
| **H** | **-2.64867300** | **-0.82374100** | **0.55096500** |
| **H** | **2.61745200** | **-0.49707900** | **0.46001800** |
| **H** | **6.41075200** | **-1.42449900** | **-2.57174800** |
| **H** | **-2.91222100** | **6.95390500** | **1.43608500** |
| **H** | **-4.95326700** | **5.89060600** | **0.53113300** |
| **H** | **-7.09737300** | **2.26064500** | **-1.86721900** |
| **H** | **-7.19787100** | **-0.11472900** | **-2.53809700** |
| **H** | **-5.62495700** | **-4.69528400** | **-2.10238300** |
| **H** | **-4.29656800** | **-6.38432700** | **-0.89138500** |
| **H** | **4.63992500** | **-5.69471000** | **-1.60309600** |
| **H** | **5.86759100** | **-3.78834500** | **-2.58009800** |
| **H** | **7.07381000** | **0.93027200** | **-2.51891900** |
| **H** | **6.67727800** | **3.23876100** | **-1.72689600** |
| **H** | **4.03095700** | **6.47634100** | **0.71811300** |
| **H** | **1.83762900** | **7.25388100** | **1.55519200** |
| **H** | **-2.71898100** | **-6.96560300** | **0.85266400** |
| **H** | **-1.16300600** | **-6.32231300** | **2.67339500** |
| **H** | **-0.75531300** | **-3.90617200** | **3.14329800** |
| **H** | **-1.83696900** | **-2.17971800** | **1.80652500** |
| **H** | **1.60988100** | **-4.15802200** | **3.17752800** |

| **H** | **1.88784500** | **-6.39105200** | **2.15061900** |
| --- | --- | --- | --- |
| **H** | **3.22898900** | **-6.62362400** | **0.06309100** |
| **N** | **2.39524900** | **-1.86734600** | **2.26366800** |
| **H** | **2.13445400** | **-1.99154600** | **3.23642400** |
| **H** | **3.17439300** | **-1.22075000** | **2.19461700** |

Transition State of Carboxyl Functionalized Expanded [13]-helicene:

X Y Z

**-----------------------------------------------------------------**

| **C** | **-2.26645700** | **5.00183700** | **1.13904100** |
| --- | --- | --- | --- |
| **C** | **-2.02569000** | **3.66708700** | **0.66841200** |
| **C** | **-0.69248200** | **3.28314700** | **0.43550100** |
| **C** | **0.39189700** | **4.16869700** | **0.57585300** |
| **C** | **0.12117700** | **5.50172400** | **1.03027900** |
| **C** | **-1.18797500** | **5.86126300** | **1.34924200** |
| **C** | **-3.18634100** | **2.82846400** | **0.33593600** |
| **C** | **1.76379300** | **3.84407100** | **0.16637300** |
| **C** | **-4.47870200** | **3.45296000** | **0.30339500** |
| **C** | **-5.55413000** | **2.76990600** | **-0.26733600** |
| **C** | **-5.43761300** | **1.46012600** | **-0.73094400** |
| **C** | **-4.21904300** | **0.74650400** | **-0.48677400** |
| **C** | **-3.11282100** | **1.46512800** | **-0.00209700** |
| **C** | **2.21095400** | **2.54523700** | **-0.12459100** |
| **C** | **3.48230600** | **2.29276600** | **-0.66305400** |
| **C** | **4.30671600** | **3.40589300** | **-1.02423900** |
| **C** | **3.92251200** | **4.68374200** | **-0.61695500** |
| **C** | **2.70031700** | **4.92191800** | **0.01734800** |
| **C** | **-4.20445000** | **-0.69230300** | **-0.77121400** |
| **C** | **4.01094700** | **0.94771700** | **-0.89324900** |
| **C** | **-5.26413600** | **-1.24469100** | **-1.56783400** |
| **C** | **-5.22147800** | **-2.59463800** | **-1.91579300** |
| **C** | **-4.25820200** | **-3.46458700** | **-1.39391500** |
| **C** | **-3.30092800** | **-2.95815700** | **-0.45641600** |
| **C** | **-3.25939700** | **-1.57111000** | **-0.23053900** |
| **C** | **3.49285900** | **-0.17711400** | **-0.24728800** |
| **C** | **3.99878500** | **-1.47658700** | **-0.42828700** |
| **C** | **5.06086600** | **-1.62815600** | **-1.38687500** |
| **C** | **5.60600100** | **-0.49854500** | **-2.01028700** |
| **C** | **5.15330500** | **0.79079500** | **-1.74122200** |
| **C** | **-2.46337900** | **-3.91278100** | **0.26376200** |
| **C** | **-2.51719700** | **-5.29355600** | **-0.09183700** |
| **C** | **4.29282100** | **-3.87440200** | **0.14394900** |
| **C** | **3.58894900** | **-2.64549000** | **0.36431600** |
| **C** | **-3.60864600** | **5.50295000** | **1.27241700** |
| **C** | **-4.66241700** | **4.78025300** | **0.82597700** |
| **C** | **-6.50811400** | **0.84003600** | **-1.46809800** |
| **C** | **-6.39332500** | **-0.42832100** | **-1.93071400** |
| **C** | **-4.25264300** | **-4.85879400** | **-1.74199100** |
| **C** | **-3.40088400** | **-5.72770800** | **-1.13979500** |
| **C** | **5.26492700** | **-3.99052000** | **-0.90797900** |
| **C** | **5.63218100** | **-2.91667000** | **-1.64532200** |
| **C** | **5.85864400** | **1.95312500** | **-2.21694700** |
| **C** | **5.49320200** | **3.19588700** | **-1.81506100** |
| **C** | **2.37532100** | **6.23662400** | **0.50212500** |
| **C** | **1.15948800** | **6.49835400** | **1.03831700** |
| **C** | **-1.74443900** | **-6.23190300** | **0.62745100** |
| **C** | **-0.97208600** | **-5.84253000** | **1.70506500** |
| **C** | **-0.94525800** | **-4.48874900** | **2.08159200** |
| **C** | **-1.65983000** | **-3.54493000** | **1.36467600** |
| **C** | **2.62992400** | **-2.65219600** | **1.43146200** |
| **C** | **2.51891100** | **-3.75572900** | **2.27847500** |
| **C** | **3.23744700** | **-4.93506500** | **2.05766300** |

| **C** | **4.08484100** | **-4.99743100** | **0.97428300** |
| --- | --- | --- | --- |
| **H** | **-0.50224900** | **2.29606000** | **0.02758600** |
| **H** | **-1.38641300** | **6.87845500** | **1.68091900** |
| **H** | **-6.50667200** | **3.28524600** | **-0.37355400** |
| **H** | **-2.16065300** | **0.95133800** | **0.08804200** |
| **H** | **1.54676700** | **1.70562200** | **0.05417600** |
| **H** | **4.58636000** | **5.52390400** | **-0.81103500** |
| **H** | **-5.99422000** | **-2.99575400** | **-2.56865600** |
| **H** | **-2.49295700** | **-1.16862800** | **0.42198000** |
| **H** | **2.69839800** | **-0.00741400** | **0.45656800** |
| **H** | **6.45404900** | **-0.63308100** | **-2.67857000** |
| **H** | **-3.74568000** | **6.50568300** | **1.66938500** |
| **H** | **-5.67065900** | **5.18618800** | **0.85196300** |
| **H** | **-7.39485700** | **1.43280900** | **-1.67872200** |
| **H** | **-7.18274300** | **-0.87831000** | **-2.52764800** |
| **H** | **-4.95666900** | **-5.20316900** | **-2.49568400** |
| **H** | **-3.40416900** | **-6.78165500** | **-1.40729100** |
| **H** | **5.72469400** | **-4.96183800** | **-1.07078300** |
| **H** | **6.39480000** | **-3.00042700** | **-2.41538700** |
| **H** | **6.72926400** | **1.80551100** | **-2.85109300** |
| **H** | **6.06837200** | **4.06829800** | **-2.11564000** |
| **H** | **3.13522600** | **7.01127400** | **0.43523700** |
| **H** | **0.91735100** | **7.48853700** | **1.41623200** |
| **H** | **-1.79053500** | **-7.27810900** | **0.33381900** |
| **H** | **-0.40118200** | **-6.57780500** | **2.26542800** |
| **H** | **-0.37284500** | **-4.17900900** | **2.95017000** |
| **H** | **1.83096300** | **-3.69960600** | **3.11229700** |
| **H** | **3.11173300** | **-5.78506800** | **2.72151300** |
| **H** | **4.63883900** | **-5.90747700** | **0.75778900** |
| **C** | **1.57843800** | **-1.61580000** | **1.67647800** |
| **O** | **0.85921200** | **-1.10083900** | **0.84163200** |
| **O** | **1.42128100** | **-1.34986100** | **3.00047800** |
| **H** | **0.66618100** | **-0.73382000** | **3.05740000** |
| **H** | **-1.62123100** | **-2.50865100** | **1.68028800** |

Transition State of Bor Doped Expanded [13]-helicene:

X Y Z

**-----------------------------------------------------------------**

| **C** | **-5.26395200** | **0.97626900** | **1.24361800** |
| --- | --- | --- | --- |
| **C** | **-3.85318900** | **0.62728100** | **1.37710700** |
| **C** | **-3.85318900** | **0.62728100** | **-1.37710700** |
| **C** | **-5.26395200** | **0.97626900** | **-1.24361800** |
| **C** | **-5.88329200** | **1.17692000** | **0.00000000** |
| **C** | **-3.31298800** | **0.30185500** | **2.68464000** |
| **C** | **-3.31298800** | **0.30185500** | **-2.68464000** |
| **C** | **-4.21971600** | **0.20395300** | **3.79158900** |
| **C** | **-3.76384000** | **-0.31929900** | **5.00202200** |
| **C** | **-2.43095900** | **-0.68515600** | **5.19654600** |
| **C** | **-1.47551900** | **-0.40716300** | **4.16435800** |
| **C** | **-1.95272900** | **0.03813800** | **2.92068700** |
| **C** | **-1.95272900** | **0.03813800** | **-2.92068700** |
| **C** | **-1.47551900** | **-0.40716300** | **-4.16435800** |
| **C** | **-2.43095900** | **-0.68515600** | **-5.19654600** |
| **C** | **-3.76384000** | **-0.31929900** | **-5.00202200** |
| **C** | **-4.21971600** | **0.20395300** | **-3.79158900** |
| **C** | **-0.05755100** | **-0.61821300** | **4.46988700** |
| **C** | **-0.05755100** | **-0.61821300** | **-4.46988700** |
| **C** | **0.29010400** | **-1.35895500** | **5.64962500** |
| **C** | **1.63276500** | **-1.63782300** | **5.90594000** |
| **C** | **2.66426700** | **-1.11527100** | **5.11836700** |
| **C** | **2.33481400** | **-0.24106300** | **4.03203700** |
| **C** | **0.97872300** | **-0.06744800** | **3.70831100** |

| **C** | **0.97872300** | **-0.06744800** | **-3.70831100** |
| --- | --- | --- | --- |
| **C** | **2.33481400** | **-0.24106300** | **-4.03203700** |
| **C** | **2.66426700** | **-1.11527100** | **-5.11836700** |
| **C** | **1.63276500** | **-1.63782300** | **-5.90594000** |
| **C** | **0.29010400** | **-1.35895500** | **-5.64962500** |
| **C** | **3.41793800** | **0.45977300** | **3.34791900** |
| **C** | **4.76904700** | **0.15337900** | **3.68983800** |
| **C** | **4.76904700** | **0.15337900** | **-3.68983800** |
| **C** | **3.41793800** | **0.45977300** | **-3.34791900** |
| **C** | **-6.07321200** | **1.01453500** | **2.43677400** |
| **C** | **-5.58966400** | **0.61213200** | **3.63617000** |
| **C** | **-2.02167000** | **-1.36274700** | **6.39925400** |
| **C** | **-0.73420900** | **-1.74309700** | **6.58544500** |
| **C** | **4.04048500** | **-1.40574600** | **5.41232500** |
| **C** | **5.04505100** | **-0.81912400** | **4.71253300** |
| **C** | **5.04505100** | **-0.81912400** | **-4.71253300** |
| **C** | **4.04048500** | **-1.40574600** | **-5.41232500** |
| **C** | **-0.73420900** | **-1.74309700** | **-6.58544500** |
| **C** | **-2.02167000** | **-1.36274700** | **-6.39925400** |
| **C** | **-5.58966400** | **0.61213200** | **-3.63617000** |
| **C** | **-6.07321200** | **1.01453500** | **-2.43677400** |
| **C** | **5.82506000** | **0.84337900** | **3.05483500** |
| **C** | **5.57230900** | **1.84260700** | **2.13439500** |
| **C** | **4.24272400** | **2.17122600** | **1.81992600** |
| **C** | **3.19219700** | **1.48499100** | **2.40361700** |
| **C** | **3.19219700** | **1.48499100** | **-2.40361700** |
| **C** | **4.24272400** | **2.17122600** | **-1.81992600** |
| **C** | **5.57230900** | **1.84260700** | **-2.13439500** |
| **C** | **5.82506000** | **0.84337900** | **-3.05483500** |
| **H** | **-6.94264700** | **1.41740600** | **0.00000000** |
| **H** | **-4.47533000** | **-0.46763300** | **5.81193600** |
| **H** | **-1.25447700** | **0.15851500** | **2.09610200** |
| **H** | **-1.25447700** | **0.15851500** | **-2.09610200** |
| **H** | **-4.47533000** | **-0.46763300** | **-5.81193600** |
| **H** | **1.88876100** | **-2.24393700** | **6.77270500** |
| **H** | **0.71695500** | **0.55666200** | **2.86168400** |
| **H** | **0.71695500** | **0.55666200** | **-2.86168400** |
| **H** | **1.88876100** | **-2.24393700** | **-6.77270500** |
| **H** | **-7.11089500** | **1.32092800** | **2.33115300** |
| **H** | **-6.23372800** | **0.58680100** | **4.51176300** |
| **H** | **-2.78363400** | **-1.59616600** | **7.13884000** |
| **H** | **-0.43835100** | **-2.29385300** | **7.47481800** |
| **H** | **4.25798000** | **-2.10573400** | **6.21537500** |
| **H** | **6.08387100** | **-1.04554900** | **4.94027000** |
| **H** | **6.08387100** | **-1.04554900** | **-4.94027000** |
| **H** | **4.25798000** | **-2.10573400** | **-6.21537500** |
| **H** | **-0.43835100** | **-2.29385300** | **-7.47481800** |
| **H** | **-2.78363400** | **-1.59616600** | **-7.13884000** |
| **H** | **-6.23372800** | **0.58680100** | **-4.51176300** |
| **H** | **-7.11089500** | **1.32092800** | **-2.33115300** |
| **H** | **6.84799700** | **0.58836800** | **3.32194500** |
| **H** | **6.39353400** | **2.37905700** | **1.66726700** |
| **H** | **4.03408400** | **2.97745400** | **1.12411200** |
| **H** | **2.17731700** | **1.77212600** | **2.14954300** |
| **H** | **2.17731700** | **1.77212600** | **-2.14954300** |
| **H** | **4.03408400** | **2.97745400** | **-1.12411200** |
| **H** | **6.39353400** | **2.37905700** | **-1.66726700** |
| **H** | **6.84799700** | **0.58836800** | **-3.32194500** |
| **B** | **-3.40824000** | **0.59321000** | **0.00000000** |

Transition State of Nitrogene Doped Expanded [13]-helicene:

X Y Z

**-----------------------------------------------------------------**

| **C** | **1.16857800** | **5.34230700** | **1.20907100** |
| --- | --- | --- | --- |
| **C** | **0.71891000** | **3.97945100** | **1.16373600** |
| **C** | **0.71891000** | **3.97945100** | **-1.16373600** |
| **C** | **1.16857800** | **5.34230700** | **-1.20907100** |
| **C** | **1.43605400** | **5.97832000** | **0.00000000** |
| **C** | **0.33188800** | **3.34181600** | **2.42725000** |
| **C** | **0.33188800** | **3.34181600** | **-2.42725000** |
| **C** | **0.21966900** | **4.17081000** | **3.59256900** |
| **C** | **-0.41933600** | **3.66250500** | **4.72597100** |
| **C** | **-0.86115700** | **2.34145800** | **4.79194900** |
| **C** | **-0.52432400** | **1.44292100** | **3.72611900** |
| **C** | **0.00505800** | **1.98158600** | **2.54234200** |
| **C** | **0.00505800** | **1.98158600** | **-2.54234200** |
| **C** | **-0.52432400** | **1.44292100** | **-3.72611900** |
| **C** | **-0.86115700** | **2.34145800** | **-4.79194900** |
| **C** | **-0.41933600** | **3.66250500** | **-4.72597100** |
| **C** | **0.21966900** | **4.17081000** | **-3.59256900** |
| **C** | **-0.76784500** | **0.01442900** | **3.94332600** |
| **C** | **-0.76784500** | **0.01442900** | **-3.94332600** |
| **C** | **-1.63821800** | **-0.38011800** | **5.01414200** |
| **C** | **-1.93875400** | **-1.73171500** | **5.18175400** |
| **C** | **-1.29613200** | **-2.73035100** | **4.44092200** |
| **C** | **-0.28253300** | **-2.35438900** | **3.50079800** |
| **C** | **-0.10531300** | **-0.98699400** | **3.22688000** |
| **C** | **-0.10531300** | **-0.98699400** | **-3.22688000** |
| **C** | **-0.28253300** | **-2.35438900** | **-3.50079800** |
| **C** | **-1.29613200** | **-2.73035100** | **-4.44092200** |
| **C** | **-1.93875400** | **-1.73171500** | **-5.18175400** |
| **C** | **-1.63821800** | **-0.38011800** | **-5.01414200** |
| **C** | **0.55849000** | **-3.40179900** | **2.92934600** |
| **C** | **0.24903300** | **-4.76776800** | **3.20209800** |
| **C** | **0.24903300** | **-4.76776800** | **-3.20209800** |
| **C** | **0.55849000** | **-3.40179900** | **-2.92934600** |
| **C** | **1.22161800** | **6.06587400** | **2.45014500** |
| **C** | **0.72044600** | **5.51913600** | **3.58334800** |
| **C** | **-1.67294600** | **1.88596900** | **5.89178400** |
| **C** | **-2.11030100** | **0.60449300** | **5.95336600** |
| **C** | **-1.59254900** | **-4.11981900** | **4.65645300** |
| **C** | **-0.87210200** | **-5.09273100** | **4.04176900** |
| **C** | **-0.87210200** | **-5.09273100** | **-4.04176900** |
| **C** | **-1.59254900** | **-4.11981900** | **-4.65645300** |
| **C** | **-2.11030100** | **0.60449300** | **-5.95336600** |
| **C** | **-1.67294600** | **1.88596900** | **-5.89178400** |
| **C** | **0.72044600** | **5.51913600** | **-3.58334800** |
| **C** | **1.22161800** | **6.06587400** | **-2.45014500** |
| **C** | **1.08232400** | **-5.78825700** | **2.69349200** |
| **C** | **2.22161400** | **-5.48486200** | **1.97291100** |
| **C** | **2.54900600** | **-4.13968300** | **1.73130700** |
| **C** | **1.72635700** | **-3.12394100** | **2.18567400** |
| **C** | **1.72635700** | **-3.12394100** | **-2.18567400** |
| **C** | **2.54900600** | **-4.13968300** | **-1.73130700** |
| **C** | **2.22161400** | **-5.48486200** | **-1.97291100** |
| **C** | **1.08232400** | **-5.78825700** | **-2.69349200** |
| **H** | **1.76997900** | **7.01430200** | **0.00000000** |
| **H** | **-0.59783200** | **4.32355300** | **5.57162300** |
| **H** | **0.13707200** | **1.35381000** | **1.66735300** |
| **H** | **0.13707200** | **1.35381000** | **-1.66735300** |
| **H** | **-0.59783200** | **4.32355300** | **-5.57162300** |
| **H** | **-2.64688300** | **-2.02377100** | **5.95473100** |
| **H** | **0.61524700** | **-0.68576800** | **2.47487200** |
| **H** | **0.61524700** | **-0.68576800** | **-2.47487200** |
| **H** | **-2.64688300** | **-2.02377100** | **-5.95473100** |

| **H** | **1.60076000** | **7.08479600** | **2.43722200** |
| --- | --- | --- | --- |
| **H** | **0.68327000** | **6.08765200** | **4.50918500** |
| **H** | **-1.96136200** | **2.61045100** | **6.64936600** |
| **H** | **-2.76402500** | **0.27669800** | **6.75788300** |
| **H** | **-2.40365900** | **-4.37345700** | **5.33468900** |
| **H** | **-1.10064100** | **-6.14205600** | **4.21215700** |
| **H** | **-1.10064100** | **-6.14205600** | **-4.21215700** |
| **H** | **-2.40365900** | **-4.37345700** | **-5.33468900** |
| **H** | **-2.76402500** | **0.27669800** | **-6.75788300** |
| **H** | **-1.96136200** | **2.61045100** | **-6.64936600** |
| **H** | **0.68327000** | **6.08765200** | **-4.50918500** |
| **H** | **1.60076000** | **7.08479600** | **-2.43722200** |
| **H** | **0.82459900** | **-6.82371800** | **2.90384400** |
| **H** | **2.86684300** | **-6.27877000** | **1.60713000** |
| **H** | **3.45897300** | **-3.89178500** | **1.19374900** |
| **H** | **2.01355300** | **-2.09511800** | **1.99608900** |
| **H** | **2.01355300** | **-2.09511800** | **-1.99608900** |
| **H** | **3.45897300** | **-3.89178500** | **-1.19374900** |
| **H** | **2.86684300** | **-6.27877000** | **-1.60713000** |
| **H** | **0.82459900** | **-6.82371800** | **-2.90384400** |
| **N** | **0.55201400** | **3.32383100** | **0.00000000** |

Transition State of Silicone Doped Expanded [13]-helicene:

X Y Z

**-----------------------------------------------------------------**

| **C** | **-5.04244600** | **0.70754400** | **1.26864600** |
| --- | --- | --- | --- |
| **C** | **-3.64757200** | **0.43498300** | **1.50235400** |
| **C** | **-3.64757200** | **0.43498300** | **-1.50235400** |
| **C** | **-5.04244600** | **0.70754400** | **-1.26864600** |
| **C** | **-5.62466700** | **0.85945300** | **0.00000000** |
| **C** | **-3.19161000** | **0.21850400** | **2.87698100** |
| **C** | **-3.19161000** | **0.21850400** | **-2.87698100** |
| **C** | **-4.16605600** | **0.16888300** | **3.92955900** |
| **C** | **-3.77521700** | **-0.20520700** | **5.21577400** |
| **C** | **-2.44739600** | **-0.48471200** | **5.53781300** |
| **C** | **-1.43587700** | **-0.29351000** | **4.53963500** |
| **C** | **-1.84447100** | **0.02346800** | **3.23458600** |
| **C** | **-1.84447100** | **0.02346800** | **-3.23458600** |
| **C** | **-1.43587700** | **-0.29351000** | **-4.53963500** |
| **C** | **-2.44739600** | **-0.48471200** | **-5.53781300** |
| **C** | **-3.77521700** | **-0.20520700** | **-5.21577400** |
| **C** | **-4.16605600** | **0.16888300** | **-3.92955900** |
| **C** | **-0.03350000** | **-0.45599700** | **4.94075500** |
| **C** | **-0.03350000** | **-0.45599700** | **-4.94075500** |
| **C** | **0.25371100** | **-1.01193800** | **6.23333600** |
| **C** | **1.58062500** | **-1.23908400** | **6.59861400** |
| **C** | **2.65313900** | **-0.86133900** | **5.78437500** |
| **C** | **2.38412700** | **-0.19399900** | **4.54548900** |
| **C** | **1.04466500** | **-0.04973400** | **4.14673200** |
| **C** | **1.04466500** | **-0.04973400** | **-4.14673200** |
| **C** | **2.38412700** | **-0.19399900** | **-4.54548900** |
| **C** | **2.65313900** | **-0.86133900** | **-5.78437500** |
| **C** | **1.58062500** | **-1.23908400** | **-6.59861400** |
| **C** | **0.25371100** | **-1.01193800** | **-6.23333600** |
| **C** | **3.50985700** | **0.32864700** | **3.77521500** |
| **C** | **4.83947200** | **0.07139700** | **4.22571100** |
| **C** | **4.83947200** | **0.07139700** | **-4.22571100** |
| **C** | **3.50985700** | **0.32864700** | **-3.77521500** |
| **C** | **-5.94751000** | **0.76985500** | **2.39419700** |
| **C** | **-5.53912100** | **0.48370400** | **3.65142200** |
| **C** | **-2.09936300** | **-0.98351500** | **6.84223900** |
| **C** | **-0.81622900** | **-1.28275700** | **7.15743400** |

| **C** | **4.00990500** | **-1.10473700** | **6.18955300** |
| --- | --- | --- | --- |
| **C** | **5.05371600** | **-0.67653900** | **5.43489600** |
| **C** | **5.05371600** | **-0.67653900** | **-5.43489600** |
| **C** | **4.00990500** | **-1.10473700** | **-6.18955300** |
| **C** | **-0.81622900** | **-1.28275700** | **-7.15743400** |
| **C** | **-2.09936300** | **-0.98351500** | **-6.84223900** |
| **C** | **-5.53912100** | **0.48370400** | **-3.65142200** |
| **C** | **-5.94751000** | **0.76985500** | **-2.39419700** |
| **C** | **5.93669400** | **0.58426900** | **3.49932500** |
| **C** | **5.74592600** | **1.36071400** | **2.37235200** |
| **C** | **4.43816100** | **1.63913100** | **1.94047000** |
| **C** | **3.34799600** | **1.12559700** | **2.62091800** |
| **C** | **3.34799600** | **1.12559700** | **-2.62091800** |
| **C** | **4.43816100** | **1.63913100** | **-1.94047000** |
| **C** | **5.74592600** | **1.36071400** | **-2.37235200** |
| **C** | **5.93669400** | **0.58426900** | **-3.49932500** |
| **H** | **-1.24304500** | **-0.05203800** | **0.00000000** |
| **H** | **-6.69342700** | **1.06004000** | **0.00000000** |
| **H** | **-4.53600300** | **-0.29846000** | **5.98828700** |
| **H** | **-1.09007000** | **0.09543200** | **2.45706700** |
| **H** | **-1.09007000** | **0.09543200** | **-2.45706700** |
| **H** | **-4.53600300** | **-0.29846000** | **-5.98828700** |
| **H** | **1.78930700** | **-1.69704800** | **7.56343000** |
| **H** | **0.83218900** | **0.42701600** | **3.19725700** |
| **H** | **0.83218900** | **0.42701600** | **-3.19725700** |
| **H** | **1.78930700** | **-1.69704800** | **-7.56343000** |
| **H** | **-6.98846800** | **1.01027200** | **2.19443600** |
| **H** | **-6.24406900** | **0.48657700** | **4.47929400** |
| **H** | **-2.90095200** | **-1.14370800** | **7.55905500** |
| **H** | **-0.56222100** | **-1.69289400** | **8.13166700** |
| **H** | **4.18013000** | **-1.64033800** | **7.12030400** |
| **H** | **6.07763500** | **-0.86695000** | **5.74727500** |
| **H** | **6.07763500** | **-0.86695000** | **-5.74727500** |
| **H** | **4.18013000** | **-1.64033800** | **-7.12030400** |
| **H** | **-0.56222100** | **-1.69289400** | **-8.13166700** |
| **H** | **-2.90095200** | **-1.14370800** | **-7.55905500** |
| **H** | **-6.24406900** | **0.48657700** | **-4.47929400** |
| **H** | **-6.98846800** | **1.01027200** | **-2.19443600** |
| **H** | **6.94176500** | **0.36979300** | **3.85510900** |
| **H** | **6.59816300** | **1.76098000** | **1.83025600** |
| **H** | **4.27743700** | **2.27139100** | **1.07340600** |
| **H** | **2.35213100** | **1.36832300** | **2.26569600** |
| **H** | **2.35213100** | **1.36832300** | **-2.26569600** |
| **H** | **4.27743700** | **2.27139100** | **-1.07340600** |
| **H** | **6.59816300** | **1.76098000** | **-1.83025600** |
| **H** | **6.94176500** | **0.36979300** | **-3.85510900** |
| **Si** | **-2.67921300** | **0.28935700** | **0.00000000** |

Transition State of Phosphore Doped Expanded [13]-helicene:

X Y Z

**-----------------------------------------------------------------**

| **C** | **-5.10351700** | **0.84619700** | **1.24870800** |
| --- | --- | --- | --- |
| **C** | **-3.71954000** | **0.51005000** | **1.39608300** |
| **C** | **-3.71954000** | **0.51005000** | **-1.39608300** |
| **C** | **-5.10351700** | **0.84619700** | **-1.24870800** |
| **C** | **-5.69981500** | **1.03679600** | **0.00000000** |
| **C** | **-3.21670100** | **0.25068200** | **2.75410400** |
| **C** | **-3.21670100** | **0.25068200** | **-2.75410400** |
| **C** | **-4.15692200** | **0.18829100** | **3.83742700** |
| **C** | **-3.73713300** | **-0.26172600** | **5.09073000** |
| **C** | **-2.40903300** | **-0.59251400** | **5.35363000** |
| **C** | **-1.42349000** | **-0.35928200** | **4.33846400** |

| **C** | **-1.86174700** | **0.01939800** | **3.06052500** |
| --- | --- | --- | --- |
| **C** | **-1.86174700** | **0.01939800** | **-3.06052500** |
| **C** | **-1.42349000** | **-0.35928200** | **-4.33846400** |
| **C** | **-2.40903300** | **-0.59251400** | **-5.35363000** |
| **C** | **-3.73713300** | **-0.26172600** | **-5.09073000** |
| **C** | **-4.15692200** | **0.18829100** | **-3.83742700** |
| **C** | **-0.01274700** | **-0.54775300** | **4.69517500** |
| **C** | **-0.01274700** | **-0.54775300** | **-4.69517500** |
| **C** | **0.30226000** | **-1.19987600** | **5.93480700** |
| **C** | **1.63640800** | **-1.45549200** | **6.25081700** |
| **C** | **2.69037500** | **-1.00209900** | **5.45046200** |
| **C** | **2.39287800** | **-0.22610400** | **4.28354100** |
| **C** | **1.04552300** | **-0.06706600** | **3.91704900** |
| **C** | **1.04552300** | **-0.06706600** | **-3.91704900** |
| **C** | **2.39287800** | **-0.22610400** | **-4.28354100** |
| **C** | **2.69037500** | **-1.00209900** | **-5.45046200** |
| **C** | **1.63640800** | **-1.45549200** | **-6.25081700** |
| **C** | **0.30226000** | **-1.19987600** | **-5.93480700** |
| **C** | **3.49826300** | **0.39285700** | **3.55709700** |
| **C** | **4.83835400** | **0.11110500** | **3.95895700** |
| **C** | **4.83835400** | **0.11110500** | **-3.95895700** |
| **C** | **3.49826300** | **0.39285700** | **-3.55709700** |
| **C** | **-5.96735000** | **0.91922000** | **2.40299400** |
| **C** | **-5.52632200** | **0.56648800** | **3.63132100** |
| **C** | **-2.03327300** | **-1.18413200** | **6.61118600** |
| **C** | **-0.74737800** | **-1.52765800** | **6.86429000** |
| **C** | **4.05657400** | **-1.27027200** | **5.80522900** |
| **C** | **5.08212600** | **-0.75704900** | **5.07888100** |
| **C** | **5.08212600** | **-0.75704900** | **-5.07888100** |
| **C** | **4.05657400** | **-1.27027200** | **-5.80522900** |
| **C** | **-0.74737800** | **-1.52765800** | **-6.86429000** |
| **C** | **-2.03327300** | **-1.18413200** | **-6.61118600** |
| **C** | **-5.52632200** | **0.56648800** | **-3.63132100** |
| **C** | **-5.96735000** | **0.91922000** | **-2.40299400** |
| **C** | **5.91582000** | **0.72156200** | **3.28000300** |
| **C** | **5.69447500** | **1.61938400** | **2.25324400** |
| **C** | **4.37577400** | **1.92310000** | **1.87461800** |
| **C** | **3.30474700** | **1.31328900** | **2.50397800** |
| **C** | **3.30474700** | **1.31328900** | **-2.50397800** |
| **C** | **4.37577400** | **1.92310000** | **-1.87461800** |
| **C** | **5.69447500** | **1.61938400** | **-2.25324400** |
| **C** | **5.91582000** | **0.72156200** | **-3.28000300** |
| **H** | **-6.75853300** | **1.28835200** | **0.00000000** |
| **H** | **-4.47620500** | **-0.37434900** | **5.88138200** |
| **H** | **-1.13189700** | **0.10737400** | **2.26232600** |
| **H** | **-1.13189700** | **0.10737400** | **-2.26232600** |
| **H** | **-4.47620500** | **-0.37434900** | **-5.88138200** |
| **H** | **1.86676800** | **-1.99097900** | **7.16969800** |
| **H** | **0.80993700** | **0.48363000** | **3.01406000** |
| **H** | **0.80993700** | **0.48363000** | **-3.01406000** |
| **H** | **1.86676800** | **-1.99097900** | **-7.16969800** |
| **H** | **-7.00258800** | **1.21119900** | **2.24632900** |
| **H** | **-6.19680000** | **0.56375800** | **4.48709900** |
| **H** | **-2.81656100** | **-1.38047400** | **7.33907800** |
| **H** | **-0.47342900** | **-2.01133400** | **7.79863000** |
| **H** | **4.24905800** | **-1.89327300** | **6.67527300** |
| **H** | **6.11341100** | **-0.96603700** | **5.35285200** |
| **H** | **6.11341100** | **-0.96603700** | **-5.35285200** |
| **H** | **4.24905800** | **-1.89327300** | **-6.67527300** |
| **H** | **-0.47342900** | **-2.01133400** | **-7.79863000** |
| **H** | **-2.81656100** | **-1.38047400** | **-7.33907800** |
| **H** | **-6.19680000** | **0.56375800** | **-4.48709900** |
| **H** | **-7.00258800** | **1.21119900** | **-2.24632900** |

| **H** | **6.92970000** | **0.48640100** | **3.59547300** |
| --- | --- | --- | --- |
| **H** | **6.53155700** | **2.09538700** | **1.75008900** |
| **H** | **4.19128000** | **2.65055100** | **1.09100300** |
| **H** | **2.29949300** | **1.57759000** | **2.19365300** |
| **H** | **2.29949300** | **1.57759000** | **-2.19365300** |
| **H** | **4.19128000** | **2.65055100** | **-1.09100300** |
| **H** | **6.53155700** | **2.09538700** | **-1.75008900** |
| **H** | **6.92970000** | **0.48640100** | **-3.59547300** |
| **P** | **-2.65674200** | **0.28031700** | **0.00000000** |

Transition State of Expanded [14]-helicene:

X Y Z

**-----------------------------------------------------------------**

| **C** | **-5.49531300** | **1.72407200** | **1.11402300** |
| --- | --- | --- | --- |
| **C** | **-4.15648400** | **1.58972600** | **0.61899700** |
| **C** | **-2.21281700** | **-3.84106400** | **-0.18753500** |
| **C** | **-3.51095200** | **2.77948700** | **0.05245900** |
| **C** | **-0.90357200** | **-4.22059700** | **-0.72494600** |
| **C** | **-4.33734100** | **3.90865500** | **-0.26632400** |
| **C** | **-3.80515700** | **4.94477700** | **-1.03742700** |
| **C** | **-2.45614900** | **4.98343400** | **-1.39380700** |
| **C** | **-1.57241500** | **3.99565000** | **-0.85102000** |
| **C** | **-2.13491800** | **2.88489100** | **-0.20174200** |
| **C** | **0.28060300** | **-3.51998600** | **-0.45088400** |
| **C** | **1.52619900** | **-3.91686300** | **-0.96018400** |
| **C** | **1.55584700** | **-4.97605200** | **-1.92295900** |
| **C** | **0.39341300** | **-5.71322600** | **-2.15361900** |
| **C** | **-0.81199300** | **-5.41474700** | **-1.51235900** |
| **C** | **-0.13197600** | **4.21881400** | **-0.98807700** |
| **C** | **2.80436500** | **-3.35510900** | **-0.50804800** |
| **C** | **0.32697400** | **5.21420100** | **-1.91396400** |
| **C** | **1.69900300** | **5.40796900** | **-2.07607600** |
| **C** | **2.64141500** | **4.75888900** | **-1.26976000** |
| **C** | **2.18580100** | **3.87097200** | **-0.24117700** |
| **C** | **0.81263700** | **3.58384600** | **-0.17555300** |
| **C** | **2.92066400** | **-2.55693800** | **0.62577300** |
| **C** | **4.16914700** | **-2.12539300** | **1.12007100** |
| **C** | **5.36477300** | **-2.46819700** | **0.40062500** |
| **C** | **5.24431400** | **-3.25686200** | **-0.75726600** |
| **C** | **4.01447300** | **-3.73381700** | **-1.19926800** |
| **C** | **3.15515800** | **3.36211600** | **0.72534400** |
| **C** | **4.53429200** | **3.68783400** | **0.56096300** |
| **C** | **-6.21774900** | **2.95735900** | **0.94285000** |
| **C** | **-5.69054400** | **3.97325400** | **0.21789500** |
| **C** | **-1.94619500** | **5.99326500** | **-2.28584000** |
| **C** | **-0.62595300** | **6.05867200** | **-2.58773300** |
| **C** | **4.04635600** | **5.02065800** | **-1.41818500** |
| **C** | **4.95131800** | **4.49619600** | **-0.55265600** |
| **C** | **6.70524500** | **-1.35558600** | **2.09753900** |
| **C** | **6.62539600** | **-2.04881000** | **0.91474700** |
| **C** | **3.94614700** | **-4.67513000** | **-2.29010600** |
| **C** | **2.78548400** | **-5.29761800** | **-2.60477900** |
| **C** | **-1.92649200** | **-6.32249000** | **-1.57755700** |
| **C** | **-3.05416800** | **-6.08685200** | **-0.86259700** |
| **C** | **5.47528500** | **3.24849700** | **1.51807700** |
| **C** | **5.08013200** | **2.52625300** | **2.62717000** |
| **C** | **3.72158600** | **2.20900800** | **2.79905900** |
| **C** | **2.78450200** | **2.61188100** | **1.86385400** |
| **H** | **-4.46121200** | **5.74993500** | **-1.36227300** |
| **H** | **-1.48106300** | **2.08039400** | **0.11999900** |
| **H** | **0.23259700** | **-2.67084800** | **0.22280400** |
| **H** | **0.43869600** | **-6.57572000** | **-2.81563600** |

| **H** | **2.04638600** | **6.13248900** | **-2.80999400** |
| --- | --- | --- | --- |
| **H** | **0.45489900** | **2.89356900** | **0.57932000** |
| **H** | **2.03664400** | **-2.29939200** | **1.20182800** |
| **H** | **6.14598700** | **-3.54909800** | **-1.29197700** |
| **H** | **-7.22789800** | **3.01979500** | **1.33972500** |
| **H** | **-6.26707400** | **4.87198200** | **0.01333600** |
| **H** | **-2.65451900** | **6.68632500** | **-2.73309000** |
| **H** | **-0.25113100** | **6.80062000** | **-3.28845600** |
| **H** | **4.36640500** | **5.66421700** | **-2.23403300** |
| **H** | **6.01196200** | **4.70704100** | **-0.66618500** |
| **H** | **7.67287700** | **-1.05305400** | **2.48875000** |
| **H** | **7.52566100** | **-2.30504300** | **0.36109100** |
| **H** | **4.86715900** | **-4.91995200** | **-2.81344000** |
| **H** | **2.75501700** | **-6.05699900** | **-3.38232200** |
| **H** | **-1.81543500** | **-7.23282500** | **-2.16138300** |
| **H** | **-3.86920500** | **-6.80623100** | **-0.85210800** |
| **H** | **6.52213700** | **3.50583800** | **1.37455700** |
| **H** | **1.74184800** | **2.36504200** | **2.03339100** |
| **C** | **4.29175900** | **-1.40042700** | **2.33952300** |
| **C** | **5.52531700** | **-1.03559900** | **2.82123300** |
| **H** | **3.40111100** | **1.65082500** | **3.67445600** |
| **H** | **3.38822000** | **-1.14903300** | **2.88938800** |
| **H** | **5.81301200** | **2.20889000** | **3.36321300** |
| **H** | **5.60474500** | **-0.49742900** | **3.76148400** |
| **C** | **-3.23663900** | **-4.84388200** | **-0.16061100** |
| **C** | **-4.43017000** | **-4.56660800** | **0.50494600** |
| **C** | **-4.72454800** | **-3.29550700** | **1.00093800** |
| **H** | **-5.17820100** | **-5.35145100** | **0.59741100** |
| **C** | **-2.53725900** | **-2.54671300** | **0.25333800** |
| **C** | **-3.80172900** | **-2.21808400** | **0.77512500** |
| **C** | **-5.97372500** | **-3.06055900** | **1.67414800** |
| **H** | **-1.79655200** | **-1.76270500** | **0.13150100** |
| **C** | **-4.27214500** | **-0.84592600** | **1.01769900** |
| **C** | **-6.36644400** | **-1.80428200** | **1.98758500** |
| **H** | **-6.60278800** | **-3.91736500** | **1.90231800** |
| **C** | **-5.57561500** | **-0.66868200** | **1.59316600** |
| **C** | **-3.58175300** | **0.30654300** | **0.59739500** |
| **H** | **-7.32006400** | **-1.62142500** | **2.47641200** |
| **C** | **-6.13526000** | **0.60827100** | **1.65179200** |
| **H** | **-2.60742200** | **0.19146700** | **0.13385200** |
| **H** | **-7.14558900** | **0.71863900** | **2.04043600** |

Transition State of Hydroxyl Functionalized Expanded [14]-helicene:

X Y Z

**-----------------------------------------------------------------**

| **C** | **5.40517900** | **-2.05277900** | **1.09130800** |
| --- | --- | --- | --- |
| **C** | **4.08067800** | **-1.82742600** | **0.59057800** |
| **C** | **2.50530000** | **3.72411000** | **-0.19376500** |
| **C** | **3.36081300** | **-2.96776100** | **0.01180800** |
| **C** | **1.22724100** | **4.19228400** | **-0.73617700** |
| **C** | **4.11295000** | **-4.14672400** | **-0.31082500** |
| **C** | **3.51783800** | **-5.14041100** | **-1.09141300** |
| **C** | **2.17146700** | **-5.08626000** | **-1.45650200** |
| **C** | **1.35174300** | **-4.04464200** | **-0.91318100** |
| **C** | **1.98260500** | **-2.97907600** | **-0.25062600** |
| **C** | **-0.00218000** | **3.57050200** | **-0.47153700** |
| **C** | **-1.21574100** | **4.05161400** | **-0.98525200** |
| **C** | **-1.17002100** | **5.11383500** | **-1.94388500** |
| **C** | **0.03981600** | **5.77370500** | **-2.16475600** |
| **C** | **1.21910600** | **5.39367500** | **-1.51792200** |
| **C** | **-0.09945300** | **-4.16700100** | **-1.06429800** |
| **C** | **-2.53070200** | **3.57529500** | **-0.54113600** |
| **C** | **-0.61457500** | **-5.12440100** | **-1.99582400** |
| **C** | **-1.99549600** | **-5.21315200** | **-2.16657100** |

| **C** | **-2.89266700** | **-4.50029300** | **-1.36407200** |
| --- | --- | --- | --- |
| **C** | **-2.39211200** | **-3.64891200** | **-0.31858600** |
| **C** | **-0.99737200** | **-3.46357300** | **-0.25284500** |
| **C** | **-2.70559700** | **2.78707000** | **0.59202100** |
| **C** | **-3.98248200** | **2.43836400** | **1.07870100** |
| **C** | **-5.14880400** | **2.85673900** | **0.35126400** |
| **C** | **-4.97020500** | **3.63634200** | **-0.80530600** |
| **C** | **-3.70926300** | **4.03307600** | **-1.23893600** |
| **C** | **-3.35114500** | **-3.08384000** | **0.63463200** |
| **C** | **-4.74870600** | **-3.32507000** | **0.43479600** |
| **C** | **6.04542600** | **-3.33013200** | **0.91565500** |
| **C** | **5.45592500** | **-4.30380900** | **0.18074500** |
| **C** | **1.60099200** | **-6.05595200** | **-2.35628700** |
| **C** | **0.28101800** | **-6.03112100** | **-2.66632900** |
| **C** | **-4.30541000** | **-4.66591100** | **-1.54907200** |
| **C** | **-5.18972800** | **-4.09013400** | **-0.69950800** |
| **C** | **-6.56975500** | **1.83255400** | **2.03883000** |
| **C** | **-6.43708500** | **2.51884500** | **0.85672700** |
| **C** | **-3.57268400** | **4.97026300** | **-2.32699000** |
| **C** | **-2.37188600** | **5.51696300** | **-2.63192100** |
| **C** | **2.39156600** | **6.22602100** | **-1.57221800** |
| **C** | **3.49683500** | **5.91270300** | **-0.85201900** |
| **C** | **-5.71357100** | **-2.86148100** | **1.35407300** |
| **C** | **-5.32976300** | **-2.18414500** | **2.49259200** |
| **C** | **-3.97164000** | **-1.93819600** | **2.71862200** |
| **C** | **-3.00545800** | **-2.35821200** | **1.81209600** |
| **H** | **4.12052600** | **-5.98612300** | **-1.41680900** |
| **H** | **1.37967300** | **-2.13806700** | **0.07650800** |
| **H** | **-0.01405900** | **2.71613800** | **0.19711800** |
| **H** | **0.05556300** | **6.63975200** | **-2.82348100** |
| **H** | **-2.39364900** | **-5.90333500** | **-2.90780300** |
| **H** | **-0.59832100** | **-2.80965000** | **0.50454000** |
| **H** | **-1.84325600** | **2.47248500** | **1.17260000** |
| **H** | **-5.84768400** | **3.98643900** | **-1.34543100** |
| **H** | **7.04705100** | **-3.46191300** | **1.31720100** |
| **H** | **5.97294000** | **-5.23743800** | **-0.02691800** |
| **H** | **2.26402800** | **-6.79367900** | **-2.80168500** |
| **H** | **-0.13910800** | **-6.74370400** | **-3.37198300** |
| **H** | **-4.64820300** | **-5.27872900** | **-2.37924300** |
| **H** | **-6.25951800** | **-4.22589100** | **-0.83617400** |
| **H** | **-7.55744200** | **1.59212200** | **2.42280300** |
| **H** | **-7.31524800** | **2.83145300** | **0.29662300** |
| **H** | **-4.47227800** | **5.27568800** | **-2.85565100** |
| **H** | **-2.28707700** | **6.27488900** | **-3.40685200** |
| **H** | **2.34456600** | **7.14446900** | **-2.15199100** |
| **H** | **4.35760400** | **6.57652500** | **-0.83327400** |
| **H** | **-6.76233400** | **-3.06642600** | **1.15815300** |
| **C** | **-4.16052200** | **1.72501300** | **2.29806100** |
| **C** | **-5.41787900** | **1.43929200** | **2.77144500** |
| **H** | **-3.65585700** | **-1.40501500** | **3.61390500** |
| **H** | **-3.27779900** | **1.42094500** | **2.85566900** |
| **H** | **-6.06819200** | **-1.84082100** | **3.21091000** |
| **H** | **-5.53867000** | **0.90839800** | **3.71157900** |
| **C** | **3.59277300** | **4.65701600** | **-0.15548500** |
| **C** | **4.76139200** | **4.29797000** | **0.51520100** |
| **C** | **4.96821900** | **3.00755200** | **1.00568600** |
| **H** | **5.55926500** | **5.03102700** | **0.61593100** |
| **C** | **2.74069800** | **2.40881400** | **0.24160400** |
| **C** | **3.97766200** | **1.99451800** | **0.76845200** |
| **C** | **6.19531500** | **2.68674700** | **1.68411600** |
| **H** | **1.95087300** | **1.67588300** | **0.11053800** |
| **C** | **4.35477900** | **0.59286000** | **1.00541400** |
| **C** | **6.50230700** | **1.40531600** | **1.99156700** |

| **H** | **6.87846500** | **3.49870600** | **1.92067800** |
| --- | --- | --- | --- |
| **C** | **5.64053500** | **0.32658900** | **1.58623700** |
| **C** | **3.59197500** | **-0.50897000** | **0.57453500** |
| **H** | **7.43920600** | **1.15702400** | **2.48394300** |
| **C** | **6.11432700** | **-0.98490800** | **1.63963300** |
| **H** | **2.62945100** | **-0.32789400** | **0.10749500** |
| **H** | **7.11314000** | **-1.16403000** | **2.03253500** |
| **O** | **-1.68643200** | **-2.08325400** | **2.06978900** |
| **H** | **-1.62967400** | **-1.64132700** | **2.93131300** |

Transition State of Methyl Functionalized Expanded [14]-helicene:

X Y Z

**-----------------------------------------------------------------**

| **C** | **5.22322800** | **-2.46399000** | **1.05803200** |
| --- | --- | --- | --- |
| **C** | **3.92147200** | **-2.12804200** | **0.55948000** |
| **C** | **2.80207800** | **3.54092500** | **-0.18290500** |
| **C** | **3.10922400** | **-3.20380300** | **-0.02103000** |
| **C** | **1.56351700** | **4.11548400** | **-0.71457000** |
| **C** | **3.76153600** | **-4.44001700** | **-0.34684300** |
| **C** | **3.08589100** | **-5.37934000** | **-1.12873200** |
| **C** | **1.74791700** | **-5.21533800** | **-1.49119600** |
| **C** | **1.01585300** | **-4.11156600** | **-0.94426300** |
| **C** | **1.73398300** | **-3.10240900** | **-0.28162200** |
| **C** | **0.29073900** | **3.58293100** | **-0.46096800** |
| **C** | **-0.88395800** | **4.16560200** | **-0.95956000** |
| **C** | **-0.75932500** | **5.24697800** | **-1.88955400** |
| **C** | **0.49748900** | **5.81544000** | **-2.10207500** |
| **C** | **1.64637700** | **5.32953900** | **-1.47184600** |
| **C** | **-0.44140800** | **-4.12317200** | **-1.08930100** |
| **C** | **-2.23039400** | **3.77483000** | **-0.52567300** |
| **C** | **-1.03075400** | **-5.03174200** | **-2.02476400** |
| **C** | **-2.41204700** | **-4.99141900** | **-2.20857600** |
| **C** | **-3.25044100** | **-4.21390500** | **-1.40294100** |
| **C** | **-2.69292700** | **-3.45190000** | **-0.31597400** |
| **C** | **-1.28799100** | **-3.36517900** | **-0.26981400** |
| **C** | **-2.46265200** | **2.95592100** | **0.57509200** |
| **C** | **-3.76093800** | **2.68833500** | **1.05658900** |
| **C** | **-4.89335400** | **3.23146600** | **0.35844500** |
| **C** | **-4.65862900** | **4.03903700** | **-0.76838900** |
| **C** | **-3.37225900** | **4.34903900** | **-1.19794000** |
| **C** | **-3.60703700** | **-2.84935500** | **0.66699200** |
| **C** | **-5.00765100** | **-2.86086400** | **0.35466900** |
| **C** | **5.75591700** | **-3.78918300** | **0.87837200** |
| **C** | **5.08754100** | **-4.70911100** | **0.14204400** |
| **C** | **1.10135500** | **-6.13525000** | **-2.39153600** |
| **C** | **-0.21252500** | **-6.00468400** | **-2.70017200** |
| **C** | **-4.66197600** | **-4.19373700** | **-1.65253500** |
| **C** | **-5.49311500** | **-3.50479700** | **-0.83576500** |
| **C** | **-6.38244700** | **2.25843900** | **2.01643000** |
| **C** | **-6.20190900** | **2.98079500** | **0.86258600** |
| **C** | **-3.16770200** | **5.30763300** | **-2.25613600** |
| **C** | **-1.92886000** | **5.76449600** | **-2.55626400** |
| **C** | **2.88042900** | **6.06798700** | **-1.51763700** |
| **C** | **3.96117300** | **5.65508600** | **-0.81071000** |
| **C** | **-5.95622600** | **-2.30581100** | **1.23751000** |
| **C** | **-5.56426600** | **-1.78462900** | **2.45059800** |
| **C** | **-4.20877200** | **-1.82065600** | **2.79545700** |
| **C** | **-3.22583200** | **-2.33030000** | **1.94868500** |
| **H** | **3.61690400** | **-6.27054100** | **-1.45739600** |
| **H** | **1.20537900** | **-2.21278200** | **0.04641000** |
| **H** | **0.21512800** | **2.71809800** | **0.18961300** |
| **H** | **0.57779600** | **6.69360200** | **-2.73972200** |

| **H** | **-2.86227800** | **-5.61902100** | **-2.97512800** |
| --- | --- | --- | --- |
| **H** | **-0.82129200** | **-2.72225100** | **0.45610600** |
| **H** | **-1.62637500** | **2.54972700** | **1.13658400** |
| **H** | **-5.50789300** | **4.47903400** | **-1.28752500** |
| **H** | **6.74359400** | **-4.00402400** | **1.27853500** |
| **H** | **5.52516100** | **-5.68173300** | **-0.06880400** |
| **H** | **1.70355000** | **-6.92216300** | **-2.83900900** |
| **H** | **-0.68926500** | **-6.67801800** | **-3.40821800** |
| **H** | **-5.04292600** | **-4.73477300** | **-2.51512300** |
| **H** | **-6.56196500** | **-3.47119400** | **-1.03148500** |
| **H** | **-7.38397700** | **2.08569500** | **2.40119300** |
| **H** | **-7.05476000** | **3.38915800** | **0.32538800** |
| **H** | **-4.04331300** | **5.70093100** | **-2.76674000** |
| **H** | **-1.78824600** | **6.53618900** | **-3.30917300** |
| **H** | **2.90317600** | **6.99809600** | **-2.08003000** |
| **H** | **4.87183300** | **6.24836000** | **-0.78558600** |
| **H** | **-7.00530800** | **-2.32482500** | **0.95312000** |
| **C** | **-3.98865600** | **1.93633400** | **2.24426800** |
| **C** | **-5.26235900** | **1.73732000** | **2.71801300** |
| **H** | **-3.90463200** | **-1.45019000** | **3.77130100** |
| **H** | **-3.13260200** | **1.52954900** | **2.77697600** |
| **H** | **-6.29432200** | **-1.37379700** | **3.14188900** |
| **H** | **-5.41958000** | **1.17569800** | **3.63409700** |
| **C** | **3.96003500** | **4.38434500** | **-0.13527900** |
| **C** | **5.09838100** | **3.92474600** | **0.52600200** |
| **C** | **5.20266200** | **2.61649700** | **1.00128500** |
| **H** | **5.95204600** | **4.59093800** | **0.63337600** |
| **C** | **2.93328300** | **2.20609100** | **0.23648000** |
| **C** | **4.13326200** | **1.68892700** | **0.75728700** |
| **C** | **6.40127700** | **2.19170100** | **1.67326400** |
| **H** | **2.08727700** | **1.54001200** | **0.09989900** |
| **C** | **4.39516800** | **0.25968100** | **0.98402500** |
| **C** | **6.60355100** | **0.88746500** | **1.97115200** |
| **H** | **7.14857200** | **2.94366400** | **1.91410000** |
| **C** | **5.65534400** | **-0.11430100** | **1.56149900** |
| **C** | **3.54377800** | **-0.77360600** | **0.54904200** |
| **H** | **7.51786600** | **0.56009800** | **2.45965900** |
| **C** | **6.01913900** | **-1.46059700** | **1.60869900** |
| **H** | **2.59950300** | **-0.51088600** | **0.08347100** |
| **H** | **7.00003700** | **-1.72341400** | **1.99954100** |
| **C** | **-1.82071400** | **-2.34551400** | **2.51619400** |
| **H** | **-1.33388100** | **-3.31907300** | **2.39906300** |
| **H** | **-1.16810900** | **-1.59293000** | **2.05544000** |
| **H** | **-1.85397000** | **-2.12036800** | **3.58646200** |

Transition State of Methoxy Functionalized Expanded [14]-helicene:

X Y Z

**-----------------------------------------------------------------**

| **C** | **5.41397000** | **-2.06481300** | **1.03877500** |
| --- | --- | --- | --- |
| **C** | **4.09444000** | **-1.82742400** | **0.53052800** |
| **C** | **2.55417700** | **3.73946600** | **-0.21725100** |
| **C** | **3.37117900** | **-2.95900300** | **-0.06089600** |
| **C** | **1.28109600** | **4.21962900** | **-0.76092900** |
| **C** | **4.11663200** | **-4.14197200** | **-0.38424800** |
| **C** | **3.51977700** | **-5.12695300** | **-1.17478900** |
| **C** | **2.17689700** | **-5.06021100** | **-1.55071700** |
| **C** | **1.36133100** | **-4.01481900** | **-1.00848400** |
| **C** | **1.99531500** | **-2.95809000** | **-0.33525300** |
| **C** | **0.04632800** | **3.60521600** | **-0.50386000** |
| **C** | **-1.16184800** | **4.09731100** | **-1.01997000** |
| **C** | **-1.10481100** | **5.16321100** | **-1.97393800** |
| **C** | **0.11023900** | **5.81634300** | **-2.18609600** |

| **C** | **1.28417500** | **5.42529600** | **-1.53608400** |
| --- | --- | --- | --- |
| **C** | **-0.08940700** | **-4.12369600** | **-1.17294900** |
| **C** | **-2.48197500** | **3.62816900** | **-0.58358300** |
| **C** | **-0.60418200** | **-5.06710200** | **-2.11839300** |
| **C** | **-1.98346800** | **-5.13094800** | **-2.30950000** |
| **C** | **-2.88102900** | **-4.41202900** | **-1.51204200** |
| **C** | **-2.38461300** | **-3.58978500** | **-0.44013200** |
| **C** | **-0.98768900** | **-3.42099100** | **-0.36217700** |
| **C** | **-2.66765600** | **2.83904500** | **0.54722500** |
| **C** | **-3.94923600** | **2.49565800** | **1.02545000** |
| **C** | **-5.10895600** | **2.92041400** | **0.29116600** |
| **C** | **-4.91947500** | **3.70241700** | **-0.86208200** |
| **C** | **-3.65399900** | **4.09429100** | **-1.28682200** |
| **C** | **-3.35067700** | **-3.02908200** | **0.51077600** |
| **C** | **-4.74632000** | **-3.21393600** | **0.25638000** |
| **C** | **6.04645200** | **-3.34560500** | **0.85991400** |
| **C** | **5.45489500** | **-4.31126300** | **0.11600500** |
| **C** | **1.60549500** | **-6.01950000** | **-2.46130700** |
| **C** | **0.28901500** | **-5.97910100** | **-2.78489100** |
| **C** | **-4.29148300** | **-4.53520100** | **-1.73753500** |
| **C** | **-5.17962800** | **-3.93965800** | **-0.90619300** |
| **C** | **-6.54515800** | **1.89684400** | **1.96612000** |
| **C** | **-6.40192400** | **2.58600800** | **0.78691100** |
| **C** | **-3.50610500** | **5.03473500** | **-2.37058100** |
| **C** | **-2.30069800** | **5.57624900** | **-2.66652300** |
| **C** | **2.46223000** | **6.25029300** | **-1.58062800** |
| **C** | **3.56244900** | **5.92569300** | **-0.85767700** |
| **C** | **-5.72633000** | **-2.73854600** | **1.15469900** |
| **C** | **-5.35667700** | **-2.11242800** | **2.32358200** |
| **C** | **-3.99841400** | **-1.93318300** | **2.61804500** |
| **C** | **-3.01395400** | **-2.36597100** | **1.73575400** |
| **H** | **4.11857800** | **-5.97547100** | **-1.50009100** |
| **H** | **1.39638500** | **-2.11303700** | **-0.01108600** |
| **H** | **0.02575000** | **2.74761100** | **0.16046800** |
| **H** | **0.13458300** | **6.68544200** | **-2.84052700** |
| **H** | **-2.38247400** | **-5.80208200** | **-3.06759500** |
| **H** | **-0.58985700** | **-2.77609000** | **0.40245900** |
| **H** | **-1.81039800** | **2.51918900** | **1.13246100** |
| **H** | **-5.79191800** | **4.05818400** | **-1.40665500** |
| **H** | **7.04448800** | **-3.48665100** | **1.26721000** |
| **H** | **5.96679600** | **-5.24739000** | **-0.09310800** |
| **H** | **2.26556400** | **-6.76114700** | **-2.90459400** |
| **H** | **-0.13073900** | **-6.68257700** | **-3.49987100** |
| **H** | **-4.62927800** | **-5.12462800** | **-2.58646000** |
| **H** | **-6.24909300** | **-4.03427700** | **-1.07624100** |
| **H** | **-7.53637200** | **1.65874100** | **2.34243100** |
| **H** | **-7.27506900** | **2.90351200** | **0.22172700** |
| **H** | **-4.40101300** | **5.34705700** | **-2.90316100** |
| **H** | **-2.20741500** | **6.33682700** | **-3.43787200** |
| **H** | **2.42357300** | **7.17241500** | **-2.15516800** |
| **H** | **4.42733900** | **6.58386600** | **-0.83132000** |
| **H** | **-6.77441500** | **-2.89611000** | **0.91555000** |
| **C** | **-4.13826200** | **1.78086900** | **2.24230500** |
| **C** | **-5.39983100** | **1.49789100** | **2.70594800** |
| **H** | **-3.72096500** | **-1.44625100** | **3.54423000** |
| **H** | **-3.26090500** | **1.47386900** | **2.80675100** |
| **H** | **-6.10741800** | **-1.75786600** | **3.02378000** |
| **H** | **-5.52866700** | **0.96473700** | **3.64373800** |
| **C** | **3.64753900** | **4.66502800** | **-0.16876100** |
| **C** | **4.81120400** | **4.29404600** | **0.50408400** |
| **C** | **5.00802700** | **2.99896100** | **0.98633000** |
| **H** | **5.61318600** | **5.02140400** | **0.61314400** |
| **C** | **2.77985100** | **2.41956600** | **0.20932700** |

| **C** | **4.01237000** | **1.99378100** | **0.73750500** |
| --- | --- | --- | --- |
| **C** | **6.22993500** | **2.66578600** | **1.66819400** |
| **H** | **1.98585200** | **1.69265100** | **0.07005600** |
| **C** | **4.38037400** | **0.58816700** | **0.96528700** |
| **C** | **6.52744800** | **1.38043600** | **1.96861200** |
| **H** | **6.91695500** | **3.47183700** | **1.91358700** |
| **C** | **5.66122900** | **0.30981600** | **1.55136900** |
| **C** | **3.61397900** | **-0.50577100** | **0.52101100** |
| **H** | **7.46030500** | **1.12305700** | **2.46398300** |
| **C** | **6.12645800** | **-1.00501000** | **1.59847500** |
| **H** | **2.65626100** | **-0.31494300** | **0.04794800** |
| **H** | **7.12174400** | **-1.19307600** | **1.99613400** |
| **O** | **-1.68598800** | **-2.19703400** | **2.01737800** |
| **C** | **-1.30054900** | **-1.67155800** | **3.27701100** |
| **H** | **-0.20929200** | **-1.69715600** | **3.28645400** |
| **H** | **-1.63878800** | **-0.63485700** | **3.40479400** |
| **H** | **-1.68628100** | **-2.28242300** | **4.10284500** |

Transition State of Amino Functionalized Expanded [14]-helicene:

X Y Z

**-----------------------------------------------------------------**

| **C** | **5.29496500** | **-2.30248500** | **1.06373500** |
| --- | --- | --- | --- |
| **C** | **3.98346600** | **-2.00780800** | **0.56469300** |
| **C** | **2.69070100** | **3.62255600** | **-0.18387900** |
| **C** | **3.20724000** | **-3.10808400** | **-0.01947700** |
| **C** | **1.43692000** | **4.15938900** | **-0.71984700** |
| **C** | **3.90073900** | **-4.32072100** | **-0.34986200** |
| **C** | **3.25685700** | **-5.27980500** | **-1.13423100** |
| **C** | **1.91367000** | **-5.16004300** | **-1.49549500** |
| **C** | **1.14569000** | **-4.08248900** | **-0.94531800** |
| **C** | **1.82917400** | **-3.05178600** | **-0.27839900** |
| **C** | **0.17942000** | **3.59119800** | **-0.46691500** |
| **C** | **-1.01106000** | **4.14099700** | **-0.96598900** |
| **C** | **-0.91611300** | **5.22504000** | **-1.89645900** |
| **C** | **0.32475100** | **5.82677500** | **-2.11068600** |
| **C** | **1.48632200** | **5.37391200** | **-1.47937700** |
| **C** | **-0.31054200** | **-4.14285000** | **-1.08713700** |
| **C** | **-2.34643000** | **3.71583800** | **-0.52957100** |
| **C** | **-0.87164600** | **-5.07224200** | **-2.02070600** |
| **C** | **-2.25474000** | **-5.07828000** | **-2.20702500** |
| **C** | **-3.11875200** | **-4.33001900** | **-1.40208000** |
| **C** | **-2.58204700** | **-3.55770300** | **-0.31370500** |
| **C** | **-1.18070500** | **-3.41356800** | **-0.26506000** |
| **C** | **-2.55534600** | **2.88687700** | **0.56835400** |
| **C** | **-3.84506100** | **2.59190900** | **1.05681800** |
| **C** | **-4.99259300** | **3.11627300** | **0.36921200** |
| **C** | **-4.78131200** | **3.92980100** | **-0.75789600** |
| **C** | **-3.50413000** | **4.26634100** | **-1.19475400** |
| **C** | **-3.51315500** | **-2.98553000** | **0.66237500** |
| **C** | **-4.91079500** | **-2.99432000** | **0.35068600** |
| **C** | **5.87130400** | **-3.60880000** | **0.88000500** |
| **C** | **5.23469600** | **-4.54743000** | **0.13916100** |
| **C** | **1.29690900** | **-6.10123700** | **-2.39433400** |
| **C** | **-0.02218200** | **-6.01658900** | **-2.69757700** |
| **C** | **-4.53200500** | **-4.33510700** | **-1.64969300** |
| **C** | **-5.37750300** | **-3.65100500** | **-0.84142500** |
| **C** | **-6.45045700** | **2.11411900** | **2.03736500** |
| **C** | **-6.29235500** | **2.84150800** | **0.88339100** |
| **C** | **-3.32641400** | **5.22898100** | **-2.25404900** |
| **C** | **-2.09971600** | **5.71354100** | **-2.56017800** |
| **C** | **2.69851000** | **6.14769300** | **-1.52487700** |
| **C** | **3.78898300** | **5.76858700** | **-0.81410800** |

| **C** | **-5.85836700** | **-2.42779700** | **1.22595000** |
| --- | --- | --- | --- |
| **C** | **-5.45618100** | **-1.89940200** | **2.43671700** |
| **C** | **-4.10985300** | **-1.95532000** | **2.80244000** |
| **C** | **-3.13849500** | **-2.49489000** | **1.95535600** |
| **H** | **3.81712500** | **-6.15216400** | **-1.46497300** |
| **H** | **1.27013500** | **-2.18344200** | **0.05621400** |
| **H** | **0.12768000** | **2.72543300** | **0.18473700** |
| **H** | **0.38076800** | **6.70633400** | **-2.74902700** |
| **H** | **-2.68120100** | **-5.71697400** | **-2.97813100** |
| **H** | **-0.75291300** | **-2.72379100** | **0.44845700** |
| **H** | **-1.70826500** | **2.49279700** | **1.12196900** |
| **H** | **-5.64243000** | **4.35345600** | **-1.27109600** |
| **H** | **6.86528200** | **-3.79221100** | **1.28028600** |
| **H** | **5.70472100** | **-5.50405600** | **-0.07524600** |
[truncated: 124,216 more chars]
